# Supplementary material for: Catching the Wave: Detecting Strain-Specific SARS-CoV-2 Peptides in Clinical Samples Collected during Infection Waves from Diverse Geographical Locations
Source: Viruses. 2022 Oct 7;14(10):2205. doi: 10.3390/v14102205 (PMC9609567; doi:10.3390/v14102205)
Supplement: Supplementary file 1 [file viruses-14-02205-s001.zip › Supplementary Data S1.pdf]

Supplementary TableS1: Sequence database search parameters for SearchGUI for the analysis of clinical datasets in Galaxy. Parameters were based upon data analysis protocols detailed in the original publications that generated the data used in our study.

Dataset Information and Search parameters	PXD019423	PXD019686	PXD020394	PXD021328	PXD022085	PXD023016	PXD024967	PXD025214	PXD026795	PXD034582
Geographical location	Germany	France	Uruguay	Brazil	China	India	USA	Brazil	USA	Brazil
Instrument used	Orbitrap Fusion Tribrid mass spectrometer	Q-Exactive HF tandem mass spectrometer	Q Exactive Plus (Q-Orbitrap) (Thermo) mass spectrometer	Q-Exactive HF-X mass spectrometer	Q Exactive HF mass spectrometer	Orbitrap Fusion Mass Spectrometer	Orbitrap Fusion Lumos Mass spectrometer	Q-Exactive HF-X mass spectrometer	Orbitrap Exploris 480 mass spectrometer	Q-Exactive HF-X mass spectrometer
Algorithms	X!Tandem, MS-GF+	X!Tandem, MS-GF+	X!Tandem, MS-GF+	X!Tandem, MS-GF+	X!Tandem, MS-GF+	X!Tandem, MS-GF+	X!Tandem, MS-GF+	X!Tandem, MS-GF+	X!Tandem, MS-GF+	X!Tandem, MS-GF+
Digestion Enzymes	Trypsin	Trypsin	Trypsin	Trypsin	Trypsin	Trypsin	Trypsin	Trypsin	Trypsin	Trypsin
Missed cleavages	2	2	2	2	2	2	2	2	2	2
Precursor Ion Tolerance	10 ppm	5 ppm	10 ppm	20 ppm	10 ppm	10 ppm	10 ppm	20 ppm	10 ppm	5 ppm
Fragment Tolerance	0.05 Da	0.02 Da	0.05 Da	20 ppm	0.05 Da	0.05 Da	0.02 Da	20 ppm	0.02 Da	20 ppm
Minimum Charge	2	2	2	2	2	2	2	2	2	2
Maximum Charge	6	6	6	6	6	6	6	6	6	6
Fixed Modifications	Carbamidomethylation of C	Carbamidomethylation of C	Carbamidomethylation of C	-	Carbamidomethylation of C	Carbamidomethylation of C	Carbamidomethylation of C	Carbamidomethylation of C	Carbamidomethylation of C	Carbamidomethylation of C
Variable Modifications	Deamidation of N, Oxidation of M	Deamidation of N, Deamidation of Q, Oxidation of M	Oxidation of M	Acetylation of protein N-term, Oxidation of M	Acetylation of protein N-term, Oxidation of M	Oxidation of M	TMT-10plex of peptide N-term, TMT 10plex of Protein N term, Oxidation of M	Acetylation of protein N-term, Oxidation of M	Oxidation of M	Oxidation of M
Minimum Peptide Length	6	8	8	8	8	8	8	8	8	8
Maximum Peptide Length	30	60	60	60	60	60	60	60	60	60
Maximum Precursor Error	10 ppm	5 ppm	10 ppm	10 ppm	10 ppm	10 ppm	10 ppm	10 ppm	10 ppm	10 ppm

Supplementary Table S2: Parameters for the PepQuery search engine for the validation of clinical datasets in Galaxy.

Search Parameters	PXD019423	PXD019686	PXD020394	PXD021328	PXD022085	PXD023016	PXD024967	PXD025214	PXD026795	PXD034582
PepQuery Parameters	Carbamidomethylation of C	Carbamidomethylation of C	Carbamidomethylation of C		Carbamidomethylation of C	Carbamidomethylation of C	Carbamidomethylation of C	Carbamidomethylation of C	Carbamidomethylation of C	Carbamidomethylation of C
Fixed modification(s)	Oxidation of M	Oxidation of M	Oxidation of M	Oxidation of M	Oxidation of M, Acetylation of peptide N-terminus	Oxidation of M	Oxidation of M	Oxidation of M	Oxidation of M	Oxidation of M
Variable modification(s)	3	3	3	3	3	3	3	3	3	3
Max Modifications	True	True	True	True	True	True	True	True	True	True
Unrestricted modification?	True	True	True	True	True	True	True	True	True	True
Amino Acid substitutions?	10	10	10	10	10	10	10	10	10	10
Precursor tolerance	ppm	ppm	ppm	ppm	ppm	ppm	ppm	ppm	ppm	ppm
Precursor unit	0.05	0.05	0.05	0.05	0.05	0.05	0.05	0.05	0.05	0.05
Product tolerance (Da)	Trypsin	Trypsin	Trypsin	Trypsin	Trypsin	Trypsin	Trypsin	Trypsin	Trypsin	Trypsin
Digestion enzyme	2	2	2	2	2	2	2	2	2	2
Max missed cleavages	CID/HCD	CID/HCD	CID/HCD	CID/HCD	CID/HCD	CID/HCD	CID/HCD	CID/HCD	CID/HCD	CID/HCD
Fragmentation	HyperScore	HyperScore	HyperScore	HyperScore	HyperScore	HyperScore	HyperScore	HyperScore	HyperScore	HyperScore

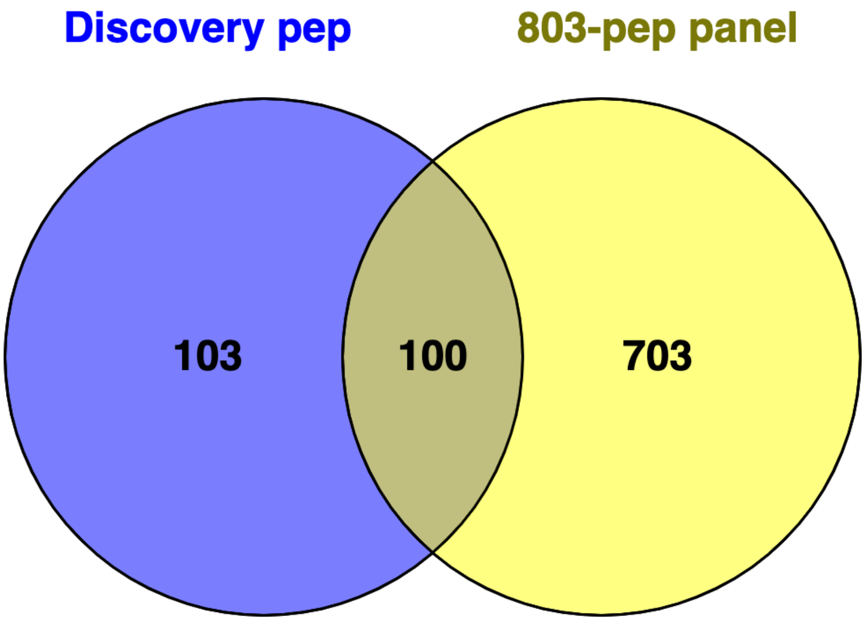

Supplementary Figure S1: Coverage of the 203 peptides obtained from the Discovery workflow to the 803 peptide panel from published data provides us with 100 unique peptides.

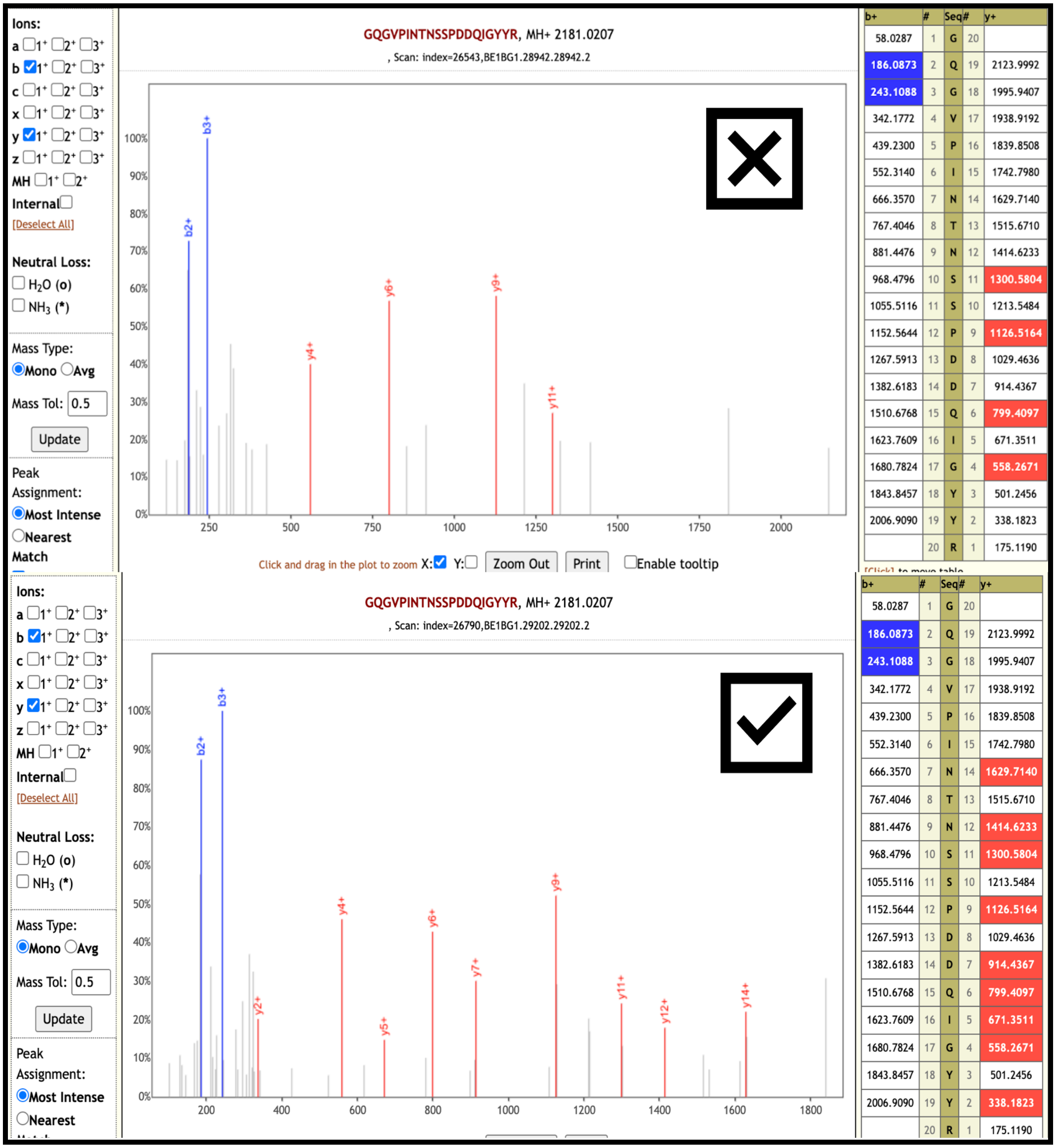

Supplementary Figure S2: Manual inspection of the spectra to validate peptide quality. The criteria for an accepted spectrum were that the spectra containing product ions should be at least a three-fold higher intensity than the noise level and the spectra should have at least three consecutive b- or y-ions in their series.

Supplementary Table S3: Pango lineage associated with the peptide sequences.

Peptide sequences	Peptide sequences (I=>L)	Matching status 1 = exact match 0.5 = M removed	Closest matching peptide sequence from GISAID allprot0203	Protein(s)	Pango Lineage	5first+5last countries	First Accession ID	First Pango lineage	Number of Accession IDs

		0.2 = non-trypsic	dated 2022/02/03						
GEGVPINTNSSPDDQIGYYR	GEGVPLNTNSSPDDQLGYYR	1	GEGVPLNTNSSPDDQLGYYR	{N}	[B.1.399, B.1.2, B.1.1.214, B.1.1.7, B.1.526, AY.122, B.1.617.2, AY.43, None, AY.3, AY.33, AY.4, AY.30, AY.103, BA.1]	USA_USA_USA_USA_Japan_USA_Wales_England_South Korea_USA	EPI_ISL_1924625	B.1.399	44
ITFGGPSDSTGSNQDGER	LTFGGPSDSTGSNQDGER	1	LTFGGPSDSTGSNQDGER	{N}	[B.1.1.448, B.4, B.1.1.7, B.1.351, B.1.1.214, B.1.429, AY.122, B.1.1.519, P.1.17, B.1.526, P.1.14, P.1, C.37, AY.4, AY.45, AY.54, AY.25, AY.4.3, AY.26, AY.110, AY.120, AY.44, AY.103, B.1.617.2, AY.129, AY.112, AY.34.1.1, AY.125, AY.100, AY.3, AY.39.1, AY.62, AY.46, AY.90, AY.7.1, AY.33, AY.121]	South Africa_England_England_England_England_Denmark_Poland_Denmark_Denmark_Germany	EPI_ISL_509346	B.1.1.448	477
MAGDGGDAALALLLLDR	MAGDGGDAALALLLLDR	1	MAGDGGDAALALLLLDR	{N}	[B.1.422, B.1.1.216, B.1.1.7, B.1.1.28, B.1, B.1.525, Q.1, P.1, AY.43, BA.1]	Canada_England_England_England_Brazil_South Africa_Germany_Germany_Germany_USA	EPI_ISL_586432	B.1.422	86
PGNGCDAALALLLLDR	PGNGCDAALALLLLDR	0.5	MPGNGCDAALALLLLDR	{N}	[AY.44, AY.120, AY.25, AY.103]	USA_USA_Sweden_Sweden_Sweden_USA_USA_USA_USA_USA	EPI_ISL_3844312	AY.44	15
SMGTSPTRMAGNGGDAALALLLLDR	SMGTSPTRMAGNGGDAALALLLLDR	0.2	NSTPGSSMGTSPTRMAGNGGDAALALLLLDR	{N}	[B.1.617.2, AY.9, AY.51, AY.9.2]	England_Portugal_Greece_Germany_Sweden_England_Portugal_Greece_Germany_Sweden	EPI_ISL_1790832	B.1.617.2	5
LVDPQIQLAVTR	LVDPQLQLAVTR	1	LVDPQLQLAVTR	{NSP16, N}	[None, B.4.2, B.1.1, B.1.1.101, B.1.595, B.1, B.1.2, B.1.564, B.1.580, B.1.221.1, B.1.177.56, B.1.525, B.1.214.2,	Italy_Saudi Arabia_England_Brazil_India_USA_USA_USA_USA_USA	EPI_ISL_414599	None	169

					B.1.400, B.1.36, B.1.617.1, B.1.1.7, Q.1, B.1.36.1, B.1.177.82, R.1, B.1.177, B.1.351, B.1.160, B.1.36.29, AY.125, B.1.147, P.1.15, AY.5, B.1.429, B.1.126, B.1.617.2, AY.122, AY.4, AY.44, AY.119, BA.1, BA.1.1]				
NSTPGSSMGTSPAR	NSTPGSSMGTSPAR	1	NSTPGSSMGTSPAR	{N}	[B.1.617.2, B.1, AY.26, B.1.617.1, AY.23, B.1.503, AY.34.1, B, B.1.2, AY.119, None, AY.39, B.1.441, B.1.36, B.1.282, AY.44, B.1.112, AY.3, AY.103, AY.25, B.1.189, AY.67, B.1.1.432, AY.25.1, B.1.459, B.1.497, AY.1, AY.118, AY.119.2, AY.100, AY.81, AY.24, AY.122, AY.62, AY.54, AY.33, AY.4, AY.3.1, AY.9.2, B.1.609, AY.45, AY.82, B.1.380, B.1.36.18, AY.29, AY.112, AY.129, AY.36, AY.43, AY.4.2.3, AY.125, AY.41, B.1.177, AY.16, AY.46.3, AY.42, AY.75, AY.98.1, B.1.36.8, B.1.177.75, AY.5, B.1.177.10, B.1.160, B.1.177.73, AY.17, B.1.1.306, B.1.234, AY.72, AY.113, B.1.177.18,	France_Switzerland_Switzerland_Switzerland_Switzerland_Poland_Poland_Poland_Poland_Poland	EPI_ISL_7360393	B.1.617.2	3854981

					B.1.1, AY.120.1, B.1.617.3, B.1.525, B.1.1.318, AY.9, AY.10, B.1.1.7, AY.98, AY.8, AY.46, AY.127, AY.70, AY.93, AY.46.1, AY.55, AY.111, AY.59, AY.13, AY.15, AY.6, AY.120, AY.61, AY.77, AY.87, AY.30, AY.7, AY.4.8, AY.106, AY.92, ...]				
GQGVPINTNSSR	GQGVPLNTNSSR	1	GQGVPLNTNSSR	{N}	[B.6.6, B.1.1.161, P.1, B.1, B.1.1.33, C.13, P.1.10, P.1.13, P.1.15, B.1.1.306, D.2, B.1.177, P.1.14, B.1.367, B.1.36, None, B.1.221, P.1.12, B.1.398, P.1.1, P.1.17, P.1.16, B.1.630, P.1.2, B.1.429, B.1.111, B.1.177.35, B.1.1, B.1.2, B.1.596, B.1.160, P.1.7.1, B.1.566, B.1.1.28, B.1.234, B.1.1.7, P.1.9, B.1.177.73, B.1.351, P.1.3, B.1.497, P.1.7, P.1.4, P.1.6, P.1.10.1, B, B.1.524, P.1.17.1, P.1.5, B.1.1.348, B.1.526, C.37, B.1.1.117, Q.8, AY.35, 0, B.1.623, B.1.1.507, P.1.8, B.1.575, B.1.399, B.1.1.54, P.1.11, B.1.533, B.1.1.519, P.1.10.2, P.7, P.4, B.1.1.222,	bat_Thailand_USA_England_USA_Germany_England_Brazil_England_Brazil	EPI_ISL_412977	B.6.6	112244

					AY.129, A.2.5, B.1.1.178, B.1.620, C.1.2, AY.68, B.1.1.265, C.36.3.1, A.2.5.3, B.1.1.307, B.1.1.317, B.1.1.121, AY.44, AY.43, B.1.1.409, B.1.617.2, AY.63, P.1.12.1, AY.39, AY.5, C.37.1, AY.112, AY.4.5, AY.47, AY.103, AY.39.1, AY.125, AY.124, AY.3, AY.26, AY.4, ...]				
ITFGGPSDSTGSNQNGGAR	LTFGGPSDSTGSNQNGGAR	1	LTFGGPSDSTGSNQNGGAR	{N}	[BA.1, BA.1.1, None, BA.2, B.1.1, BA.3, AY.43, AY.113, AY.44, 0, AY.45, B.1.617.2, AY.39, AY.23, AY.122, B.1.1.294, AY.127, AY.3, C.36.3, AY.75, AY.36, AY.126, AY.118, B.1.623, AY.100, AY.117, AY.103, AY.112, AY.125, AY.98.1, AY.25.1, AY.46.6, B.1, AY.4.7, AY.116, B.1.639, AY.20, AY.25, AY.98, P.1, AY.119, C.36.3.1, B.1.1.263, P.1.12, B.1.1.1, B.1.367, B, A, AY.79, AY.9.2, AY.1, B.1.619, C.37.1, AY.46, AY.4, B.1.1.161, AY.107, AY.121, AY.114]	England_Czech Republic_USA_USA_USA_Poland_Poland_Poland_Poland_Poland	EPI_ISL_8989562	BA.1	752651
AYETQALPQR	AYETQALPQR	1	AYETQALPQR	{N}	[B.1.1.514, B.1.177, AD.2, B.1.258.3, B.1.1.216, B.1.184, B.1.1, B.1.2, B.1.367, B,	Greece_England_Wales_England_india_Slovakia_Poland_Mexico_Poland_Poland	EPI_ISL_1096987	B.1.1.514	3935774

					B.1.617.2, AY.26, B.1.617.1, A.2, AY.23, B.1, A.5, A.11, B.1.400, B.1.110.3, AY.34.1, B.29, B.1.1.71, B.4.2, B.1.428, B.1.1.28, B.1.1.87, B.1.1.33, B.1.1.222, B.1.391, B.1.8, B.1.214, AY.119, B.1.1.464, B.1.1.339, B.1.390, B.1.279, B.1.335, B.1.1.409, B.1.620, B.1.1.521, A, B.1.1.366, B.1.600, B.1.195, B.1.1.86, B.1.162, C.36, B.1.36, B.1.1.25, AY.39, AE.1, B.1.443, B.1.1.368, B.1.1.326, B.1.1.37, B.1.243, B.1.1.301, B.1.22, B.1.1.306, AY.44, AY.3, AY.103, B.1.546, B.1.595, B.1.1.54, AY.25, B.1.450, AY.67, B.1.1.1, B.1.509, B.1.1.291, B.1.558, B.1.404, B.1.401, B.1.609, B.1.1.70, B.1.366, B.1.369, None, B.1.206, AY.25.1, B.1.556, B.1.570, B.1.567, B.1.36.35, B.1.497, B.1.36.19, B.1.305, B.1.1.288, B.1.459, AY.1, AY.118, B.1.1.284, AY.119.2, B.1.1.198, B.1.580, AY.100,				

					B.1.313, AY.81, ...]				
KAYETQALPQR	KAYETQALPQR	1	KAYETQALPQR	{N}	[B.1.258.3, B.1.367, B, B.1.177, AD.2, B.1.2, B.1.1.216, B.1.184, B.1.1.514, B.1.1, B.1.617.2, AY.26, B.1.617.1, A.2, AY.23, B.1, A.5, A.11, B.1.400, B.1.110.3, AY.34.1, B.29, B.1.1.71, B.4.2, B.1.428, B.1.1.28, B.1.1.87, B.1.1.33, B.1.1.222, B.1.391, B.1.8, B.1.214, AY.119, B.1.1.464, B.1.1.339, B.1.390, B.1.279, B.1.1.409, B.1.335, B.1.620, B.1.1.521, A, B.1.1.366, B.1.195, B.1.600, B.1.1.86, B.1.162, C.36, B.1.36, B.1.1.25, AE.1, AY.39, B.1.443, B.1.1.368, B.1.1.37, B.1.1.326, B.1.243, B.1.1.301, B.1.22, B.1.1.306, AY.44, AY.3, AY.103, B.1.595, B.1.546, B.1.1.54, AY.25, B.1.450, AY.67, B.1.1.291, B.1.1.1, B.1.509, B.1.558, B.1.401, B.1.404, B.1.609, B.1.1.70, B.1.366, B.1.369, None, B.1.206, AY.25.1, B.1.556, B.1.570, B.1.36.35, B.1.567,	England_England_France_England_Netherlands_Poland_Poland_Mexico_Poland_Poland	EPI_ISL_1637200	B.1.258.3	3930635

					B.1.36.19, B.1.497, B.1.305, B.1.1.288, B.1.459, AY.1, AY.118, B.1.1.284, AY.119.2, B.1.1.198, B.1.580, B.1.313, AY.100, AY.81, ...]				
QYNVTQAFGR	QYNVTQAFGR	1	QYNVTQAFGR	{N}	[B.1.177, D.2, B.1.240.1, B.1.240, None, B.6.6, B.1, B.1.210, B.1.36, B.1.36.17]	bat_bat_bat_pangolin_pangolin_bat_bat_bat_bat_bat	EPI_ISL_852604	B.1.177	27
GPEQTQGNFGDQELTR	GPEQTQGNFGDQELTR	1	GPEQTQGNFGDQELTR	{N}	[B.1.1.33, B.1.1, N.2, None, N.6, B.1, N.1, N.4, N.3, N.5, B.1.499, C.27, N.7, N.8, B.1.91, B.1.1.332, B.1.2, B.1.139, AK.1, N.9, B.1.177.51, B.1.1.303, B.1.243, B.1.1.70, B.1.258, C.16, B.1.1.362, B.1.160.16, B.1.1.7, N.10, BA.1, B.1.429, B.1.1.448, B.4, B.1.160, B.1.1.393, B.1.177, B.1.36.18, B.1.221, B.1.517, P.1, B.1.1.294, BA.1.1, B.1.177.75, B.1.177.44, B.1.609, B.1.526, B.1.404, B.1.351, B.1.1.369, B.1.1.318, AZ.5, P.1.1, P.1.12, B.1.617.2, AY.4, B.1.466.2, AY.3, AY.117, B.1.1.1, AY.2, AY.98.1, AY.44, AY.26, AY.47, AY.20, AY.29, AY.13, AY.68, AY.75, AY.25.1, AY.43, B.1.621, AY.39.1, AY.122,	Brazil_Brazil_Brazil_Brazil_Brazil_England_England_England_Denmark_England	EPI_ISL_1181624	B.1.1.33	4804

					AY.25, AY.39, AY.103, AY.42, AY.102, AY.9.2, AY.73, AY.126, AY.99, AY.112, AY.46.2, AY.46.1, AY.23, AY.34.2, AY.98, AY.4.2.2, AY.43.3, AY.121.1, AY.4.2, AY.121, BA.2]				
RGPEQTQG NFGDQELTR	RGPEQTQG NFGDQELTR	1	RGPEQTQG NFGDQELTR	{N}	[B.1.1.33, B.1.1, N.2, None, N.6, B.1, N.1, N.4, N.3, N.5, B.1.499, C.27, N.7, N.8, B.1.91, B.1.1.332, B.1.2, B.1.139, AK.1, N.9, B.1.177.51, B.1.1.303, B.1.243, B.1.1.70, B.1.258, C.16, B.1.1.362, B.1.160.16, B.1.1.7, N.10, BA.1, B.1.429, B.1.1.448, B.4, B.1.160, B.1.1.393, B.1.177, B.1.36.18, B.1.221, B.1.517, P.1, B.1.1.294, BA.1.1, B.1.177.75, B.1.177.44, B.1.609, B.1.526, B.1.404, B.1.351, B.1.1.369, B.1.1.318, AZ.5, P.1.1, P.1.12, B.1.617.2, AY.4, B.1.466.2, AY.3, AY.117, B.1.1.1, AY.2, AY.98.1, AY.44, AY.26, AY.47, AY.20, AY.29, AY.13, AY.68, AY.75, AY.25.1, AY.43, B.1.621, AY.39.1, AY.122, AY.25, AY.39, AY.103, AY.42,	Brazil_Brazil_Brazil_Brazil_Brazil_England_England_England_Denmark_England	EPI_ISL_1181624	B.1.1.33	4804

					AY.102, AY.9.2, AY.73, AY.126, AY.99, AY.112, AY.46.2, AY.46.1, AY.23, AY.34.2, AY.98, AY.4.2.2, AY.43.3, AY.121.1, AY.4.2, AY.121, BA.2]				
IIWVATEGALNTPK	LLWVATEGALNTPK	1	LLWVATEGALNTPK	{N}	[B.1.2]	USA_USA	EPI_ISL_1080160	B.1.2	1
GQGVPINTNSSPDDQIGY	GQGVPLNTNSSPDDQLGY	1	GQGVPLNTNSSPDDQLGY	{N}	[B.1.177, B.1.1.7]	Sweden_Bulgaria_Sweden_Bulgaria	EPI_ISL_2408682	B.1.177	2
NTNSSPDDQIGYYR	NTNSSPDDQLGYYR	1	NTNSSPDDQLGYYR	{N}	[BA.1.1, BA.1, B.1.1.7, AY.122, AY.43, AY.93]	England_England_England_England_Spain_Germany_Germany_USA_USA	EPI_ISL_8242277	BA.1.1	36

Supplementary Table S4: Number of peptide-spectral matches (PSMs) and peptide intensities of the detected variant peptides from the 12 datasets. The PSM numbers reported here are PSMs that qualify after PepQuery analysis (PSM rank output) with a P-value of 0.05. The peptide intensity values reported in parentheses have been extracted from MaxQuant or FlashLFQ software outputs.

Variant peptides	PXD019423	PXD019686	PXD020394	PXD021328	PXD022085	PXD023016	PXD024967	PXD025214	PXD026795	PXD034582 (August2021)	PXD034582 (September 2021)	PXD034582 (January 2022)
GQGVPINTNSSR	-	-	-	-	**1**	-	-	**6 (5.4x10^{8})**	-	**1(1.2x10^{7})**	**1 (1.7x10^{6})**	-
AYETQALPQR	-	-	-	-	-	-	-	-	-	-	**6 (6x10^{8})**	-
GEGVPINTNSSPDDQIGYYR	-	-	-	-	-	-	-	-	-	**1(8.8x10^{5})**	**1**	**1**
SMGTSPTRMAGNGGDAALALLLLDR	-	-	-	**1 (1.1x10^{7})**	-	-	-	-	-	-	-	-
PGNGCDAALALLLLDR	-	-	-	**1**	-	-	-	**2**	-	-	-	-
ITFGGPSDSTGSNQNGGAR	-	-	-	-	-	-	-	-	-	-	-	**7 (8.3x10^{6})**
