# Supplementary material for: Catching the Wave: Detecting Strain-Specific SARS-CoV-2 Peptides in Clinical Samples Collected during Infection Waves from Diverse Geographical Locations
Source: Viruses. 2022 Oct 7;14(10):2205. doi: 10.3390/v14102205 (PMC9609567; doi:10.3390/v14102205)
Supplement: Supplementary file 1 [file viruses-14-02205-s001.zip › Supplementary Data S2.pdf]

Covid-19 paper

Spectrum (MVP)

PXD019423=0

PXD019686

KADETQALPQR

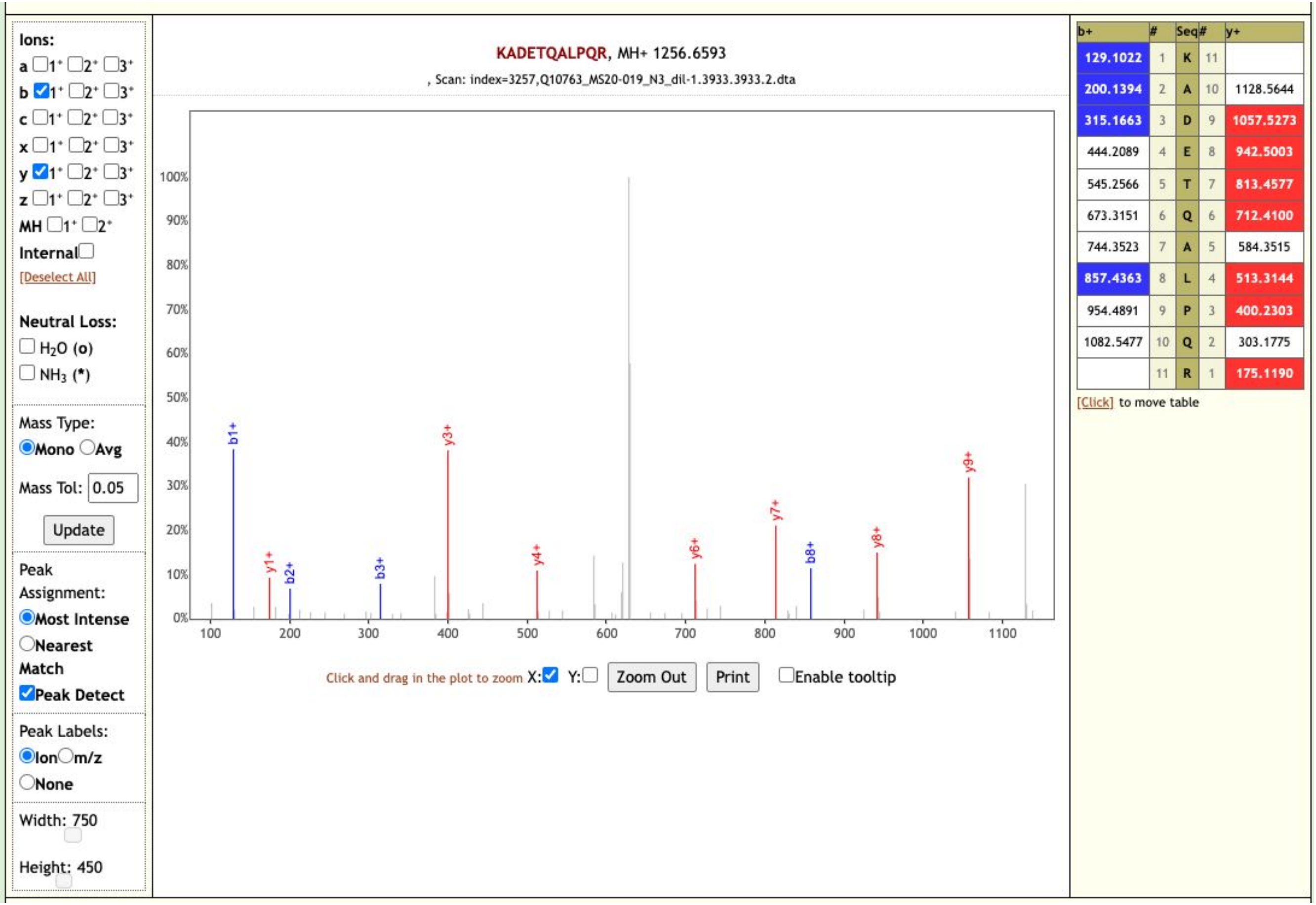

b+	#	Seq	#	y+
129.1022	1	K	11	
200.1394	2	A	10	1128.5644
315.1663	3	D	9	1057.5273
444.2089	4	E	8	942.5003
545.2566	5	T	7	813.4577
673.3151	6	Q	6	712.4100
744.3523	7	A	5	584.3515
857.4363	8	L	4	513.3144
954.4891	9	P	3	400.2303
1082.5477	10	Q	2	303.1775
	11	R	1	175.1190

KKADETQALPQR

Ions:
a 1+ 2+ 3+
b ☑1+ 2+ 3+
c 1+ 2+ 3+
x 1+ 2+ 3+
y ☑1+ 2+ 3+
z 1+ 2+ 3+
MH 1+ 2+
Internal
[Deselect All]

Neutral Loss:
H_2O (o)
NH_3 (*)

Mass Type:
Mono Avg

Mass Tol: 0.05

Update

Peak Assignment:
Most Intense
Nearest Match
☑Peak Detect

Peak Labels:
Ion m/z
None

Width: 750

Height: 450

KKADETQALPQR, MH+ 1384.7543

, Scan: index=1253,Q10761_MS20-019_N3_dil-2.1804.1804.3.dta

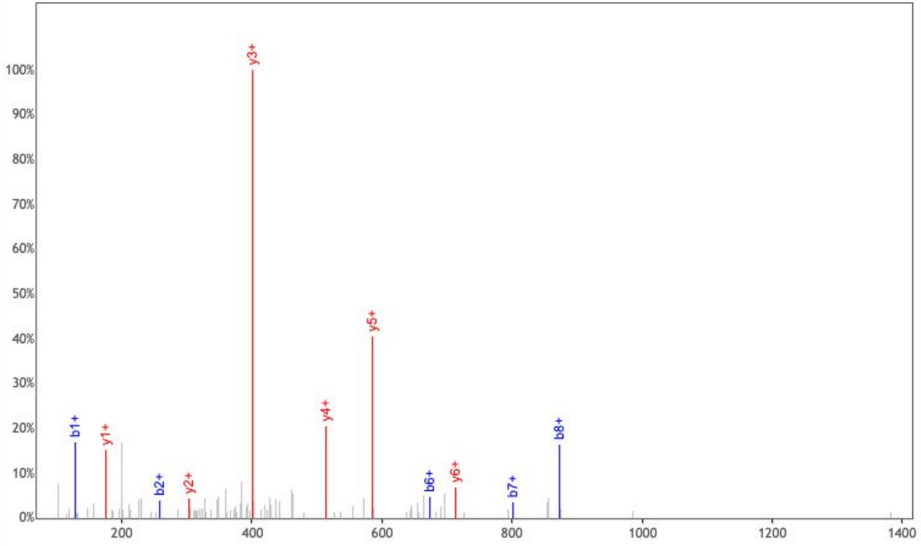

Click and drag in the plot to zoom X:☑ Y: Zoom Out Print Enable tooltip

b+	#	Seq	#	y+
129.1022	1	K	12	
257.1972	2	K	11	1256.6593
328.2343	3	A	10	1128.5644
443.2613	4	D	9	1057.5273
572.3039	5	E	8	942.5003
673.3515	6	T	7	813.4577
801.4101	7	Q	6	712.4100
872.4472	8	A	5	584.3515
985.5313	9	L	4	513.3144
1082.5840	10	P	3	400.2303
1210.6426	11	Q	2	303.1775
	12	R	1	175.1190

[Click] to move table

ADETQALPQR

Ions:
a 1+ 2+ 3+
b 1+ 2+ 3+
c 1+ 2+ 3+
x 1+ 2+ 3+
y 1+ 2+ 3+
z 1+ 2+ 3+
MH 1+ 2+
Internal
[Deselect All]

Neutral Loss:
H_2O (o)
NH_3 (*)

Mass Type:
Mono Avg

Mass Tol: 0.05

Update

Peak Assignment:
Most Intense
Nearest Match
Peak Detect

Peak Labels:
Ion m/z
None

Width: 750

Height: 450

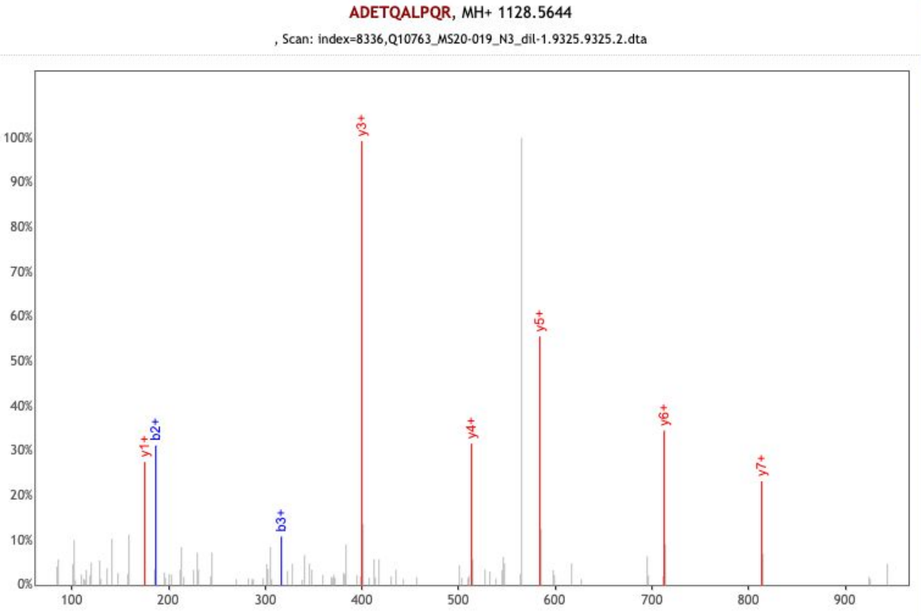

Click and drag in the plot to zoom X: Y: Zoom Out Print Enable tooltip

b+	#	Seq	#	y+
72.0444	1	A	10	
187.0713	2	D	9	1057.5273
316.1139	3	E	8	942.5003
417.1616	4	T	7	813.4577
545.2202	5	Q	6	712.4100
616.2573	6	A	5	584.3515
729.3414	7	L	4	513.3144
826.3941	8	P	3	400.2303
954.4527	9	Q	2	303.1775
	10	R	1	175.1190

[Click] to move table

GFYAEGSR

Ions:
a 1+ 2+ 3+
b 1+ 2+ 3+
c 1+ 2+ 3+
x 1+ 2+ 3+
y 1+ 2+ 3+
z 1+ 2+ 3+
MH 1+ 2+
Internal
[Deselect All]

Neutral Loss:
H_2O (o)
NH_3 (*)

Mass Type:
Mono Avg

Mass Tol: 0.05

Update

Peak Assignment:
Most Intense
Nearest Match
Peak Detect

Peak Labels:
Ion m/z
None

Width: 750

Height: 450

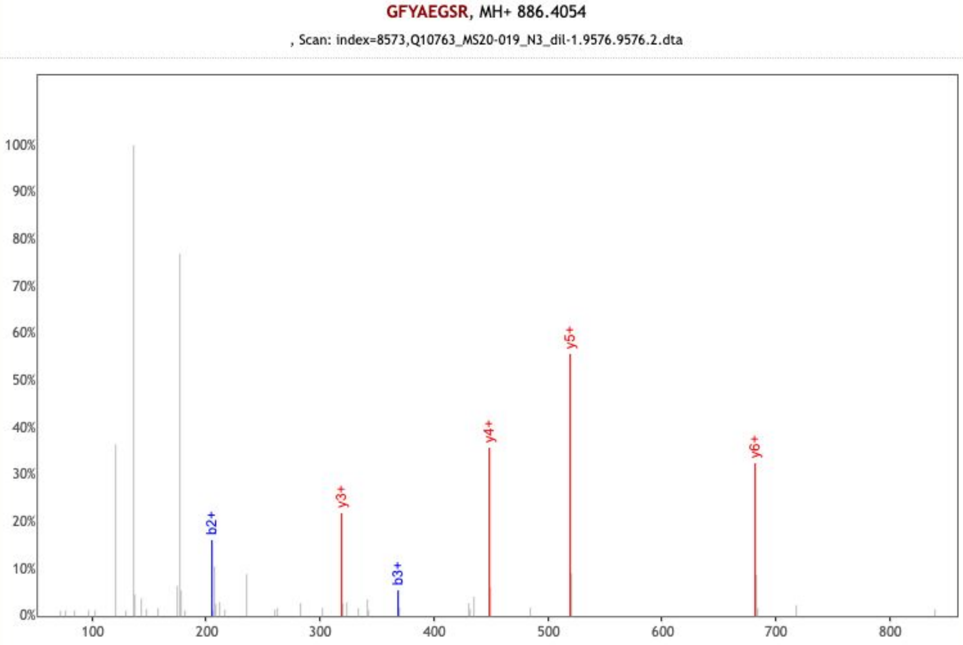

Click and drag in the plot to zoom X: Y: Zoom Out Print Enable tooltip

b+	#	Seq	#	y+
58.0287	1	G	8	
205.0972	2	F	7	829.3839
368.1605	3	Y	6	682.3155
439.1976	4	A	5	519.2522
568.2402	5	E	4	448.2150
625.2617	6	G	3	319.1724
712.2937	7	S	2	262.1510
	8	R	1	175.1190

[Click] to move table

EITVATSR

Ions:
a 1+ 2+ 3+
b ☑1+ 2+ 3+
c 1+ 2+ 3+
x 1+ 2+ 3+
y ☑1+ 2+ 3+
z 1+ 2+ 3+
MH 1+ 2+
Internal
[Deselect All]

Neutral Loss:
H_2O (o)
NH_3 (*)

Mass Type:
◉Mono ○Avg

Mass Tol: 0.05

Update

Peak Assignment:
◉Most Intense
○Nearest Match
☑Peak Detect

Peak Labels:
◉Ion ○m/z
○None

Width: 750

Height: 450

EITVATSR, MH+ 876.4785

, Scan: index=7437,Q10760_MS20-019_N1_dil-2.8358.8358.2.dta

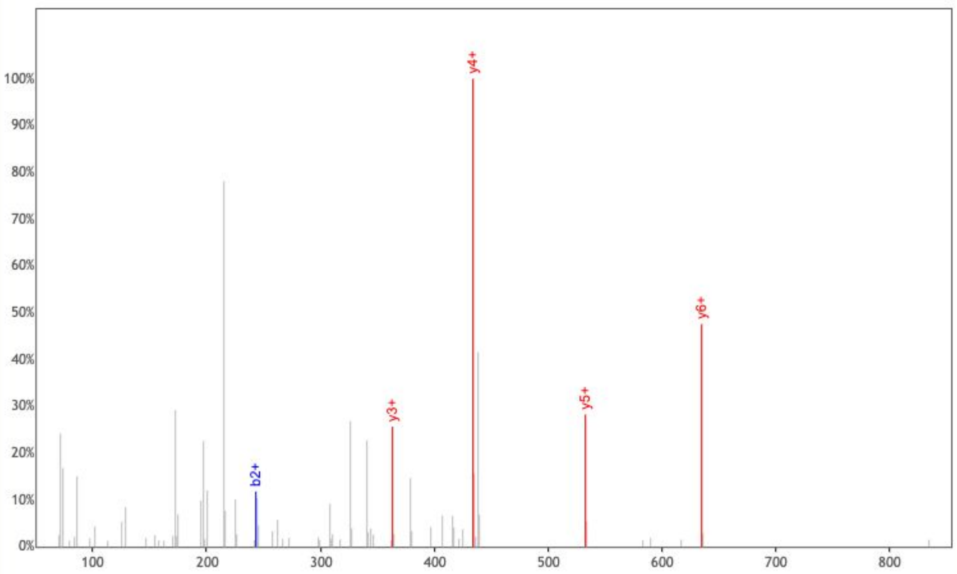

Click and drag in the plot to zoom X:☑ Y: Zoom Out Print Enable tooltip

b+	#	Seq	#	y+
130.0499	1	E	8	
243.1339	2	I	7	747.4359
344.1816	3	T	6	634.3519
443.2500	4	V	5	533.3042
514.2871	5	A	4	434.2358
615.3348	6	T	3	363.1987
702.3668	7	S	2	262.1510
	8	R	1	175.1190

[Click] to move table

MSECVLGQSK

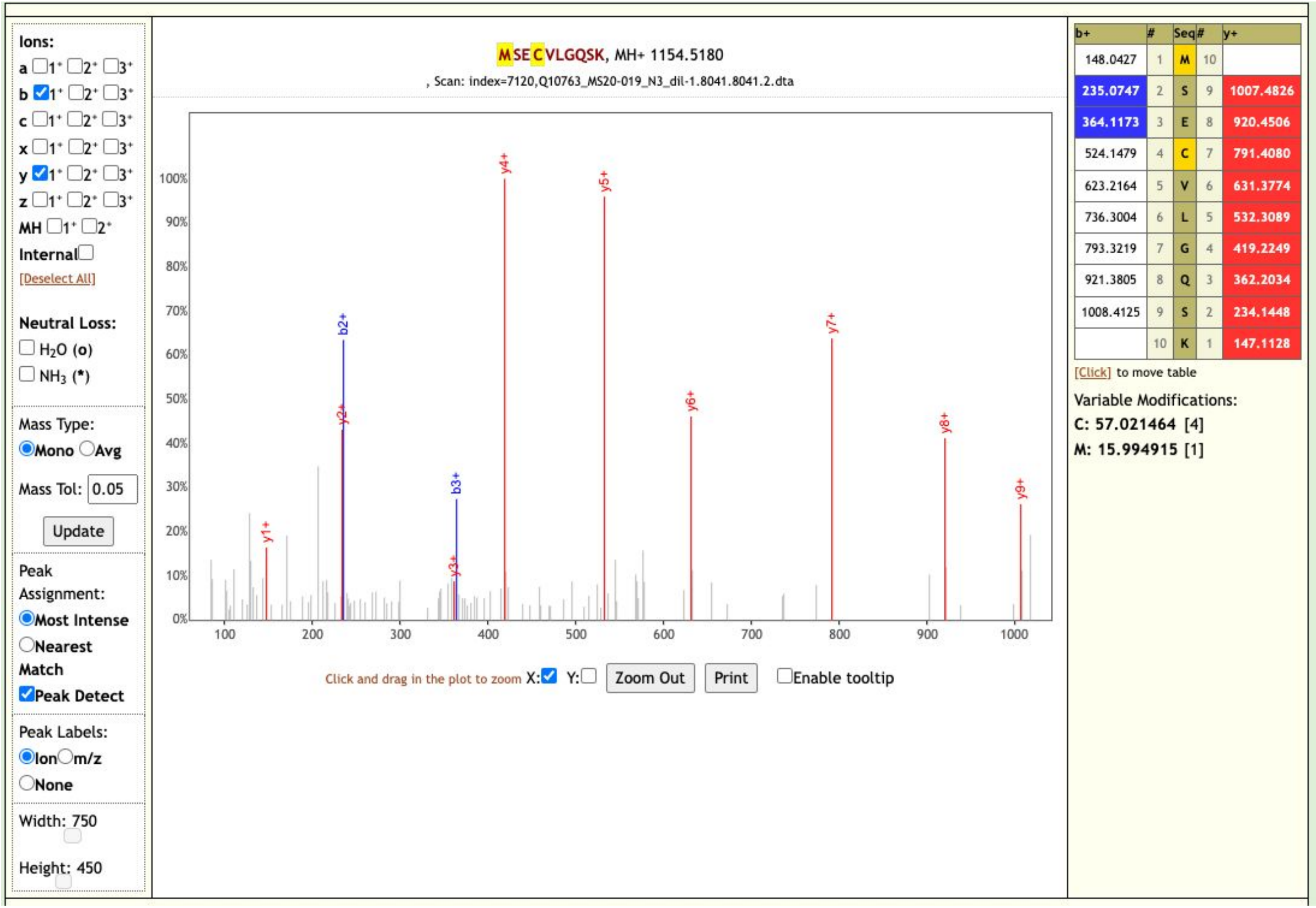
Ions:
a 1+ 2+ 3+
b 1+ 2+ 3+
c 1+ 2+ 3+
x 1+ 2+ 3+
y 1+ 2+ 3+
z 1+ 2+ 3+
MH 1+ 2+
Internal
[Deselect All]
Neutral Loss:
H2O (o)
NH3 (*)
Mass Type:
Mono Avg
Mass Tol: 0.05
Update
Peak Assignment:
Most Intense
Nearest Match
Peak Detect
Peak Labels:
Ion m/z
None
Width: 750
Height: 450
MSECVLGQSK, MH+ 1154.5180
, Scan: index=7120,Q10763_MS20-019_N3_dil-1.8041.8041.2.dta
100%
90%
80%
70%
60%
50%
40%
30%
20%
10%
0%
100
200
300
400
500
600
700
800
900
1000
y1+
b2+
y2+
b3+
y3+
y4+
y5+
y6+
y7+
y8+
y9+
Click and drag in the plot to zoom X: Y:
Zoom Out
Print
Enable tooltip
b+ # Seq # y+
148.0427 1 M 10
235.0747 2 S 9 1007.4826
364.1173 3 E 8 920.4506
524.1479 4 C 7 791.4080
623.2164 5 V 6 631.3774
736.3004 6 L 5 532.3089
793.3219 7 G 4 419.2249
921.3805 8 Q 3 362.2034
1008.4125 9 S 2 234.1448
10 K 1 147.1128
[Click] to move table
Variable Modifications:
C: 57.021464 [4]
M: 15.994915 [1]

IAGHHLGR

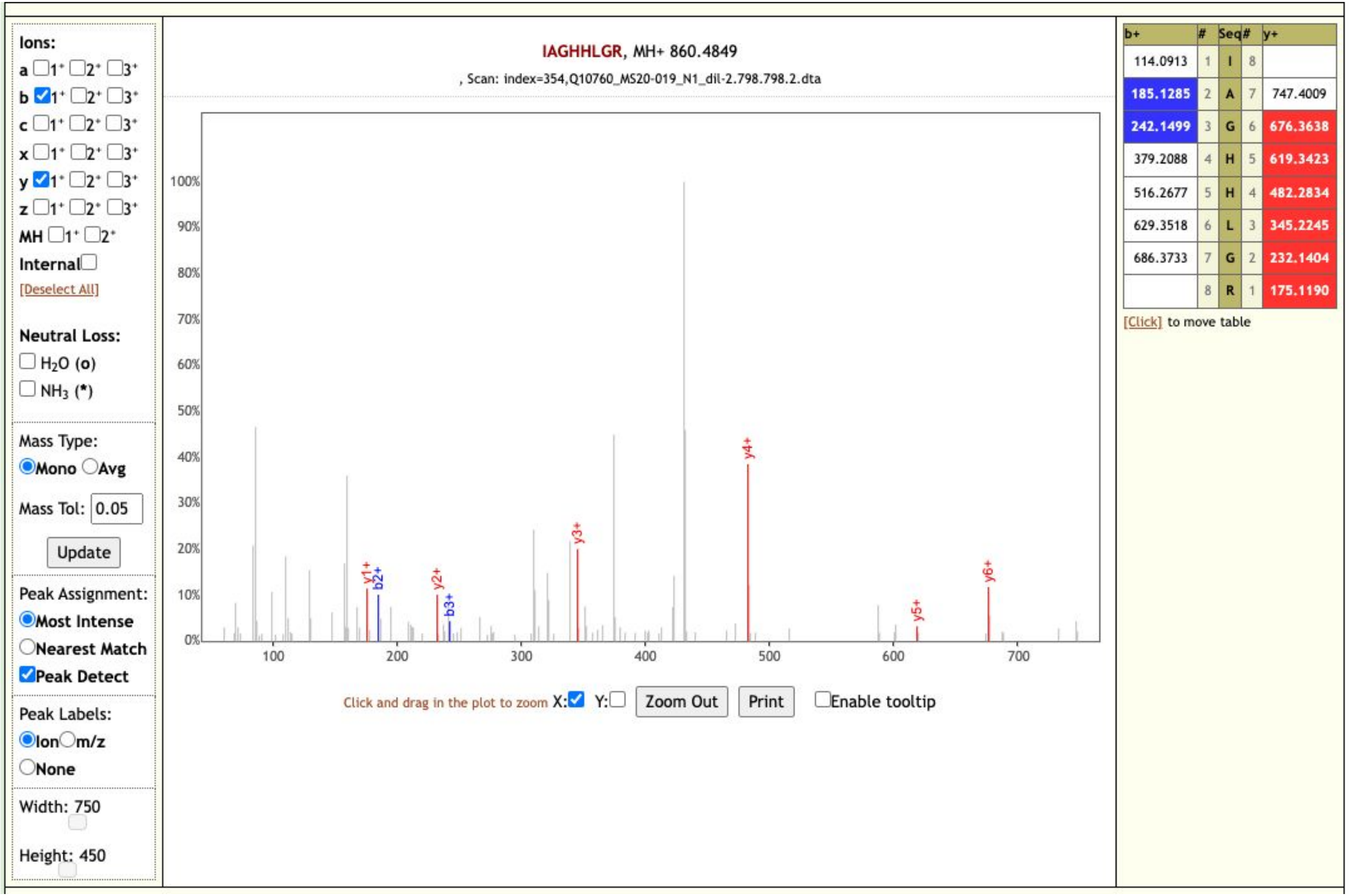

b+	#	Seq	#	y+
114.0913	1	I	8	
185.1285	2	A	7	747.4009
242.1499	3	G	6	676.3638
379.2088	4	H	5	619.3423
516.2677	5	H	4	482.2834
629.3518	6	L	3	345.2245
686.3733	7	G	2	232.1404
	8	R	1	175.1190

QLQQSMSSADSTQA

Ions:
a ☐1+ ☐2+ ☐3+
b ☑1+ ☐2+ ☐3+
c ☐1+ ☐2+ ☐3+
x ☐1+ ☐2+ ☐3+
y ☑1+ ☐2+ ☐3+
z ☐1+ ☐2+ ☐3+
MH ☐1+ ☐2+
Internal ☐
[Deselect All]

Neutral Loss:
☐ H_2O (o)
☐ NH_3 (*)

Mass Type: ◉Mono ○Avg

Mass Tol: 0.05

Update

Peak Assignment: ◉Most Intense ○Nearest Match
☑Peak Detect

Peak Labels: ◉Ion ○m/z ○None

Width: 750

Height: 450

QLQQSMSSADSTQA, MH+ 1497.6486

, Scan: index=5614,Q10761_MS20-019_N3_dil-2.6424.6424.2.dta

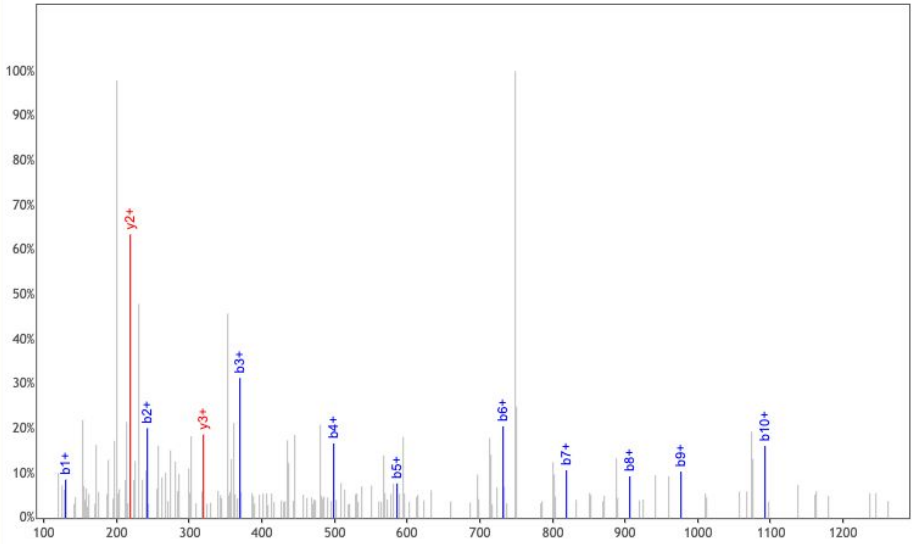

Click and drag in the plot to zoom X:☑ Y:☐ Zoom Out Print ☐Enable tooltip

b+	#	Seq	#	y+
129.0659	1	Q	14	
242.1499	2	L	13	1369.5900
370.2085	3	Q	12	1256.5059
498.2671	4	Q	11	1128.4474
585.2991	5	S	10	1000.3888
732.3345	6	M	9	913.3568
819.3665	7	S	8	766.3214
906.3986	8	S	7	679.2893
977.4357	9	A	6	592.2573
1092.4626	10	D	5	521.2202
1179.4946	11	S	4	406.1932
1280.5423	12	T	3	319.1612
1408.6009	13	Q	2	218.1135
	14	A	1	90.0550

[Click] to move table

Variable Modifications:

M: 15.994915 [6]

ASANLAATK

Ions:
a 1+ 2+ 3+
b 1+ 2+ 3+
c 1+ 2+ 3+
x 1+ 2+ 3+
y 1+ 2+ 3+
z 1+ 2+ 3+
MH 1+ 2+
Internal
[Deselect All]

Neutral Loss:
H_2O (o)
NH_3 (*)

Mass Type:
Mono Avg

Mass Tol: 0.05

Update

Peak Assignment:
Most Intense
Nearest Match
Peak Detect

Peak Labels:
Ion m/z
None

Width: 750

Height: 450

ASANLAATK, MH+ 846.4680

, Scan: index=2027,Q10763_MS20-019_N3_dil-1.2627.2627.2.dta

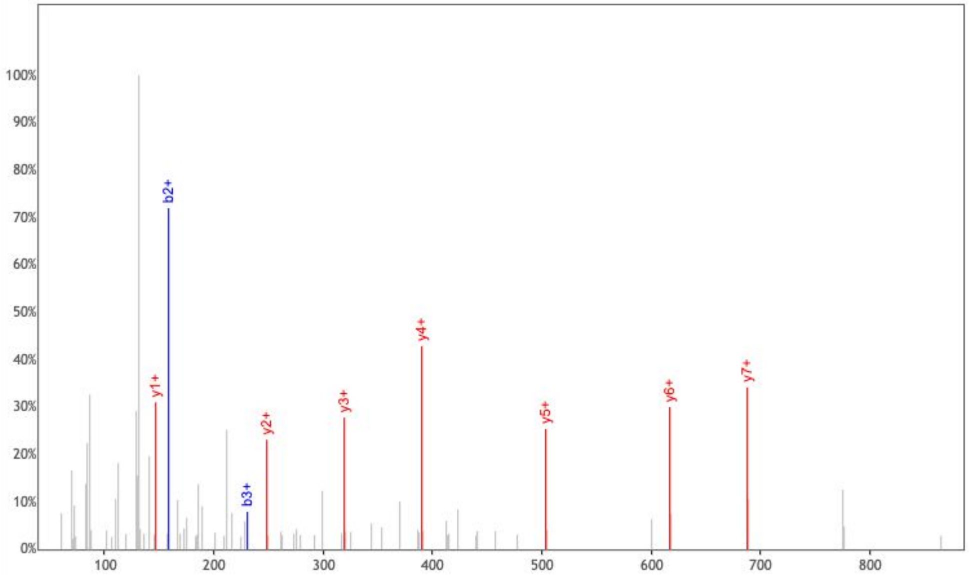

Click and drag in the plot to zoom X: Y: Zoom Out Print Enable tooltip

b+	#	Seq	#	y+
72.0444	1	A	9	
159.0764	2	S	8	775.4308
230.1135	3	A	7	688.3988
344.1565	4	N	6	617.3617
457.2405	5	L	5	503.3188
528.2776	6	A	4	390.2347
599.3148	7	A	3	319.1976
700.3624	8	T	2	248.1605
	9	K	1	147.1128

[Click] to move table

SNLKPFER

Ions:
a ☐1+ ☐2+ ☐3+
b ☑1+ ☐2+ ☐3+
c ☐1+ ☐2+ ☐3+
x ☐1+ ☐2+ ☐3+
y ☑1+ ☐2+ ☐3+
z ☐1+ ☐2+ ☐3+
MH ☐1+ ☐2+
Internal ☐
[Deselect All]

Neutral Loss:
☐ H_2O (o)
☐ NH_3 (*)

Mass Type:
◉Mono ○Avg

Mass Tol: 0.05

Update

Peak Assignment:
◉Most Intense
○Nearest Match
☑Peak Detect

Peak Labels:
◉Ion ○m/z
○None

Width: 750

Height: 450

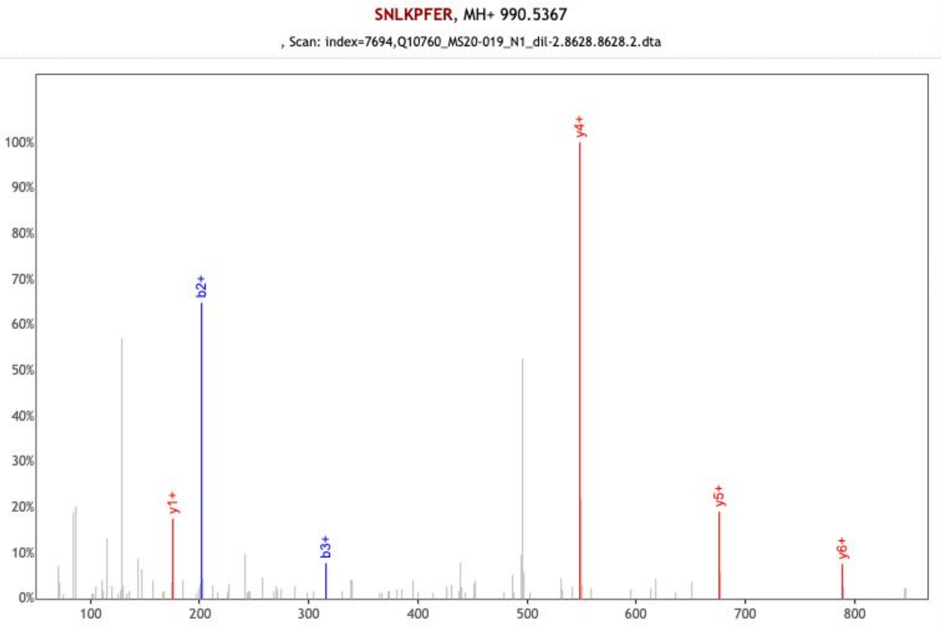

Click and drag in the plot to zoom X:☑ Y:☐ Zoom Out Print ☐Enable tooltip

b+	#	Seq	#	y+
88.0393	1	S	8	
202.0822	2	N	7	903.5047
315.1663	3	L	6	789.4617
443.2613	4	K	5	676.3777
540.3140	5	P	4	548.2827
687.3824	6	F	3	451.2300
816.4250	7	E	2	304.1615
	8	R	1	175.1190

[Click] to move table

CDIKDLPK

Ions:
a ☐1+ ☐2+ ☐3+
b ☑1+ ☐2+ ☐3+
c ☐1+ ☐2+ ☐3+
x ☐1+ ☐2+ ☐3+
y ☑1+ ☐2+ ☐3+
z ☐1+ ☐2+ ☐3+
MH ☐1+ ☐2+
Internal ☐
[Deselect All]

Neutral Loss:
☐ H_2O (o)
☐ NH_3 (*)

Mass Type:
◉Mono ○Avg

Mass Tol: 0.05

Update

Peak Assignment:
◉Most Intense
○Nearest Match
☑Peak Detect

Peak Labels:
◉Ion ○m/z
○None

Width: 750

Height: 450

CDIKDLPK, MH+ 988.5132

, Scan: index=9540,Q10763_MS20-019_N3_dil-1.10599.10599.2.dta

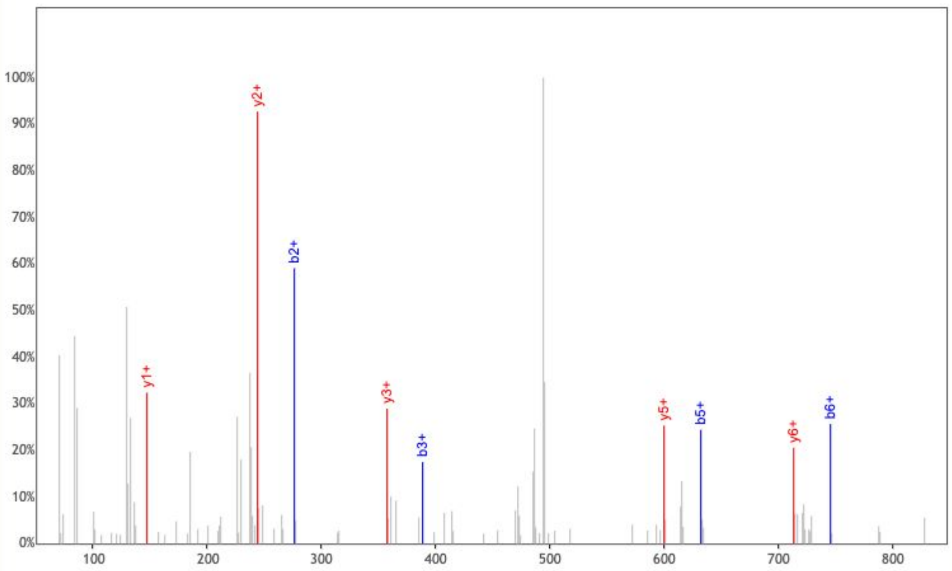

Click and drag in the plot to zoom X:☑ Y:☐ Zoom Out Print ☐Enable tooltip

b+	#	Seq	#	y+
161.0379	1	C	8	
276.0649	2	D	7	828.4825
389.1489	3	I	6	713.4556
517.2439	4	K	5	600.3715
632.2708	5	D	4	472.2766
745.3549	6	L	3	357.2496
842.4077	7	P	2	244.1656
	8	K	1	147.1128

[Click] to move table

Static Modifications:
C: 57.021464

PXD020394

TATKAYNVTQAFGR

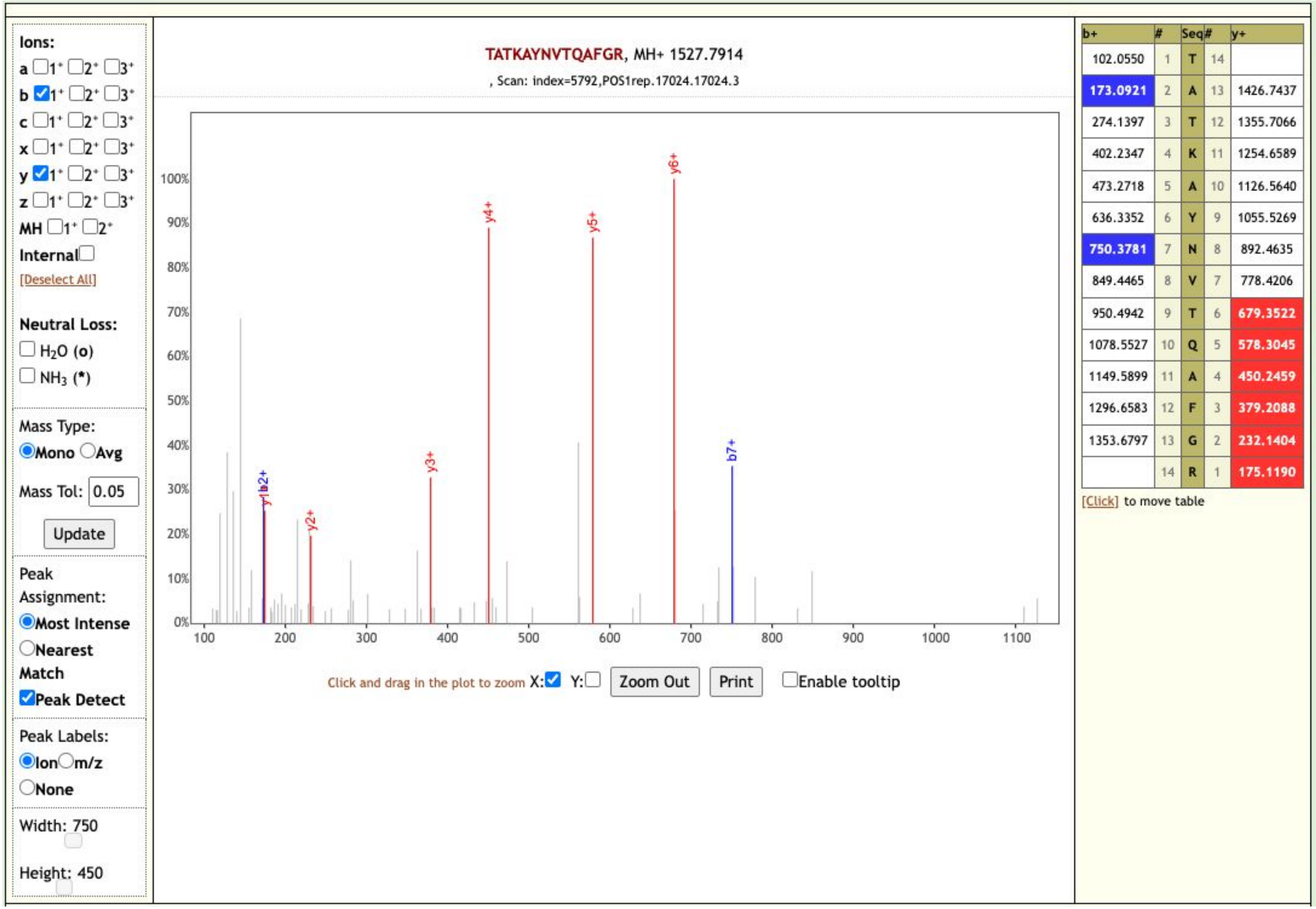

b+	#	Seq	#	y+
102.0550	1	T	14	
173.0921	2	A	13	1426.7437
274.1397	3	T	12	1355.7066
402.2347	4	K	11	1254.6589
473.2718	5	A	10	1126.5640
636.3352	6	Y	9	1055.5269
750.3781	7	N	8	892.4635
849.4465	8	V	7	778.4206
950.4942	9	T	6	679.3522
1078.5527	10	Q	5	578.3045
1149.5899	11	A	4	450.2459
1296.6583	12	F	3	379.2088
1353.6797	13	G	2	232.1404
	14	R	1	175.1190

[Click] to move table

DGIIWVATEGALNTPK

Ions:
a ☐1+ ☐2+ ☐3+
b ☑1+ ☐2+ ☐3+
c ☐1+ ☐2+ ☐3+
x ☐1+ ☐2+ ☐3+
y ☑1+ ☐2+ ☐3+
z ☐1+ ☐2+ ☐3+
MH ☐1+ ☐2+
Internal ☐
[Deselect All]

Neutral Loss:
☐ H_2O (o)
☐ NH_3 (*)

Mass Type:
◉ Mono ○ Avg

Mass Tol: 0.05

Update

Peak Assignment:
◉ Most Intense
○ Nearest Match
☑ Peak Detect

Peak Labels:
◉ Ion ○ m/z
○ None

Width: 750

Height: 450

DGIIWVATEGALNTPK, MH+ 1684.8905

, Scan: index=15922,POS1.34763.34763.2

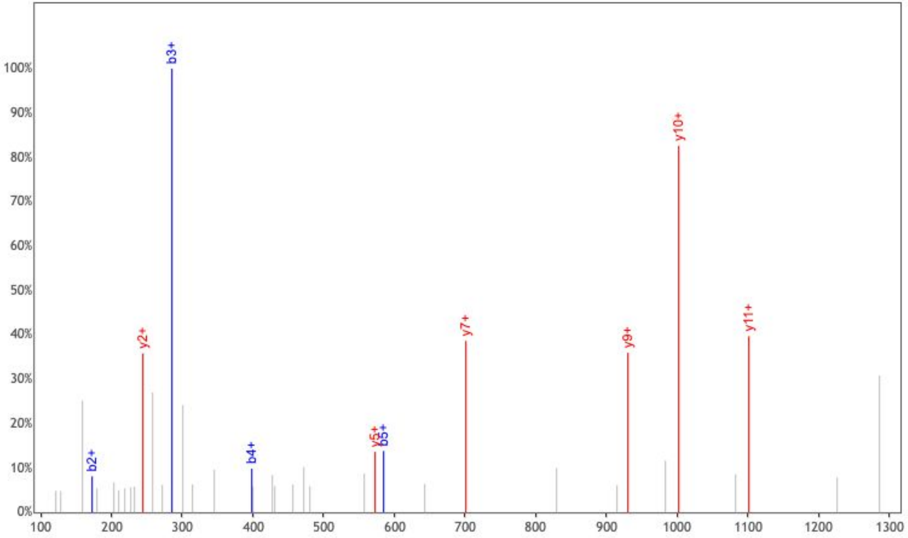

Click and drag in the plot to zoom X: ☑ Y: ☐ Zoom Out Print ☐ Enable tooltip

b+	#	Seq	#	y+
116.0342	1	D	16	
173.0557	2	G	15	1569.8635
286.1397	3	I	14	1512.8421
399.2238	4	I	13	1399.7580
585.3031	5	W	12	1286.6739
684.3715	6	V	11	1100.5946
755.4087	7	A	10	1001.5262
856.4563	8	T	9	930.4891
985.4989	9	E	8	829.4414
1042.5204	10	G	7	700.3988
1113.5575	11	A	6	643.3774
1226.6416	12	L	5	572.3402
1340.6845	13	N	4	459.2562
1441.7322	14	T	3	345.2132
1538.7849	15	P	2	244.1656
	16	K	1	147.1128

[Click] to move table

MAGNGGDAALALLLLDR

Ions:
a 1+ 2+ 3+
b 1+ 2+ 3+
c 1+ 2+ 3+
x 1+ 2+ 3+
y 1+ 2+ 3+
z 1+ 2+ 3+
MH 1+ 2+
Internal
[Deselect All]

Neutral Loss:
H_2O (o)
NH_3 (*)

Mass Type:
Mono Avg

Mass Tol: 0.05

Update

Peak Assignment:
Most Intense
Nearest Match
Peak Detect

Peak Labels:
Ion m/z
None

Width: 750

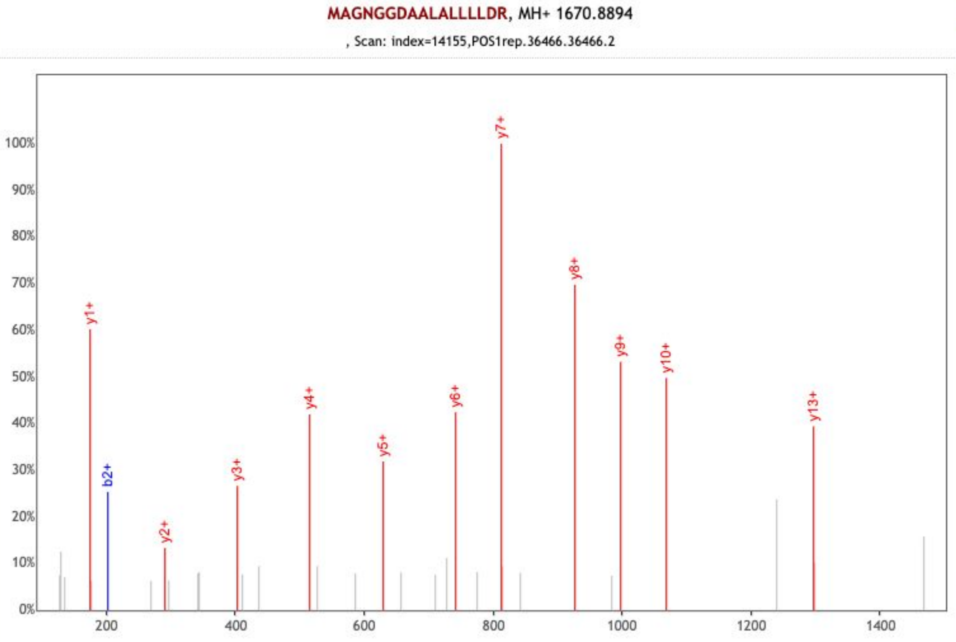

Click and drag in the plot to zoom X: Y: Zoom Out Print Enable tooltip

b+	#	Seq	#	y+
132.0478	1	M	17	
203.0849	2	A	16	1539.8489
260.1063	3	G	15	1468.8118
374.1493	4	N	14	1411.7904
431.1707	5	G	13	1297.7474
488.1922	6	G	12	1240.7260
603.2191	7	D	11	1183.7045
674.2562	8	A	10	1068.6776
745.2934	9	A	9	997.6404
858.3774	10	L	8	926.6033
929.4145	11	A	7	813.5193
1042.4986	12	L	6	742.4822
1155.5827	13	L	5	629.3981
1268.6667	14	L	4	516.3140
1381.7508	15	L	3	403.2300
1496.7777	16	D	2	290.1459
	17	R	1	175.1190

[Click] to move table

QGTDYKHWPQIAQFAPSASAFFGMSR

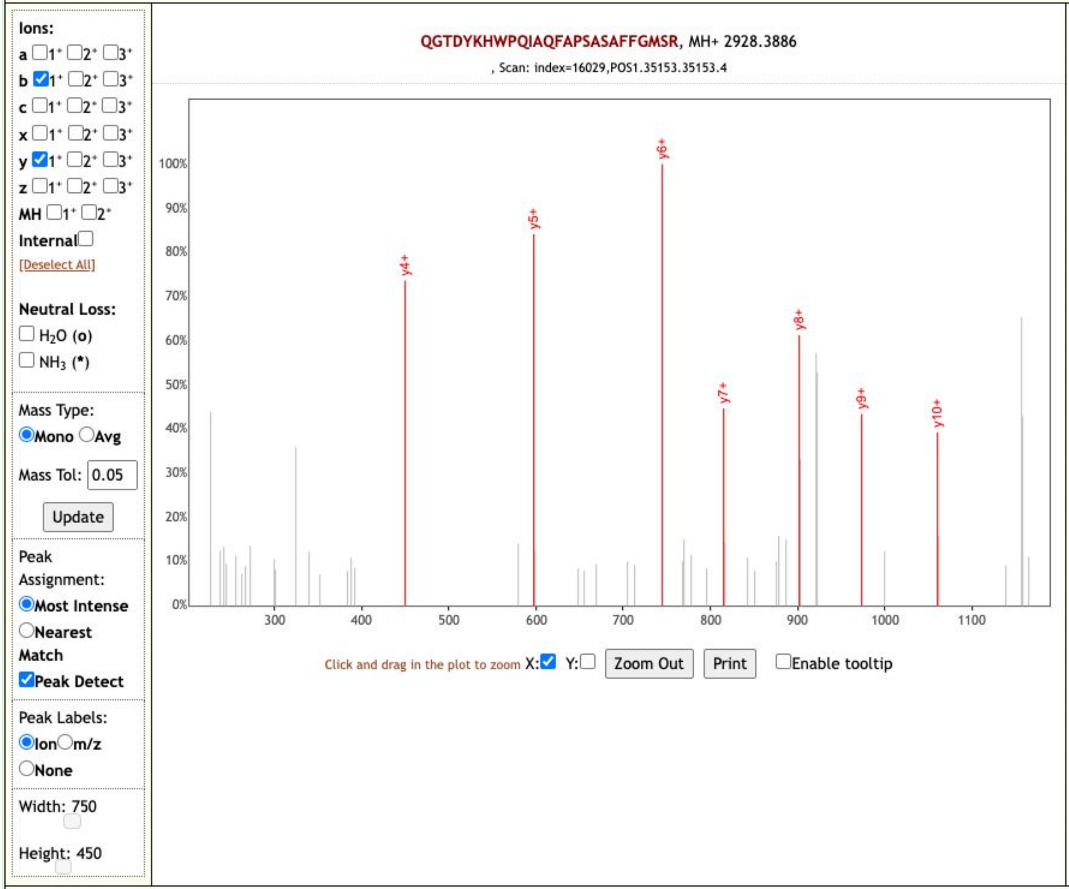

b+	#	Seq	#	y+
129.0659	1	Q	26	
186.0873	2	G	25	2800.3300
287.1350	3	T	24	2743.3086
402.1619	4	D	23	2642.2609
565.2253	5	Y	22	2527.2339
693.3202	6	K	21	2364.1706
830.3791	7	H	20	2236.0757
1016.4585	8	W	19	2099.0167
1113.5112	9	P	18	1912.9374
1241.5698	10	Q	17	1815.8847
1354.6539	11	I	16	1687.8261
1425.6910	12	A	15	1574.7420
1553.7496	13	Q	14	1503.7049
1700.8180	14	F	13	1375.6463
1771.8551	15	A	12	1228.5779
1868.9078	16	P	11	1157.5408
1955.9399	17	S	10	1060.4880
2026.9770	18	A	9	973.4560
2114.0090	19	S	8	902.4189
2185.0461	20	A	7	815.3869
2332.1145	21	F	6	744.3498
2479.1830	22	F	5	597.2813
2536.2044	23	G	4	450.2129
2667.2449	24	M	3	393.1915
2754.2769	25	S	2	262.1510
	26	R	1	175.1190

[Click] to move table

MAGDGGDAALALLLLDR

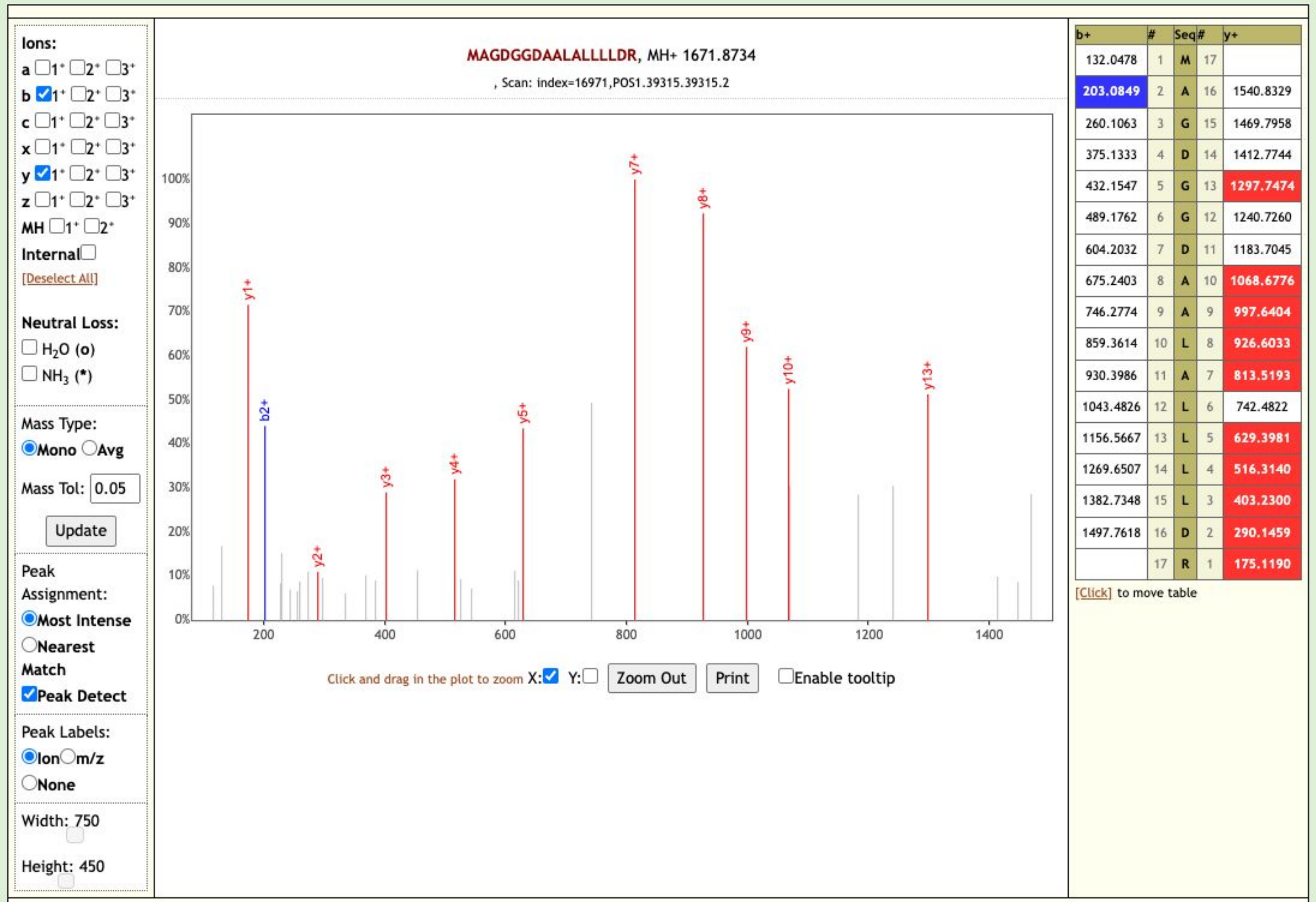

b+	#	Seq	#	y+
132.0478	1	M	17	
203.0849	2	A	16	1540.8329
260.1063	3	G	15	1469.7958
375.1333	4	D	14	1412.7744
432.1547	5	G	13	1297.7474
489.1762	6	G	12	1240.7260
604.2032	7	D	11	1183.7045
675.2403	8	A	10	1068.6776
746.2774	9	A	9	997.6404
859.3614	10	L	8	926.6033
930.3986	11	A	7	813.5193
1043.4826	12	L	6	742.4822
1156.5667	13	L	5	629.3981
1269.6507	14	L	4	516.3140
1382.7348	15	L	3	403.2300
1497.7618	16	D	2	290.1459
	17	R	1	175.1190

MAGNGGDAALALLLLDR

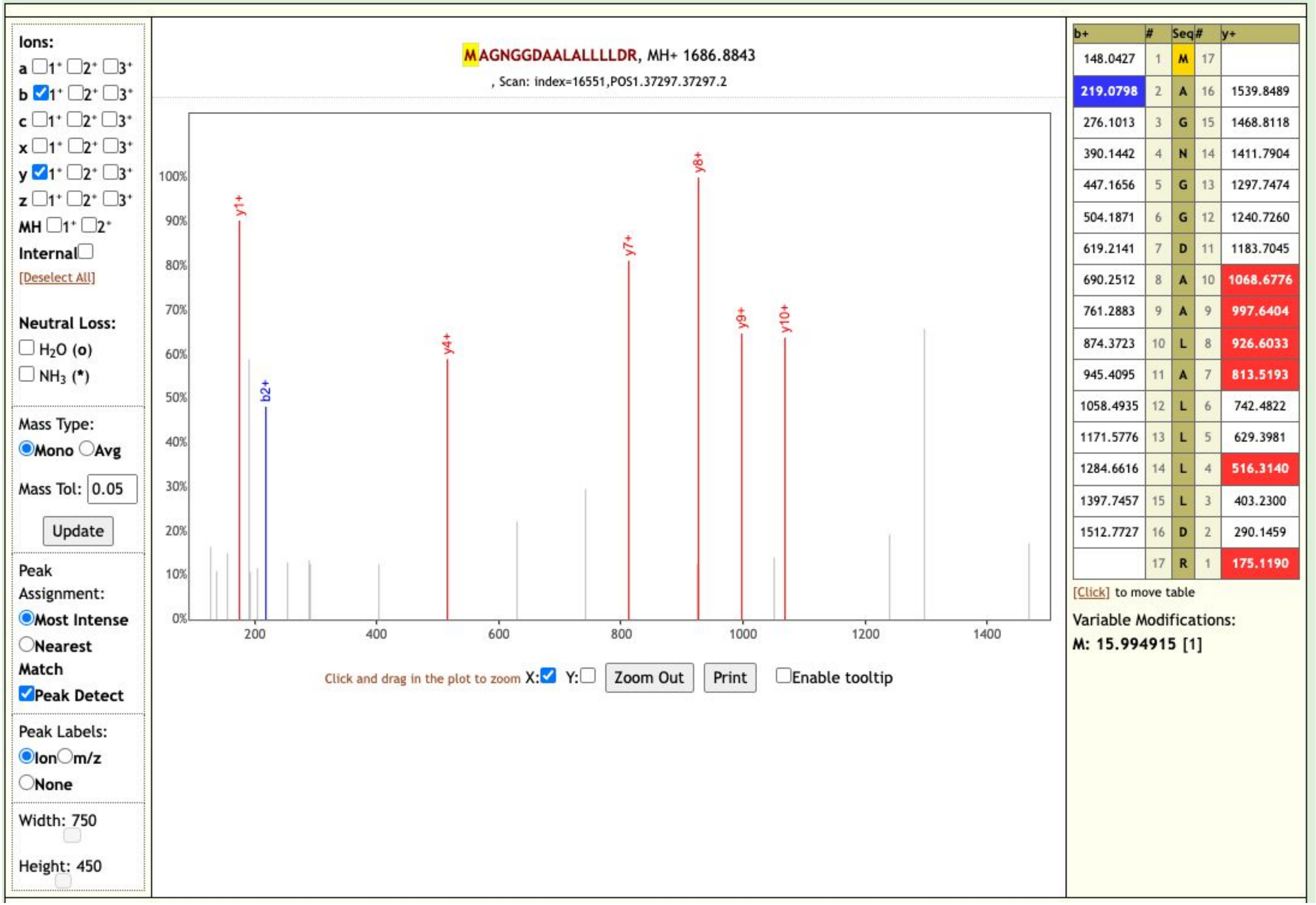

QKRTATKAYNVTQAFGR

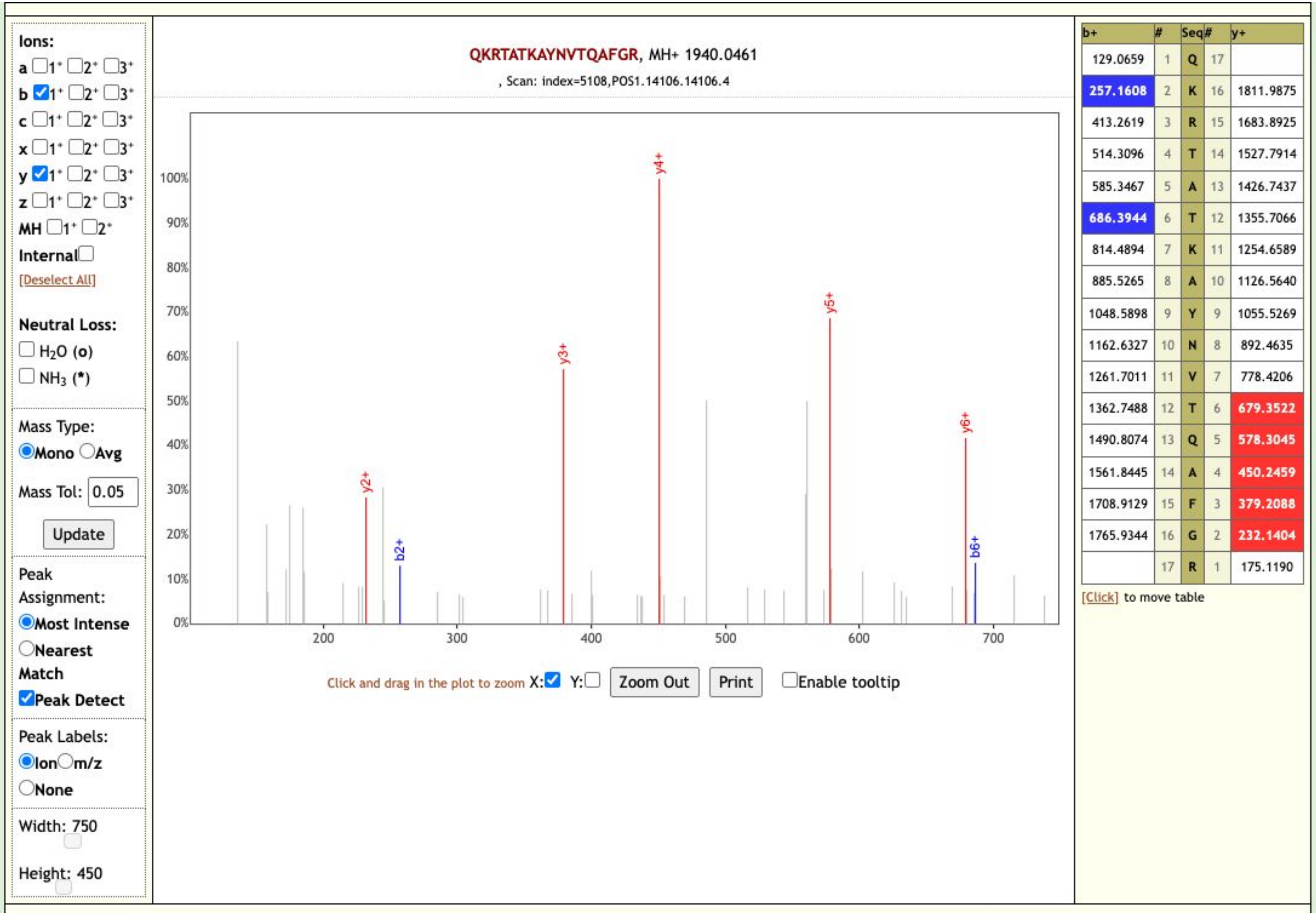

b+	#	Seq	#	y+
129.0659	1	Q	17	
257.1608	2	K	16	1811.9875
413.2619	3	R	15	1683.8925
514.3096	4	T	14	1527.7914
585.3467	5	A	13	1426.7437
686.3944	6	T	12	1355.7066
814.4894	7	K	11	1254.6589
885.5265	8	A	10	1126.5640
1048.5898	9	Y	9	1055.5269
1162.6327	10	N	8	892.4635
1261.7011	11	V	7	778.4206
1362.7488	12	T	6	679.3522
1490.8074	13	Q	5	578.3045
1561.8445	14	A	4	450.2459
1708.9129	15	F	3	379.2088
1765.9344	16	G	2	232.1404
	17	R	1	175.1190

PXD021328

DGIIWVATEGALNTPK

Ions:
a ☐1⁺ ☐2⁺ ☐3⁺
b ☑1⁺ ☐2⁺ ☐3⁺
c ☐1⁺ ☐2⁺ ☐3⁺
x ☐1⁺ ☐2⁺ ☐3⁺
y ☑1⁺ ☐2⁺ ☐3⁺
z ☐1⁺ ☐2⁺ ☐3⁺
MH ☐1⁺ ☐2⁺
Internal ☐
[Deselect All]

Neutral Loss:
☐ H_2O (o)
☐ NH_3 (*)

Mass Type:
◉ Mono ○ Avg

Mass Tol: 0.05

Update

Peak Assignment:
◉ Most Intense
○ Nearest Match
☑ Peak Detect

Peak Labels:
◉ Ion ○ m/z
○ None

Width: 750

DGIIWVATEGALNTPK, MH+ 1684.8905

, Scan: index=33994,PXD019119_pool-51.36978.36978.2

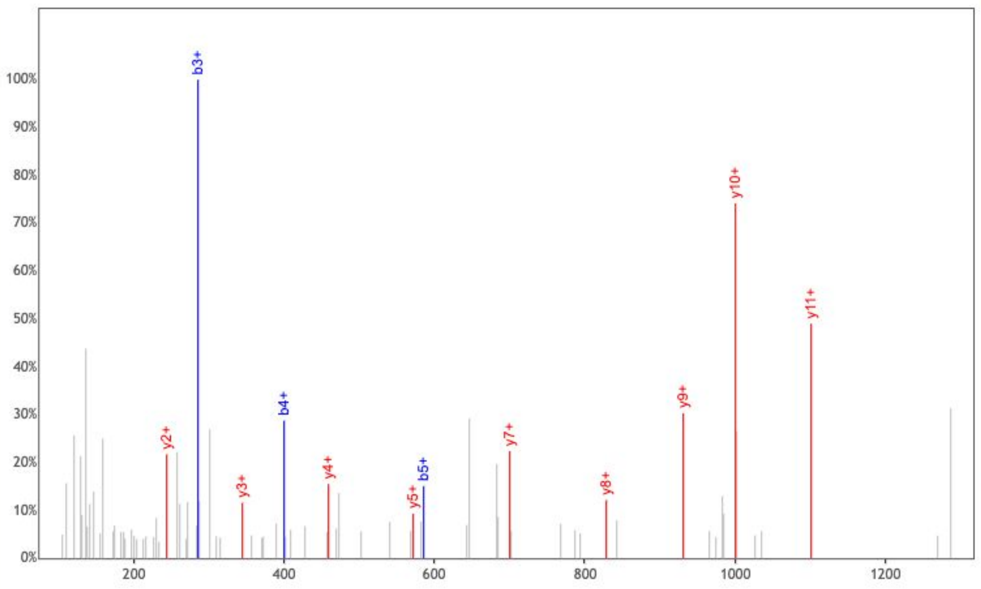

Click and drag in the plot to zoom X: ☑ Y: ☐ Zoom Out Print ☐ Enable tooltip

b+	#	Seq	#	y+
116.0342	1	D	16	
173.0557	2	G	15	1569.8635
286.1397	3	I	14	1512.8421
399.2238	4	I	13	1399.7580
585.3031	5	W	12	1286.6739
684.3715	6	V	11	1100.5946
755.4087	7	A	10	1001.5262
856.4563	8	T	9	930.4891
985.4989	9	E	8	829.4414
1042.5204	10	G	7	700.3988
1113.5575	11	A	6	643.3774
1226.6416	12	L	5	572.3402
1340.6845	13	N	4	459.2562
1441.7322	14	T	3	345.2132
1538.7849	15	P	2	244.1656
	16	K	1	147.1128

[Click] to move table

LDDKDPNFKDQVILLNK

Ions:
a ☐1⁺ ☐2⁺ ☐3⁺
b ☑1⁺ ☐2⁺ ☐3⁺
c ☐1⁺ ☐2⁺ ☐3⁺
x ☐1⁺ ☐2⁺ ☐3⁺
y ☑1⁺ ☐2⁺ ☐3⁺
z ☐1⁺ ☐2⁺ ☐3⁺
MH ☐1⁺ ☐2⁺
Internal ☐
[Deselect All]

Neutral Loss:
☐ H_2O (o)
☐ NH_3 (*)

Mass Type:
◉Mono ○Avg

Mass Tol: 0.05

Update

Peak Assignment:
◉Most Intense
○Nearest Match
☑Peak Detect

Peak Labels:
◉Ion ○m/z
○None

Width: 750

Height: 450

LDDKDPNFKDQVILLNK, MH+ 2015.0808

, Scan: index=19829,PXD019119_pool-34.21874.21874.4

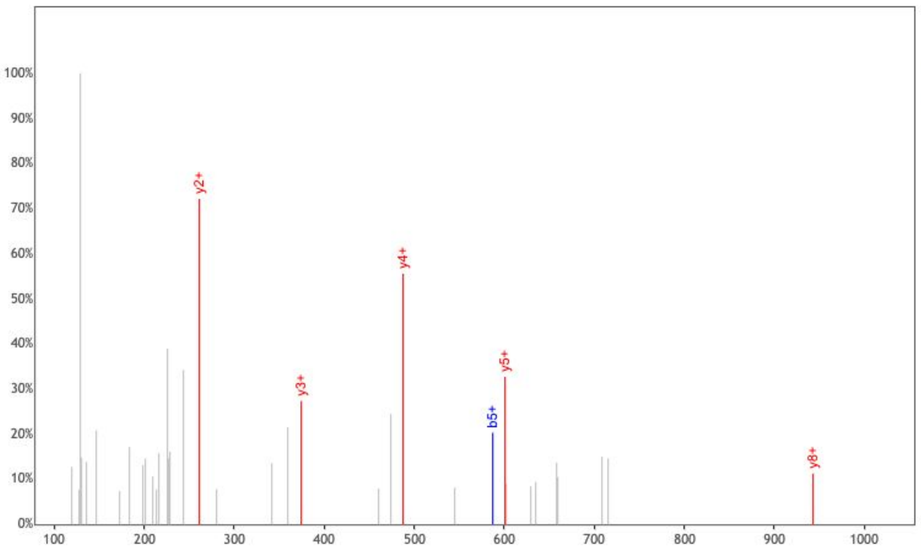

Click and drag in the plot to zoom X:☑ Y:☐ Zoom Out Print ☐Enable tooltip

b+	#	Seq	#	y+
114.0913	1	L	17	
229.1183	2	D	16	1901.9967
344.1452	3	D	15	1786.9698
472.2402	4	K	14	1671.9428
587.2671	5	D	13	1543.8479
684.3199	6	P	12	1428.8209
798.3628	7	N	11	1331.7682
945.4312	8	F	10	1217.7252
1073.5262	9	K	9	1070.6568
1188.5531	10	D	8	942.5619
1316.6117	11	Q	7	827.5349
1415.6801	12	V	6	699.4763
1528.7642	13	I	5	600.4079
1641.8483	14	L	4	487.3239
1754.9323	15	L	3	374.2398
1868.9753	16	N	2	261.1557
	17	K	1	147.1128

[Click] to move table

AYNVTQAFGR

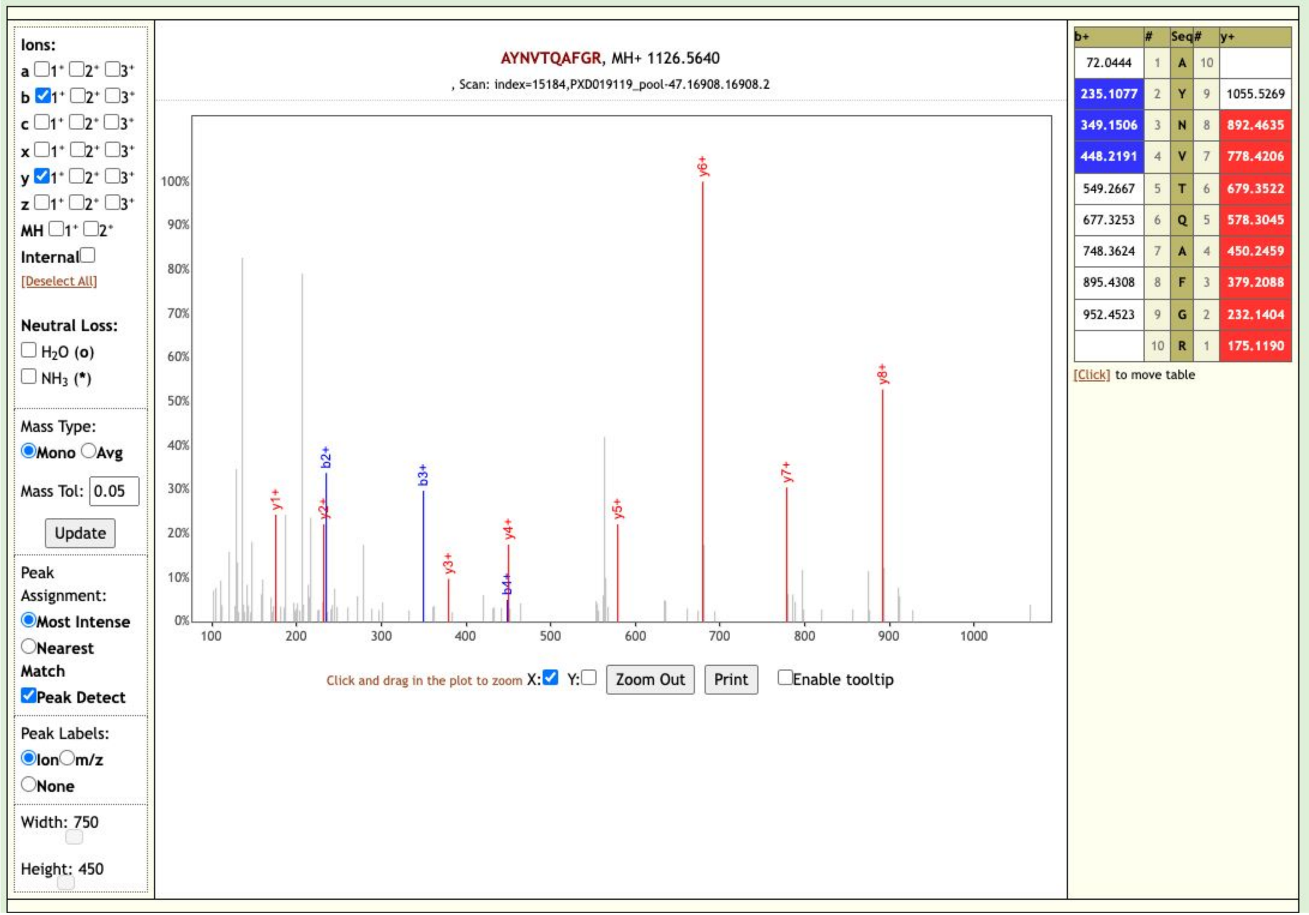

AYNVTQAFGR, MH+ 1126.5640
, Scan: index=15184,PXD019119_pool-47.16908.16908.2
Ions:
Neutral Loss:
Mass Type:
Mono Avg
Mass Tol: 0.05
Update
Peak Assignment:
Most Intense
Nearest Match
Peak Detect
Peak Labels:
Ion m/z
None
Width: 750
Height: 450
[Deselect All]
y1+ y2+ b2+ b3+ y3+ b4+ y4+ y5+ y6+ y7+ y8+
Click and drag in the plot to zoom X: Y:
Zoom Out
Print
Enable tooltip
b+ # Seq # y+
72.0444 1 A 10
235.1077 2 Y 9 1055.5269
349.1506 3 N 8 892.4635
448.2191 4 V 7 778.4206
549.2667 5 T 6 679.3522
677.3253 6 Q 5 578.3045
748.3624 7 A 4 450.2459
895.4308 8 F 3 379.2088
952.4523 9 G 2 232.1404
10 R 1 175.1190
[Click] to move table

GQGVPINTNSSPDDQIGYYR

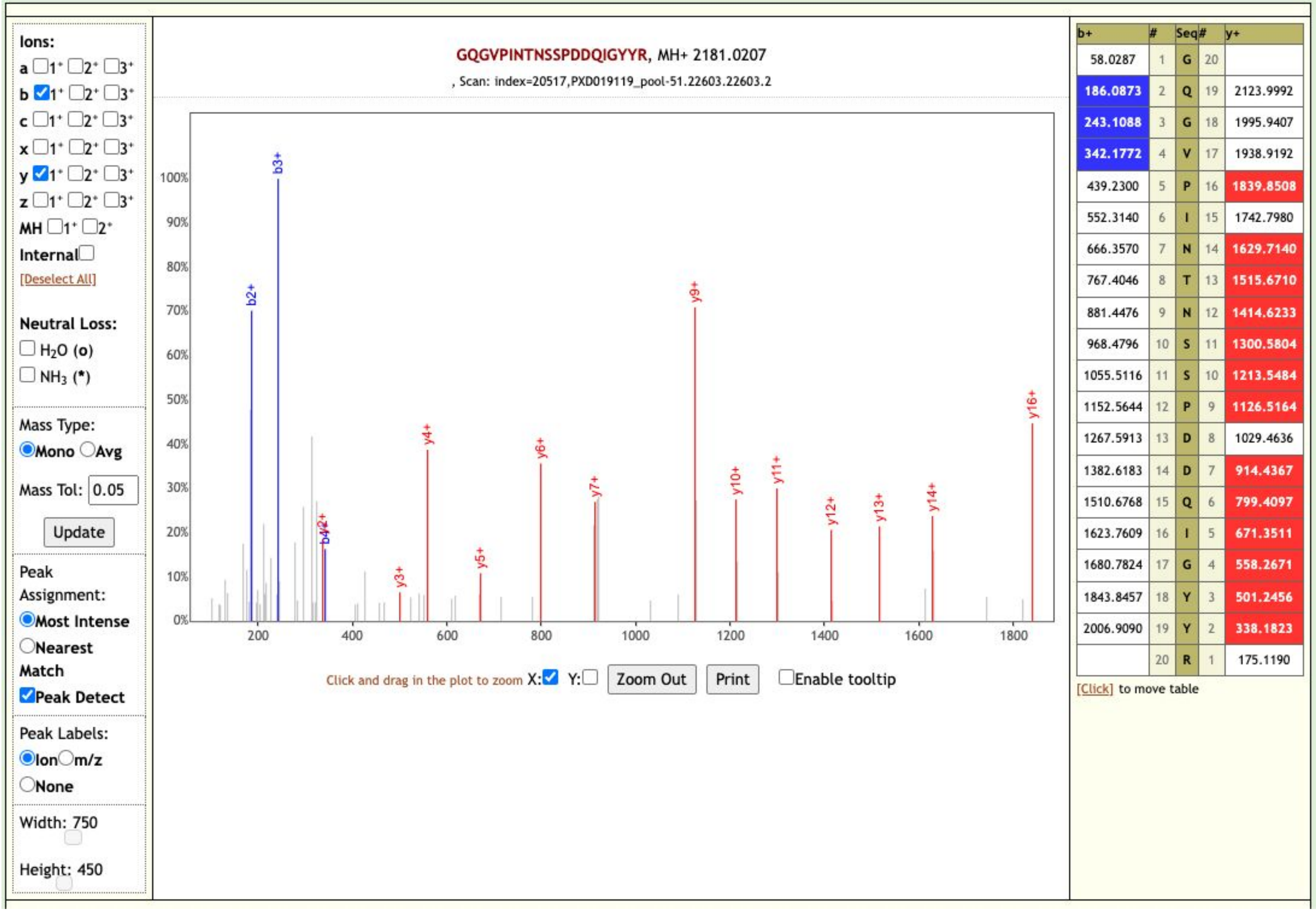

b+	#	Seq	#	y+
58.0287	1	G	20	
186.0873	2	Q	19	2123.9992
243.1088	3	G	18	1995.9407
342.1772	4	V	17	1938.9192
439.2300	5	P	16	1839.8508
552.3140	6	I	15	1742.7980
666.3570	7	N	14	1629.7140
767.4046	8	T	13	1515.6710
881.4476	9	N	12	1414.6233
968.4796	10	S	11	1300.5804
1055.5116	11	S	10	1213.5484
1152.5644	12	P	9	1126.5164
1267.5913	13	D	8	1029.4636
1382.6183	14	D	7	914.4367
1510.6768	15	Q	6	799.4097
1623.7609	16	I	5	671.3511
1680.7824	17	G	4	558.2671
1843.8457	18	Y	3	501.2456
2006.9090	19	Y	2	338.1823
	20	R	1	175.1190

[Click] to move table

HWPQIAQFAPSASAFF

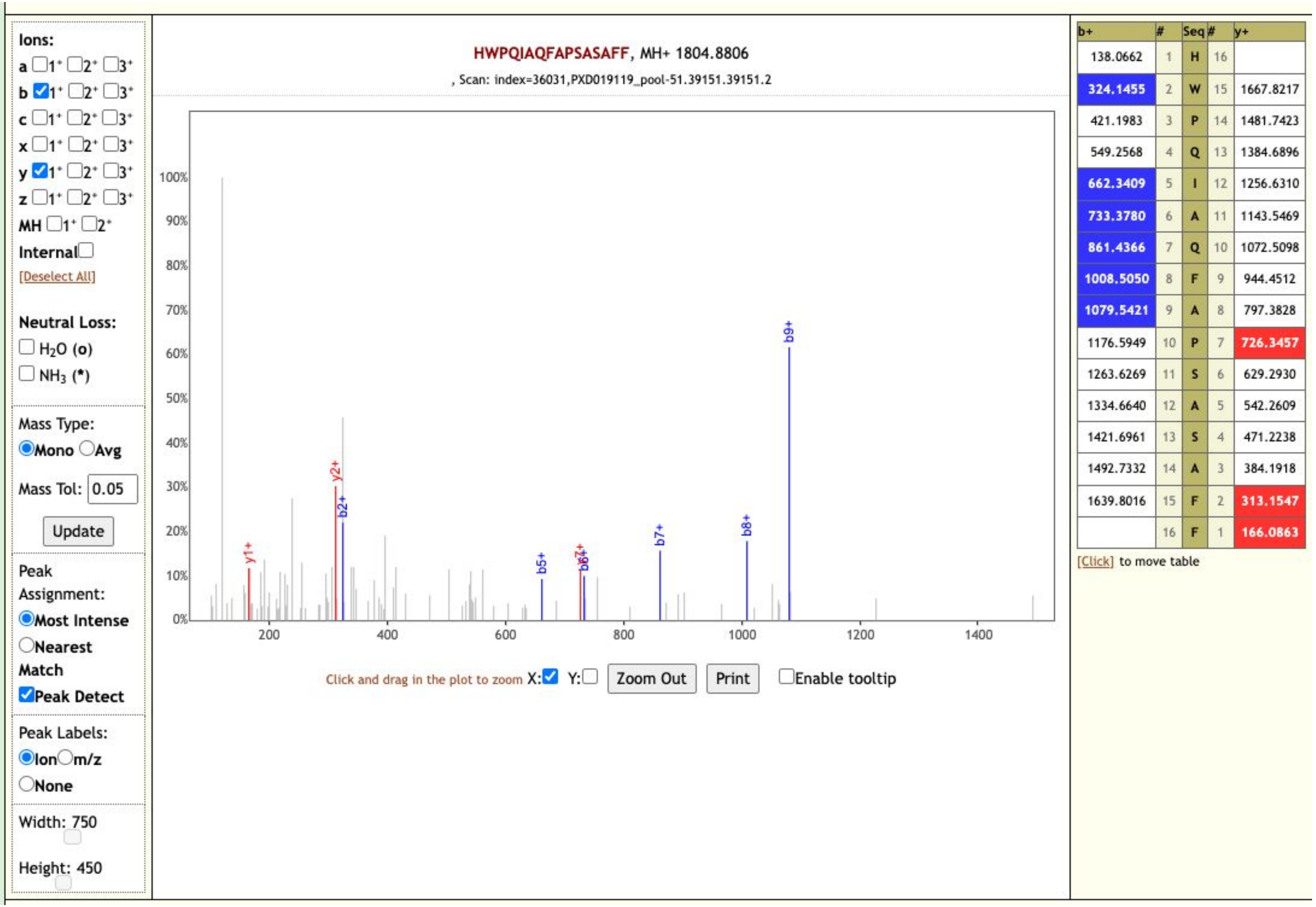

b+	#	Seq	#	y+
138.0662	1	H	16	
324.1455	2	W	15	1667.8217
421.1983	3	P	14	1481.7423
549.2568	4	Q	13	1384.6896
662.3409	5	I	12	1256.6310
733.3780	6	A	11	1143.5469
861.4366	7	Q	10	1072.5098
1008.5050	8	F	9	944.4512
1079.5421	9	A	8	797.3828
1176.5949	10	P	7	726.3457
1263.6269	11	S	6	629.2930
1334.6640	12	A	5	542.2609
1421.6961	13	S	4	471.2238
1492.7332	14	A	3	384.1918
1639.8016	15	F	2	313.1547
	16	F	1	166.0863

KQQTVTLLPAADLDDFSK

Ions:
a 1+ 2+ 3+
b ☑1+ 2+ 3+
c 1+ 2+ 3+
x 1+ 2+ 3+
y ☑1+ 2+ 3+
z 1+ 2+ 3+
MH 1+ 2+
Internal
[Deselect All]

Neutral Loss:
H_2O (o)
NH_3 (*)

Mass Type:
Mono Avg

Mass Tol: 0.05

Update

Peak Assignment:
Most Intense
Nearest Match
☑Peak Detect

Peak Labels:
Ion m/z
None

Width: 750

Height: 450

KQQTVTLLPAADLDDFSK, MH+ 1990.0491

, Scan: index=27474,PXD019119_pool-47.30017.30017.2

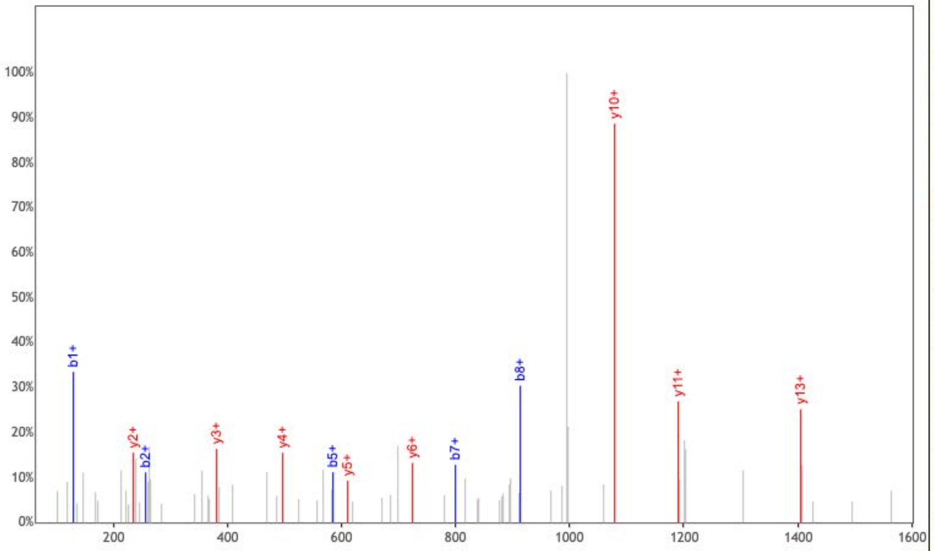

Click and drag in the plot to zoom X: Y: Zoom Out Print Enable tooltip

b+	#	Seq	#	y+
129.1022	1	K	18	
257.1608	2	Q	17	1861.9542
385.2194	3	Q	16	1733.8956
486.2671	4	T	15	1605.8370
585.3355	5	V	14	1504.7894
686.3832	6	T	13	1405.7209
799.4672	7	L	12	1304.6733
912.5513	8	L	11	1191.5892
1009.6041	9	P	10	1078.5051
1080.6412	10	A	9	981.4524
1151.6783	11	A	8	910.4153
1266.7052	12	D	7	839.3781
1379.7893	13	L	6	724.3512
1494.8162	14	D	5	611.2671
1609.8432	15	D	4	496.2402
1756.9116	16	F	3	381.2132
1843.9436	17	S	2	234.1448
	18	K	1	147.1128

[Click] to move table

KADETQALPQR

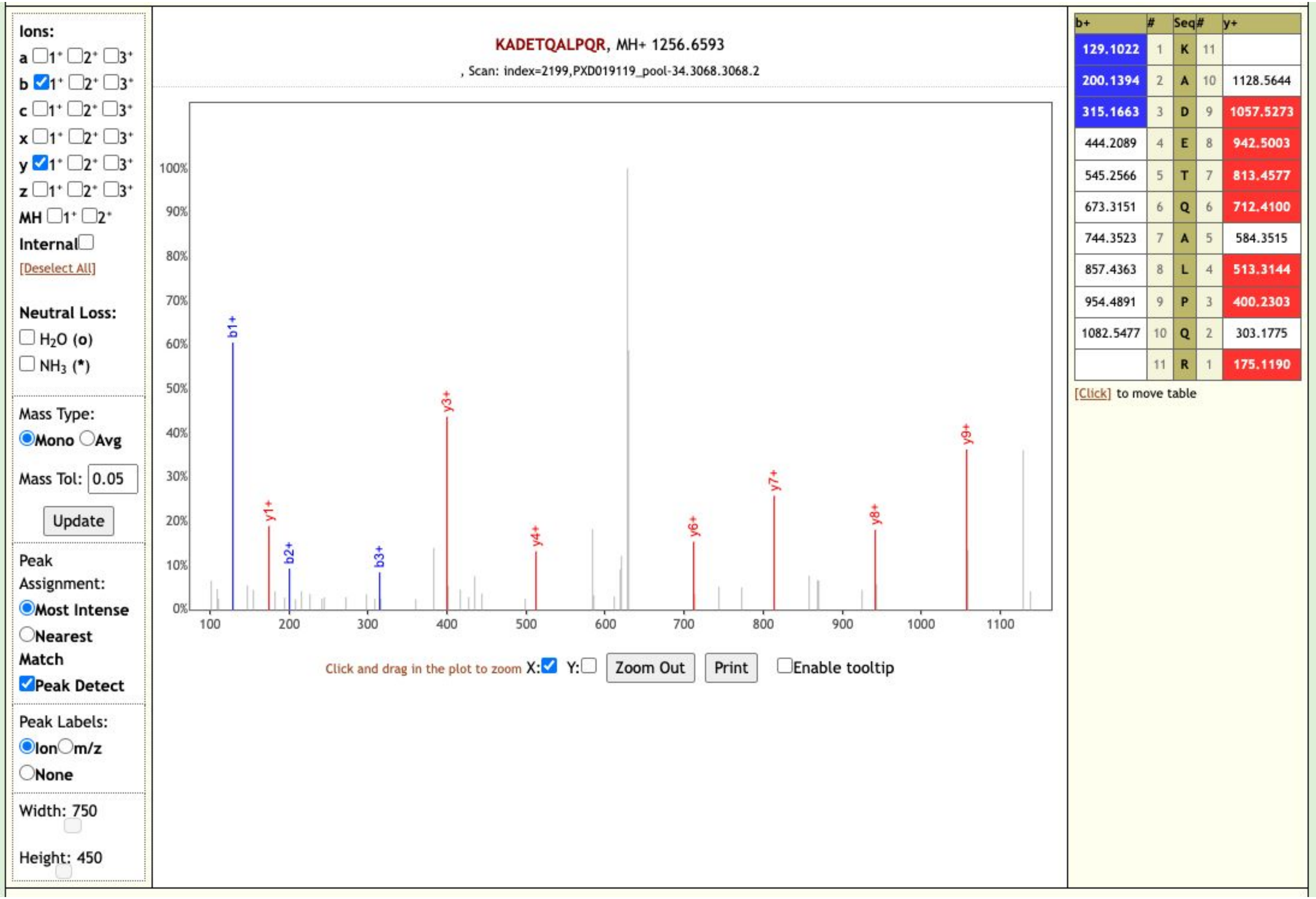

ADETQALPQR

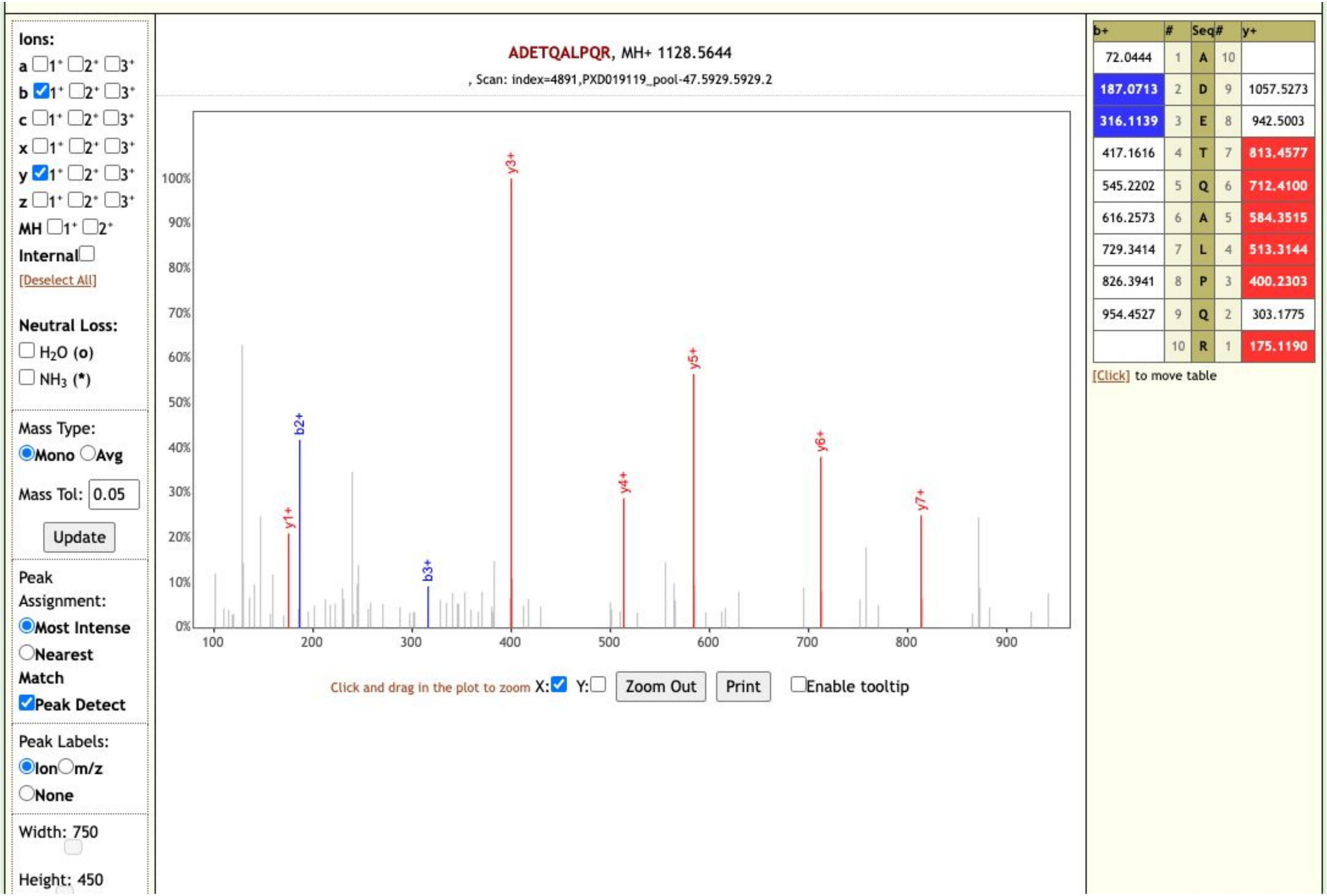

b+	#	Seq	#	y+
72.0444	1	A	10	
187.0713	2	D	9	1057.5273
316.1139	3	E	8	942.5003
417.1616	4	T	7	813.4577
545.2202	5	Q	6	712.4100
616.2573	6	A	5	584.3515
729.3414	7	L	4	513.3144
826.3941	8	P	3	400.2303
954.4527	9	Q	2	303.1775
	10	R	1	175.1190

ITFGGPSDSTGSNQNGER

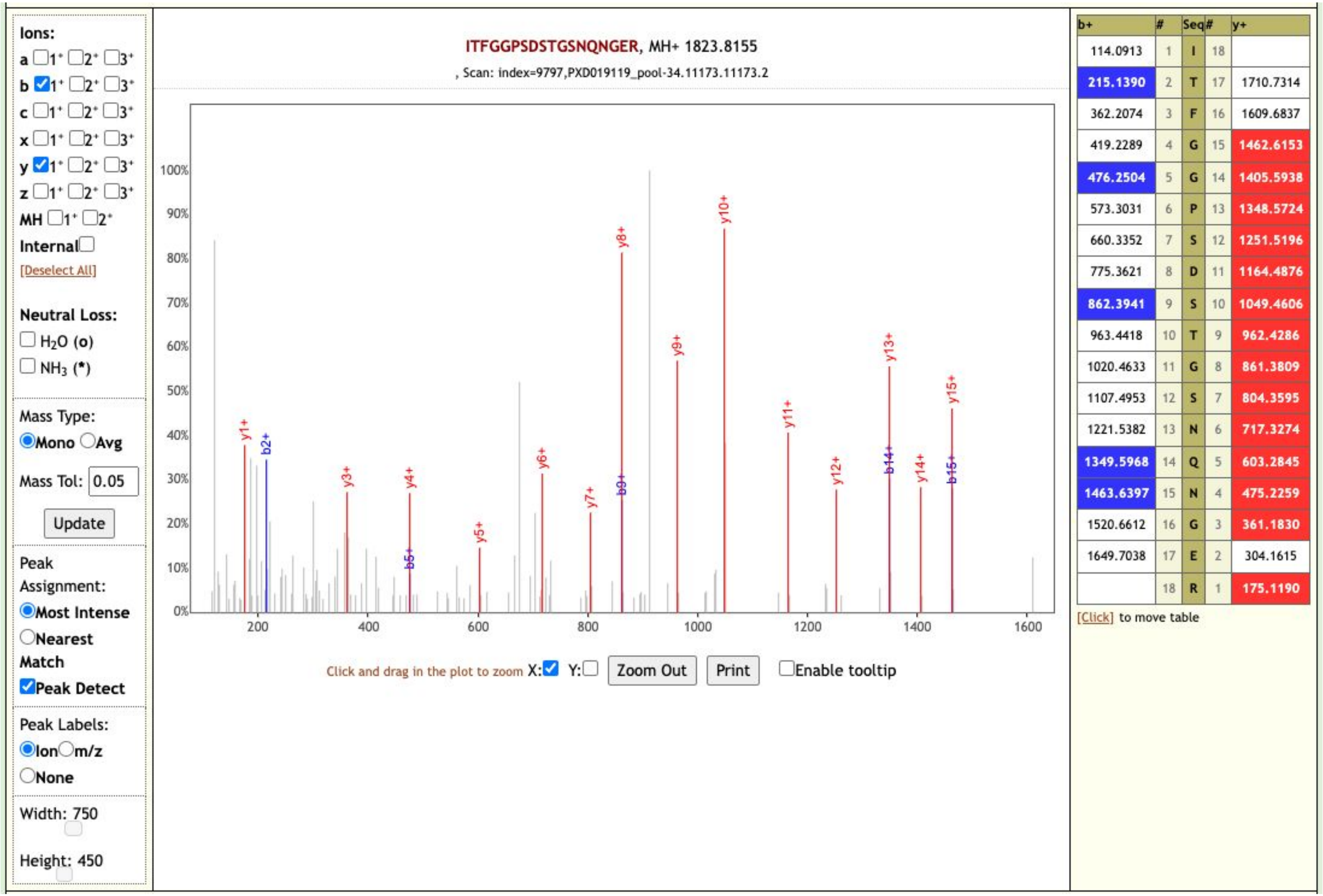

b+	#	Seq	#	y+
114.0913	1	I	18	
215.1390	2	T	17	1710.7314
362.2074	3	F	16	1609.6837
419.2289	4	G	15	1462.6153
476.2504	5	G	14	1405.5938
573.3031	6	P	13	1348.5724
660.3352	7	S	12	1251.5196
775.3621	8	D	11	1164.4876
862.3941	9	S	10	1049.4606
963.4418	10	T	9	962.4286
1020.4633	11	G	8	861.3809
1107.4953	12	S	7	804.3595
1221.5382	13	N	6	717.3274
1349.5968	14	Q	5	603.2845
1463.6397	15	N	4	475.2259
1520.6612	16	G	3	361.1830
1649.7038	17	E	2	304.1615
	18	R	1	175.1190

LDDKDPNFK

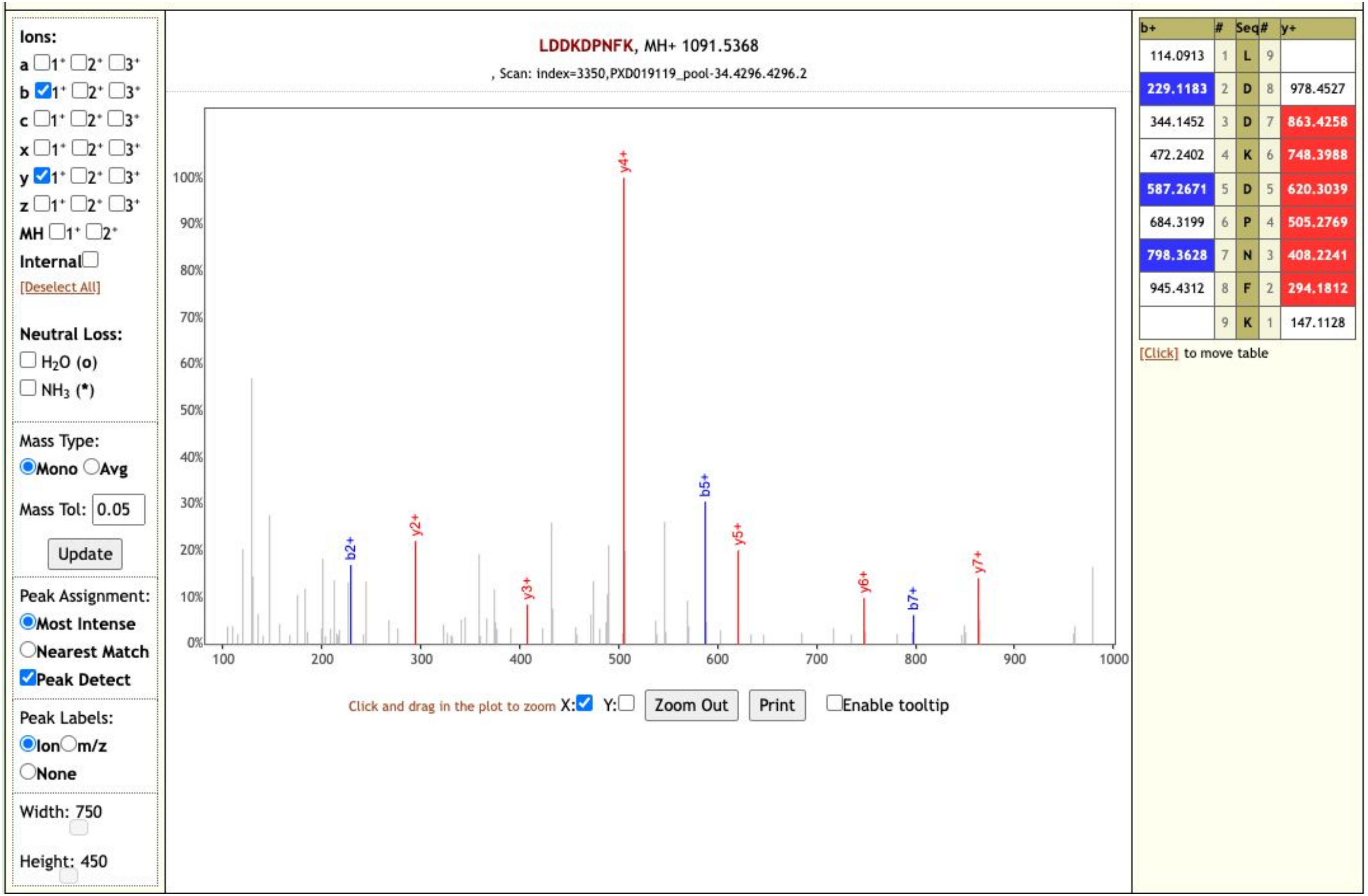

b+	#	Seq	#	y+
114.0913	1	L	9	
229.1183	2	D	8	978.4527
344.1452	3	D	7	863.4258
472.2402	4	K	6	748.3988
587.2671	5	D	5	620.3039
684.3199	6	P	4	505.2769
798.3628	7	N	3	408.2241
945.4312	8	F	2	294.1812
	9	K	1	147.1128

IGMEVTPSGTWLTYTGAIK

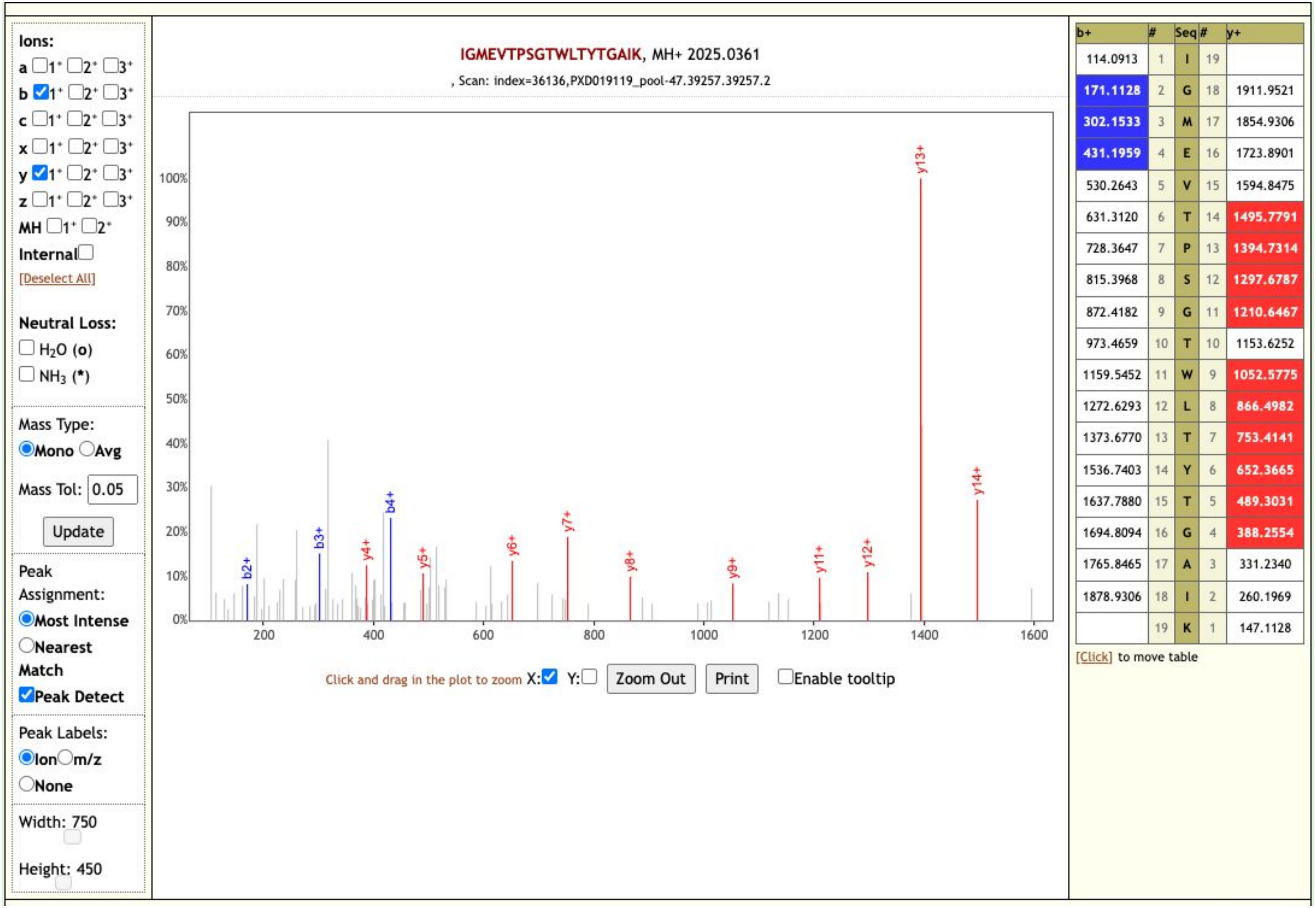

b+	#	Seq	#	y+
114.0913	1	I	19	
171.1128	2	G	18	1911.9521
302.1533	3	M	17	1854.9306
431.1959	4	E	16	1723.8901
530.2643	5	V	15	1594.8475
631.3120	6	T	14	1495.7791
728.3647	7	P	13	1394.7314
815.3968	8	S	12	1297.6787
872.4182	9	G	11	1210.6467
973.4659	10	T	10	1153.6252
1159.5452	11	W	9	1052.5775
1272.6293	12	L	8	866.4982
1373.6770	13	T	7	753.4141
1536.7403	14	Y	6	652.3665
1637.7880	15	T	5	489.3031
1694.8094	16	G	4	388.2554
1765.8465	17	A	3	331.2340
1878.9306	18	I	2	260.1969
	19	K	1	147.1128

LQDVVNQNAQALNTLVK

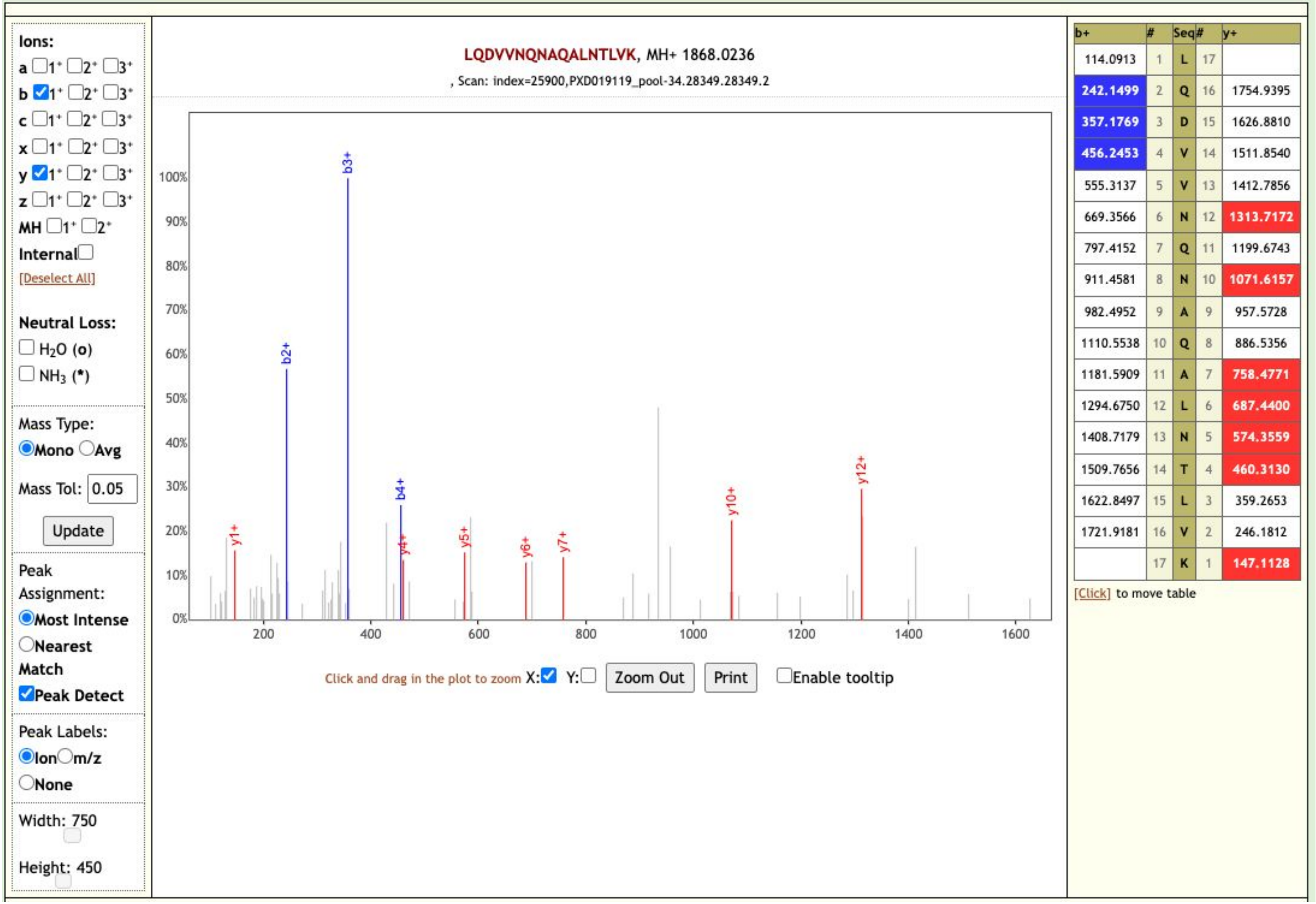

KKADETQALPQR

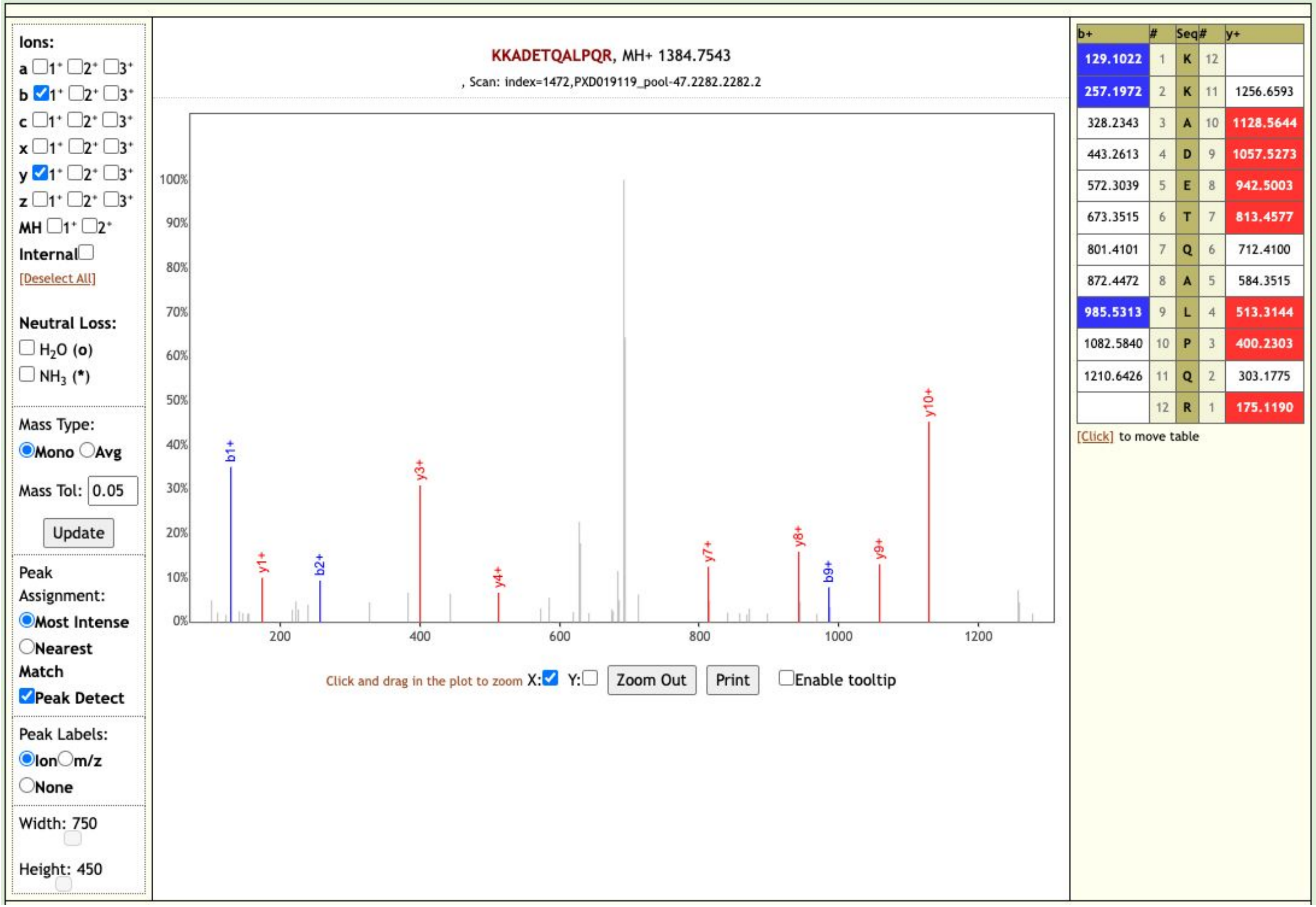

b+	#	Seq	#	y+
129.1022	1	K	12	
257.1972	2	K	11	1256.6593
328.2343	3	A	10	1128.5644
443.2613	4	D	9	1057.5273
572.3039	5	E	8	942.5003
673.3515	6	T	7	813.4577
801.4101	7	Q	6	712.4100
872.4472	8	A	5	584.3515
985.5313	9	L	4	513.3144
1082.5840	10	P	3	400.2303
1210.6426	11	Q	2	303.1775
	12	R	1	175.1190

KSNLKPFER

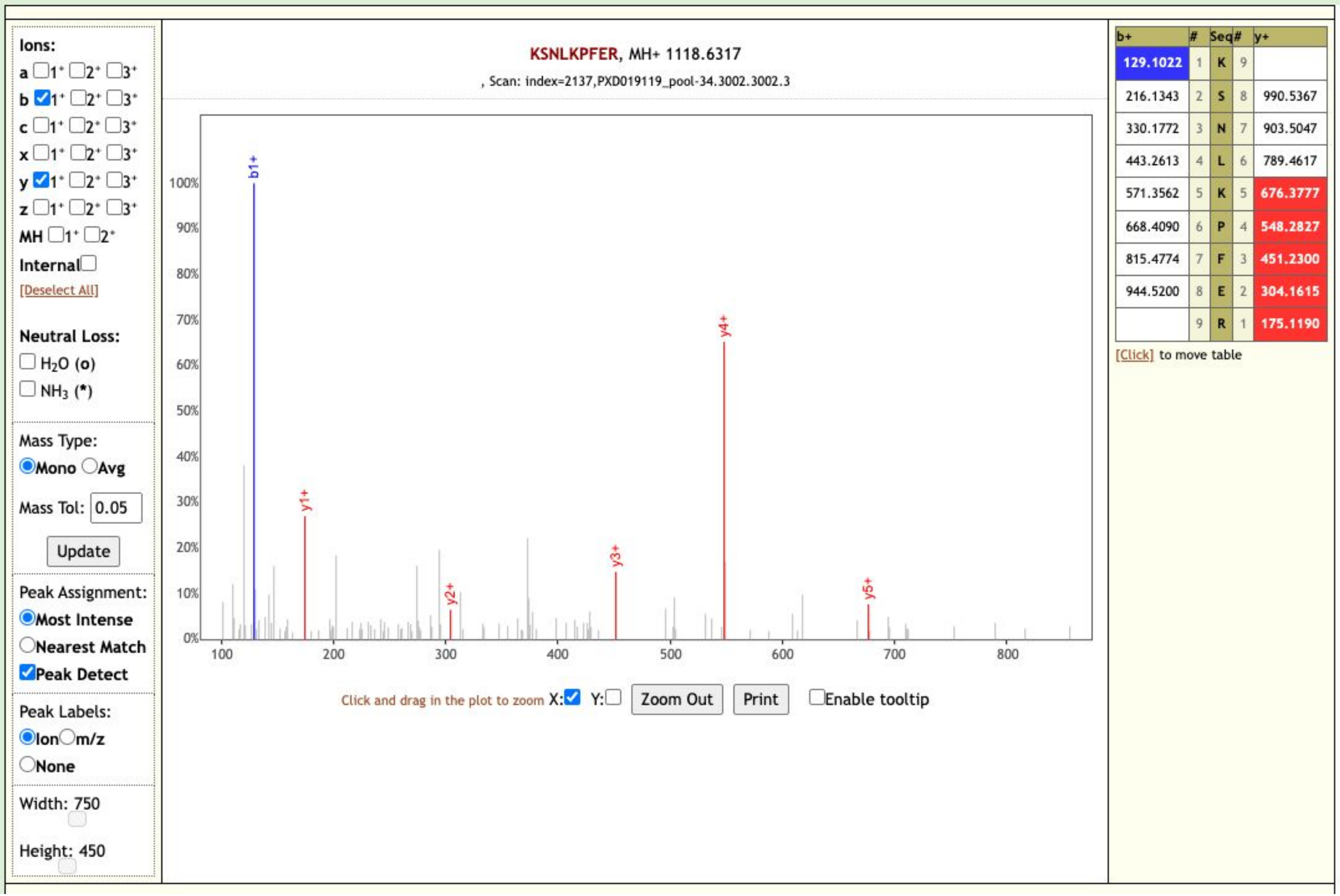
Ions:
a 1+ 2+ 3+
b 1+ 2+ 3+
c 1+ 2+ 3+
x 1+ 2+ 3+
y 1+ 2+ 3+
z 1+ 2+ 3+
MH 1+ 2+
Internal
[Deselect All]
Neutral Loss:
H2O (o)
NH3 (*)
Mass Type:
Mono Avg
Mass Tol: 0.05
Update
Peak Assignment:
Most Intense
Nearest Match
Peak Detect
Peak Labels:
Ion m/z
None
Width: 750
Height: 450
KSNLKPFER, MH+ 1118.6317
, Scan: index=2137,PXD019119_pool-34.3002.3002.3
b1+
y1+
y2+
y3+
y4+
y5+
100%
90%
80%
70%
60%
50%
40%
30%
20%
10%
0%
100
200
300
400
500
600
700
800
Click and drag in the plot to zoom X: Y:
Zoom Out
Print
Enable tooltip
b+ # Seq # y+
129.1022 1 K 9
216.1343 2 S 8 990.5367
330.1772 3 N 7 903.5047
443.2613 4 L 6 789.4617
571.3562 5 K 5 676.3777
668.4090 6 P 4 548.2827
815.4774 7 F 3 451.2300
944.5200 8 E 2 304.1615
9 R 1 175.1190
[Click] to move table

LGTGPEAGLPYGANK

Ions:
a 1+ 2+ 3+
b 1+ 2+ 3+
c 1+ 2+ 3+
x 1+ 2+ 3+
y 1+ 2+ 3+
z 1+ 2+ 3+
MH 1+ 2+
Internal
[Deselect All]

Neutral Loss:
H_2O (o)
NH_3 (*)

Mass Type:
Mono Avg

Mass Tol: 0.05

Update

Peak Assignment:
Most Intense
Nearest Match
Peak Detect

Peak Labels:
Ion m/z
None

Width: 750

Height: 450

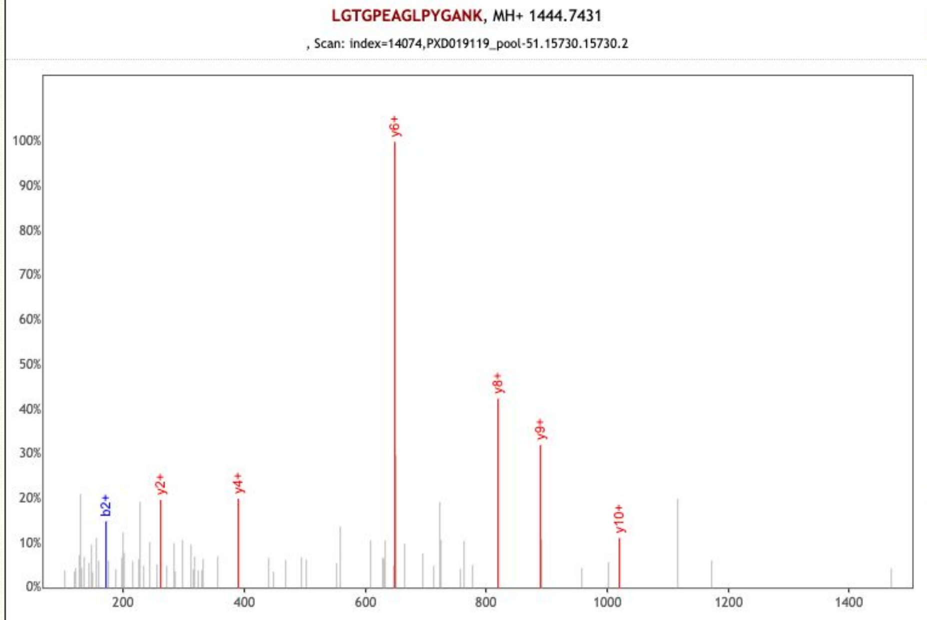

Click and drag in the plot to zoom X: Y: Zoom Out Print Enable tooltip

b+	#	Seq	#	y+
114.0913	1	L	15	
171.1128	2	G	14	1331.6590
272.1605	3	T	13	1274.6375
329.1819	4	G	12	1173.5899
426.2347	5	P	11	1116.5684
555.2773	6	E	10	1019.5156
626.3144	7	A	9	890.4730
683.3359	8	G	8	819.4359
796.4199	9	L	7	762.4145
893.4727	10	P	6	649.3304
1056.5360	11	Y	5	552.2776
1113.5575	12	G	4	389.2143
1184.5946	13	A	3	332.1928
1298.6375	14	N	2	261.1557
	15	K	1	147.1128

[Click] to move table

IAGHHLGR

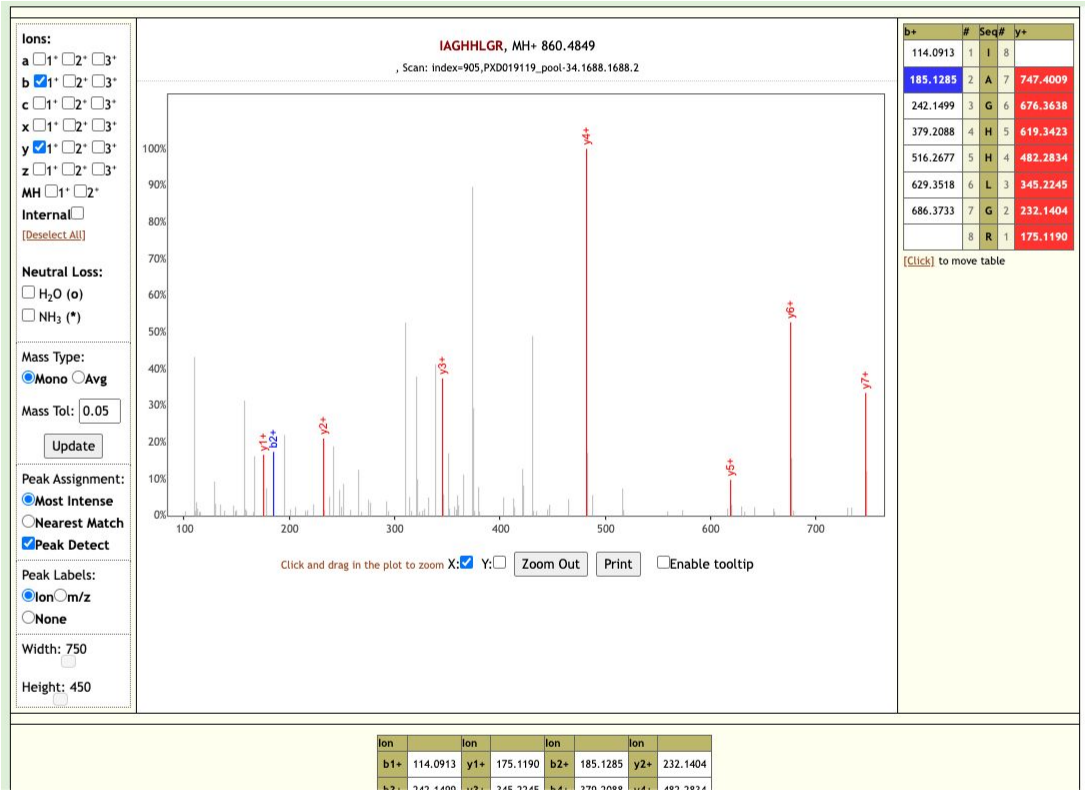
IAGHHLGR, MH+ 860.4849
, Scan: index=905,PXD019119_pool-34.1688.1688.2
Ions:
Neutral Loss:
Mass Type:
Mono Avg
Mass Tol: 0.05
Update
Peak Assignment:
Most Intense
Nearest Match
Peak Detect
Peak Labels:
Ion m/z
None
Width: 750
Height: 450
[Deselect All]
y1+ b2+ y2+ y3+ y4+ y5+ y6+ y7+
Click and drag in the plot to zoom
Zoom Out
Print
Enable tooltip
b+ # Seq # y+
114.0913 1 I 8
185.1285 2 A 7 747.4009
242.1499 3 G 6 676.3638
379.2088 4 H 5 619.3423
516.2677 5 H 4 482.2834
629.3518 6 L 3 345.2245
686.3733 7 G 2 232.1404
8 R 1 175.1190
[Click] to move table
Ion Ion Ion Ion
b1+ 114.0913 y1+ 175.1190 b2+ 185.1285 y2+ 232.1404

DDKDPNFKDQVILLNK

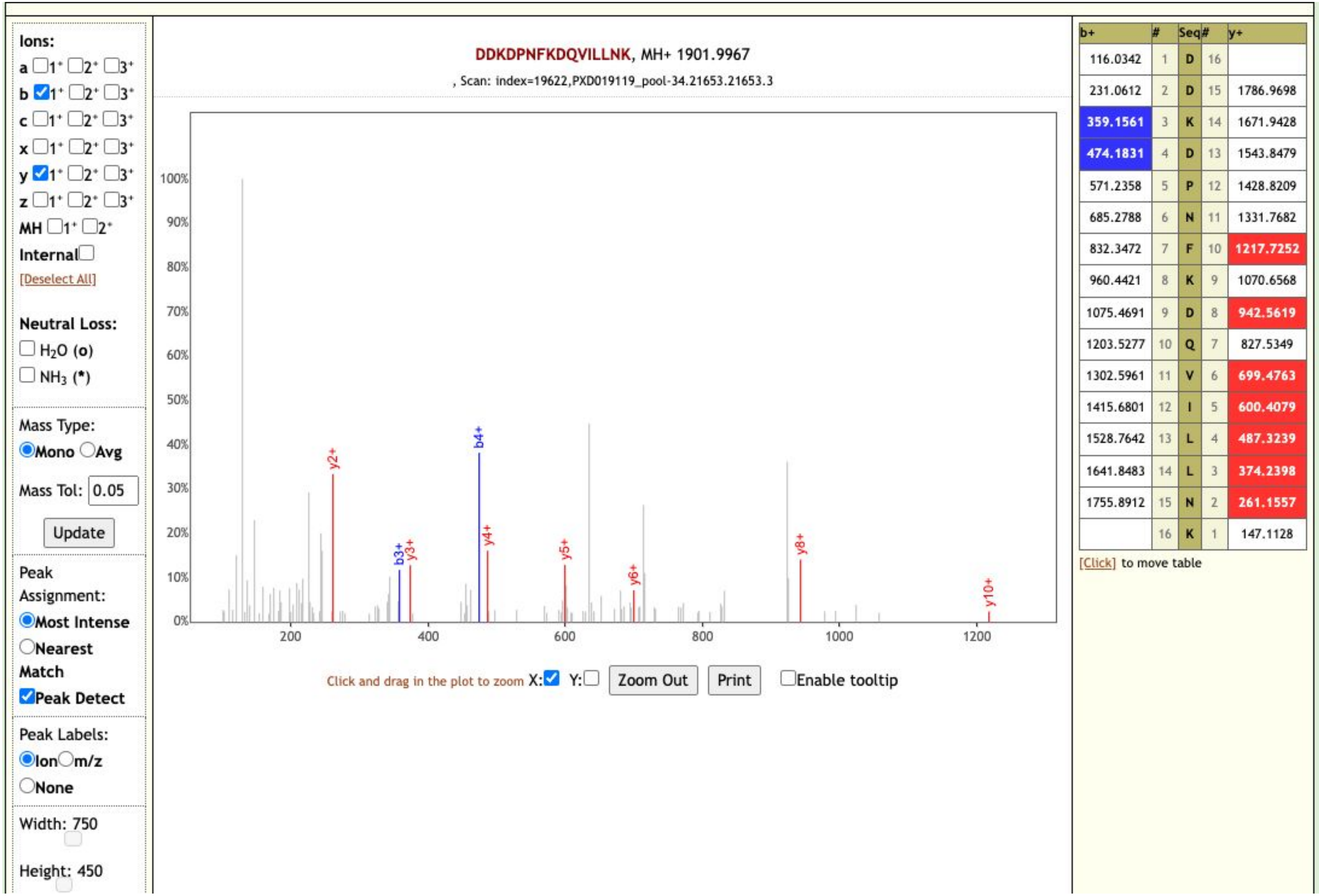

b+	#	Seq	#	y+
116.0342	1	D	16	
231.0612	2	D	15	1786.9698
359.1561	3	K	14	1671.9428
474.1831	4	D	13	1543.8479
571.2358	5	P	12	1428.8209
685.2788	6	N	11	1331.7682
832.3472	7	F	10	1217.7252
960.4421	8	K	9	1070.6568
1075.4691	9	D	8	942.5619
1203.5277	10	Q	7	827.5349
1302.5961	11	V	6	699.4763
1415.6801	12	I	5	600.4079
1528.7642	13	L	4	487.3239
1641.8483	14	L	3	374.2398
1755.8912	15	N	2	261.1557
	16	K	1	147.1128

[Click] to move table

FDNPVLPFNDGVYFASTEK

Ions:
a 1+ 2+ 3+
b 1+ 2+ 3+
c 1+ 2+ 3+
x 1+ 2+ 3+
y 1+ 2+ 3+
z 1+ 2+ 3+
MH 1+ 2+
Internal
[Deselect All]

Neutral Loss:
H_2O (o)
NH_3 (*)

Mass Type:
Mono Avg

Mass Tol: 0.05

Update

Peak Assignment:
Most Intense
Nearest Match
Peak Detect

Peak Labels:
Ion m/z
None

Width: 750

Height: 450

FDNPVLPFNDGVYFASTEK, MH+ 2160.0284

, Scan: index=36929,PXD019119_pool-47.40102.40102.2

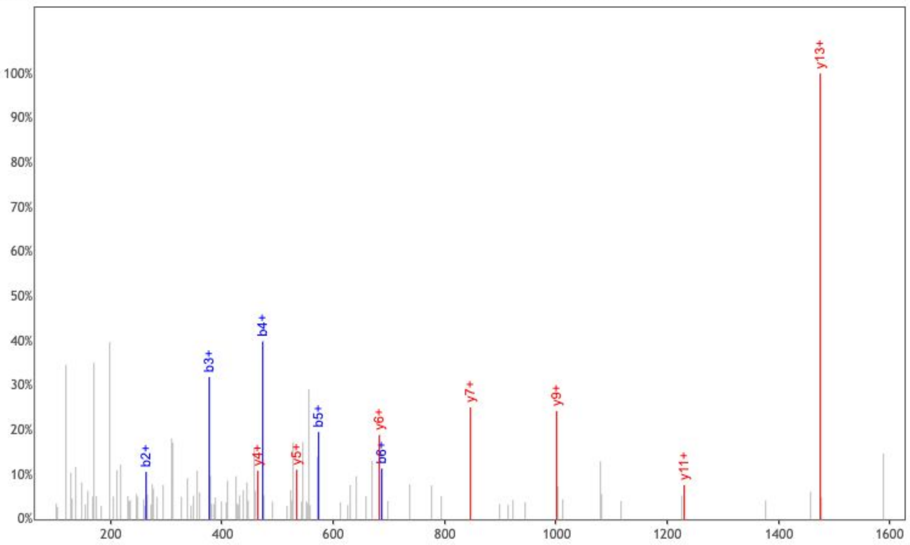

Click and drag in the plot to zoom X: Y: Zoom Out Print Enable tooltip

b+	#	Seq	#	y+
148.0757	1	F	19	
263.1026	2	D	18	2012.9600
377.1456	3	N	17	1897.9331
474.1983	4	P	16	1783.8901
573.2667	5	V	15	1686.8374
686.3508	6	L	14	1587.7690
783.4036	7	P	13	1474.6849
930.4720	8	F	12	1377.6321
1044.5149	9	N	11	1230.5637
1159.5419	10	D	10	1116.5208
1216.5633	11	G	9	1001.4938
1315.6317	12	V	8	944.4724
1478.6951	13	Y	7	845.4040
1625.7635	14	F	6	682.3406
1696.8006	15	A	5	535.2722
1783.8326	16	S	4	464.2351
1884.8803	17	T	3	377.2031
2013.9229	18	E	2	276.1554
	19	K	1	147.1128

[Click] to move table

FNGIGVTQNVLYENQK

Ions:
a 1+ 2+ 3+
b 1+ 2+ 3+
c 1+ 2+ 3+
x 1+ 2+ 3+
y 1+ 2+ 3+
z 1+ 2+ 3+
MH 1+ 2+
Internal
[Deselect All]

Neutral Loss:
H_2O (o)
NH_3 (*)

Mass Type:
Mono Avg

Mass Tol: 0.05

Update

Peak Assignment:
Most Intense
Nearest Match
Peak Detect

Peak Labels:
Ion m/z
None

Width: 750

Height: 450

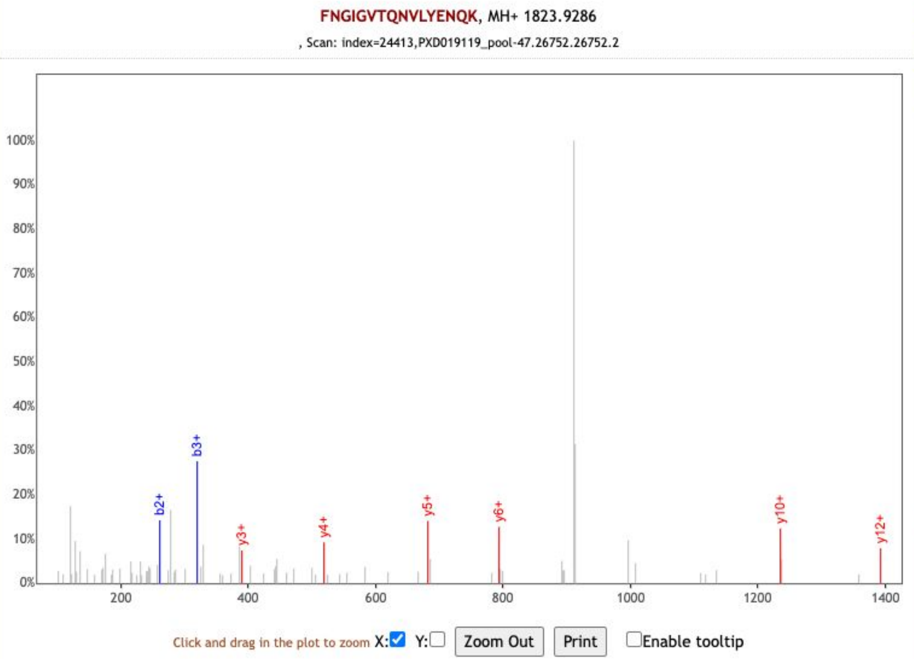

b+	#	Seq	#	y+
148.0757	1	F	16	
262.1186	2	N	15	1676.8602
319.1401	3	G	14	1562.8173
432.2241	4	I	13	1505.7958
489.2456	5	G	12	1392.7118
588.3140	6	V	11	1335.6903
689.3617	7	T	10	1236.6219
817.4203	8	Q	9	1135.5742
931.4632	9	N	8	1007.5156
1030.5316	10	V	7	893.4727
1143.6157	11	L	6	794.4043
1306.6790	12	Y	5	681.3202
1435.7216	13	E	4	518.2569
1549.7645	14	N	3	389.2143
1677.8231	15	Q	2	275.1714
	16	K	1	147.1128

[Click] to move table

GPEQTQGNFGDQELIR

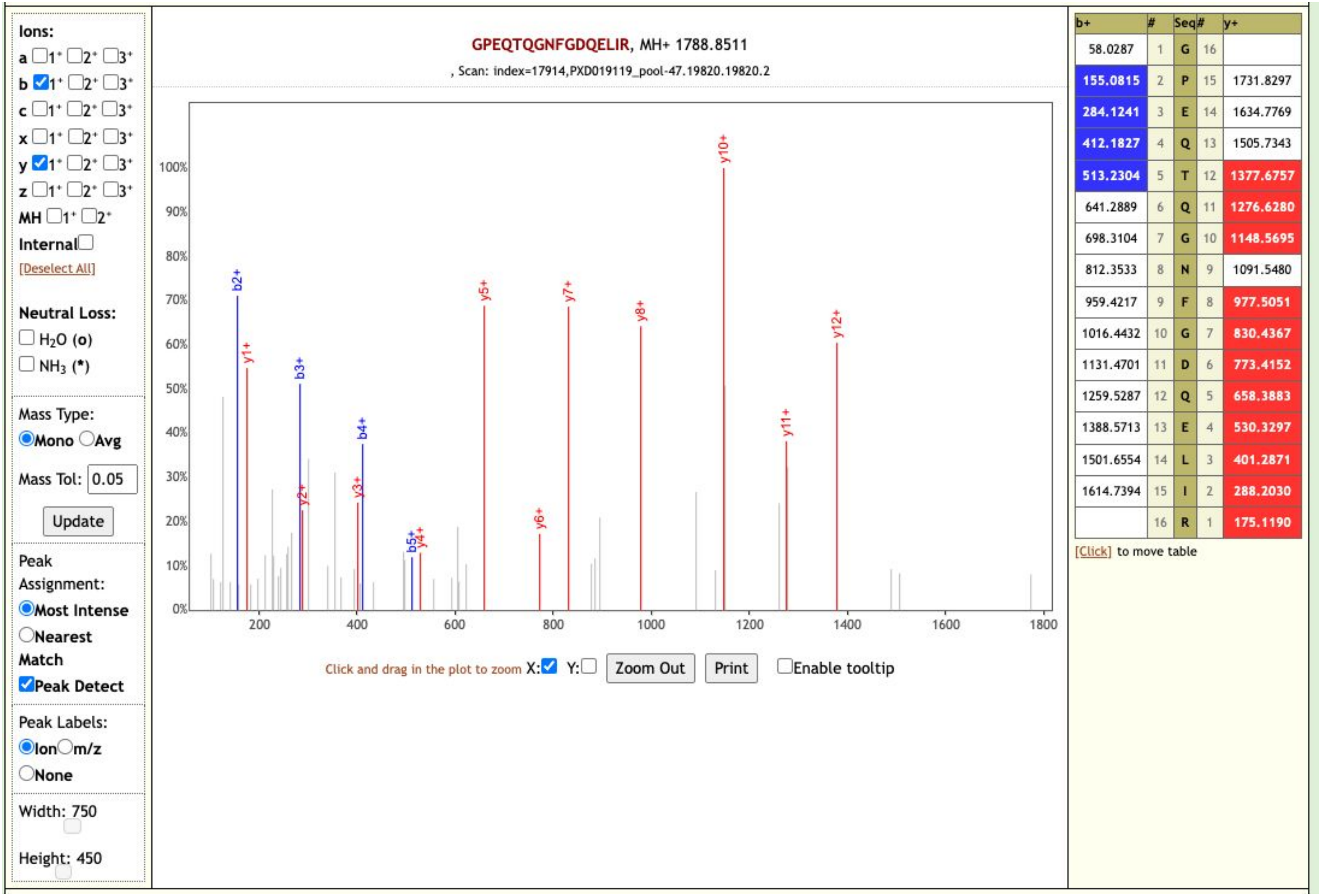

b+	#	Seq	#	y+
58.0287	1	G	16	
155.0815	2	P	15	1731.8297
284.1241	3	E	14	1634.7769
412.1827	4	Q	13	1505.7343
513.2304	5	T	12	1377.6757
641.2889	6	Q	11	1276.6280
698.3104	7	G	10	1148.5695
812.3533	8	N	9	1091.5480
959.4217	9	F	8	977.5051
1016.4432	10	G	7	830.4367
1131.4701	11	D	6	773.4152
1259.5287	12	Q	5	658.3883
1388.5713	13	E	4	530.3297
1501.6554	14	L	3	401.2871
1614.7394	15	I	2	288.2030
	16	R	1	175.1190

GQGVPINTNSSPDDQIGY

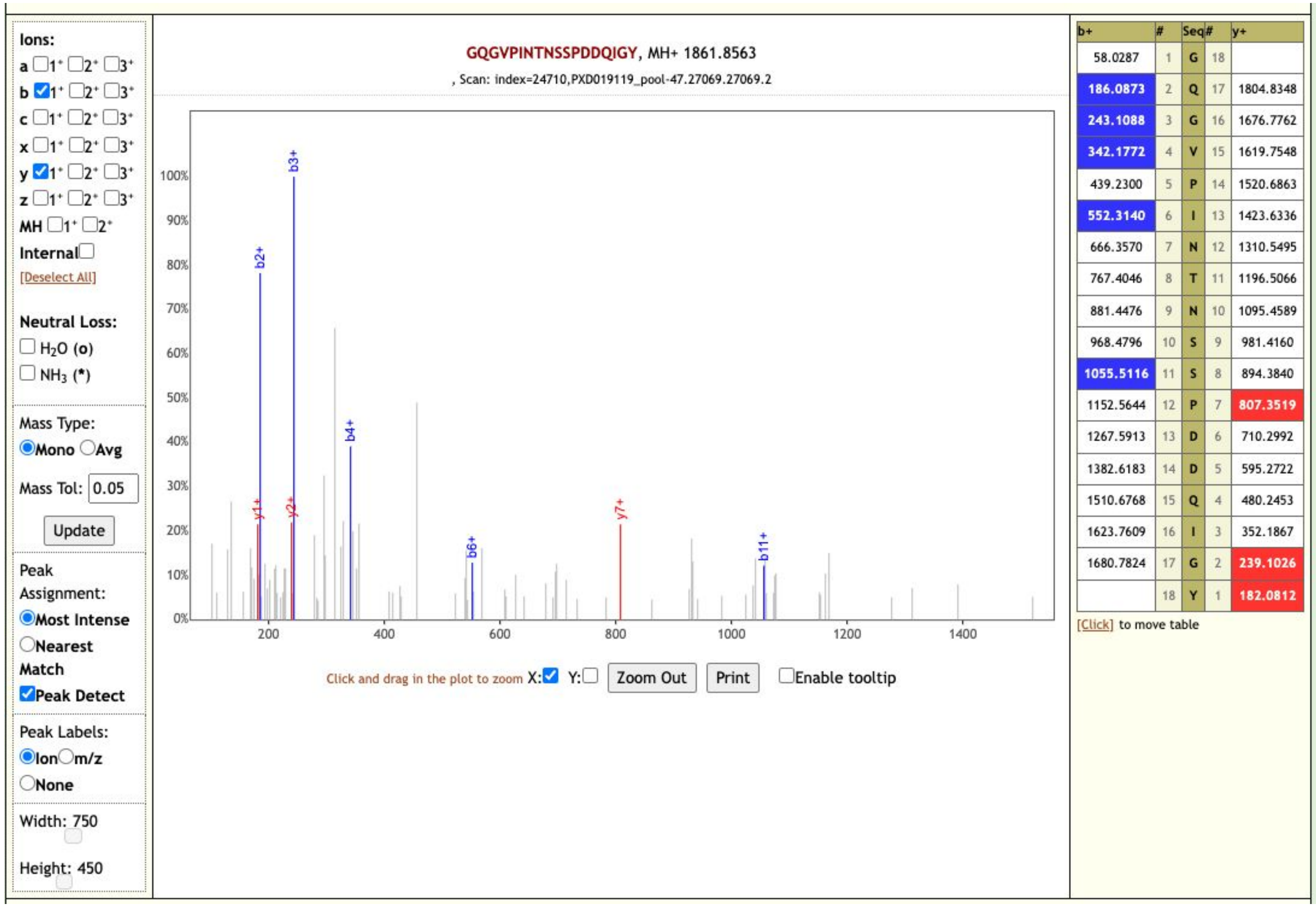

b+	#	Seq	#	y+
58.0287	1	G	18	
186.0873	2	Q	17	1804.8348
243.1088	3	G	16	1676.7762
342.1772	4	V	15	1619.7548
439.2300	5	P	14	1520.6863
552.3140	6	I	13	1423.6336
666.3570	7	N	12	1310.5495
767.4046	8	T	11	1196.5066
881.4476	9	N	10	1095.4589
968.4796	10	S	9	981.4160
1055.5116	11	S	8	894.3840
1152.5644	12	P	7	807.3519
1267.5913	13	D	6	710.2992
1382.6183	14	D	5	595.2722
1510.6768	15	Q	4	480.2453
1623.7609	16	I	3	352.1867
1680.7824	17	G	2	239.1026
	18	Y	1	182.0812

[Click] to move table

LQSLQTYVTQQLIR

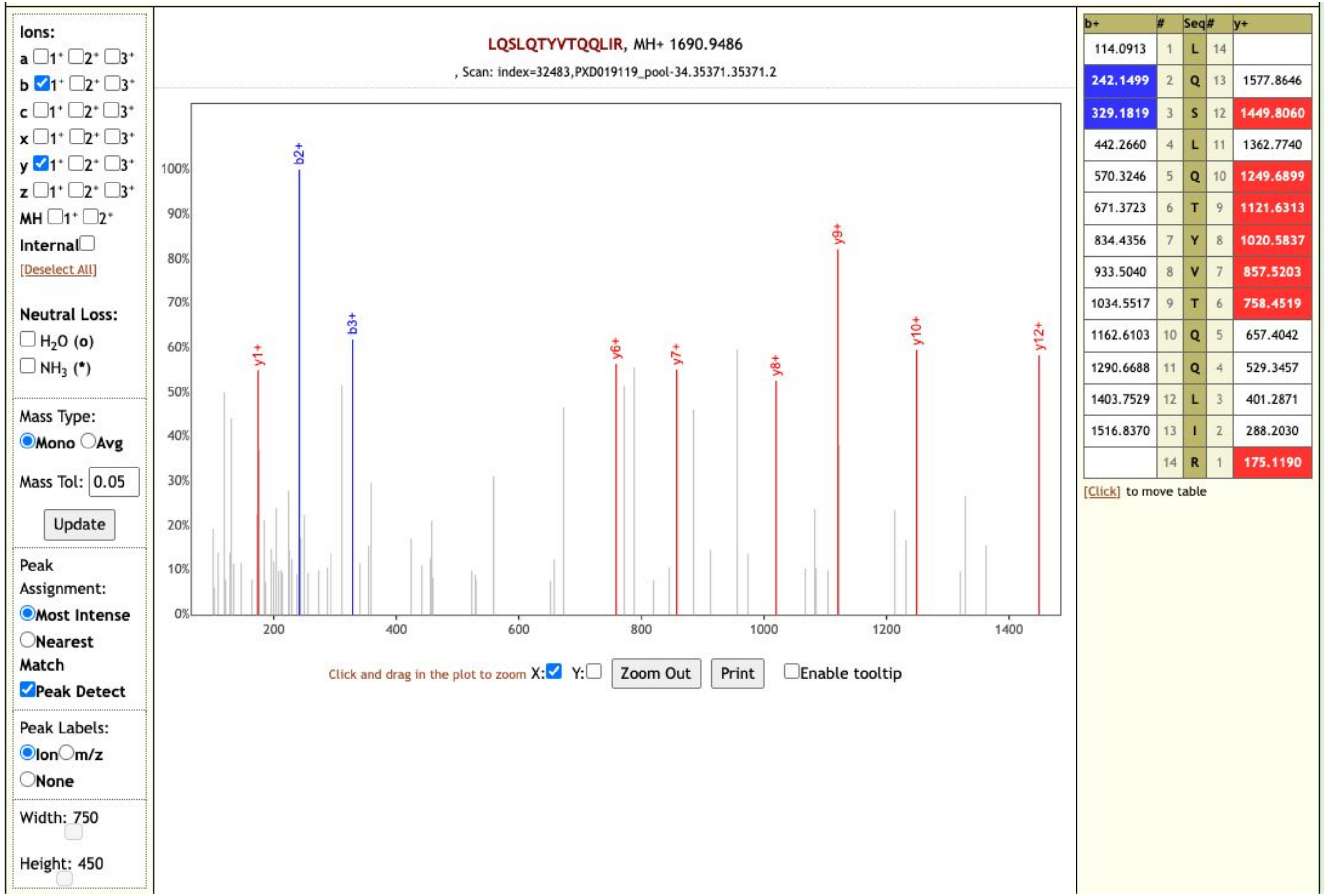

b+	#	Seq	#	y+
114.0913	1	L	14	
242.1499	2	Q	13	1577.8646
329.1819	3	S	12	1449.8060
442.2660	4	L	11	1362.7740
570.3246	5	Q	10	1249.6899
671.3723	6	T	9	1121.6313
834.4356	7	Y	8	1020.5837
933.5040	8	V	7	857.5203
1034.5517	9	T	6	758.4519
1162.6103	10	Q	5	657.4042
1290.6688	11	Q	4	529.3457
1403.7529	12	L	3	401.2871
1516.8370	13	I	2	288.2030
	14	R	1	175.1190

[Click] to move table

LTYTGAIK

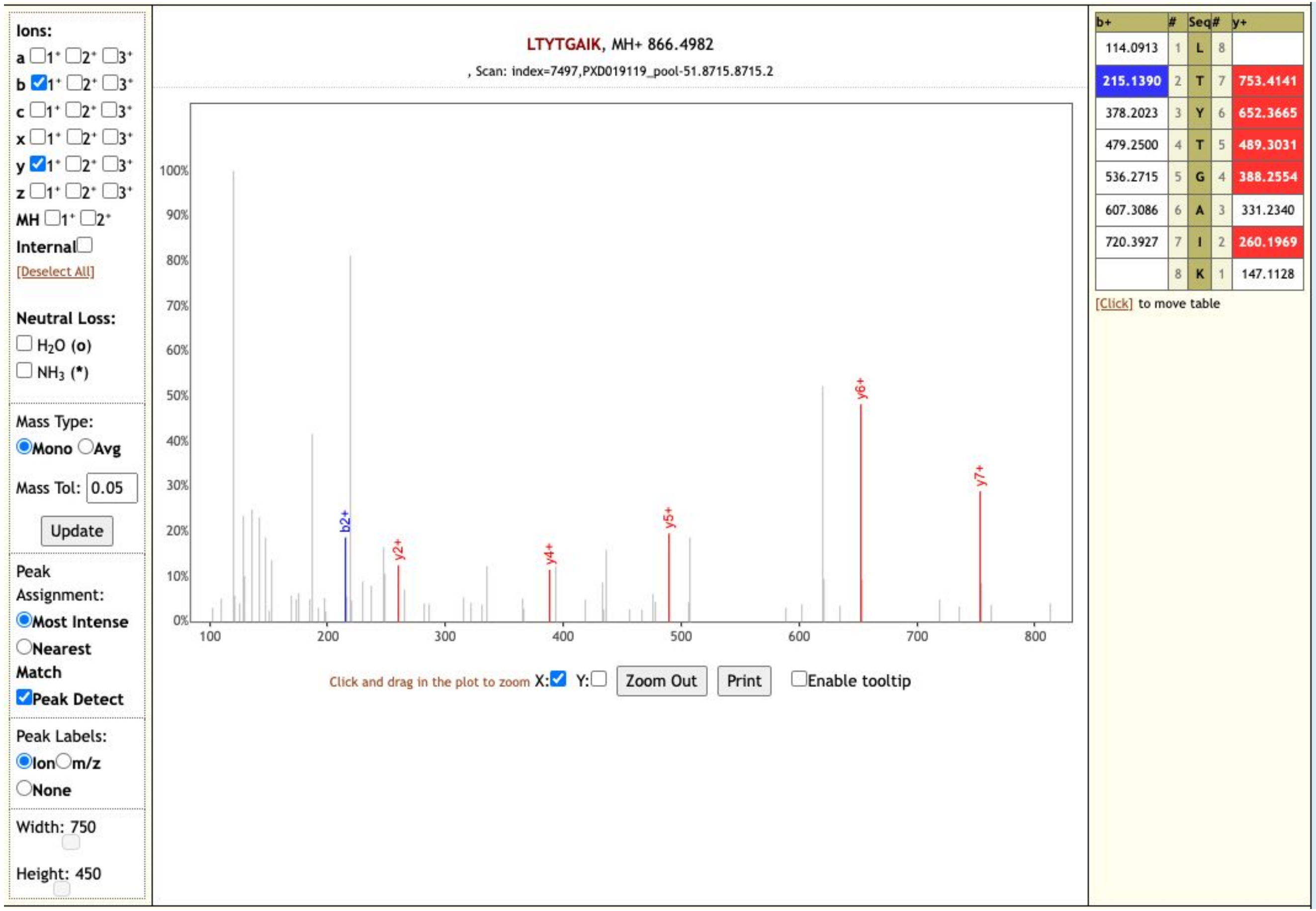

b+	#	Seq	#	y+
114.0913	1	L	8	
215.1390	2	T	7	753.4141
378.2023	3	Y	6	652.3665
479.2500	4	T	5	489.3031
536.2715	5	G	4	388.2554
607.3086	6	A	3	331.2340
720.3927	7	I	2	260.1969
	8	K	1	147.1128

GFQPTNGVGYQPYR

Ions:
a 1+ 2+ 3+
b ☑1+ 2+ 3+
c 1+ 2+ 3+
x 1+ 2+ 3+
y ☑1+ 2+ 3+
z 1+ 2+ 3+
MH 1+ 2+
Internal
[Deselect All]

Neutral Loss:
H_2O (o)
NH_3 (*)

Mass Type:
◉Mono ○Avg

Mass Tol: 0.05

Update

Peak Assignment:
◉Most Intense
○Nearest Match
☑Peak Detect

Peak Labels:
◉Ion ○m/z
○None

Width: 750

Height: 450

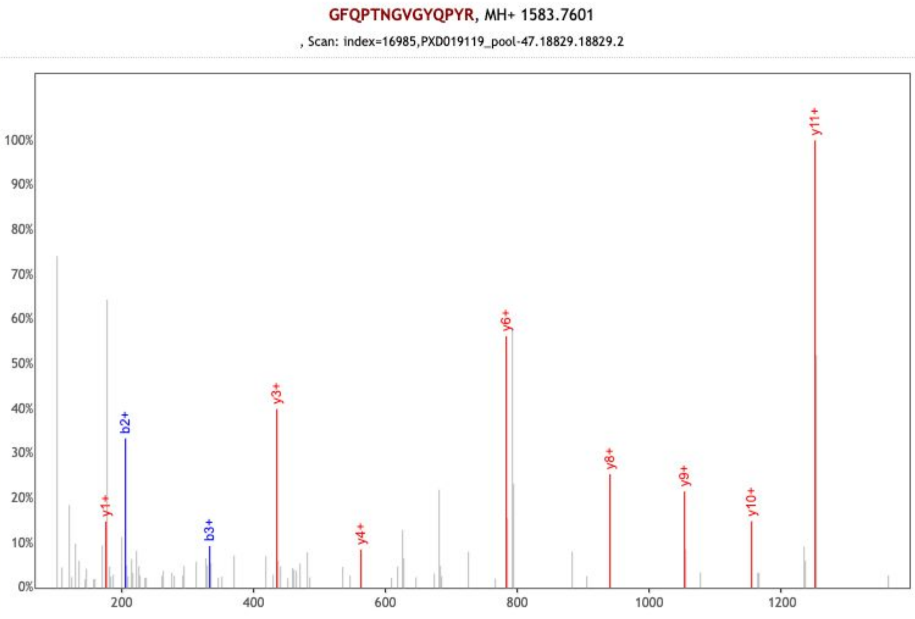

Click and drag in the plot to zoom X: ☑ Y: ☐ Zoom Out Print ☐Enable tooltip

b+	#	Seq	#	y+
58.0287	1	G	14	
205.0972	2	F	13	1526.7387
333.1557	3	Q	12	1379.6702
430.2085	4	P	11	1251.6117
531.2562	5	T	10	1154.5589
645.2991	6	N	9	1053.5112
702.3206	7	G	8	939.4683
801.3890	8	V	7	882.4468
858.4104	9	G	6	783.3784
1021.4738	10	Y	5	726.3570
1149.5323	11	Q	4	563.2936
1246.5851	12	P	3	435.2350
1409.6484	13	Y	2	338.1823
	14	R	1	175.1190

[Click] to move table

GPEQTQGNFGDQELTR

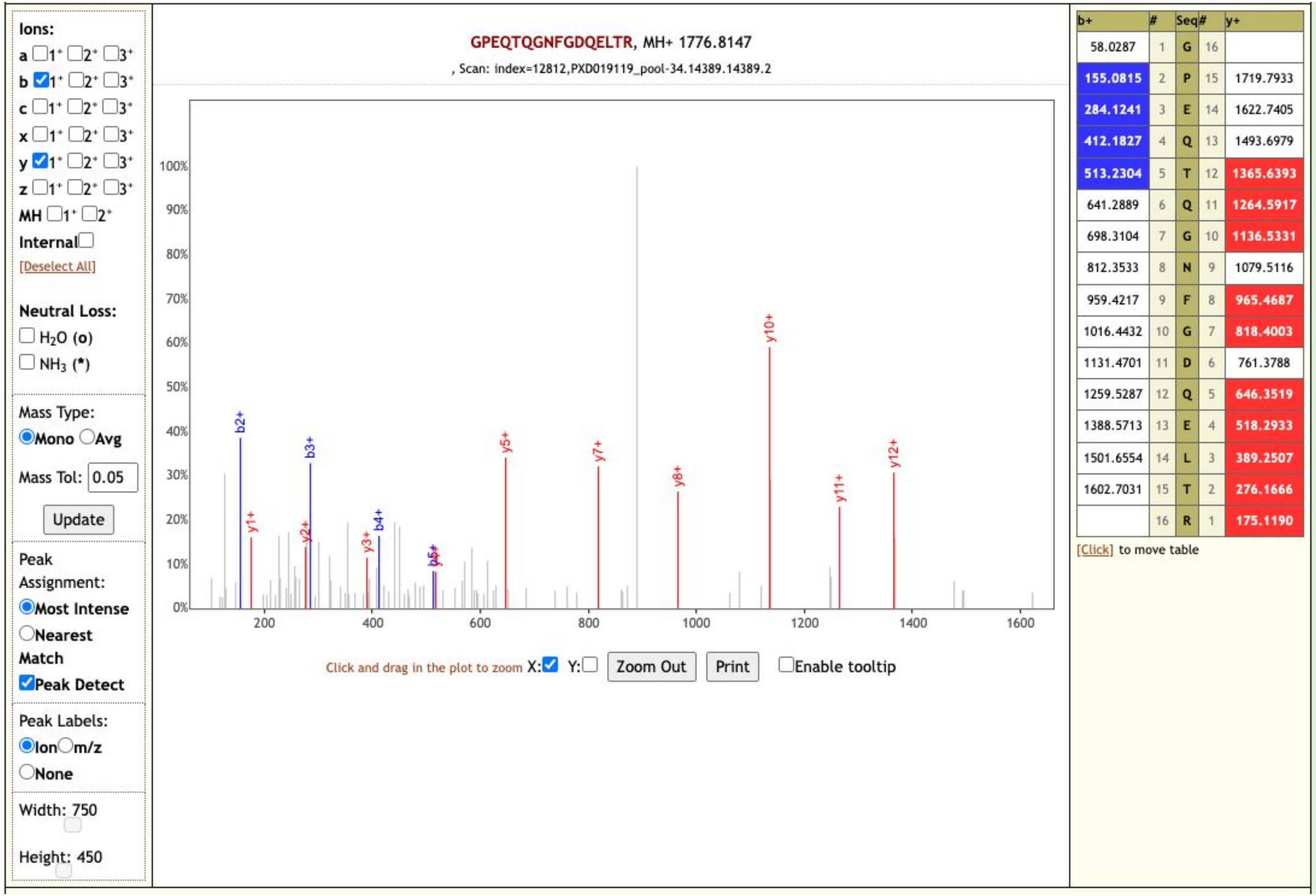

b+	#	Seq	#	y+
58.0287	1	G	16	
155.0815	2	P	15	1719.7933
284.1241	3	E	14	1622.7405
412.1827	4	Q	13	1493.6979
513.2304	5	T	12	1365.6393
641.2889	6	Q	11	1264.5917
698.3104	7	G	10	1136.5331
812.3533	8	N	9	1079.5116
959.4217	9	F	8	965.4687
1016.4432	10	G	7	818.4003
1131.4701	11	D	6	761.3788
1259.5287	12	Q	5	646.3519
1388.5713	13	E	4	518.2933
1501.6554	14	L	3	389.2507
1602.7031	15	T	2	276.1666
	16	R	1	175.1190

GGDAALALLLLDR

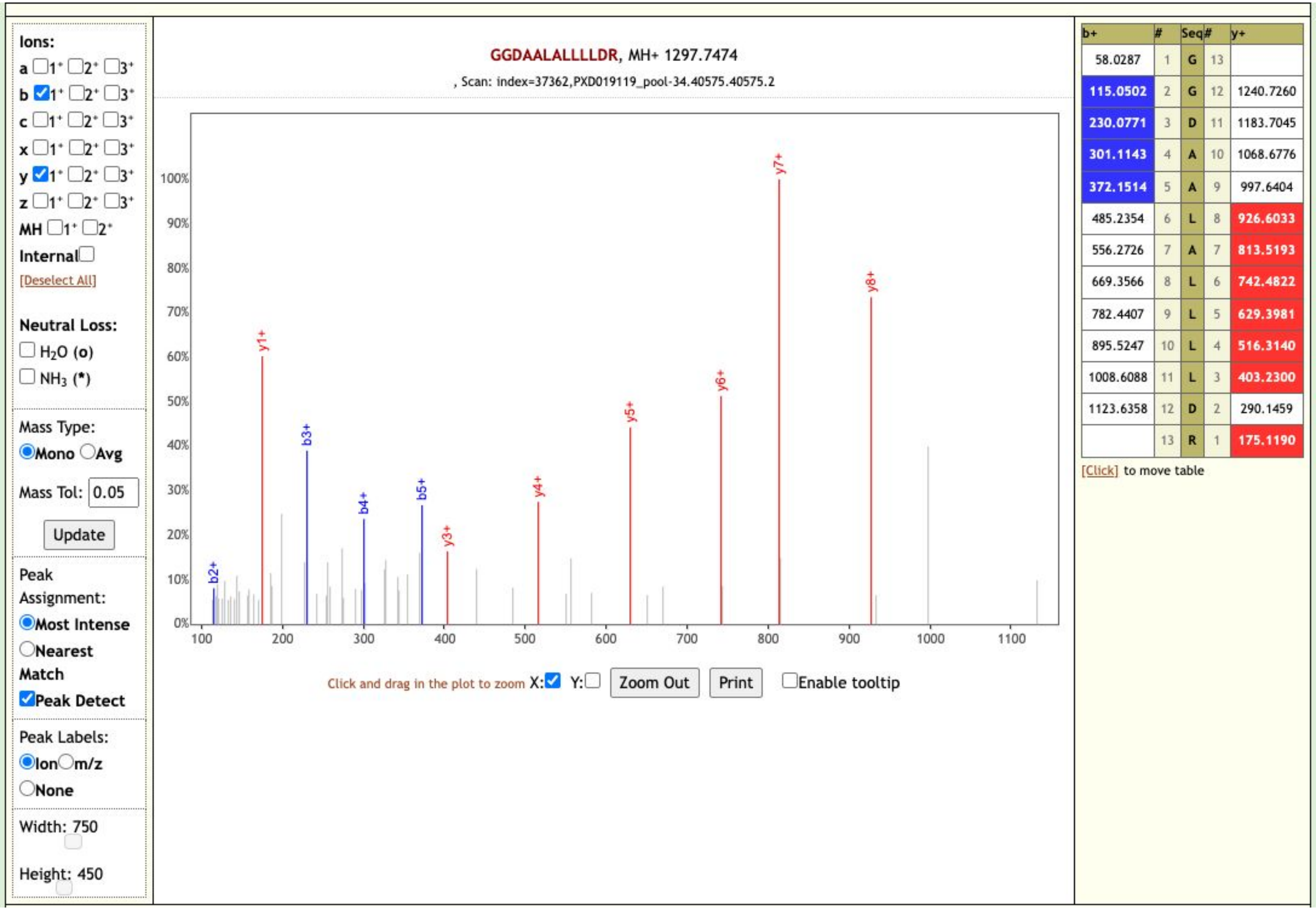

b+	#	Seq	#	y+
58.0287	1	G	13	
115.0502	2	G	12	1240.7260
230.0771	3	D	11	1183.7045
301.1143	4	A	10	1068.6776
372.1514	5	A	9	997.6404
485.2354	6	L	8	926.6033
556.2726	7	A	7	813.5193
669.3566	8	L	6	742.4822
782.4407	9	L	5	629.3981
895.5247	10	L	4	516.3140
1008.6088	11	L	3	403.2300
1123.6358	12	D	2	290.1459
	13	R	1	175.1190

[Click] to move table

IGMEVTPSGTWLTYTGAIK

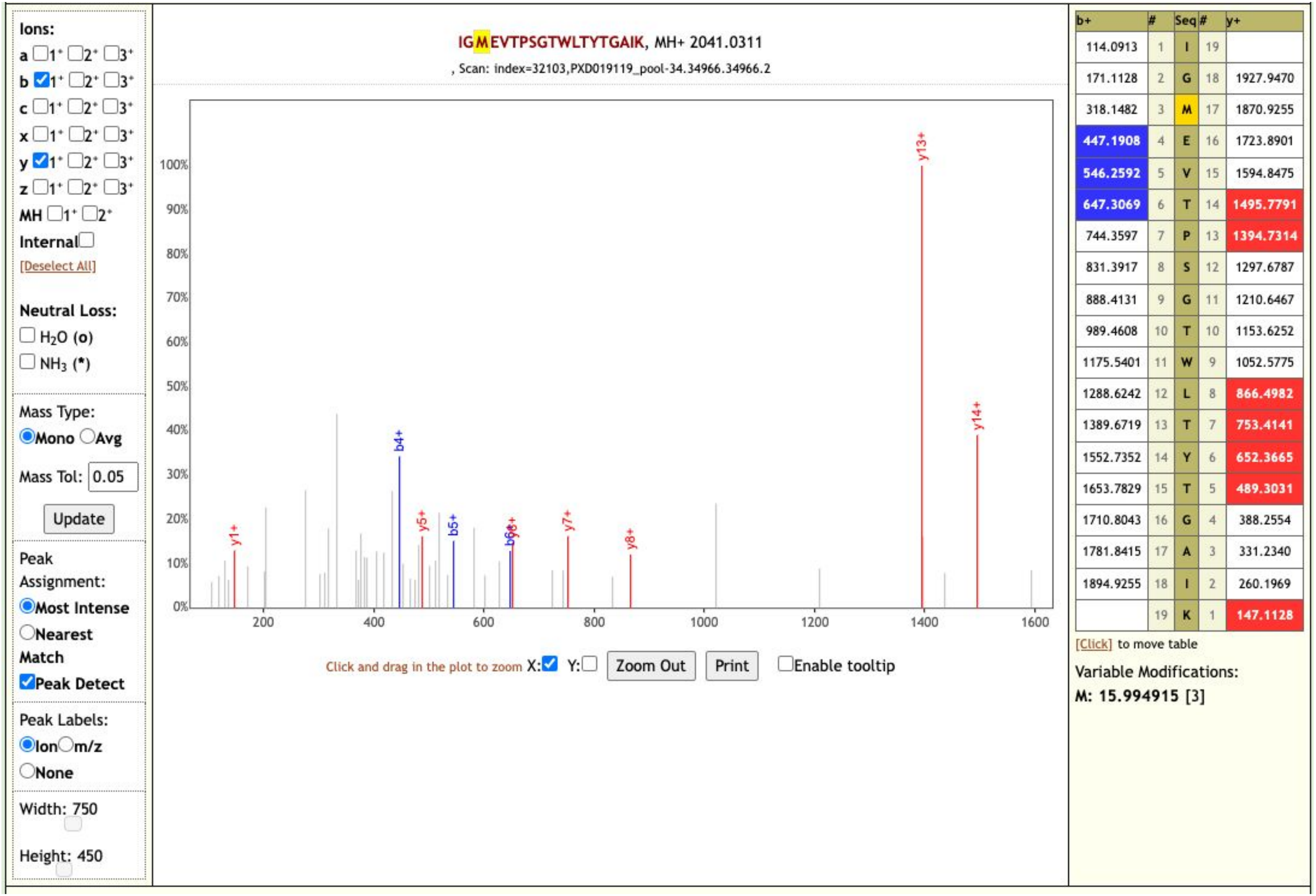

b+	#	Seq	#	y+
114.0913	1	I	19	
171.1128	2	G	18	1927.9470
318.1482	3	M	17	1870.9255
447.1908	4	E	16	1723.8901
546.2592	5	V	15	1594.8475
647.3069	6	T	14	1495.7791
744.3597	7	P	13	1394.7314
831.3917	8	S	12	1297.6787
888.4131	9	G	11	1210.6467
989.4608	10	T	10	1153.6252
1175.5401	11	W	9	1052.5775
1288.6242	12	L	8	866.4982
1389.6719	13	T	7	753.4141
1552.7352	14	Y	6	652.3665
1653.7829	15	T	5	489.3031
1710.8043	16	G	4	388.2554
1781.8415	17	A	3	331.2340
1894.9255	18	I	2	260.1969
	19	K	1	147.1128

[Click] to move table

Variable Modifications:

M: 15.994915 [3]

LIANQFNSAIGK

Ions:
a ☐1⁺ ☐2⁺ ☐3⁺
b ☑1⁺ ☐2⁺ ☐3⁺
c ☐1⁺ ☐2⁺ ☐3⁺
x ☐1⁺ ☐2⁺ ☐3⁺
y ☑1⁺ ☐2⁺ ☐3⁺
z ☐1⁺ ☐2⁺ ☐3⁺
MH ☐1⁺ ☐2⁺
Internal ☐
[Deselect All]

Neutral Loss:
☐ H_2O (o)
☐ NH_3 (*)

Mass Type: ◉Mono ○Avg

Mass Tol: 0.05

Update

Peak Assignment:
◉Most Intense
○Nearest Match
☑Peak Detect

Peak Labels: ◉Ion ○m/z ○None

Width: 750

Height: 450

LIANQFNSAIGK, MH+ 1275.7056

, Scan: index=16743,PXD019119_pool-34.18582.18582.2

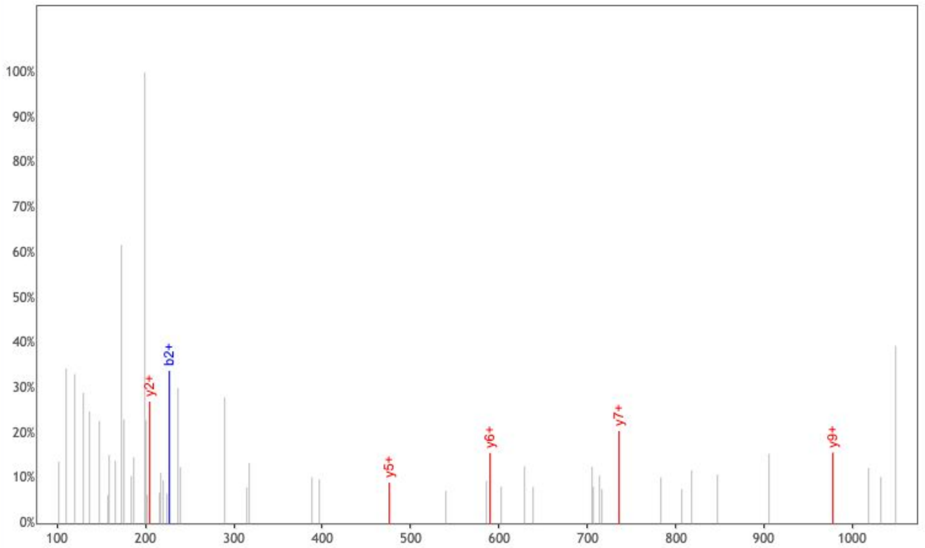

Click and drag in the plot to zoom X:☑ Y:☐ Zoom Out Print ☐Enable tooltip

b+	#	Seq	#	y+
114.0913	1	L	12	
227.1754	2	I	11	1162.6215
298.2125	3	A	10	1049.5374
412.2554	4	N	9	978.5003
540.3140	5	Q	8	864.4574
687.3824	6	F	7	736.3988
801.4254	7	N	6	589.3304
888.4574	8	S	5	475.2875
959.4945	9	A	4	388.2554
1072.5786	10	I	3	317.2183
1129.6000	11	G	2	204.1343
	12	K	1	147.1128

[Click] to move table

RPQGLPNNTASWFTALTQHGK

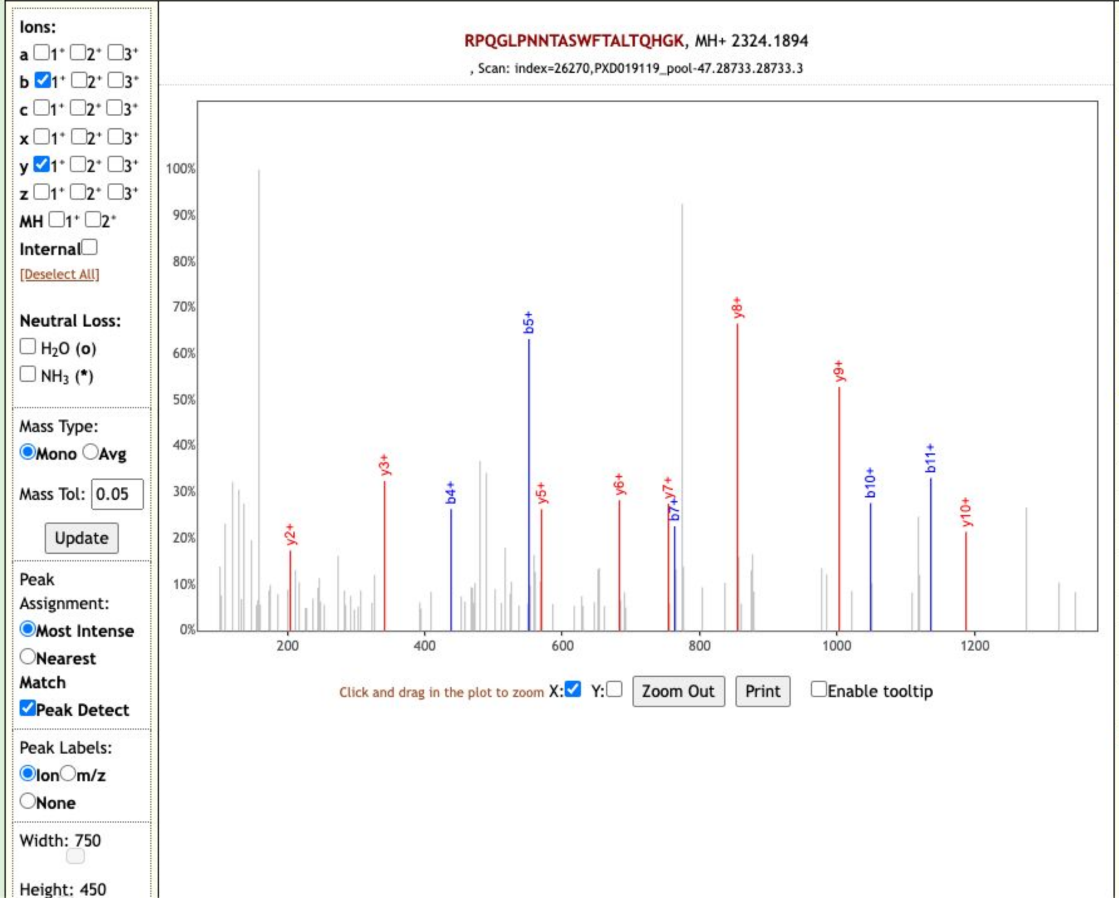

b+	#	Seq	#	y+
157.1084	1	R	21	
254.1612	2	P	20	2168.0883
382.2197	3	Q	19	2071.0356
439.2412	4	G	18	1942.9770
552.3253	5	L	17	1885.9555
649.3780	6	P	16	1772.8715
763.4209	7	N	15	1675.8187
877.4639	8	N	14	1561.7758
978.5116	9	T	13	1447.7328
1049.5487	10	A	12	1346.6852
1136.5807	11	S	11	1275.6480
1322.6600	12	W	10	1188.6160
1469.7284	13	F	9	1002.5367
1570.7761	14	T	8	855.4683
1641.8132	15	A	7	754.4206
1754.8973	16	L	6	683.3835
1855.9450	17	T	5	570.2994
1984.0035	18	Q	4	469.2518
2121.0624	19	H	3	341.1932
2178.0839	20	G	2	204.1343
	21	K	1	147.1128

[Click] to move table

RPQGLPNNTASWFTALTQHGKEDLK

Ions:
a ☐1+ ☐2+ ☐3+
b ☑1+ ☐2+ ☐3+
c ☐1+ ☐2+ ☐3+
x ☐1+ ☐2+ ☐3+
y ☑1+ ☐2+ ☐3+
z ☐1+ ☐2+ ☐3+
MH ☐1+ ☐2+
Internal ☐
[Deselect All]

Neutral Loss:
☐ H_2O (o)
☐ NH_3 (*)

Mass Type:
◉Mono ○Avg

Mass Tol: 0.05

Update

Peak Assignment:
◉Most Intense
○Nearest Match
☑Peak Detect

Peak Labels:
◉Ion ○m/z
○None

Width: 750

Height: 450

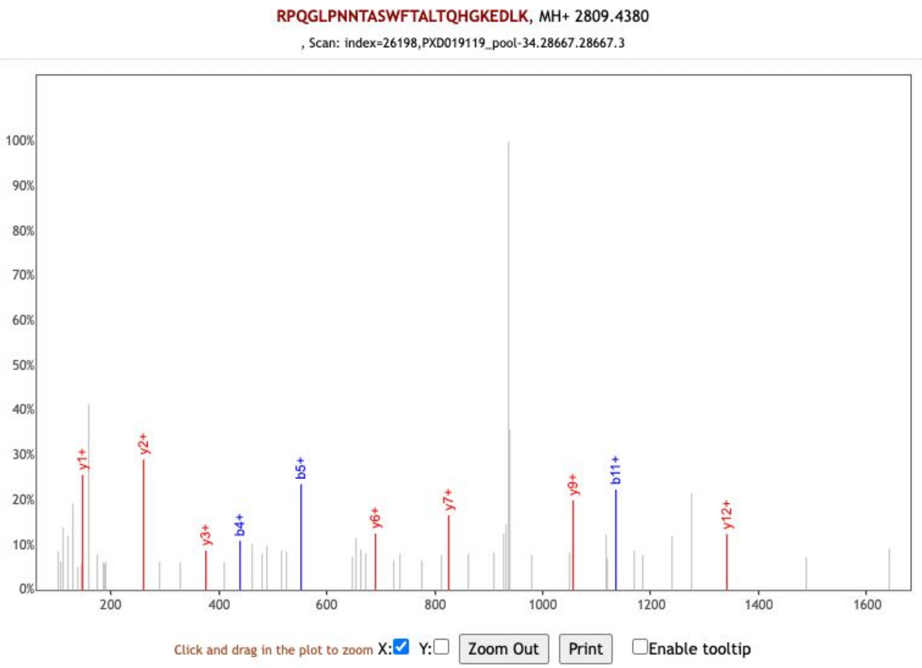

b+	#	Seq	#	y+
157.1084	1	R	25	
254.1612	2	P	24	2653.3369
382.2197	3	Q	23	2556.2841
439.2412	4	G	22	2428.2255
552.3253	5	L	21	2371.2041
649.3780	6	P	20	2258.1200
763.4209	7	N	19	2161.0673
877.4639	8	N	18	2047.0243
978.5116	9	T	17	1932.9814
1049.5487	10	A	16	1831.9337
1136.5807	11	S	15	1760.8966
1322.6600	12	W	14	1673.8646
1469.7284	13	F	13	1487.7853
1570.7761	14	T	12	1340.7169
1641.8132	15	A	11	1239.6692
1754.8973	16	L	10	1168.6321
1855.9450	17	T	9	1055.5480
1984.0035	18	Q	8	954.5003
2121.0624	19	H	7	826.4417
2178.0839	20	G	6	689.3828
2306.1789	21	K	5	632.3614
2435.2215	22	E	4	504.2664
2550.2484	23	D	3	375.2238
2663.3325	24	L	2	260.1969
	25	K	1	147.1128

[Click] to move table

QQTVTLLPAADLDDFSK

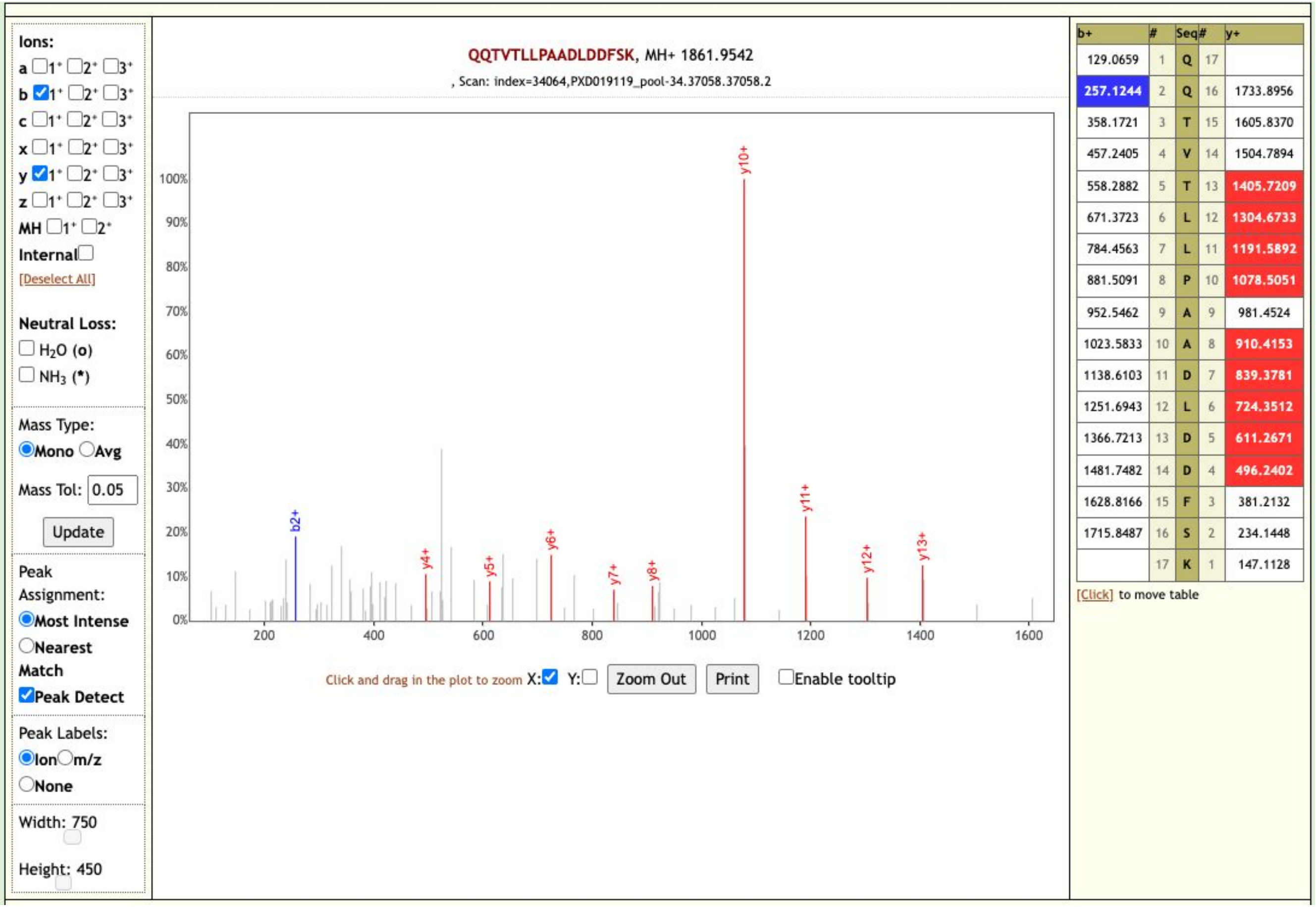

b+	#	Seq	#	y+
129.0659	1	Q	17	
257.1244	2	Q	16	1733.8956
358.1721	3	T	15	1605.8370
457.2405	4	V	14	1504.7894
558.2882	5	T	13	1405.7209
671.3723	6	L	12	1304.6733
784.4563	7	L	11	1191.5892
881.5091	8	P	10	1078.5051
952.5462	9	A	9	981.4524
1023.5833	10	A	8	910.4153
1138.6103	11	D	7	839.3781
1251.6943	12	L	6	724.3512
1366.7213	13	D	5	611.2671
1481.7482	14	D	4	496.2402
1628.8166	15	F	3	381.2132
1715.8487	16	S	2	234.1448
	17	K	1	147.1128

MAGNGGDAALALLLLDR

Ions:
a 1+ 2+ 3+
b 1+ 2+ 3+
c 1+ 2+ 3+
x 1+ 2+ 3+
y 1+ 2+ 3+
z 1+ 2+ 3+
MH 1+ 2+
Internal
[Deselect All]

Neutral Loss:
H_2O (o)
NH_3 (*)

Mass Type:
Mono Avg

Mass Tol: 0.05

Update

Peak Assignment:
Most Intense
Nearest Match
Peak Detect

Peak Labels:
Ion m/z
None

Width: 750

Height: 450

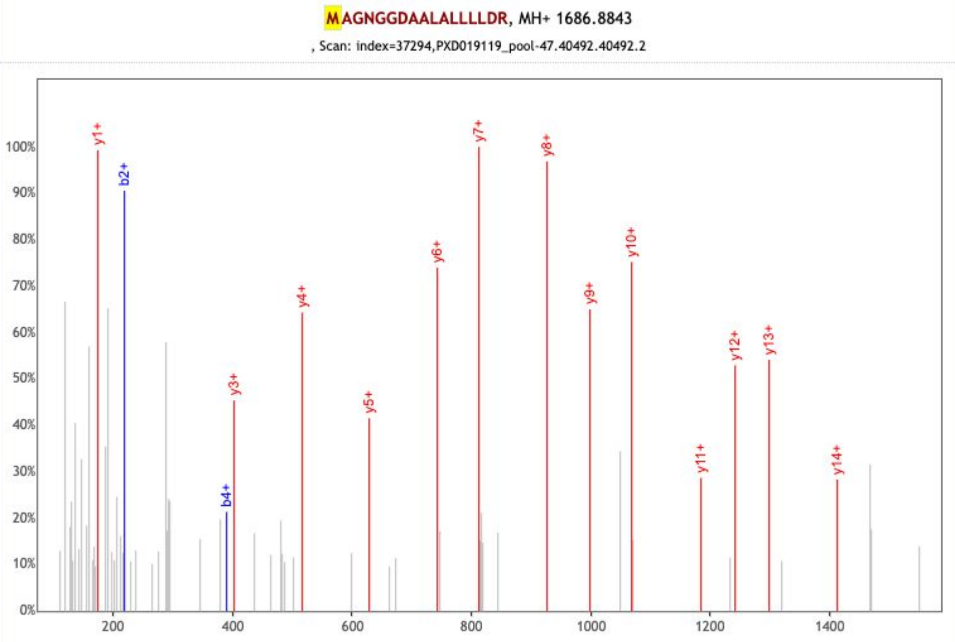

Click and drag in the plot to zoom X: Y: Zoom Out Print Enable tooltip

b+	#	Seq	#	y+
148.0427	1	M	17	
219.0798	2	A	16	1539.8489
276.1013	3	G	15	1468.8118
390.1442	4	N	14	1411.7904
447.1656	5	G	13	1297.7474
504.1871	6	G	12	1240.7260
619.2141	7	D	11	1183.7045
690.2512	8	A	10	1068.6776
761.2883	9	A	9	997.6404
874.3723	10	L	8	926.6033
945.4095	11	A	7	813.5193
1058.4935	12	L	6	742.4822
1171.5776	13	L	5	629.3981
1284.6616	14	L	4	516.3140
1397.7457	15	L	3	403.2300
1512.7727	16	D	2	290.1459
	17	R	1	175.1190

[Click] to move table

Variable Modifications:
M: 15.994915 [1]

MAGNGGDAALALLLLDRLNQLESK

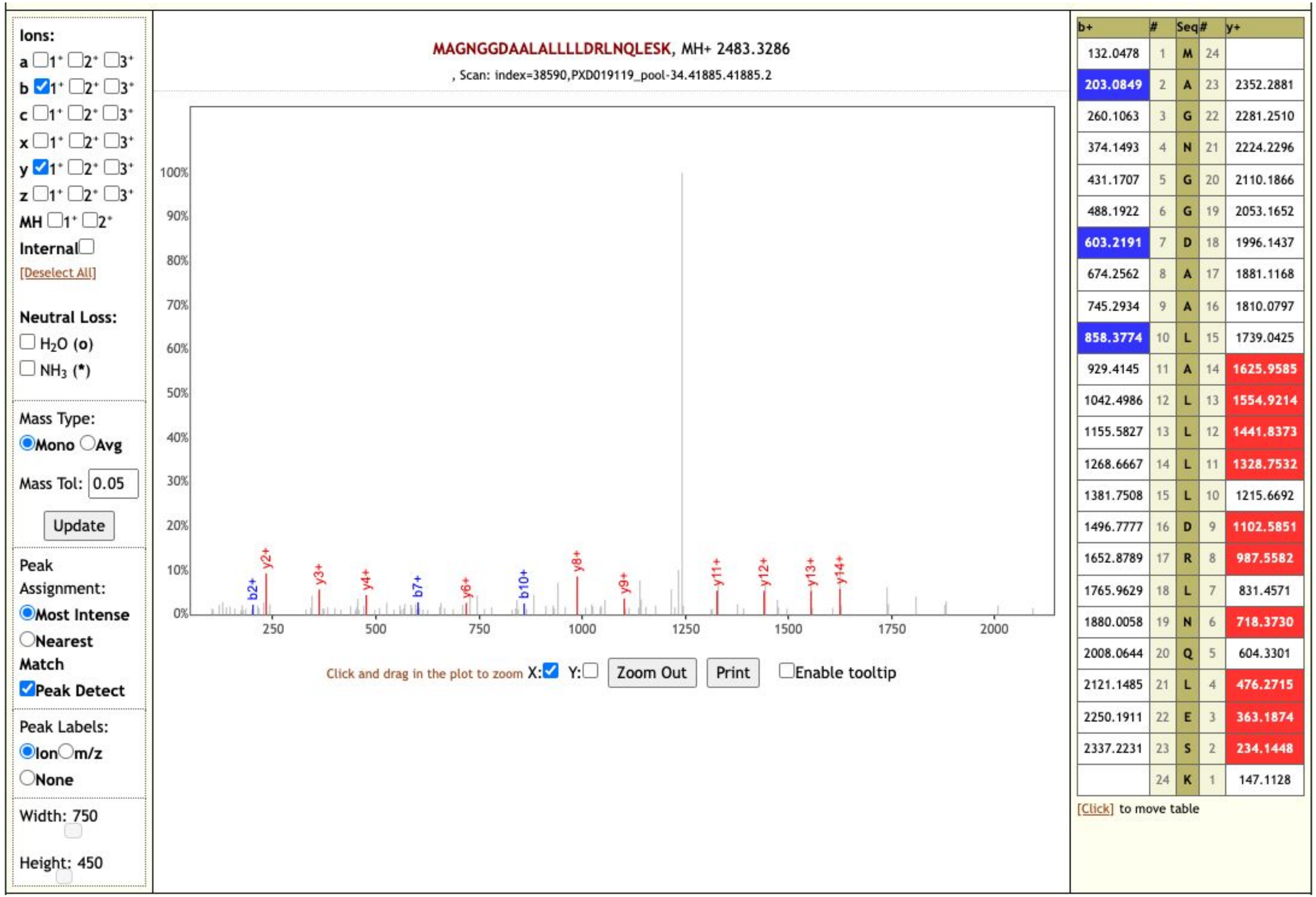

b+	#	Seq	#	y+
132.0478	1	M	24	
203.0849	2	A	23	2352.2881
260.1063	3	G	22	2281.2510
374.1493	4	N	21	2224.2296
431.1707	5	G	20	2110.1866
488.1922	6	G	19	2053.1652
603.2191	7	D	18	1996.1437
674.2562	8	A	17	1881.1168
745.2934	9	A	16	1810.0797
858.3774	10	L	15	1739.0425
929.4145	11	A	14	1625.9585
1042.4986	12	L	13	1554.9214
1155.5827	13	L	12	1441.8373
1268.6667	14	L	11	1328.7532
1381.7508	15	L	10	1215.6692
1496.7777	16	D	9	1102.5851
1652.8789	17	R	8	987.5582
1765.9629	18	L	7	831.4571
1880.0058	19	N	6	718.3730
2008.0644	20	Q	5	604.3301
2121.1485	21	L	4	476.2715
2250.1911	22	E	3	363.1874
2337.2231	23	S	2	234.1448
	24	K	1	147.1128

[Click] to move table

RGPEQTQGNFGDQELTR

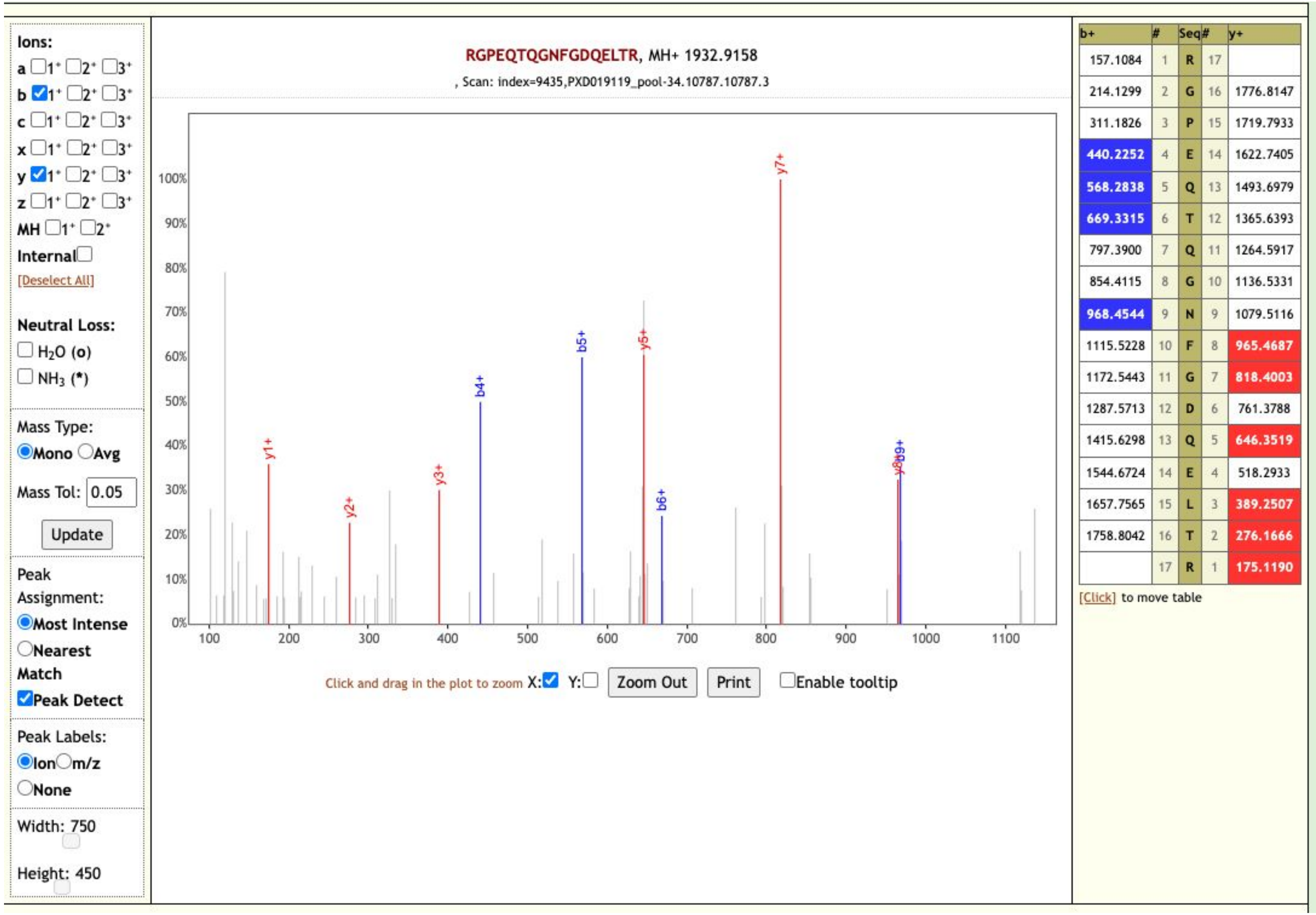

b+	#	Seq	#	y+
157.1084	1	R	17	
214.1299	2	G	16	1776.8147
311.1826	3	P	15	1719.7933
440.2252	4	E	14	1622.7405
568.2838	5	Q	13	1493.6979
669.3315	6	T	12	1365.6393
797.3900	7	Q	11	1264.5917
854.4115	8	G	10	1136.5331
968.4544	9	N	9	1079.5116
1115.5228	10	F	8	965.4687
1172.5443	11	G	7	818.4003
1287.5713	12	D	6	761.3788
1415.6298	13	Q	5	646.3519
1544.6724	14	E	4	518.2933
1657.7565	15	L	3	389.2507
1758.8042	16	T	2	276.1666
	17	R	1	175.1190

RPQGLPNNTASW

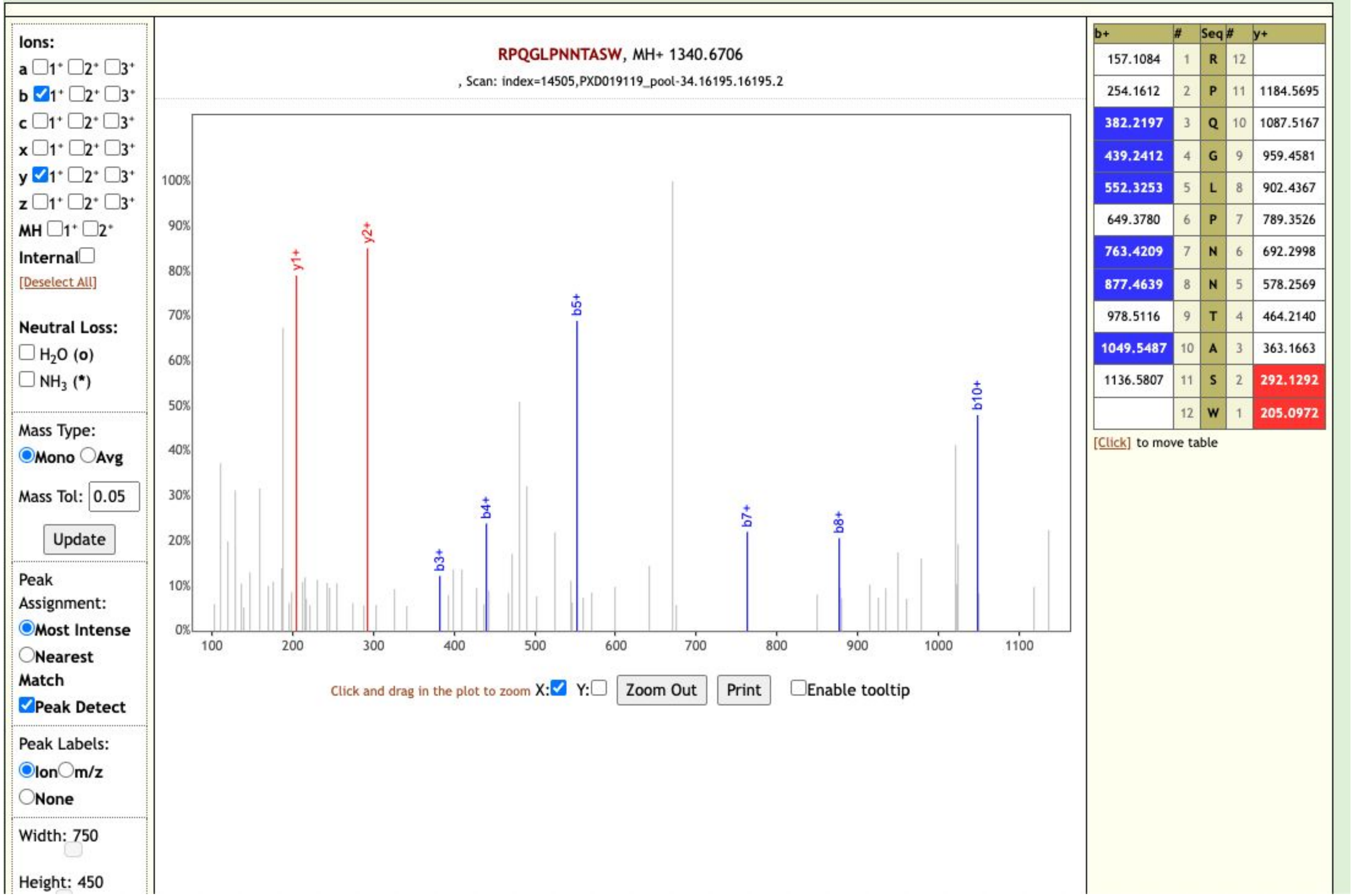

YLGTGPEAGLPYGANK

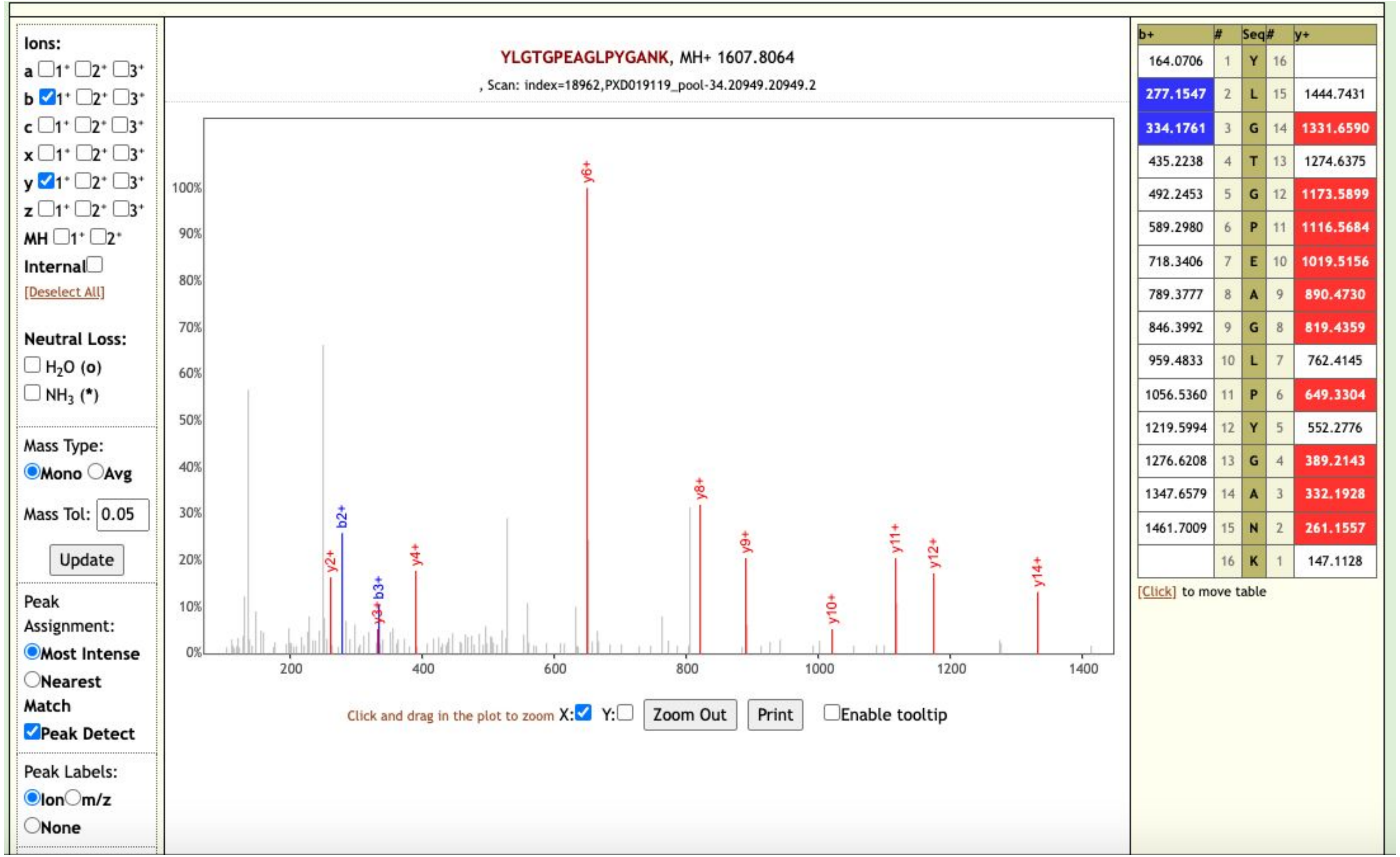

b+	#	Seq	#	y+
164.0706	1	Y	16	
277.1547	2	L	15	1444.7431
334.1761	3	G	14	1331.6590
435.2238	4	T	13	1274.6375
492.2453	5	G	12	1173.5899
589.2980	6	P	11	1116.5684
718.3406	7	E	10	1019.5156
789.3777	8	A	9	890.4730
846.3992	9	G	8	819.4359
959.4833	10	L	7	762.4145
1056.5360	11	P	6	649.3304
1219.5994	12	Y	5	552.2776
1276.6208	13	G	4	389.2143
1347.6579	14	A	3	332.1928
1461.7009	15	N	2	261.1557
	16	K	1	147.1128

[Click] to move table

TALTQHGKEDLK

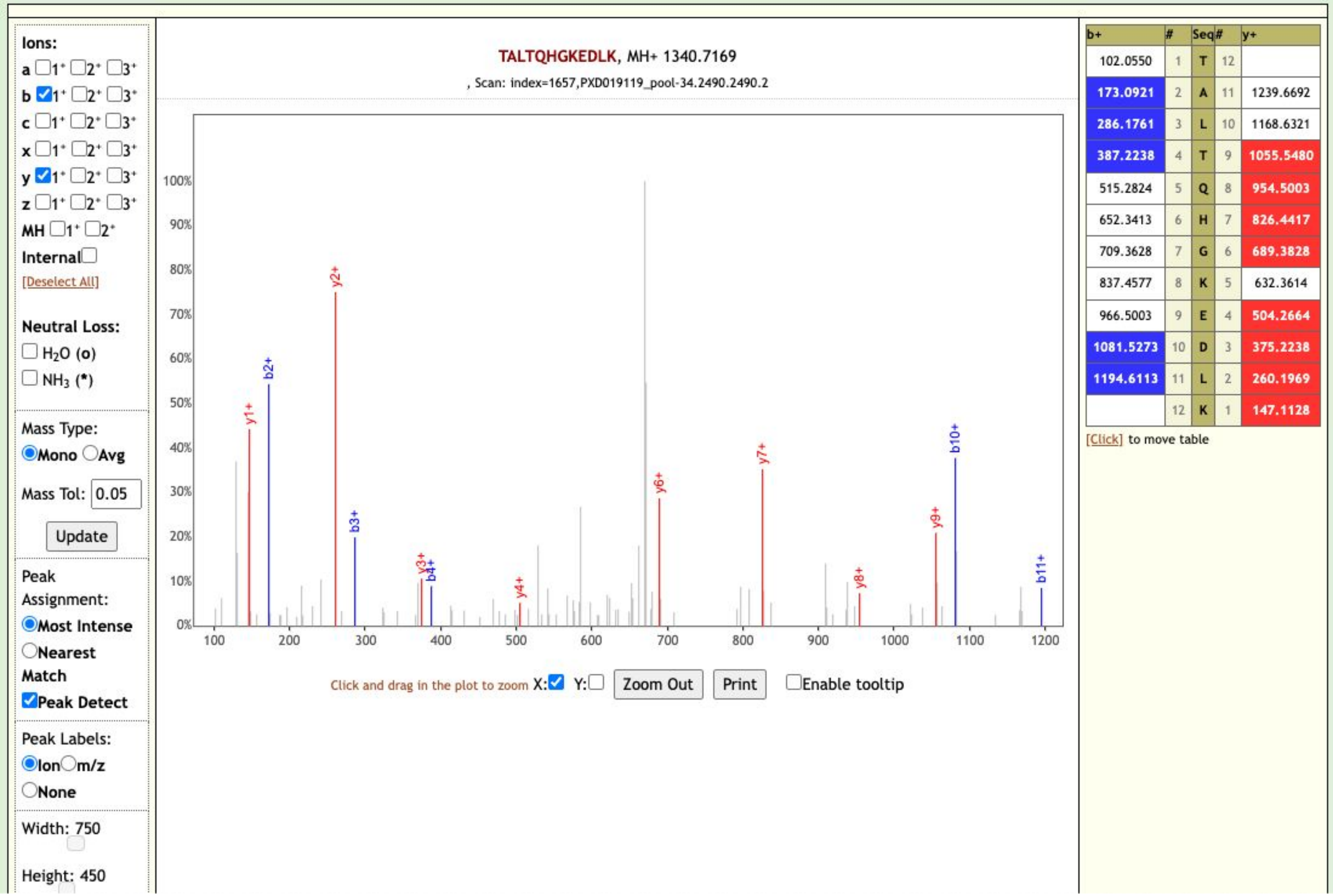

b+	#	Seq	#	y+
102.0550	1	T	12	
173.0921	2	A	11	1239.6692
286.1761	3	L	10	1168.6321
387.2238	4	T	9	1055.5480
515.2824	5	Q	8	954.5003
652.3413	6	H	7	826.4417
709.3628	7	G	6	689.3828
837.4577	8	K	5	632.3614
966.5003	9	E	4	504.2664
1081.5273	10	D	3	375.2238
1194.6113	11	L	2	260.1969
	12	K	1	147.1128

TALTQHGKEDLK

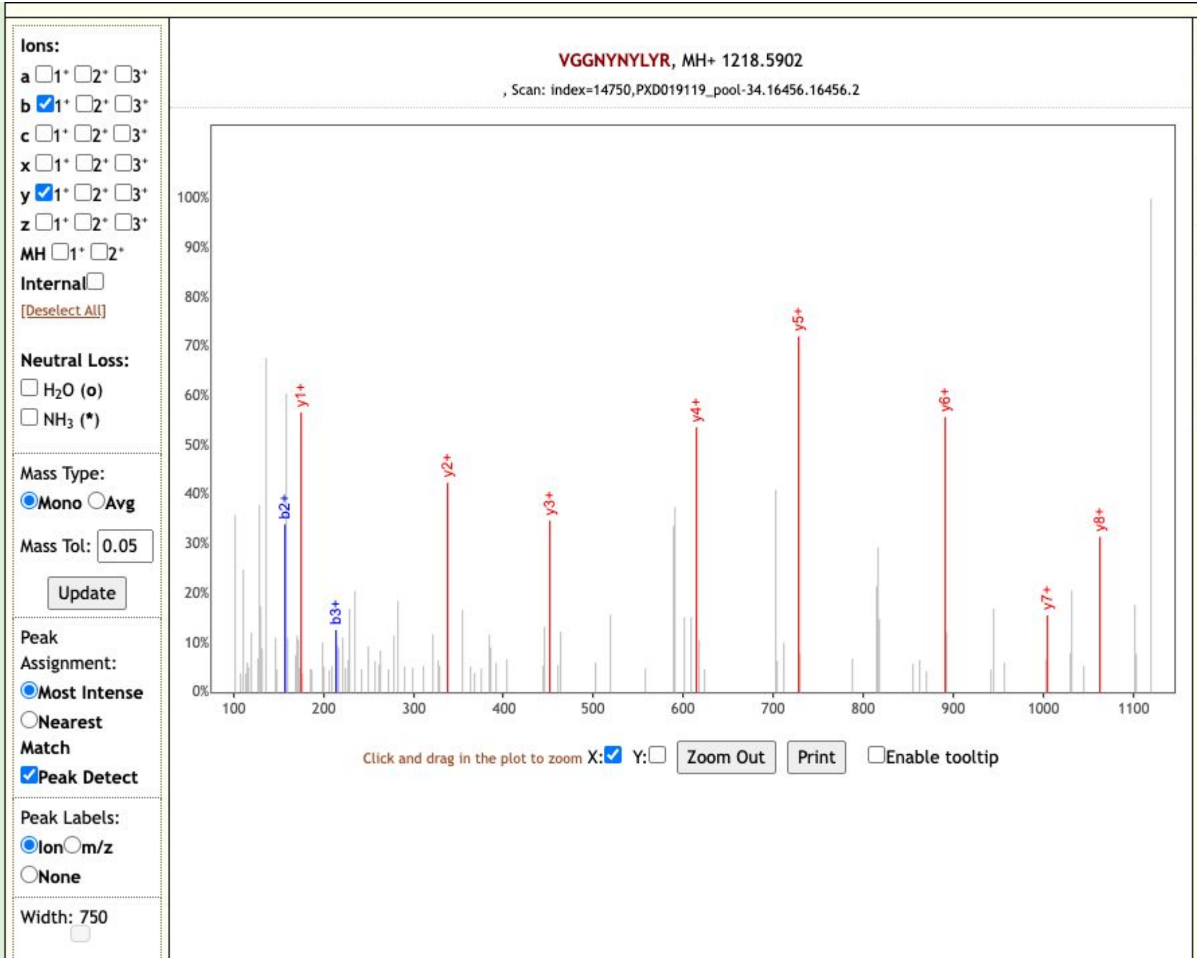

b+	#	Seq	#	y+
100.0757	1	V	10	
157.0972	2	G	9	1119.5218
214.1186	3	G	8	1062.5003
328.1615	4	N	7	1005.4789
491.2249	5	Y	6	891.4359
605.2678	6	N	5	728.3726
768.3311	7	Y	4	614.3297
881.4152	8	L	3	451.2663
1044.4785	9	Y	2	338.1823
	10	R	1	175.1190

PAADLDDFSK

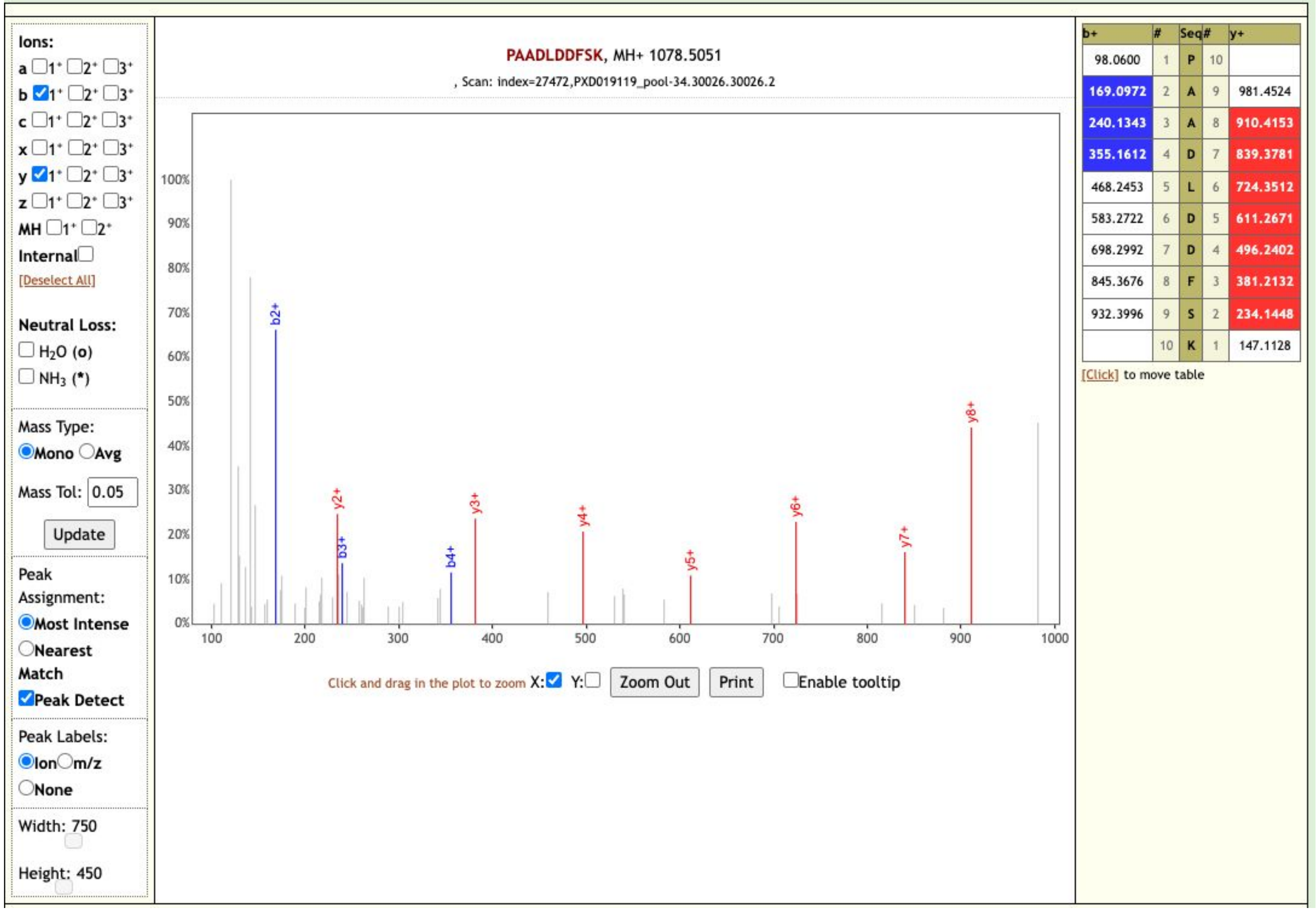

b+	#	Seq	#	y+
98.0600	1	P	10	
169.0972	2	A	9	981.4524
240.1343	3	A	8	910.4153
355.1612	4	D	7	839.3781
468.2453	5	L	6	724.3512
583.2722	6	D	5	611.2671
698.2992	7	D	4	496.2402
845.3676	8	F	3	381.2132
932.3996	9	S	2	234.1448
	10	K	1	147.1128

VATEGALNTPK

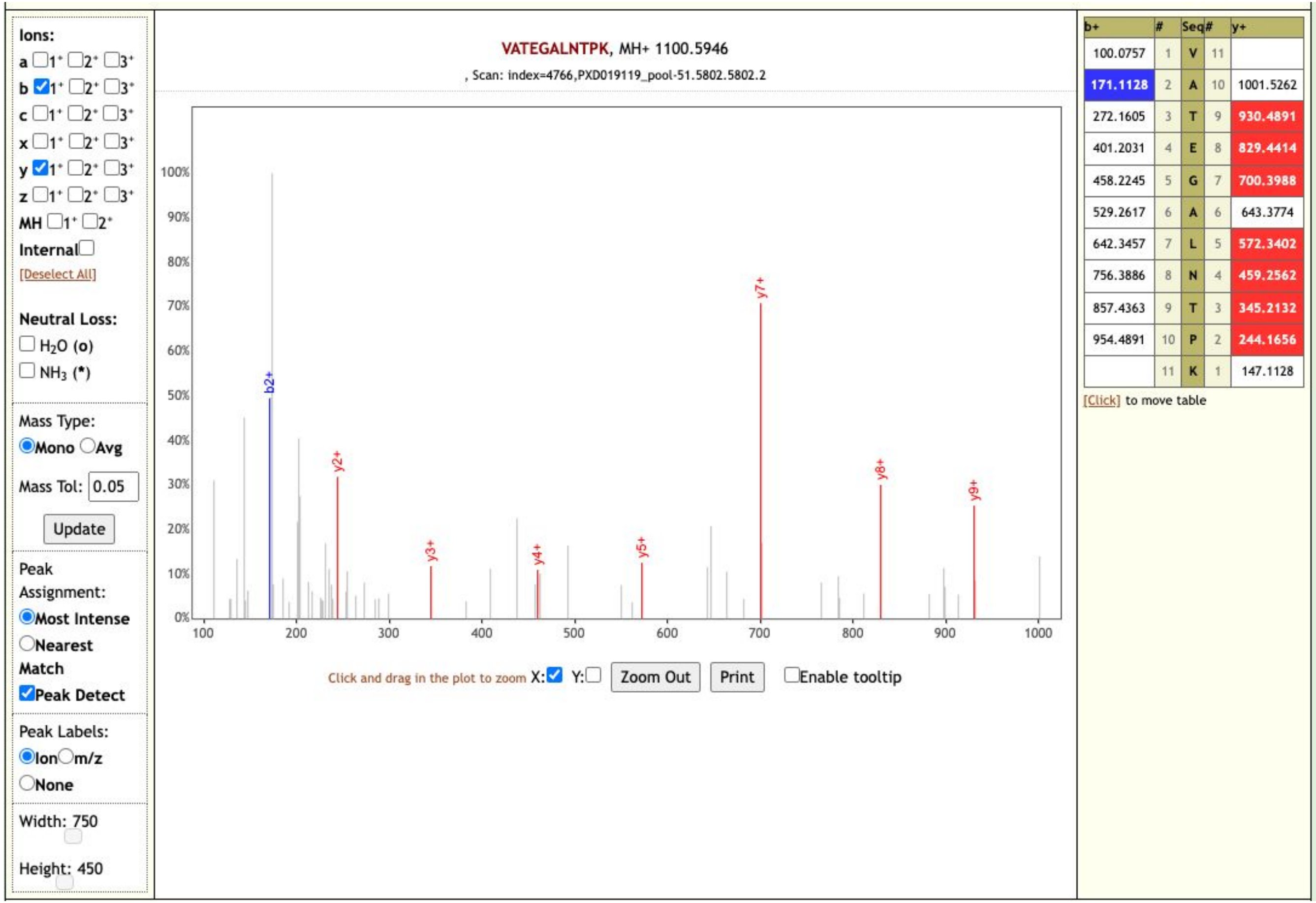

WYFYYLGTGPEAGLPYGANK

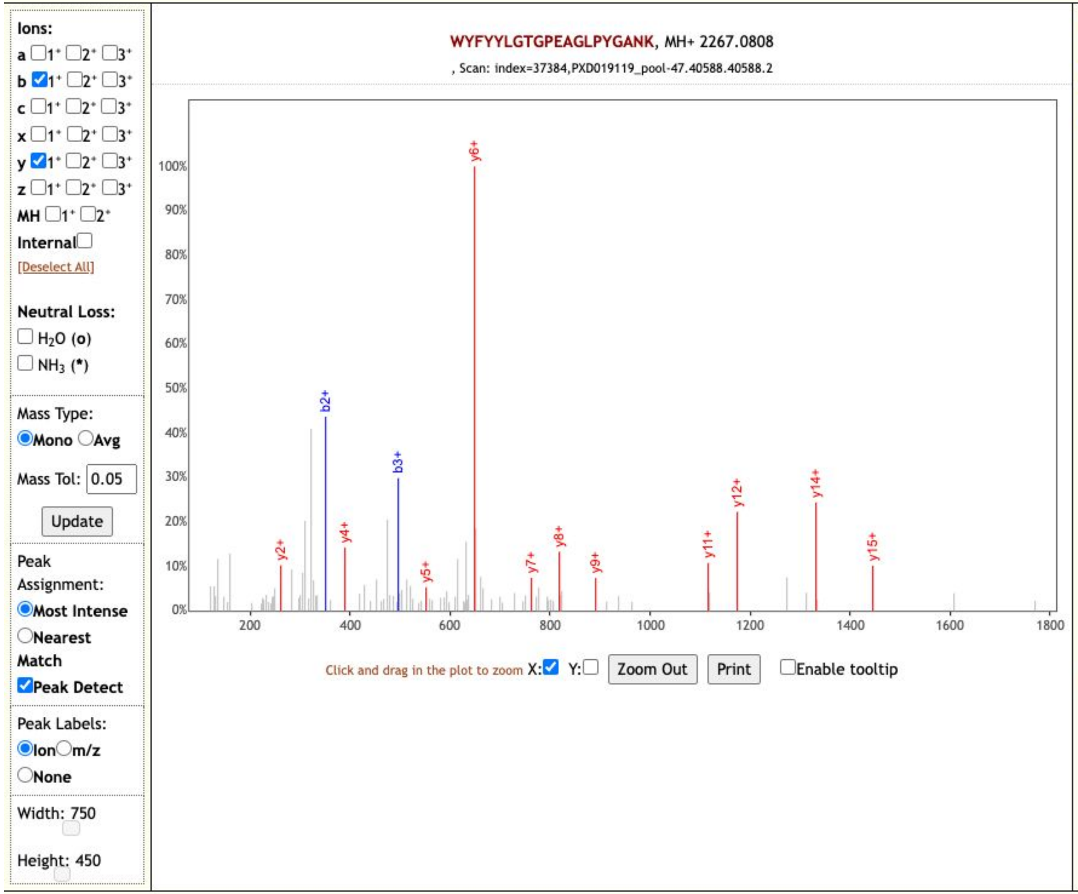

b+	#	Seq	#	y+
187.0866	1	W	20	
350.1499	2	Y	19	2081.0015
497.2183	3	F	18	1917.9381
660.2817	4	Y	17	1770.8697
823.3450	5	Y	16	1607.8064
936.4291	6	L	15	1444.7431
993.4505	7	G	14	1331.6590
1094.4982	8	T	13	1274.6375
1151.5197	9	G	12	1173.5899
1248.5724	10	P	11	1116.5684
1377.6150	11	E	10	1019.5156
1448.6521	12	A	9	890.4730
1505.6736	13	G	8	819.4359
1618.7577	14	L	7	762.4145
1715.8104	15	P	6	649.3304
1878.8737	16	Y	5	552.2776
1935.8952	17	G	4	389.2143
2006.9323	18	A	3	332.1928
2120.9753	19	N	2	261.1557
	20	K	1	147.1128

[Click] to move table

VAGDSGFAAYSR

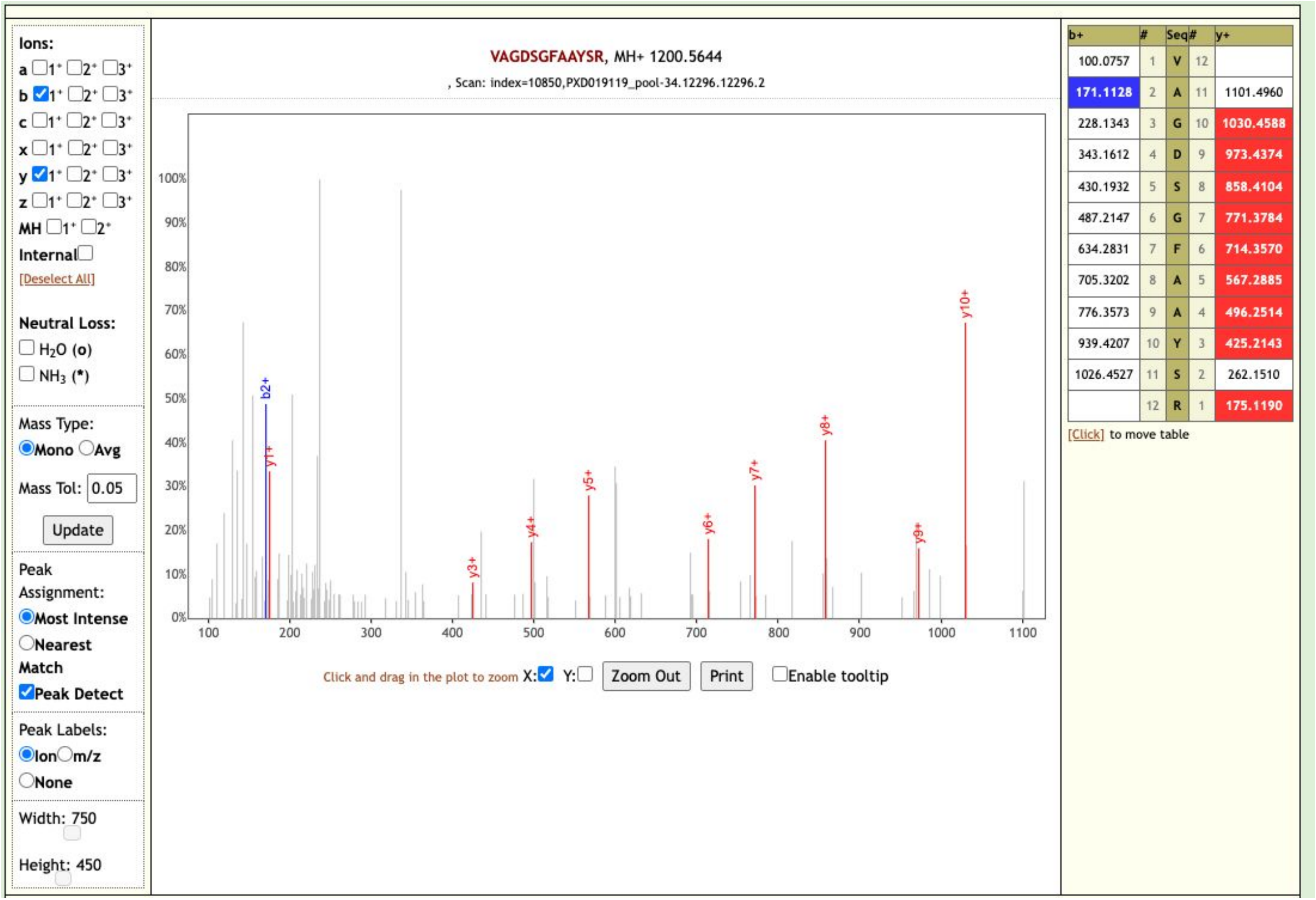

b+	#	Seq	#	y+
100.0757	1	V	12	
171.1128	2	A	11	1101.4960
228.1343	3	G	10	1030.4588
343.1612	4	D	9	973.4374
430.1932	5	S	8	858.4104
487.2147	6	G	7	771.3784
634.2831	7	F	6	714.3570
705.3202	8	A	5	567.2885
776.3573	9	A	4	496.2514
939.4207	10	Y	3	425.2143
1026.4527	11	S	2	262.1510
	12	R	1	175.1190

YYLGTGPEAGLPYGANK

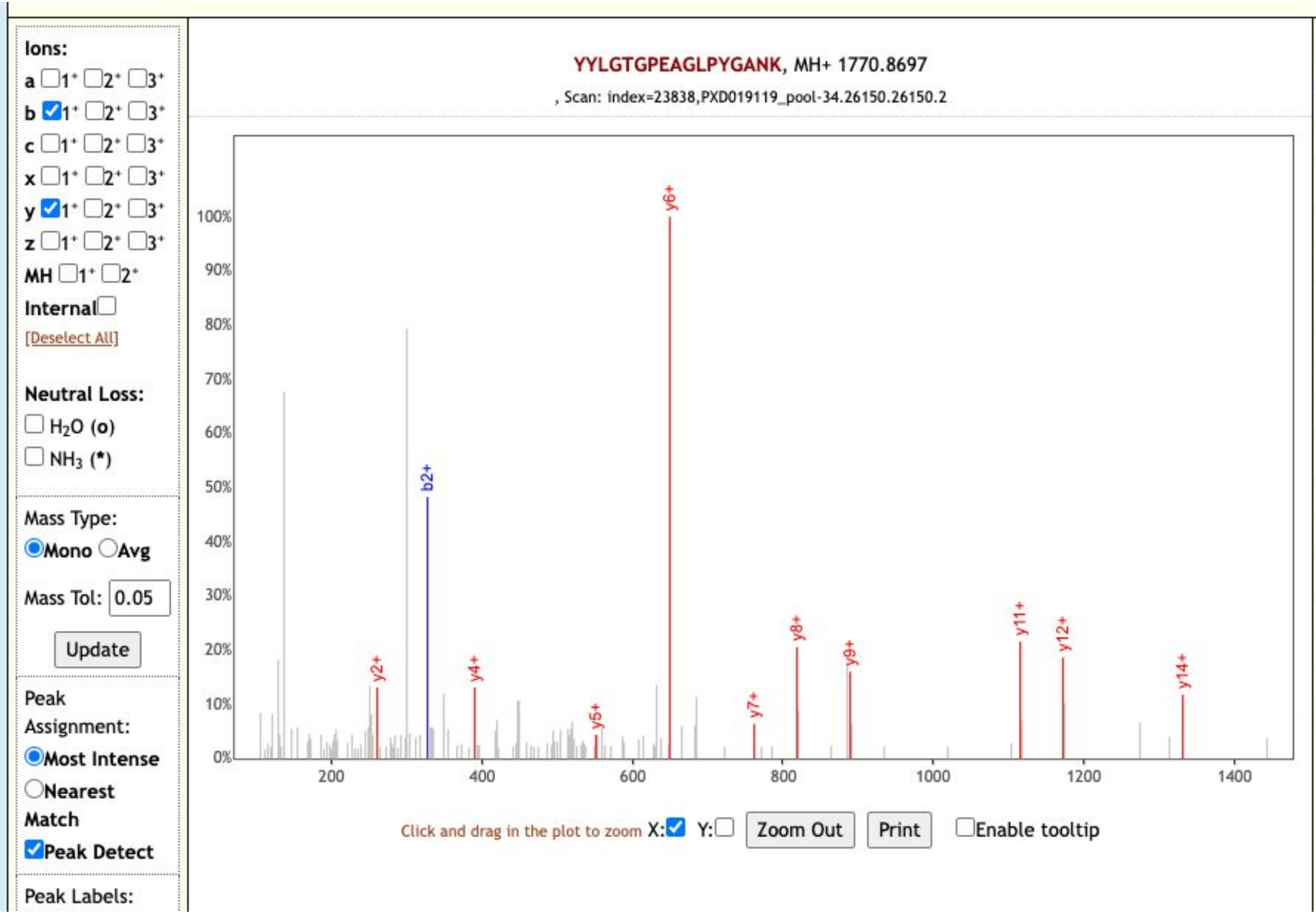

b+	#	Seq	#	y+
164.0706	1	Y	17	
327.1339	2	Y	16	1607.8064
440.2180	3	L	15	1444.7431
497.2395	4	G	14	1331.6590
598.2871	5	T	13	1274.6375
655.3086	6	G	12	1173.5899
752.3614	7	P	11	1116.5684
881.4040	8	E	10	1019.5156
952.4411	9	A	9	890.4730
1009.4625	10	G	8	819.4359
1122.5466	11	L	7	762.4145
1219.5994	12	P	6	649.3304
1382.6627	13	Y	5	552.2776
1439.6842	14	G	4	389.2143
1510.7213	15	A	3	332.1928
1624.7642	16	N	2	261.1557
	17	K	1	147.1128

[Click] to move table

SFIEDLLFNK

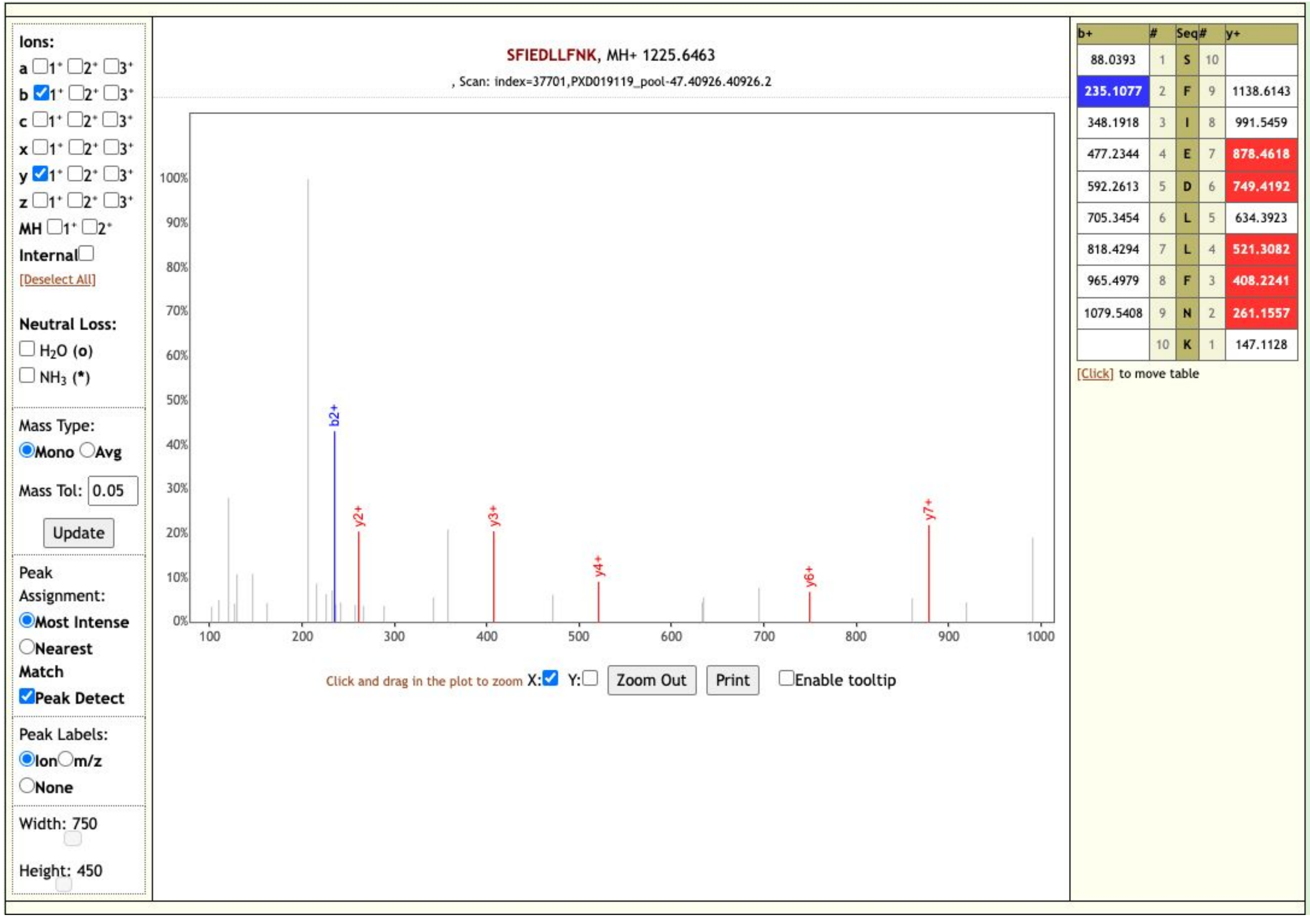

b+	#	Seq	#	y+
88.0393	1	S	10	
235.1077	2	F	9	1138.6143
348.1918	3	I	8	991.5459
477.2344	4	E	7	878.4618
592.2613	5	D	6	749.4192
705.3454	6	L	5	634.3923
818.4294	7	L	4	521.3082
965.4979	8	F	3	408.2241
1079.5408	9	N	2	261.1557
	10	K	1	147.1128

SMGTSPTRMAGNGGDAALALLLLDR

Ions:
a 1+ 2+ 3+
b 1+ 2+ 3+
c 1+ 2+ 3+
x 1+ 2+ 3+
y 1+ 2+ 3+
z 1+ 2+ 3+
MH 1+ 2+
Internal
[Deselect All]

Neutral Loss:
H_2O (o)
NH_3 (*)

Mass Type:
Mono Avg

Mass Tol: 0.05

Update

Peak Assignment:
Most Intense
Nearest Match
Peak Detect

Peak Labels:
Ion m/z
None

Width: 750

Height: 450

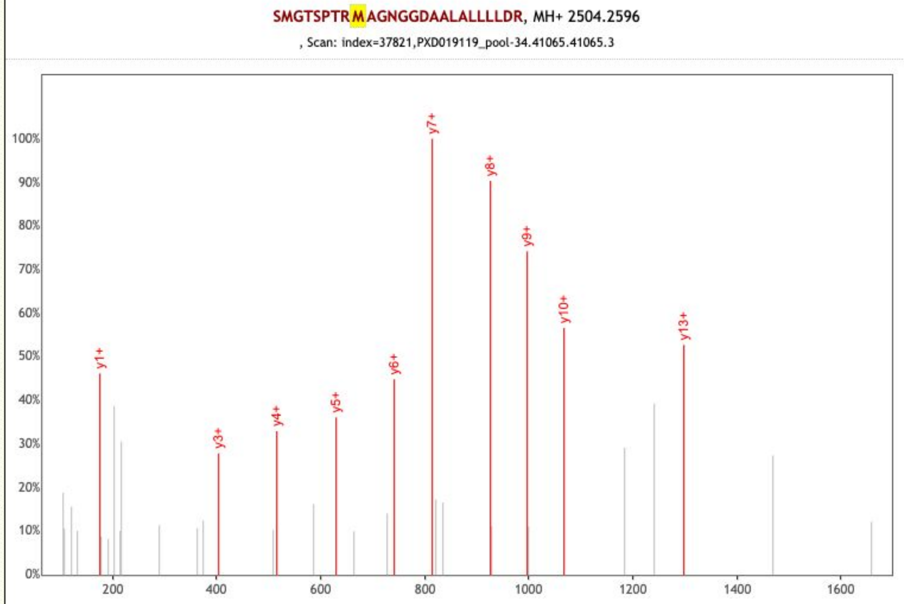

Click and drag in the plot to zoom X: Y: Zoom Out Print Enable tooltip

b+	#	Seq	#	y+
88.0393	1	S	25	
219.0798	2	M	24	2417.2275
276.1013	3	G	23	2286.1871
377.1489	4	T	22	2229.1656
464.1810	5	S	21	2128.1179
561.2337	6	P	20	2041.0859
662.2814	7	T	19	1944.0331
818.3825	8	R	18	1842.9854
965.4179	9	M	17	1686.8843
1036.4550	10	A	16	1539.8489
1093.4765	11	G	15	1468.8118
1207.5194	12	N	14	1411.7904
1264.5409	13	G	13	1297.7474
1321.5623	14	G	12	1240.7260
1436.5893	15	D	11	1183.7045
1507.6264	16	A	10	1068.6776
1578.6635	17	A	9	997.6404
1691.7476	18	L	8	926.6033
1762.7847	19	A	7	813.5193
1875.8688	20	L	6	742.4822
1988.9528	21	L	5	629.3981
2102.0369	22	L	4	516.3140
2215.1209	23	L	3	403.2300
2330.1479	24	D	2	290.1459
	25	R	1	175.1190

[Click] to move table

Variable Modifications:

M: 15.994915 [9]

NPANNAAIVLQLPQGT

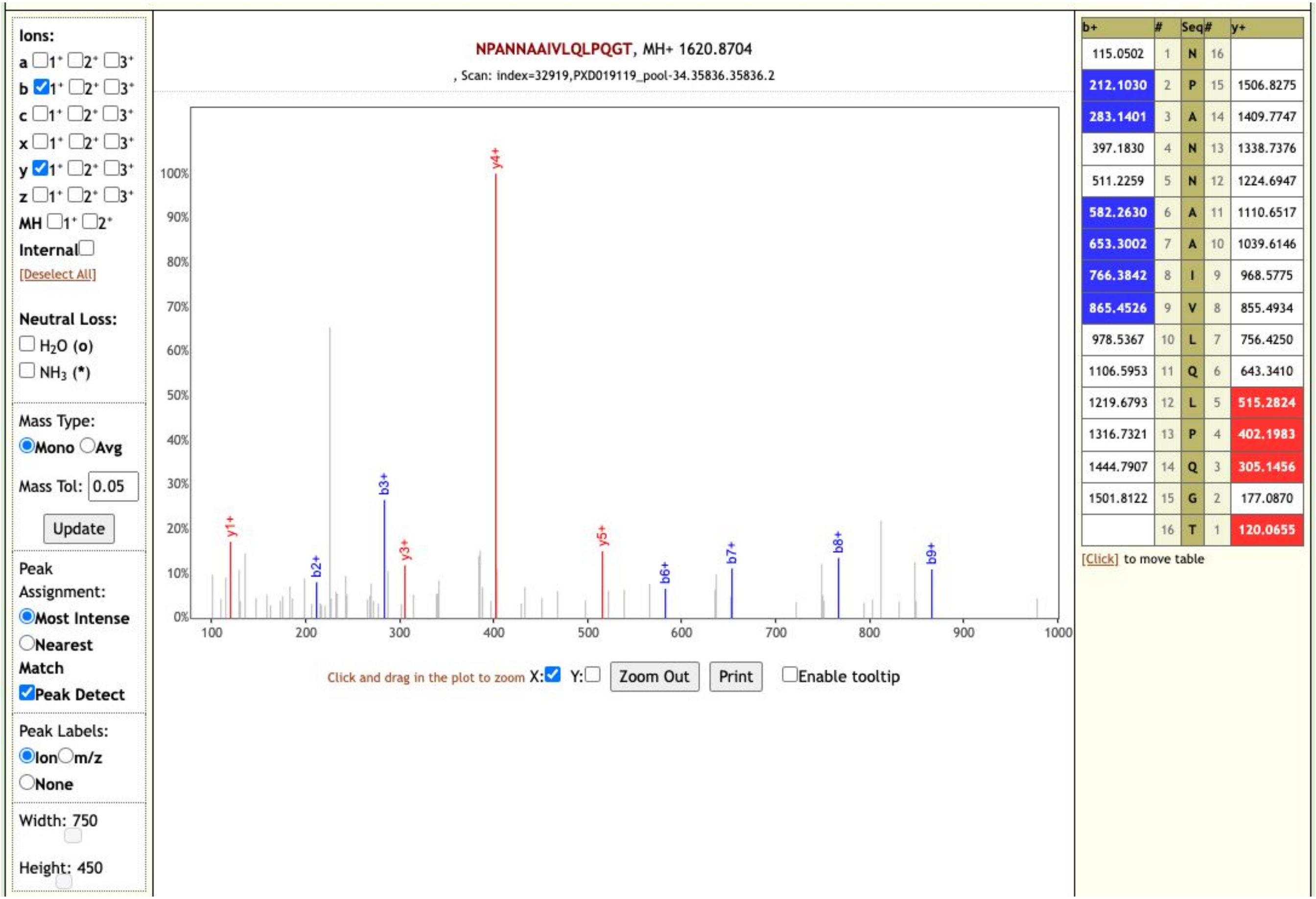

b+	#	Seq	#	y+
115.0502	1	N	16	
212.1030	2	P	15	1506.8275
283.1401	3	A	14	1409.7747
397.1830	4	N	13	1338.7376
511.2259	5	N	12	1224.6947
582.2630	6	A	11	1110.6517
653.3002	7	A	10	1039.6146
766.3842	8	I	9	968.5775
865.4526	9	V	8	855.4934
978.5367	10	L	7	756.4250
1106.5953	11	Q	6	643.3410
1219.6793	12	L	5	515.2824
1316.7321	13	P	4	402.1983
1444.7907	14	Q	3	305.1456
1501.8122	15	G	2	177.0870
	16	T	1	120.0655

[Click] to move table

NTNSSPDDQIGYYR

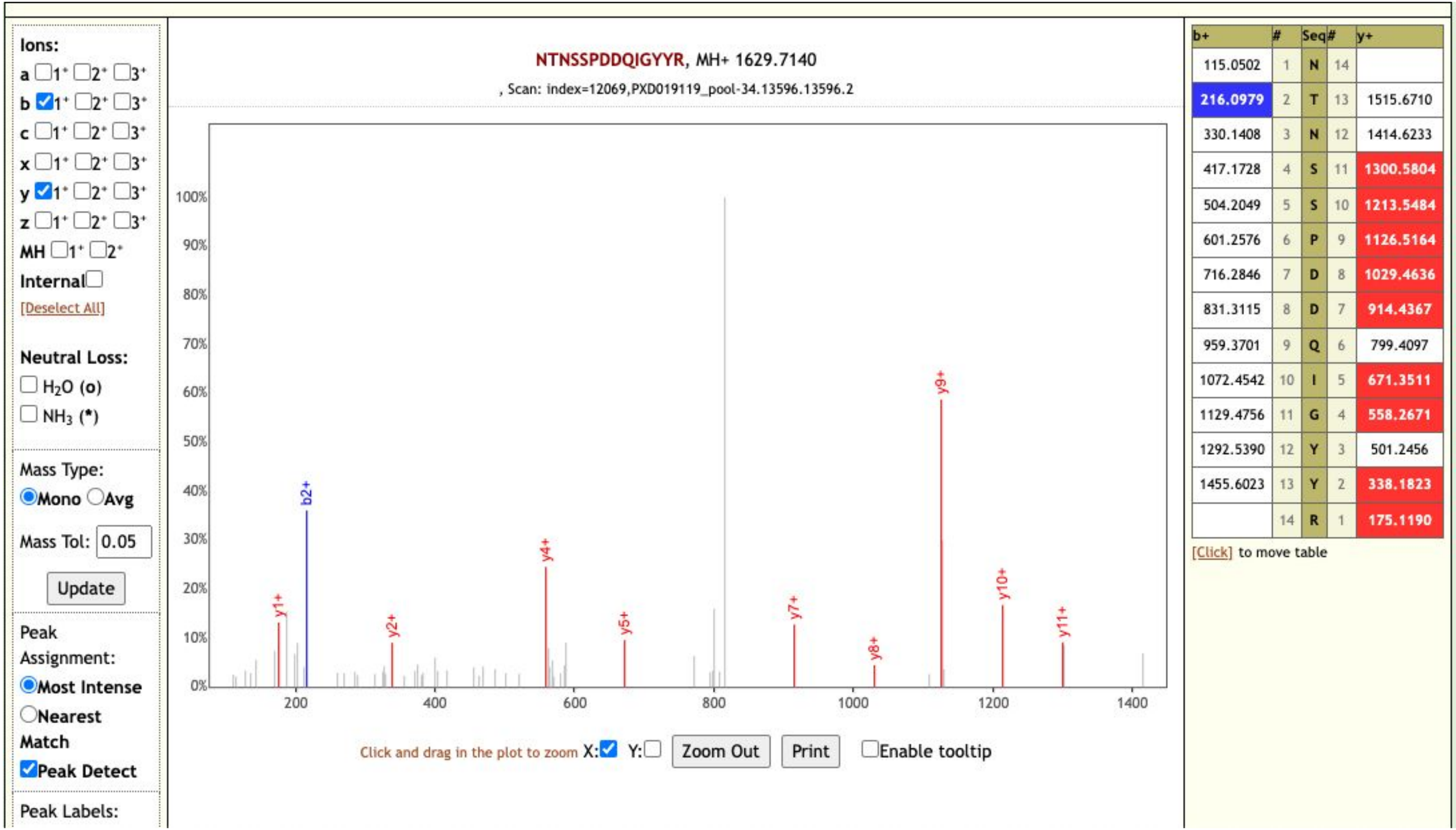

b+	#	Seq	#	y+
115.0502	1	N	14	
216.0979	2	T	13	1515.6710
330.1408	3	N	12	1414.6233
417.1728	4	S	11	1300.5804
504.2049	5	S	10	1213.5484
601.2576	6	P	9	1126.5164
716.2846	7	D	8	1029.4636
831.3115	8	D	7	914.4367
959.3701	9	Q	6	799.4097
1072.4542	10	I	5	671.3511
1129.4756	11	G	4	558.2671
1292.5390	12	Y	3	501.2456
1455.6023	13	Y	2	338.1823
	14	R	1	175.1190

NSSPDDQIGYYR

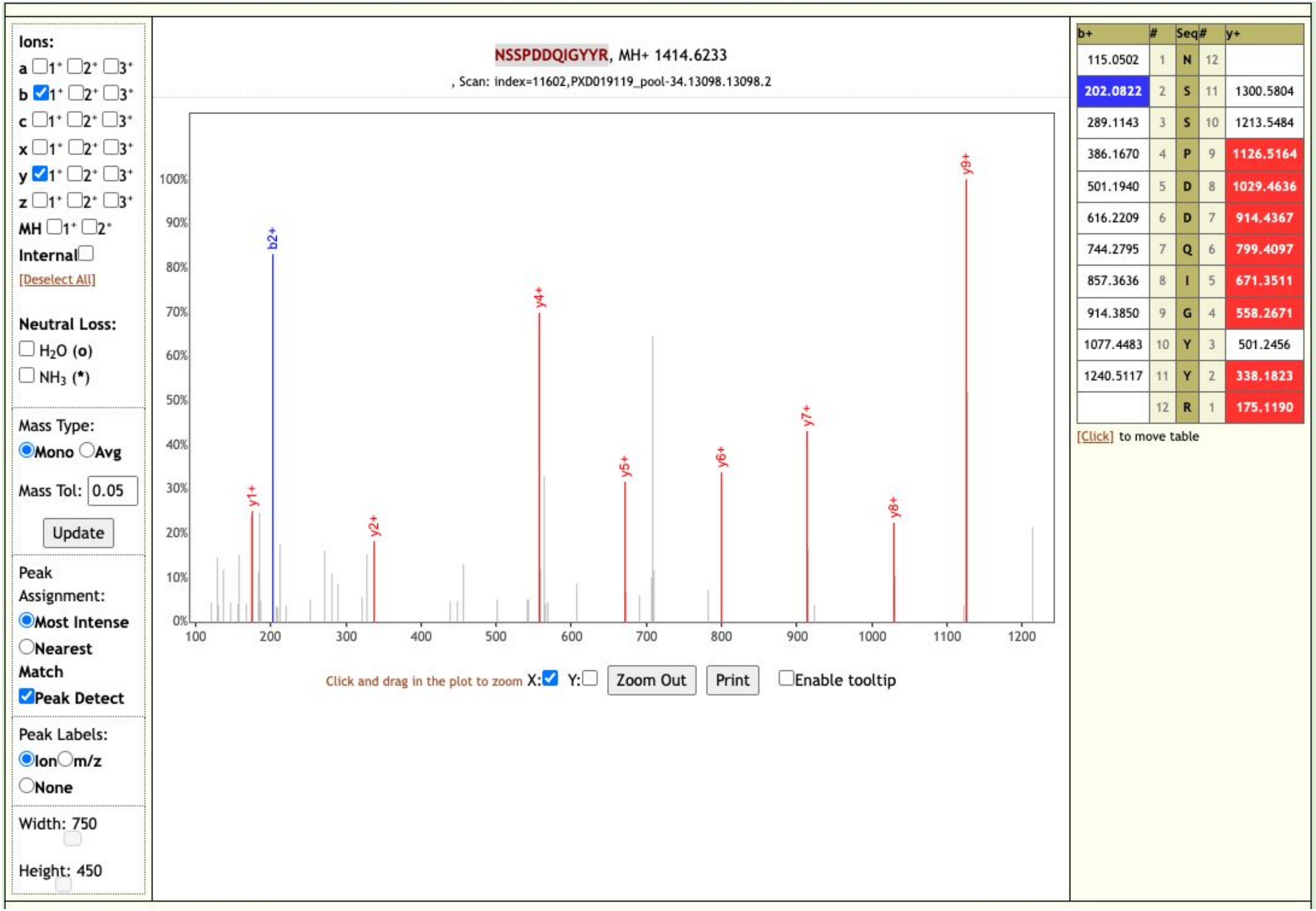

PXD023016

GIIWVATEGALNTPK

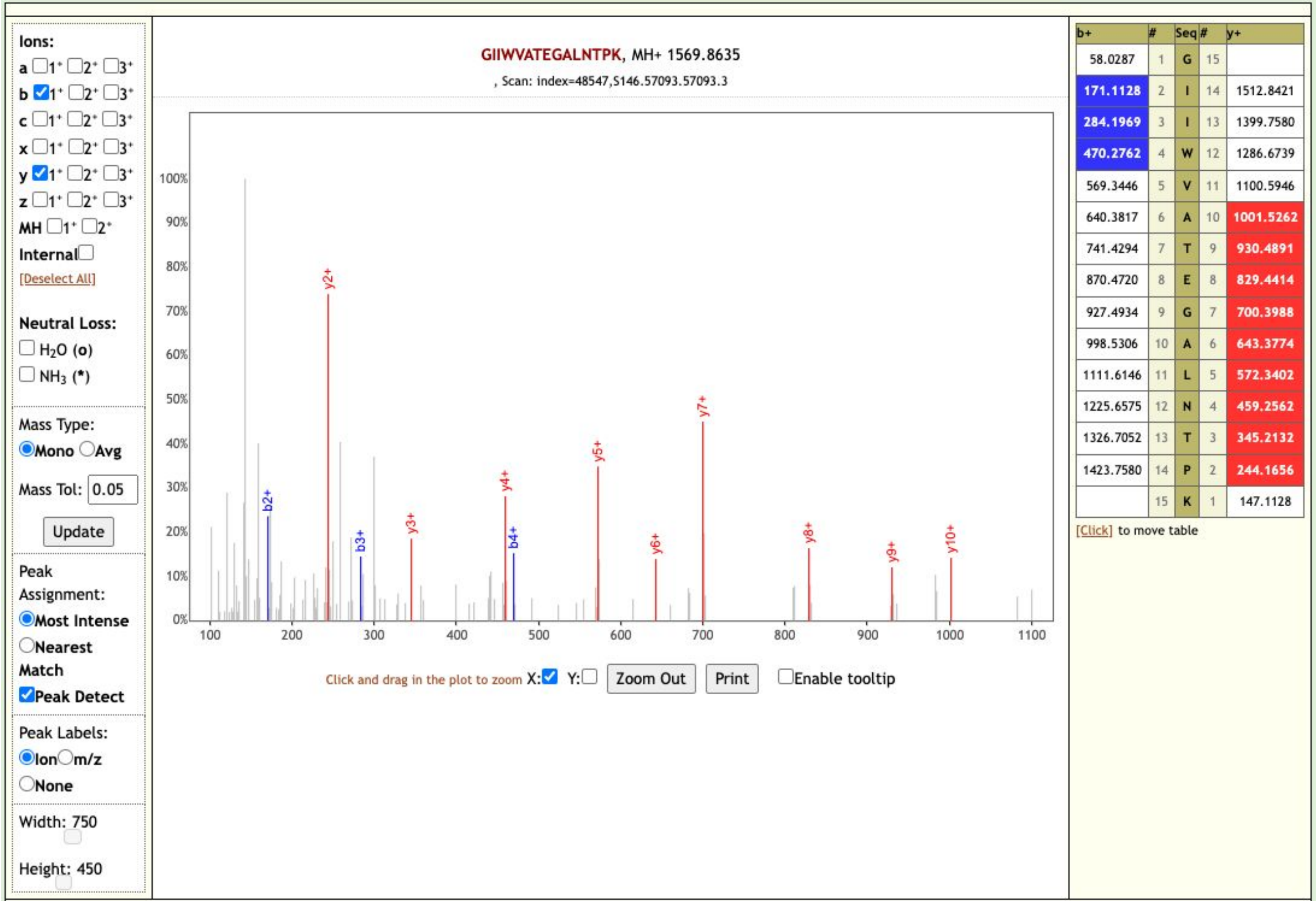

b+	#	Seq	#	y+
58.0287	1	G	15	
171.1128	2	I	14	1512.8421
284.1969	3	I	13	1399.7580
470.2762	4	W	12	1286.6739
569.3446	5	V	11	1100.5946
640.3817	6	A	10	1001.5262
741.4294	7	T	9	930.4891
870.4720	8	E	8	829.4414
927.4934	9	G	7	700.3988
998.5306	10	A	6	643.3774
1111.6146	11	L	5	572.3402
1225.6575	12	N	4	459.2562
1326.7052	13	T	3	345.2132
1423.7580	14	P	2	244.1656
	15	K	1	147.1128

IIWVATEGALNTPK

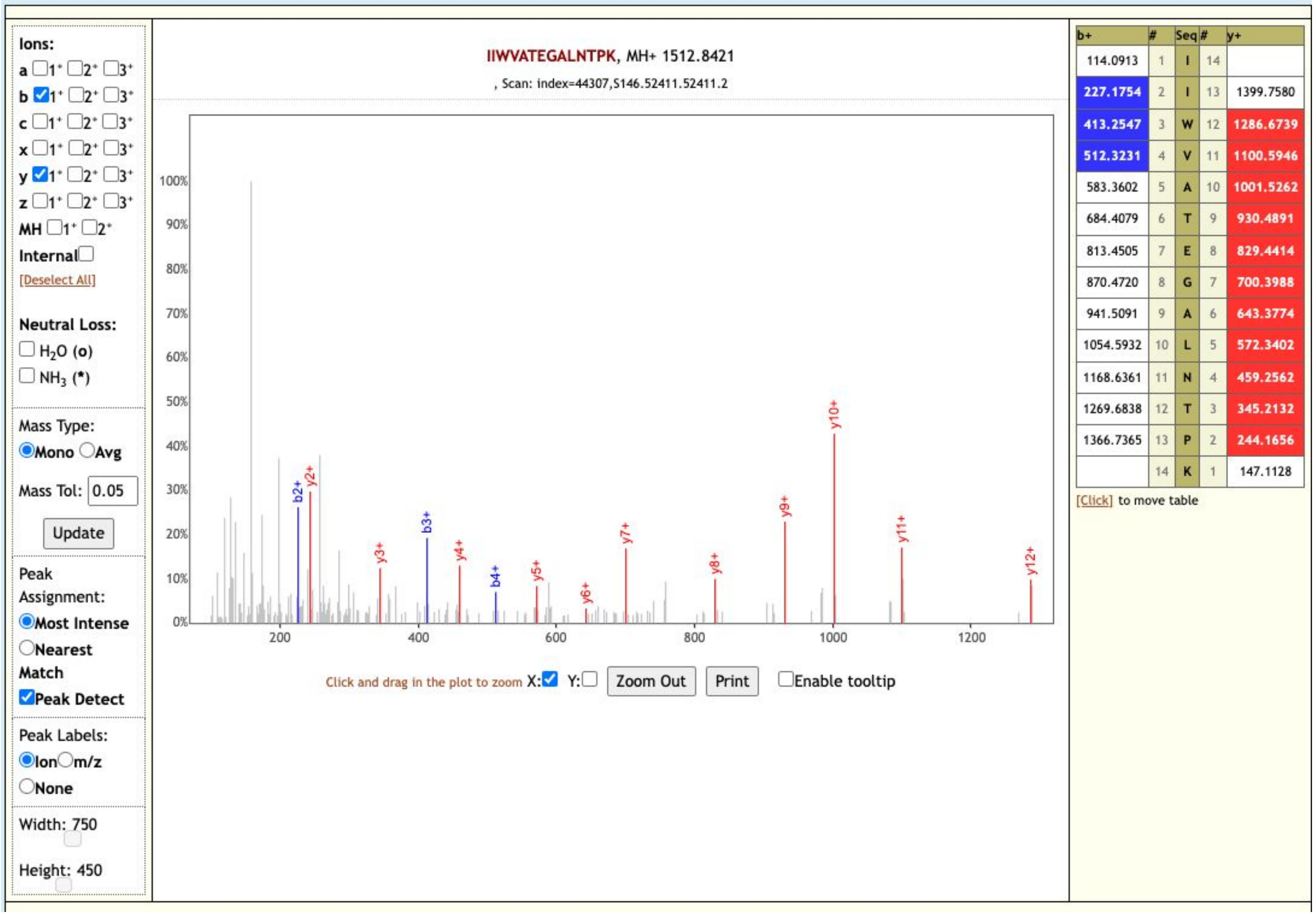

b+	#	Seq	#	y+
114.0913	1	I	14	
227.1754	2	I	13	1399.7580
413.2547	3	W	12	1286.6739
512.3231	4	V	11	1100.5946
583.3602	5	A	10	1001.5262
684.4079	6	T	9	930.4891
813.4505	7	E	8	829.4414
870.4720	8	G	7	700.3988
941.5091	9	A	6	643.3774
1054.5932	10	L	5	572.3402
1168.6361	11	N	4	459.2562
1269.6838	12	T	3	345.2132
1366.7365	13	P	2	244.1656
	14	K	1	147.1128

IWVATEGALNTPK

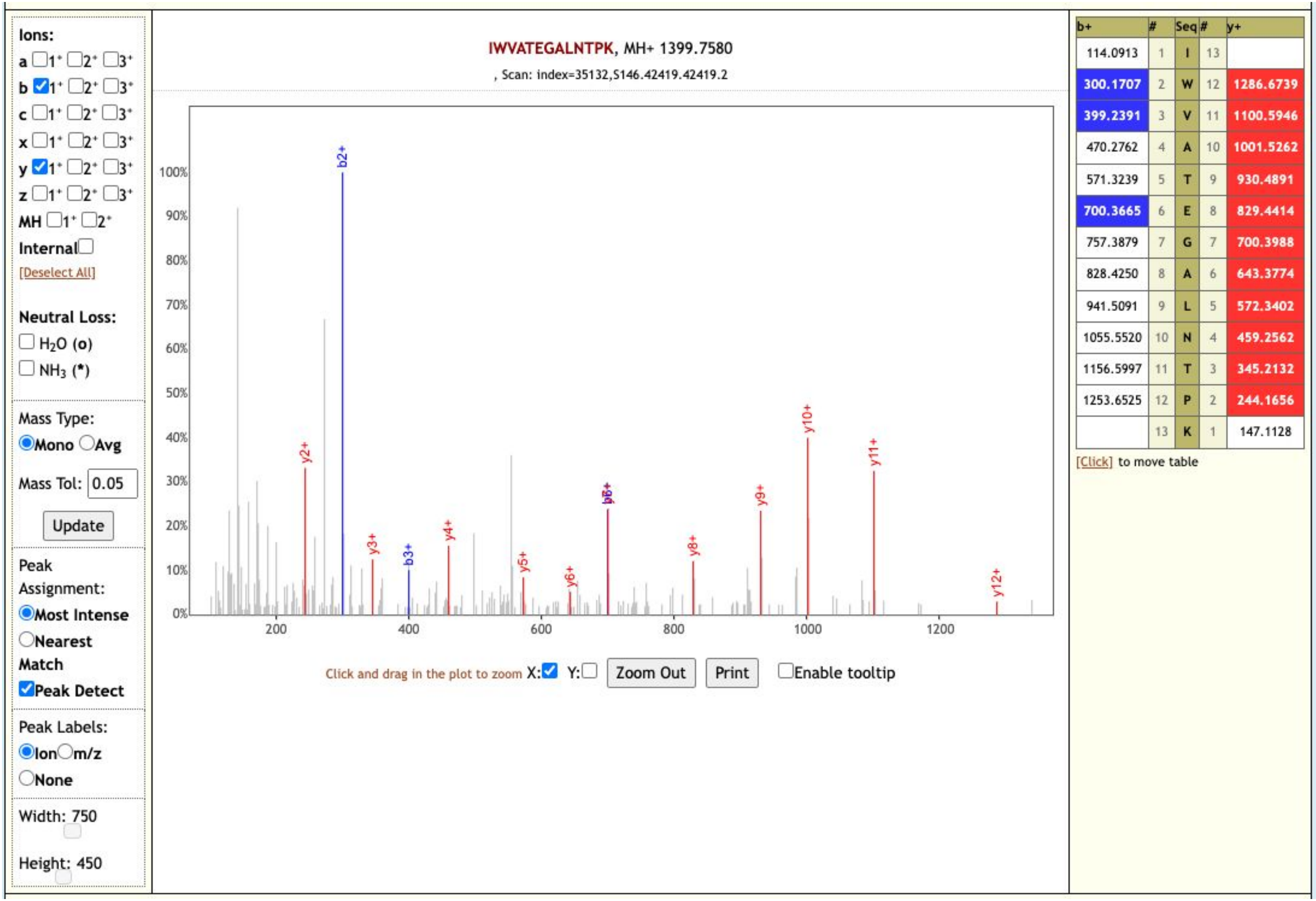

b+	#	Seq	#	y+
114.0913	1	I	13	
300.1707	2	W	12	1286.6739
399.2391	3	V	11	1100.5946
470.2762	4	A	10	1001.5262
571.3239	5	T	9	930.4891
700.3665	6	E	8	829.4414
757.3879	7	G	7	700.3988
828.4250	8	A	6	643.3774
941.5091	9	L	5	572.3402
1055.5520	10	N	4	459.2562
1156.5997	11	T	3	345.2132
1253.6525	12	P	2	244.1656
	13	K	1	147.1128

[Click] to move table

VATEGALNTPK

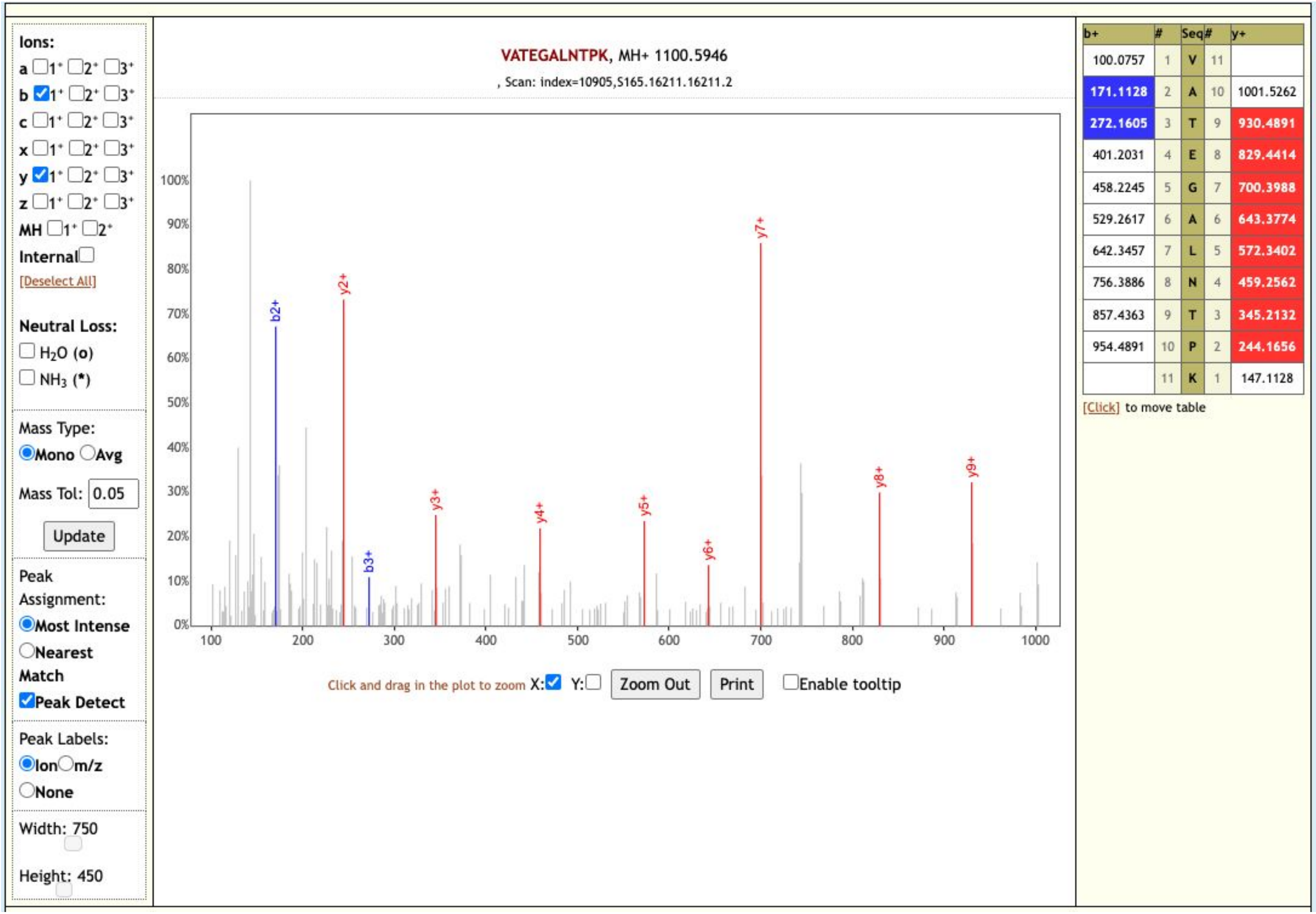

b+	#	Seq	#	y+
100.0757	1	V	11	
171.1128	2	A	10	1001.5262
272.1605	3	T	9	930.4891
401.2031	4	E	8	829.4414
458.2245	5	G	7	700.3988
529.2617	6	A	6	643.3774
642.3457	7	L	5	572.3402
756.3886	8	N	4	459.2562
857.4363	9	T	3	345.2132
954.4891	10	P	2	244.1656
	11	K	1	147.1128

DGIIWVATEGALNTPKG

Ions:
a ☐1⁺ ☐2⁺ ☐3⁺
b ☑1⁺ ☐2⁺ ☐3⁺
c ☐1⁺ ☐2⁺ ☐3⁺
x ☐1⁺ ☐2⁺ ☐3⁺
y ☑1⁺ ☐2⁺ ☐3⁺
z ☐1⁺ ☐2⁺ ☐3⁺
MH ☐1⁺ ☐2⁺
Internal ☐
[Deselect All]

Neutral Loss:
☐ H_2O (o)
☐ NH_3 (*)

Mass Type:
◉Mono ○Avg

Mass Tol: 0.05

Update

Peak Assignment:
◉Most Intense
○Nearest Match
☑Peak Detect

Peak Labels:
◉Ion ○m/z
○None

Width: 750

Height: 450

DGIIWVATEGALNTPKG, MH+ 1741.9119
, Scan: index=56572,S165.65336.65336.2

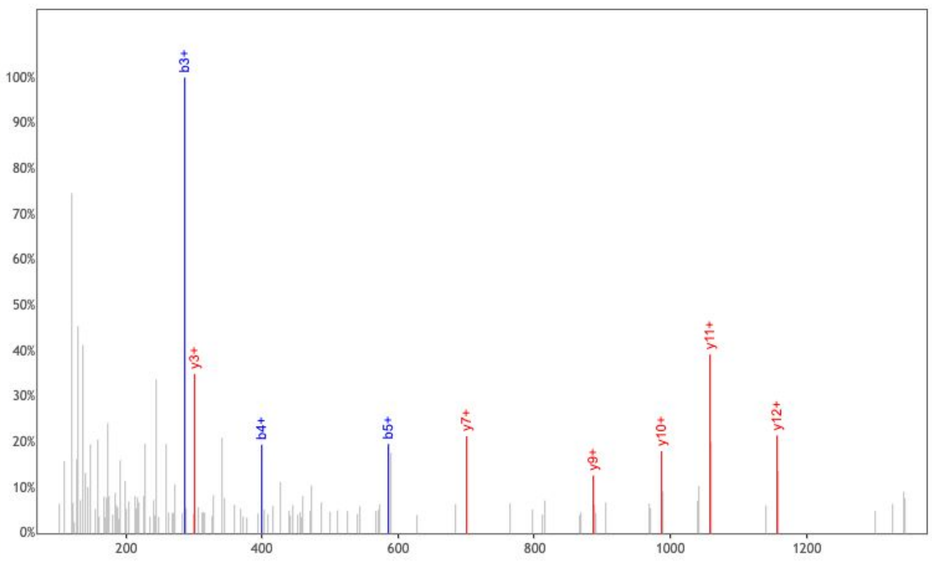

Click and drag in the plot to zoom X:☑ Y:☐ Zoom Out Print ☐Enable tooltip

b+	#	Seq	#	y+
116.0342	1	D	17	
173.0557	2	G	16	1626.8850
286.1397	3	I	15	1569.8635
399.2238	4	I	14	1456.7795
585.3031	5	W	13	1343.6954
684.3715	6	V	12	1157.6161
755.4087	7	A	11	1058.5477
856.4563	8	T	10	987.5106
985.4989	9	E	9	886.4629
1042.5204	10	G	8	757.4203
1113.5575	11	A	7	700.3988
1226.6416	12	L	6	629.3617
1340.6845	13	N	5	516.2776
1441.7322	14	T	4	402.2347
1538.7849	15	P	3	301.1870
1666.8799	16	K	2	204.1343
	17	G	1	76.0393

[Click] to move table

WVATEGALNTPK

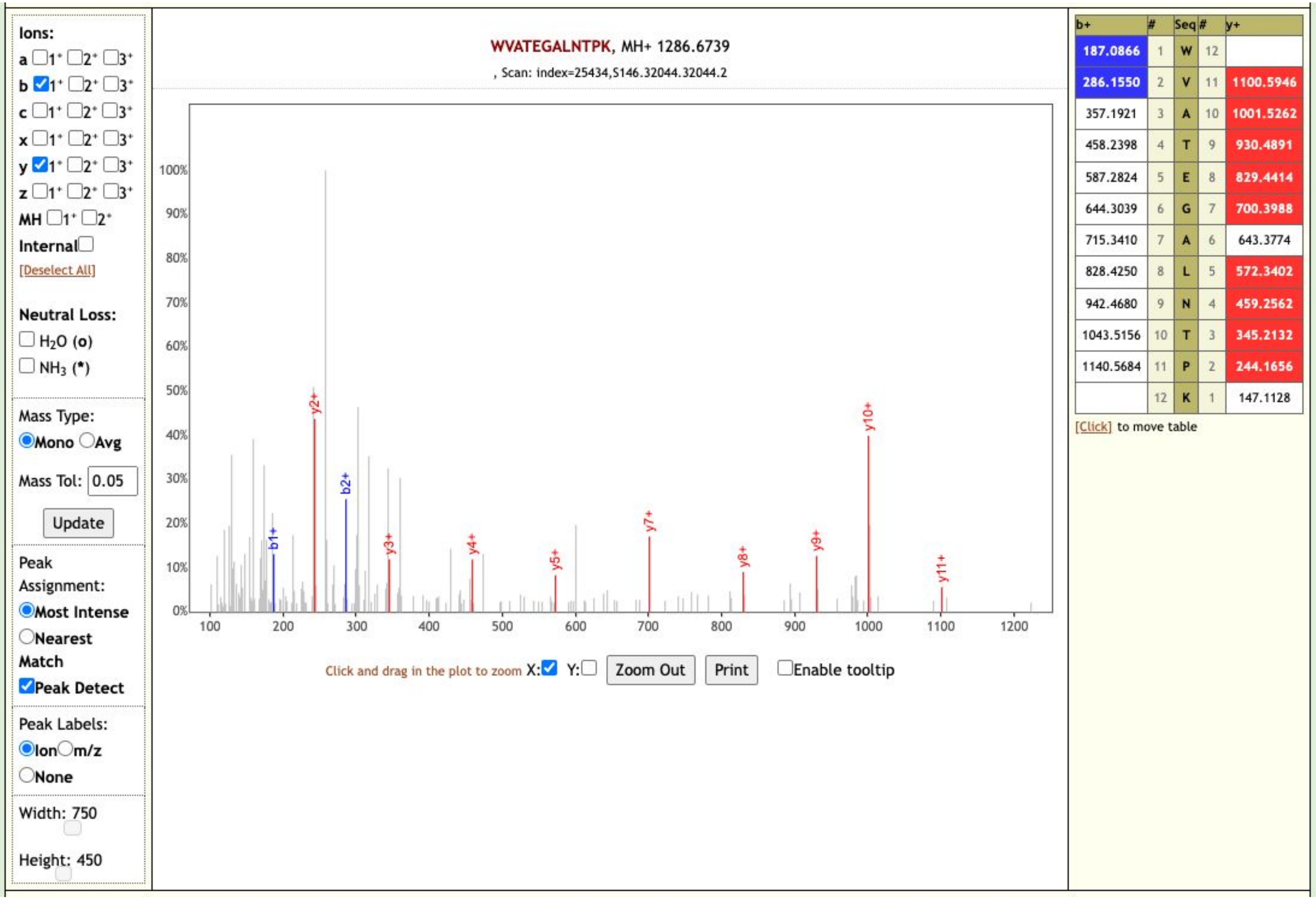

b+	#	Seq	#	y+
187.0866	1	W	12	
286.1550	2	V	11	1100.5946
357.1921	3	A	10	1001.5262
458.2398	4	T	9	930.4891
587.2824	5	E	8	829.4414
644.3039	6	G	7	700.3988
715.3410	7	A	6	643.3774
828.4250	8	L	5	572.3402
942.4680	9	N	4	459.2562
1043.5156	10	T	3	345.2132
1140.5684	11	P	2	244.1656
	12	K	1	147.1128

[Click] to move table

PXD024967

KADETQALPQR

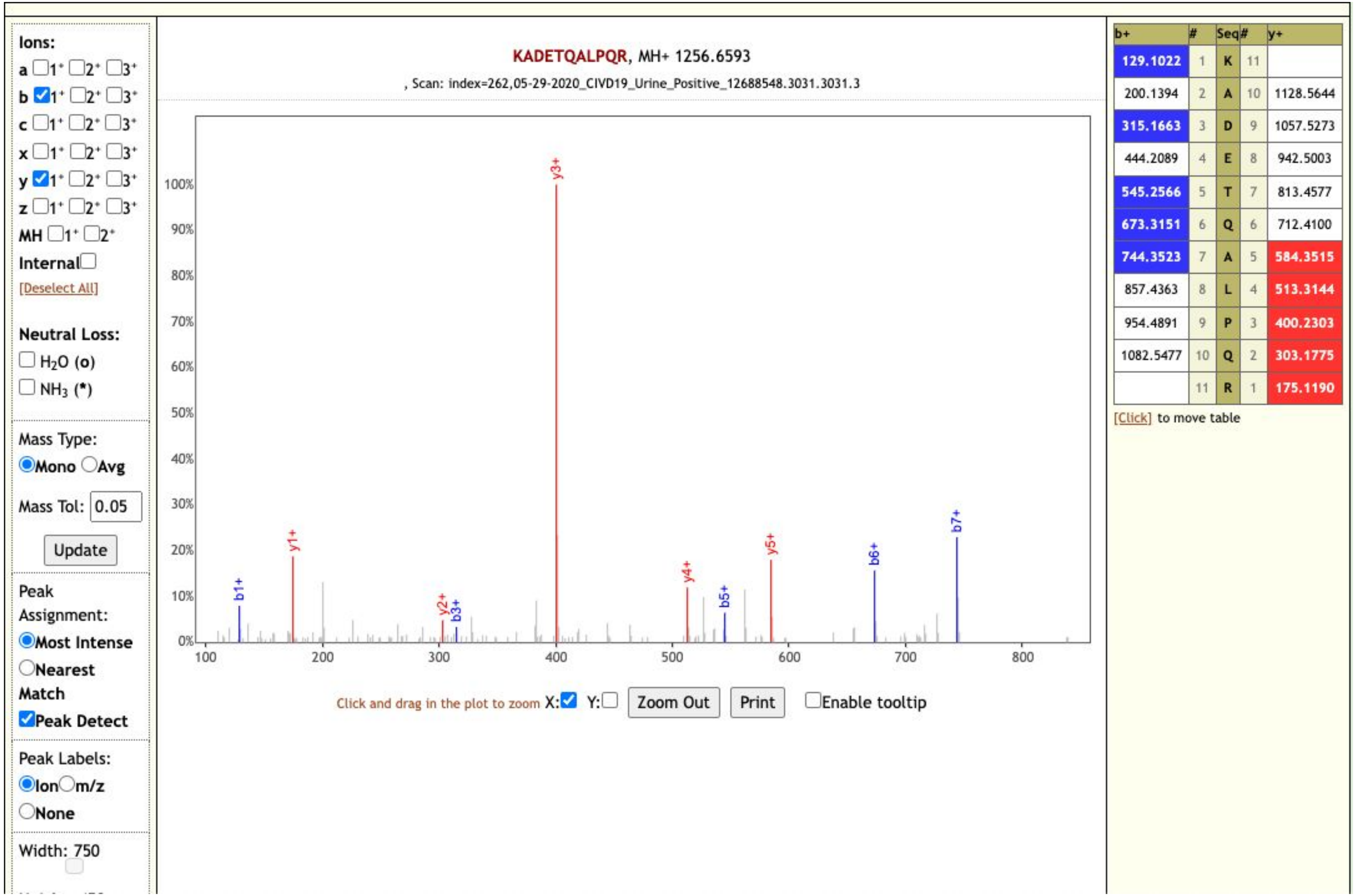

b+	#	Seq	#	y+
129.1022	1	K	11	
200.1394	2	A	10	1128.5644
315.1663	3	D	9	1057.5273
444.2089	4	E	8	942.5003
545.2566	5	T	7	813.4577
673.3151	6	Q	6	712.4100
744.3523	7	A	5	584.3515
857.4363	8	L	4	513.3144
954.4891	9	P	3	400.2303
1082.5477	10	Q	2	303.1775
	11	R	1	175.1190

KADETQALPQR

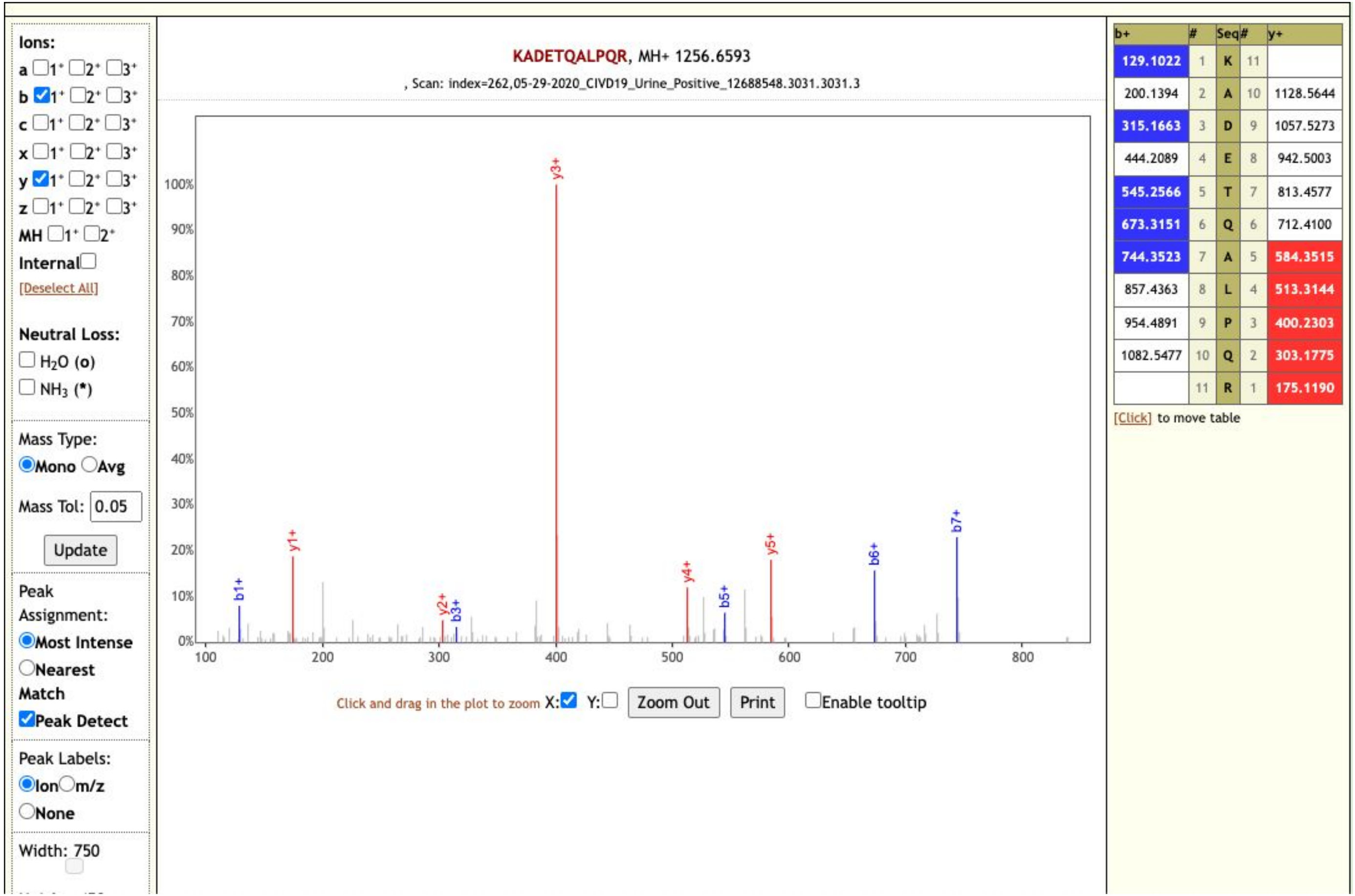

b+	#	Seq	#	y+
129.1022	1	K	11	
200.1394	2	A	10	1128.5644
315.1663	3	D	9	1057.5273
444.2089	4	E	8	942.5003
545.2566	5	T	7	813.4577
673.3151	6	Q	6	712.4100
744.3523	7	A	5	584.3515
857.4363	8	L	4	513.3144
954.4891	9	P	3	400.2303
1082.5477	10	Q	2	303.1775
	11	R	1	175.1190

QQTVTLLPAADLDDFSK

Ions:
a 1+ 2+ 3+
b 1+ 2+ 3+
c 1+ 2+ 3+
x 1+ 2+ 3+
y 1+ 2+ 3+
z 1+ 2+ 3+
MH 1+ 2+
Internal
[Deselect All]

Neutral Loss:
H_2O (o)
NH_3 (*)

Mass Type:
Mono Avg

Mass Tol: 0.05

Update

Peak Assignment:
Most Intense
Nearest Match
Peak Detect

Peak Labels:
Ion m/z
None

Width: 750

Height: 450

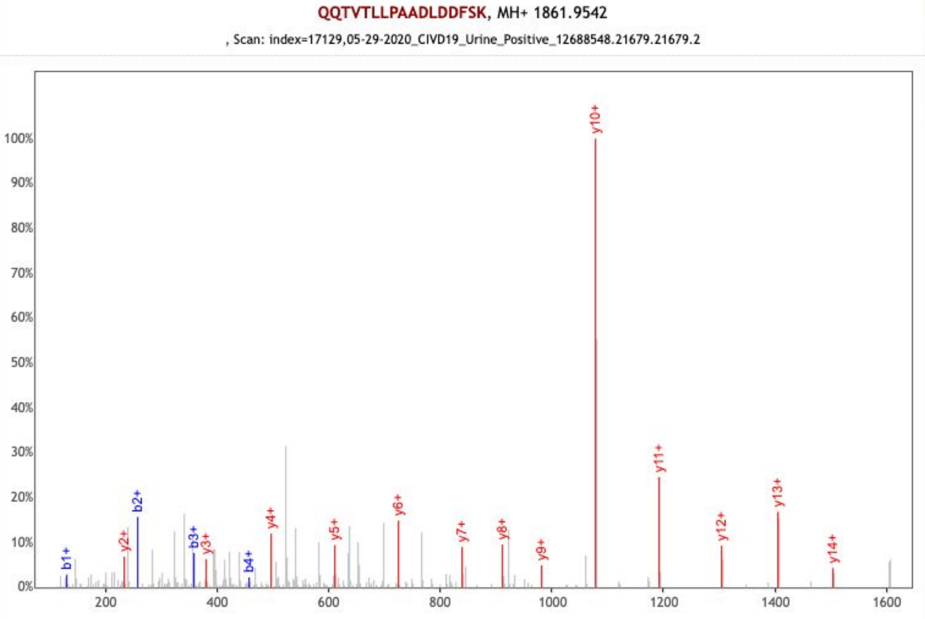

Click and drag in the plot to zoom X: Y: Zoom Out Print Enable tooltip

b+	#	Seq	#	y+
129.0659	1	Q	17	
257.1244	2	Q	16	1733.8956
358.1721	3	T	15	1605.8370
457.2405	4	V	14	1504.7894
558.2882	5	T	13	1405.7209
671.3723	6	L	12	1304.6733
784.4563	7	L	11	1191.5892
881.5091	8	P	10	1078.5051
952.5462	9	A	9	981.4524
1023.5833	10	A	8	910.4153
1138.6103	11	D	7	839.3781
1251.6943	12	L	6	724.3512
1366.7213	13	D	5	611.2671
1481.7482	14	D	4	496.2402
1628.8166	15	F	3	381.2132
1715.8487	16	S	2	234.1448
	17	K	1	147.1128

[Click] to move table

KQQTVTLLPAADLDDFSK

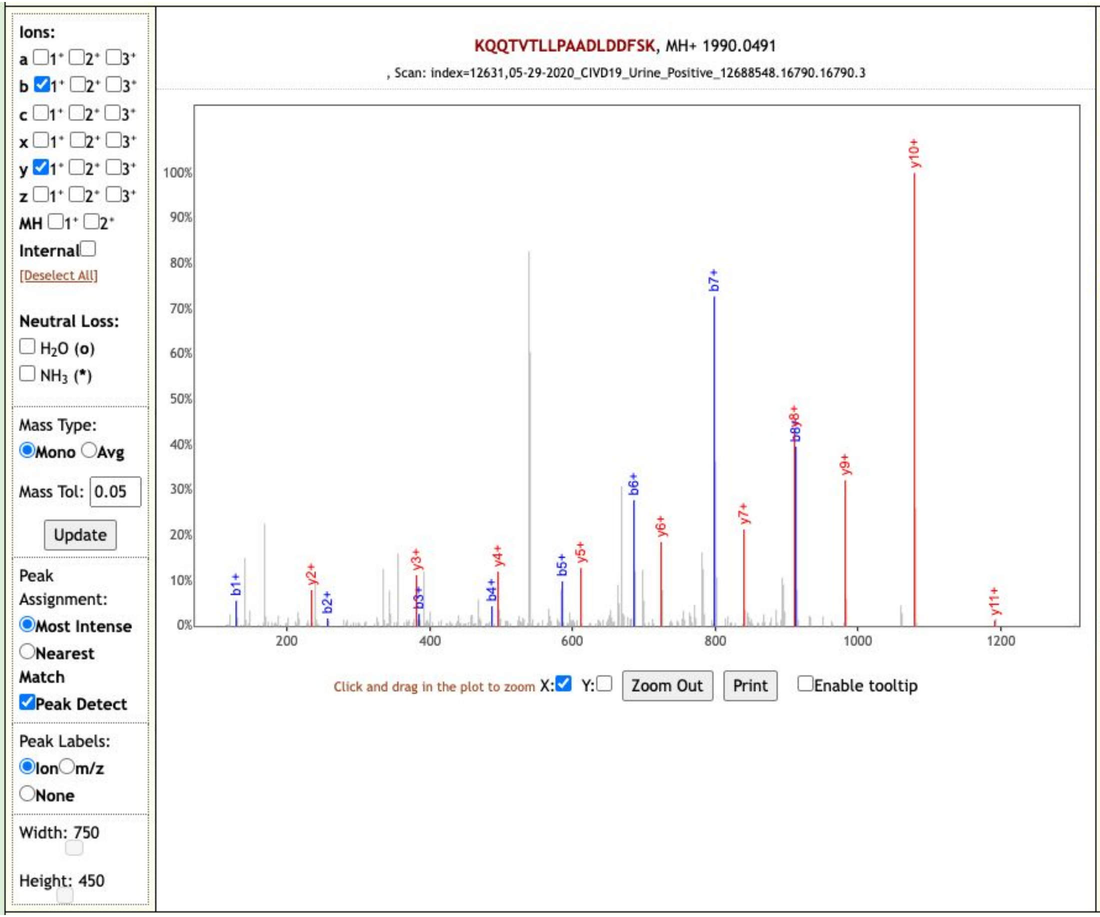

b+	#	Seq	#	y+
129.1022	1	K	18	
257.1608	2	Q	17	1861.9542
385.2194	3	Q	16	1733.8956
486.2671	4	T	15	1605.8370
585.3355	5	V	14	1504.7894
686.3832	6	T	13	1405.7209
799.4672	7	L	12	1304.6733
912.5513	8	L	11	1191.5892
1009.6041	9	P	10	1078.5051
1080.6412	10	A	9	981.4524
1151.6783	11	A	8	910.4153
1266.7052	12	D	7	839.3781
1379.7893	13	L	6	724.3512
1494.8162	14	D	5	611.2671
1609.8432	15	D	4	496.2402
1756.9116	16	F	3	381.2132
1843.9436	17	S	2	234.1448
	18	K	1	147.1128

[Click] to move table

ADETQALPQR

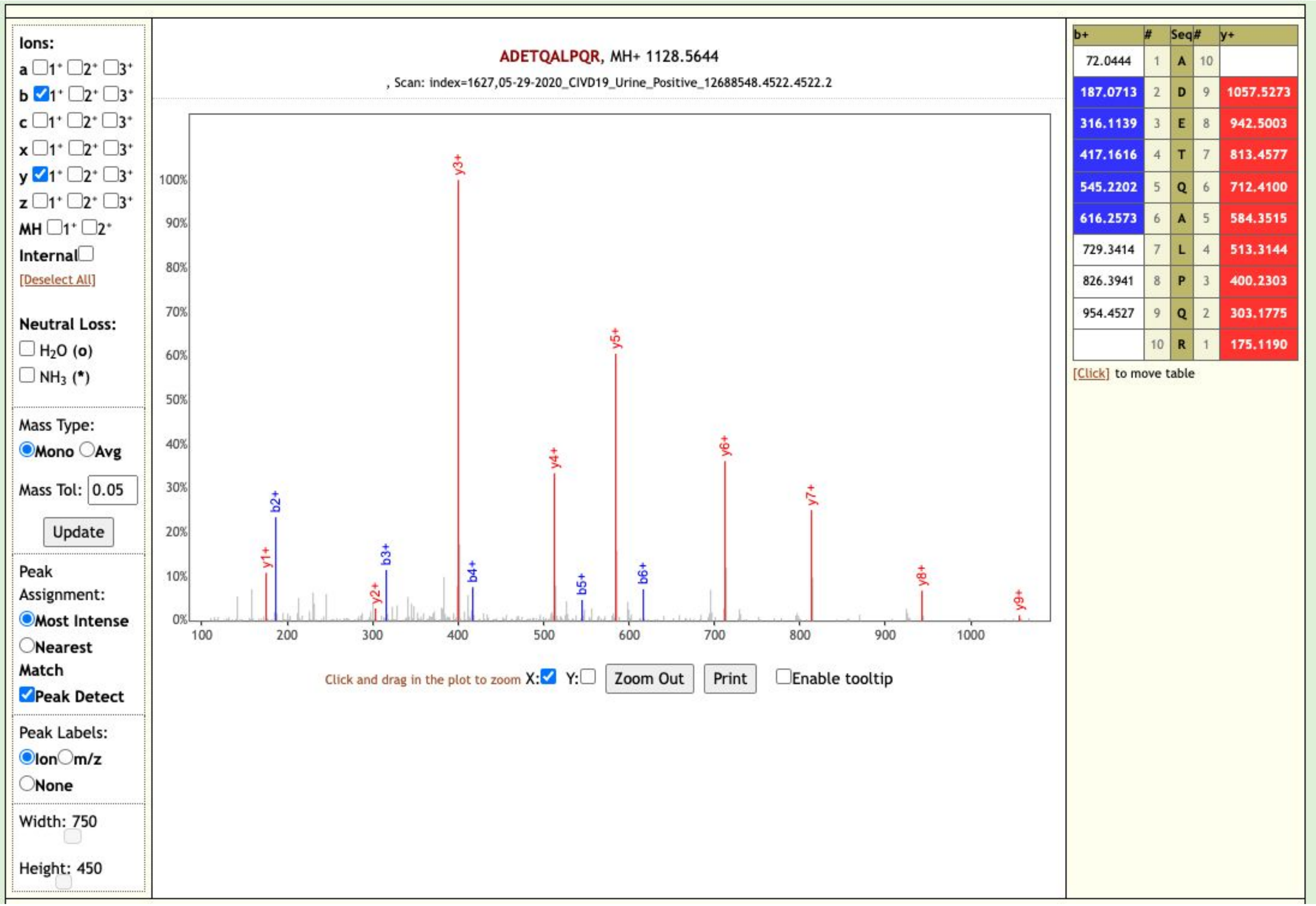

b+	#	Seq	#	y+
72.0444	1	A	10	
187.0713	2	D	9	1057.5273
316.1139	3	E	8	942.5003
417.1616	4	T	7	813.4577
545.2202	5	Q	6	712.4100
616.2573	6	A	5	584.3515
729.3414	7	L	4	513.3144
826.3941	8	P	3	400.2303
954.4527	9	Q	2	303.1775
	10	R	1	175.1190

QLQQSMSSADSTQA

Ions:
a 1+ 2+ 3+
b 1+ 2+ 3+
c 1+ 2+ 3+
x 1+ 2+ 3+
y 1+ 2+ 3+
z 1+ 2+ 3+
MH 1+ 2+
Internal
[Deselect All]

Neutral Loss:
H_2O (o)
NH_3 (*)

Mass Type:
Mono Avg

Mass Tol: 0.05

Update

Peak Assignment:
Most Intense
Nearest Match
Peak Detect

Peak Labels:
Ion m/z
None

Width: 750

Height: 450

QLQQSMSSADSTQA, MH+ 1497.6486

, Scan: index=1066,05-29-2020_CIVD19_Urine_Positive_12688548.3894.3894.2

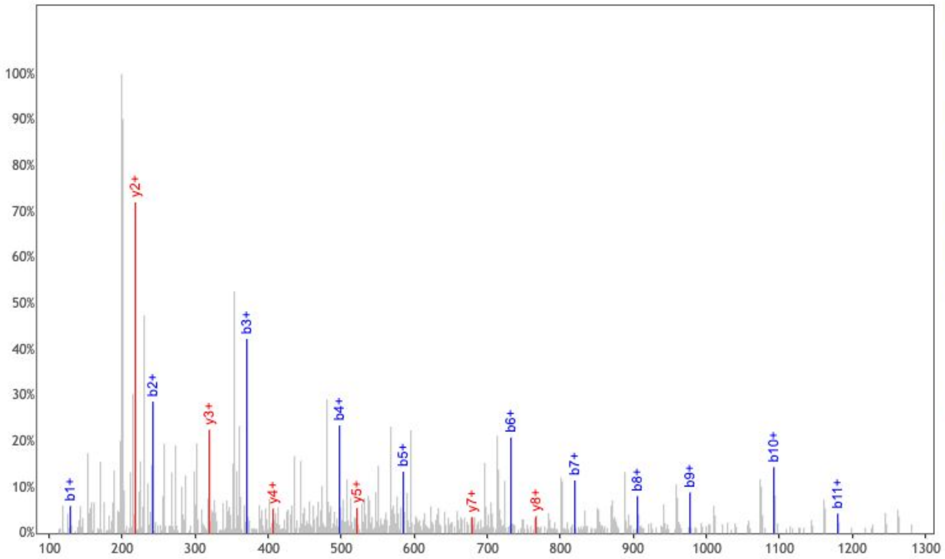

Click and drag in the plot to zoom X: Y: Zoom Out Print Enable tooltip

b+	#	Seq	#	y+
129.0659	1	Q	14	
242.1499	2	L	13	1369.5900
370.2085	3	Q	12	1256.5059
498.2671	4	Q	11	1128.4474
585.2991	5	S	10	1000.3888
732.3345	6	M	9	913.3568
819.3665	7	S	8	766.3214
906.3986	8	S	7	679.2893
977.4357	9	A	6	592.2573
1092.4626	10	D	5	521.2202
1179.4946	11	S	4	406.1932
1280.5423	12	T	3	319.1612
1408.6009	13	Q	2	218.1135
	14	A	1	90.0550

[Click] to move table

Variable Modifications:

M: 15.994915 [6]

PAADLDDFSK

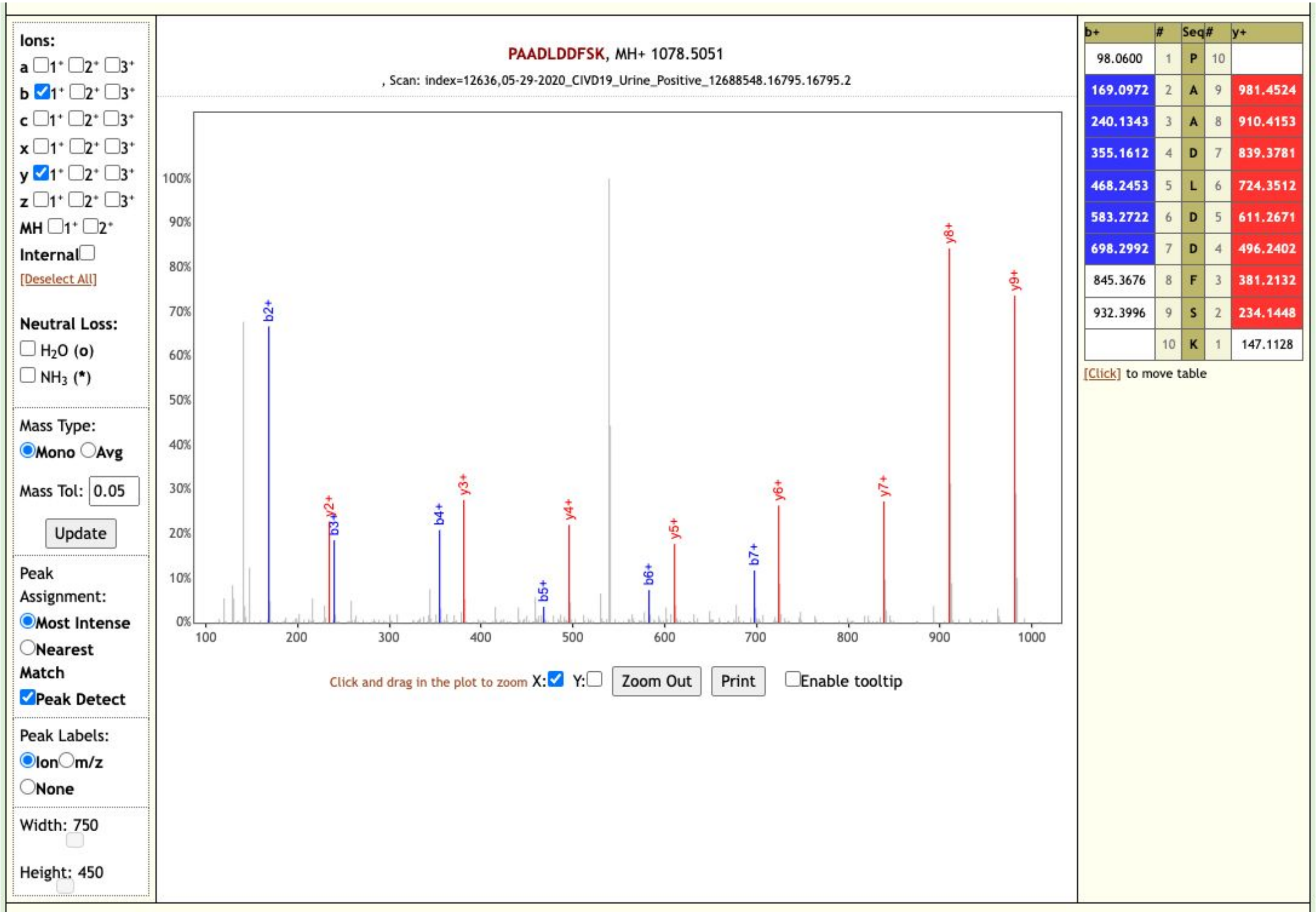

b+	#	Seq	#	y+
98.0600	1	P	10	
169.0972	2	A	9	981.4524
240.1343	3	A	8	910.4153
355.1612	4	D	7	839.3781
468.2453	5	L	6	724.3512
583.2722	6	D	5	611.2671
698.2992	7	D	4	496.2402
845.3676	8	F	3	381.2132
932.3996	9	S	2	234.1448
	10	K	1	147.1128

[Click] to move table

ITFGGPSDSTGSNQNGER

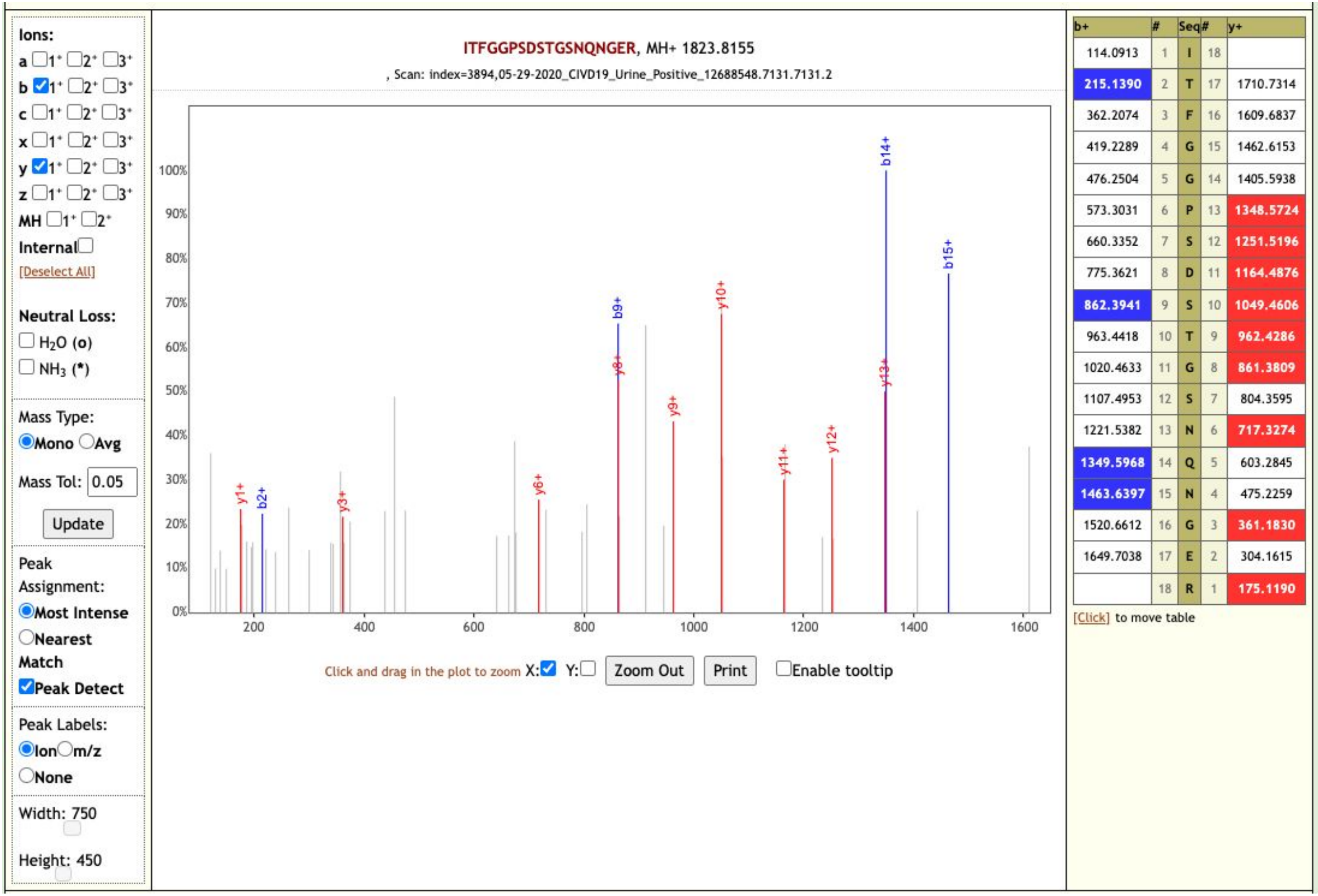

b+	#	Seq	#	y+
114.0913	1	I	18	
215.1390	2	T	17	1710.7314
362.2074	3	F	16	1609.6837
419.2289	4	G	15	1462.6153
476.2504	5	G	14	1405.5938
573.3031	6	P	13	1348.5724
660.3352	7	S	12	1251.5196
775.3621	8	D	11	1164.4876
862.3941	9	S	10	1049.4606
963.4418	10	T	9	962.4286
1020.4633	11	G	8	861.3809
1107.4953	12	S	7	804.3595
1221.5382	13	N	6	717.3274
1349.5968	14	Q	5	603.2845
1463.6397	15	N	4	475.2259
1520.6612	16	G	3	361.1830
1649.7038	17	E	2	304.1615
	18	R	1	175.1190

[Click] to move table

QLQQSMSSADSTQA

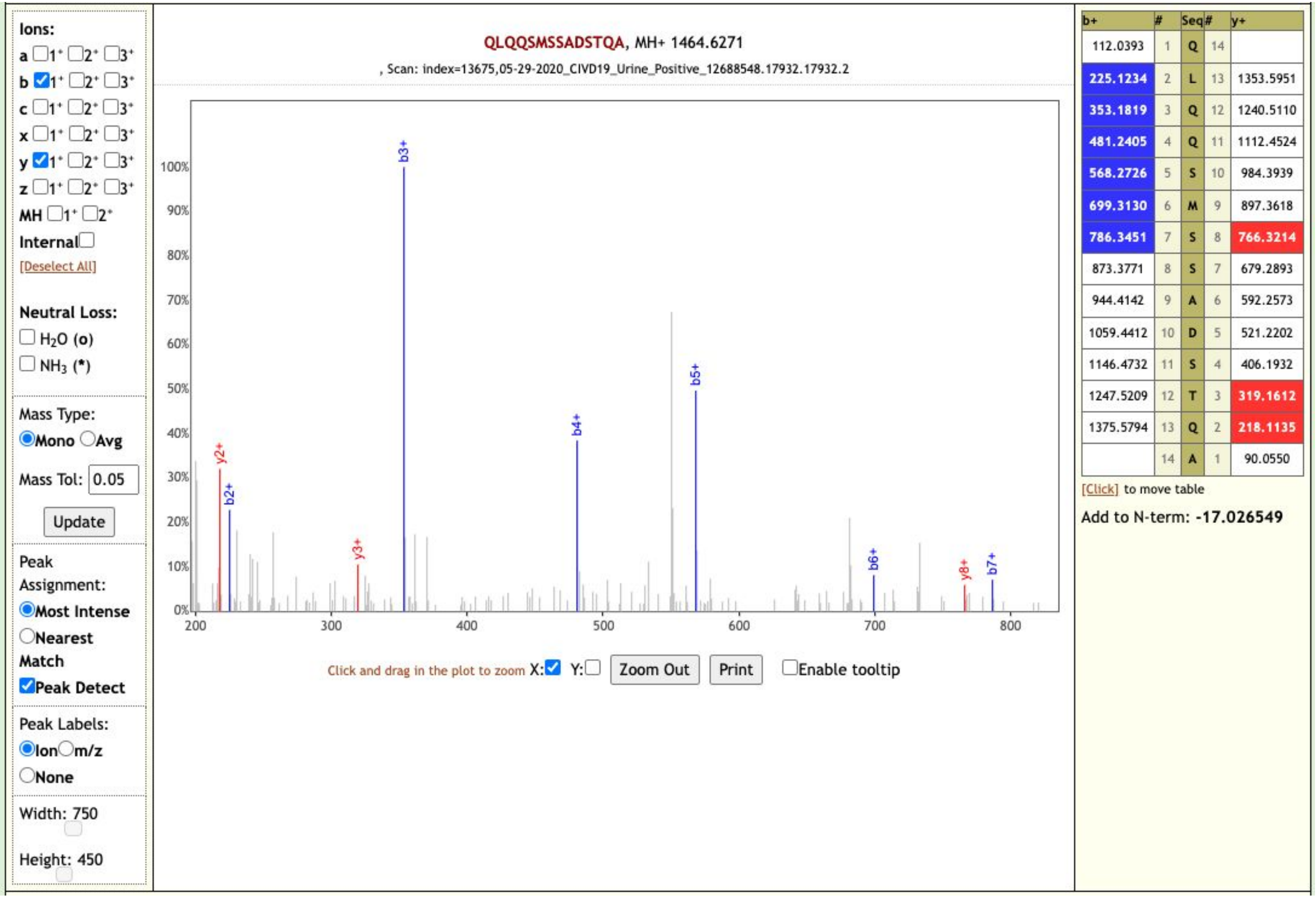

b+	#	Seq	#	y+
112.0393	1	Q	14	
225.1234	2	L	13	1353.5951
353.1819	3	Q	12	1240.5110
481.2405	4	Q	11	1112.4524
568.2726	5	S	10	984.3939
699.3130	6	M	9	897.3618
786.3451	7	S	8	766.3214
873.3771	8	S	7	679.2893
944.4142	9	A	6	592.2573
1059.4412	10	D	5	521.2202
1146.4732	11	S	4	406.1932
1247.5209	12	T	3	319.1612
1375.5794	13	Q	2	218.1135
	14	A	1	90.0550

[Click] to move table

Add to N-term: **-17.026549**

KADETQALPQR

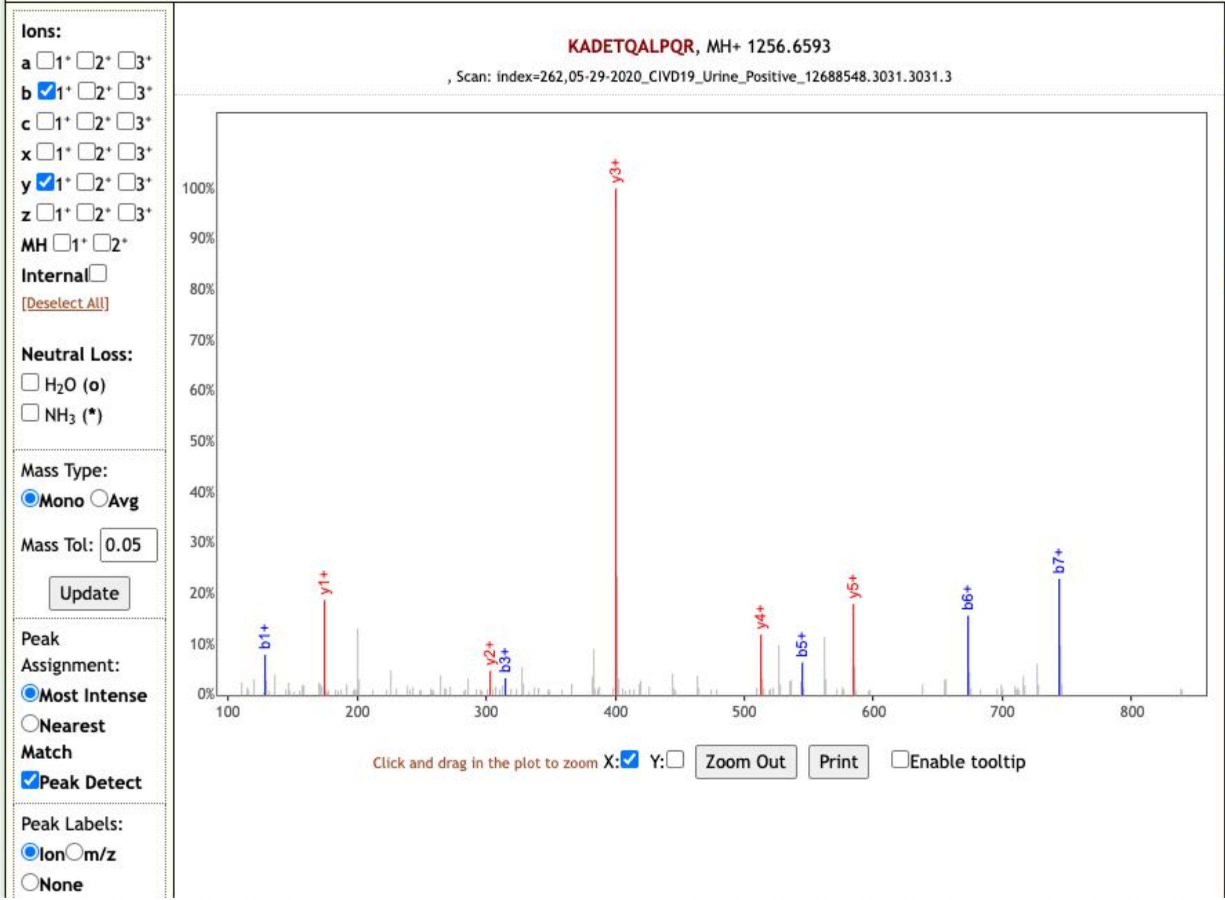

b+	#	Seq	#	y+
129.1022	1	K	11	
200.1394	2	A	10	1128.5644
315.1663	3	D	9	1057.5273
444.2089	4	E	8	942.5003
545.2566	5	T	7	813.4577
673.3151	6	Q	6	712.4100
744.3523	7	A	5	584.3515
857.4363	8	L	4	513.3144
954.4891	9	P	3	400.2303
1082.5477	10	Q	2	303.1775
	11	R	1	175.1190

[Click] to move table

QQTVTLLPAADLDDFSK

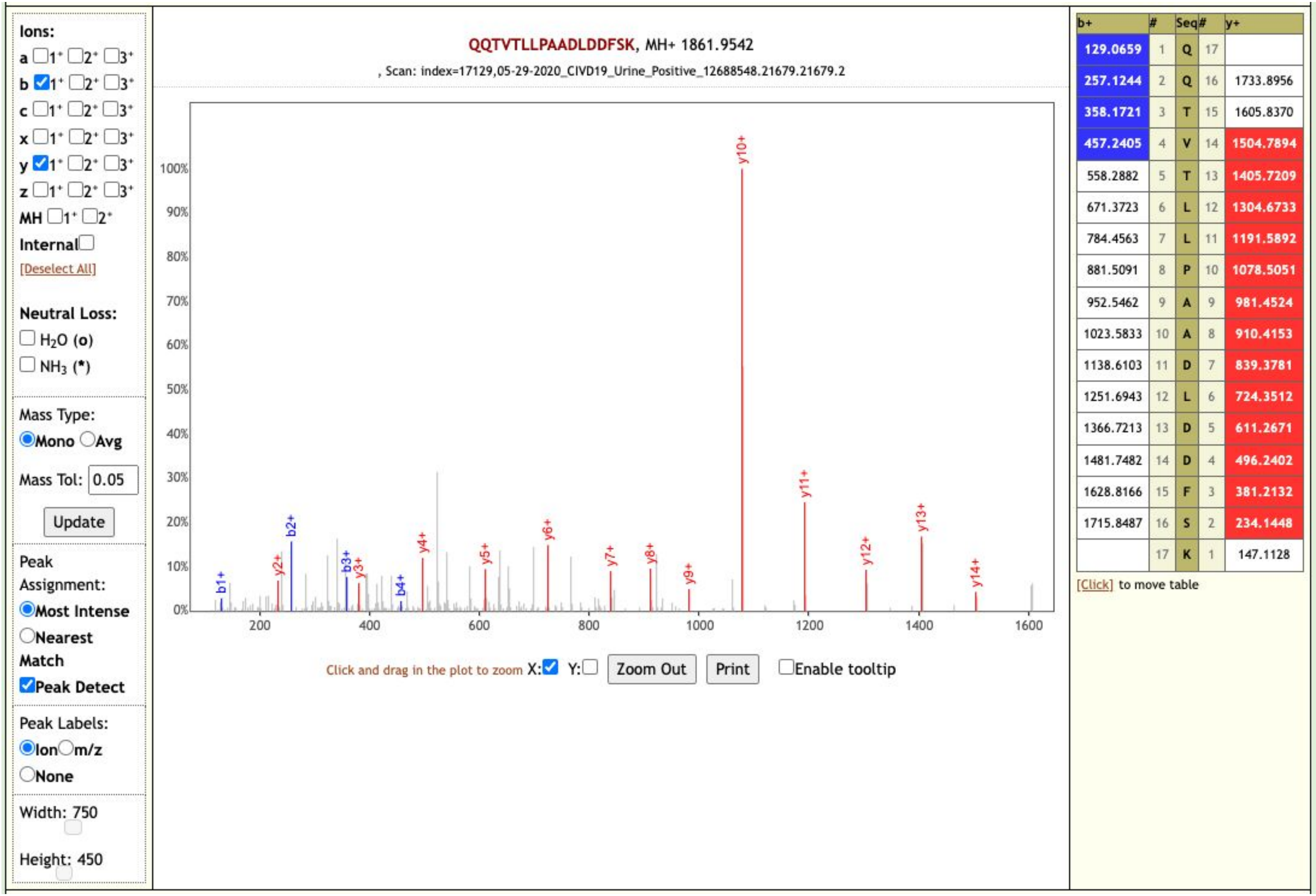

b+	#	Seq	#	y+
129.0659	1	Q	17	
257.1244	2	Q	16	1733.8956
358.1721	3	T	15	1605.8370
457.2405	4	V	14	1504.7894
558.2882	5	T	13	1405.7209
671.3723	6	L	12	1304.6733
784.4563	7	L	11	1191.5892
881.5091	8	P	10	1078.5051
952.5462	9	A	9	981.4524
1023.5833	10	A	8	910.4153
1138.6103	11	D	7	839.3781
1251.6943	12	L	6	724.3512
1366.7213	13	D	5	611.2671
1481.7482	14	D	4	496.2402
1628.8166	15	F	3	381.2132
1715.8487	16	S	2	234.1448
	17	K	1	147.1128

[Click] to move table

KQQTVTLLPAADLDDFSK

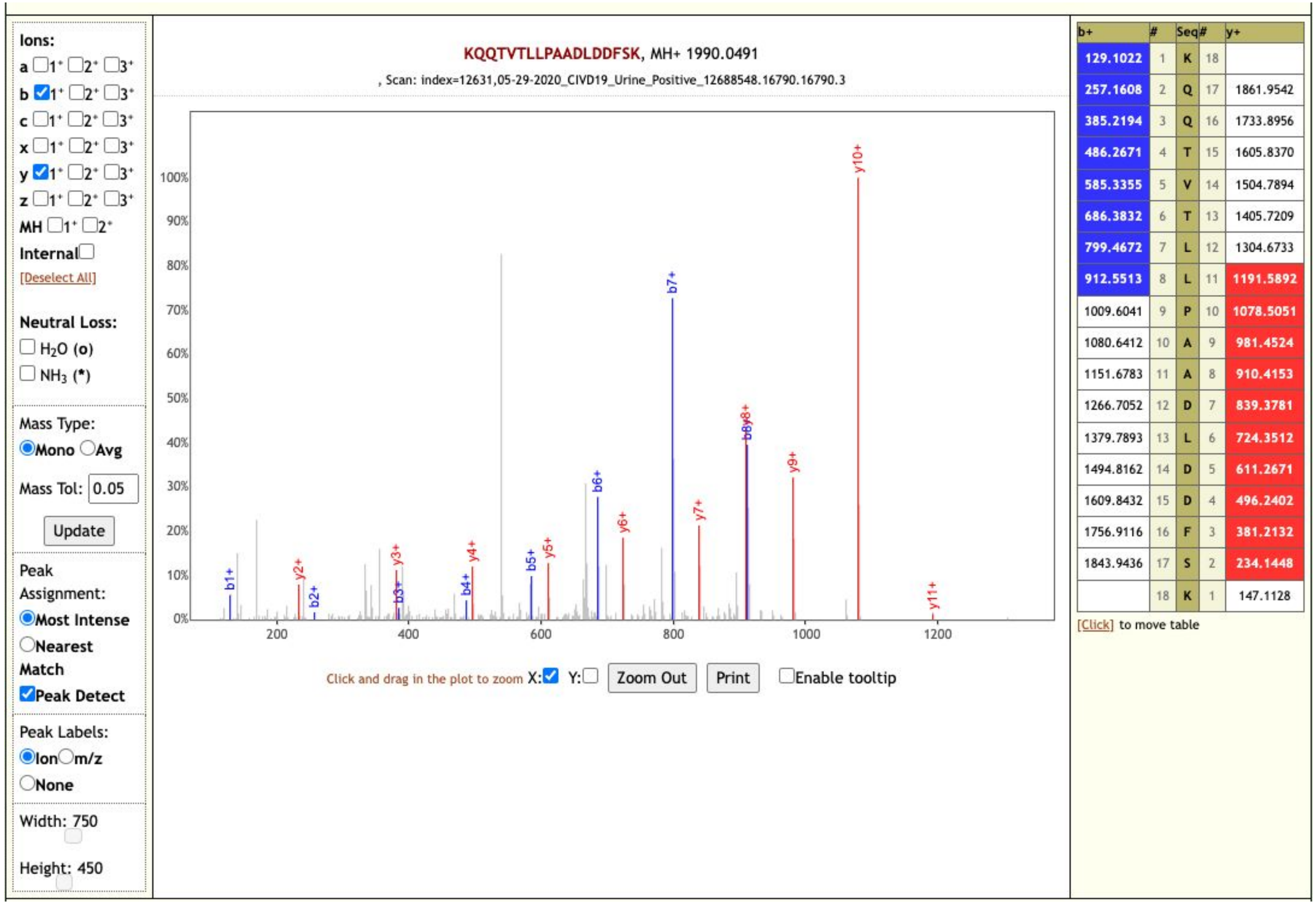

b+	#	Seq	#	y+
129.1022	1	K	18	
257.1608	2	Q	17	1861.9542
385.2194	3	Q	16	1733.8956
486.2671	4	T	15	1605.8370
585.3355	5	V	14	1504.7894
686.3832	6	T	13	1405.7209
799.4672	7	L	12	1304.6733
912.5513	8	L	11	1191.5892
1009.6041	9	P	10	1078.5051
1080.6412	10	A	9	981.4524
1151.6783	11	A	8	910.4153
1266.7052	12	D	7	839.3781
1379.7893	13	L	6	724.3512
1494.8162	14	D	5	611.2671
1609.8432	15	D	4	496.2402
1756.9116	16	F	3	381.2132
1843.9436	17	S	2	234.1448
	18	K	1	147.1128

[Click] to move table

QLQQSMSSADSTQA

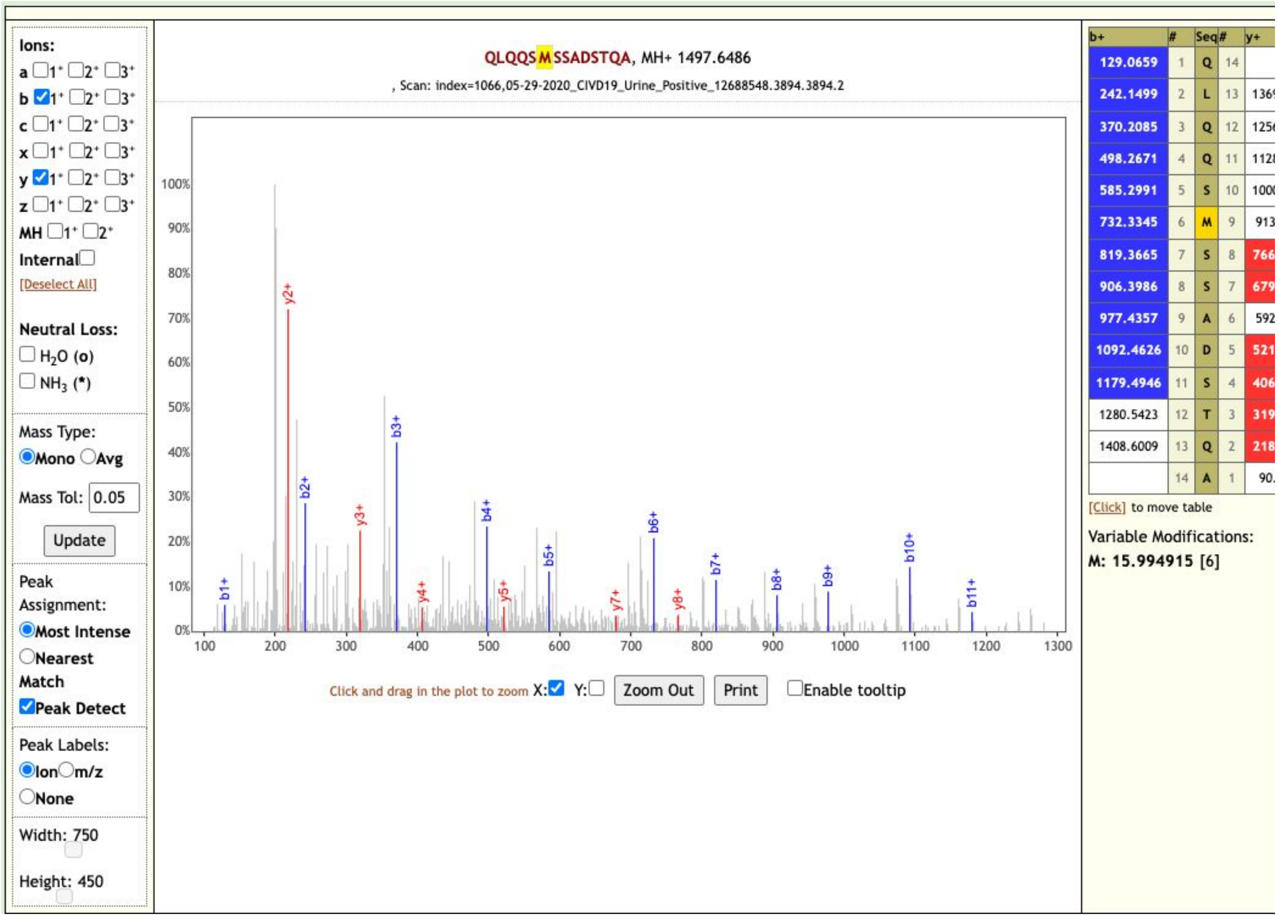

ADETQALPQR

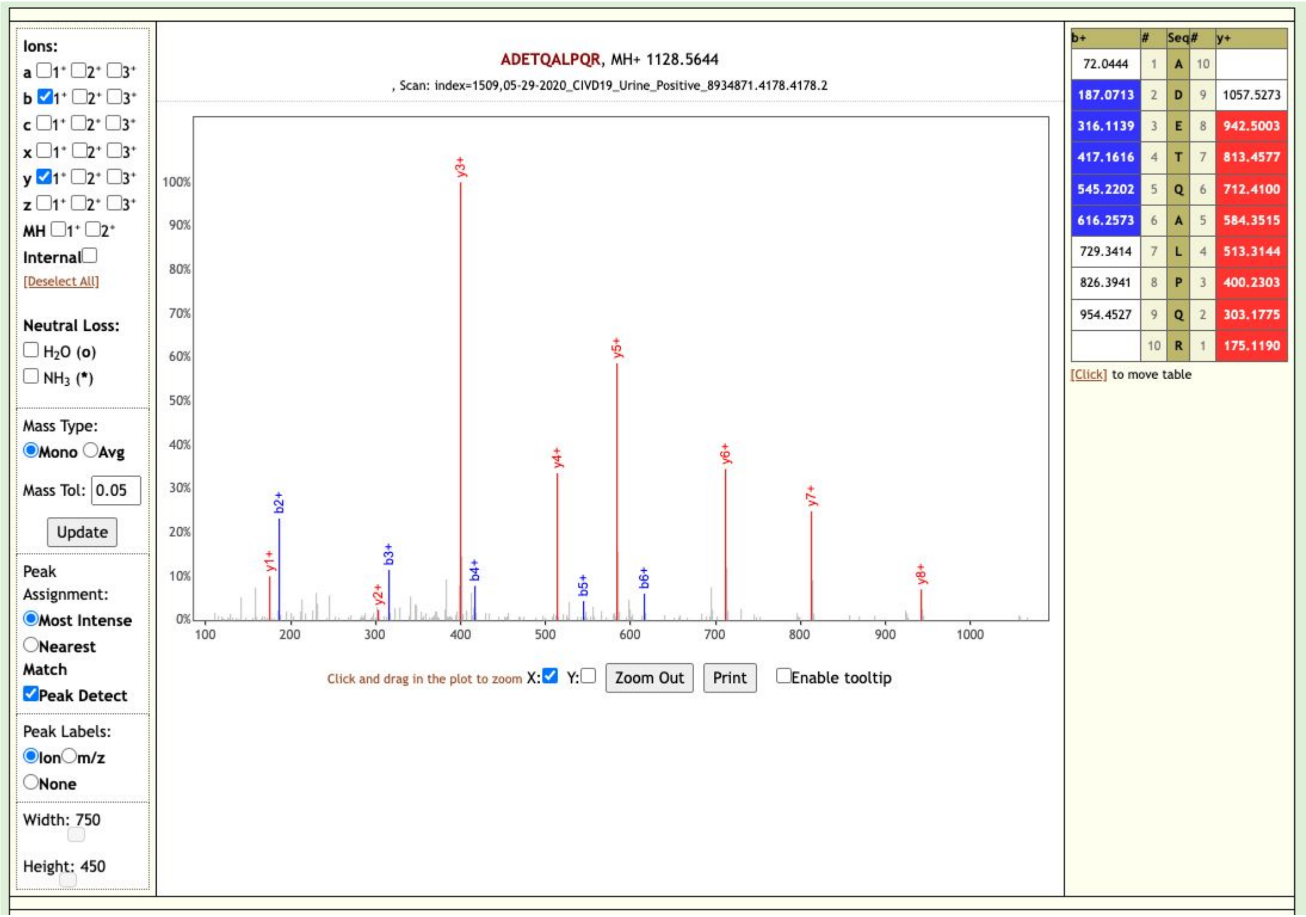

b+	#	Seq	#	y+
72.0444	1	A	10	
187.0713	2	D	9	1057.5273
316.1139	3	E	8	942.5003
417.1616	4	T	7	813.4577
545.2202	5	Q	6	712.4100
616.2573	6	A	5	584.3515
729.3414	7	L	4	513.3144
826.3941	8	P	3	400.2303
954.4527	9	Q	2	303.1775
	10	R	1	175.1190

ITFGGPSDSTGSNQNGER

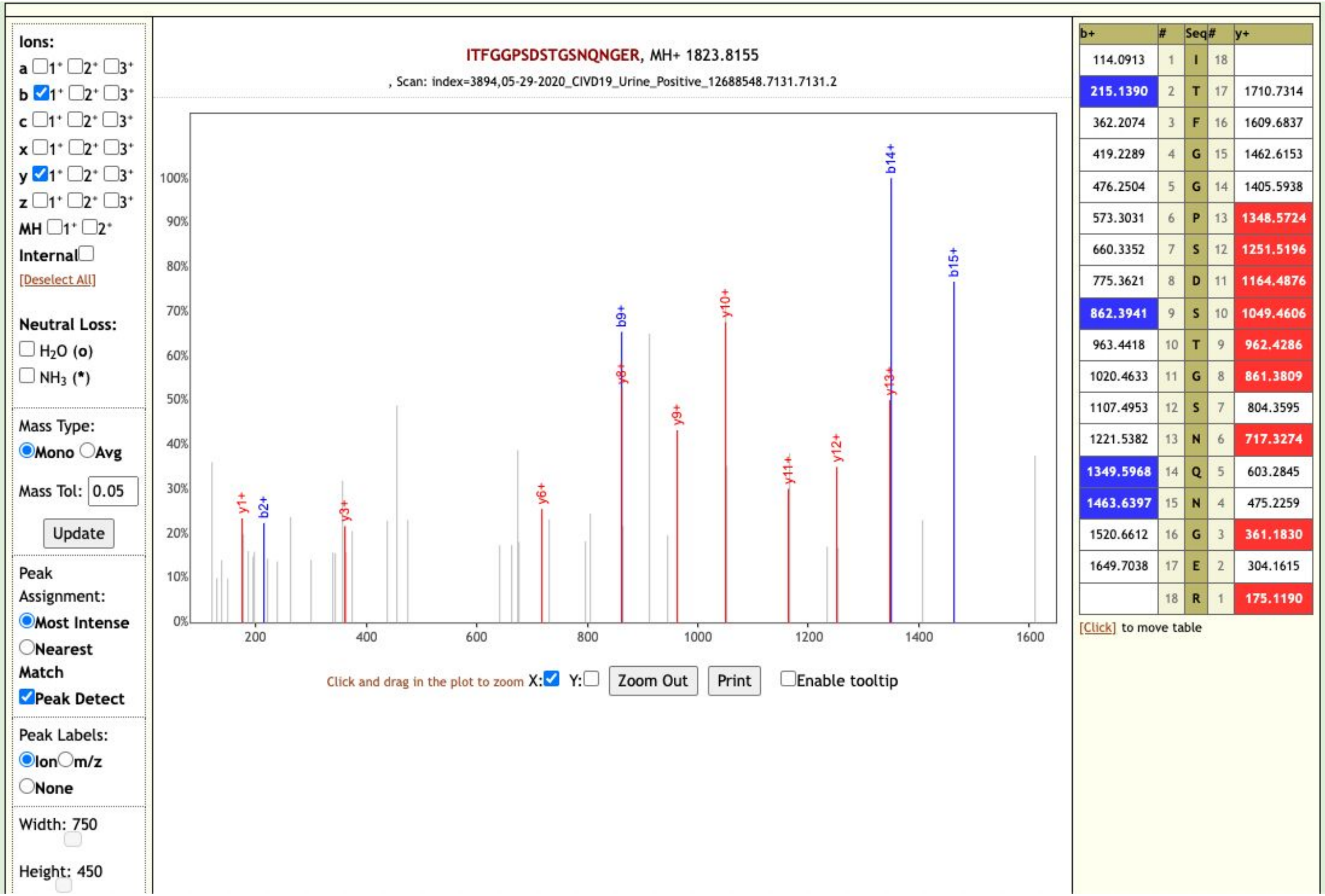

b+	#	Seq	#	y+
114.0913	1	I	18	
215.1390	2	T	17	1710.7314
362.2074	3	F	16	1609.6837
419.2289	4	G	15	1462.6153
476.2504	5	G	14	1405.5938
573.3031	6	P	13	1348.5724
660.3352	7	S	12	1251.5196
775.3621	8	D	11	1164.4876
862.3941	9	S	10	1049.4606
963.4418	10	T	9	962.4286
1020.4633	11	G	8	861.3809
1107.4953	12	S	7	804.3595
1221.5382	13	N	6	717.3274
1349.5968	14	Q	5	603.2845
1463.6397	15	N	4	475.2259
1520.6612	16	G	3	361.1830
1649.7038	17	E	2	304.1615
	18	R	1	175.1190

[Click] to move table

PAADLDDFSK

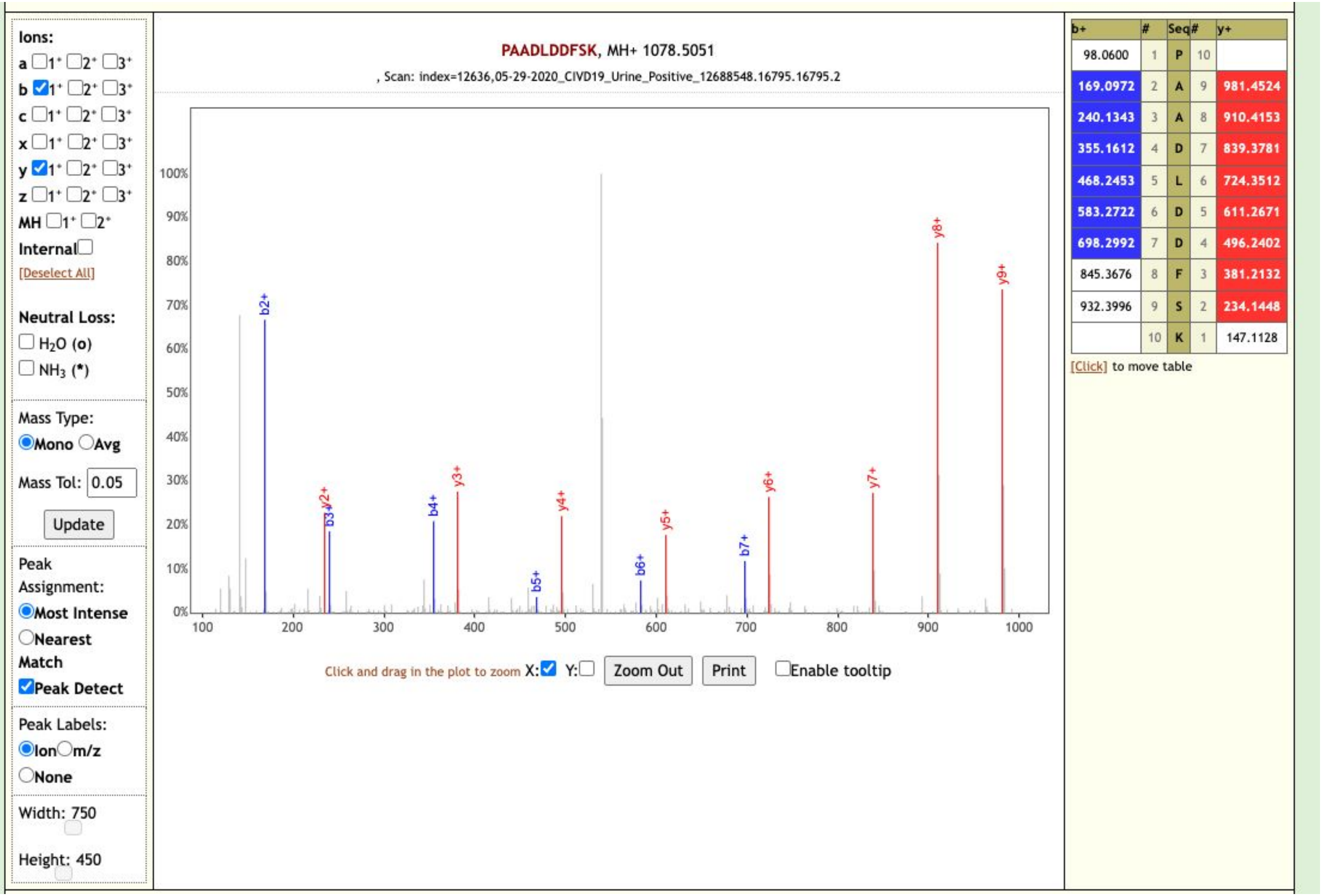

b+	#	Seq	#	y+
98.0600	1	P	10	
169.0972	2	A	9	981.4524
240.1343	3	A	8	910.4153
355.1612	4	D	7	839.3781
468.2453	5	L	6	724.3512
583.2722	6	D	5	611.2671
698.2992	7	D	4	496.2402
845.3676	8	F	3	381.2132
932.3996	9	S	2	234.1448
	10	K	1	147.1128

QLQQSMSSADSTQA

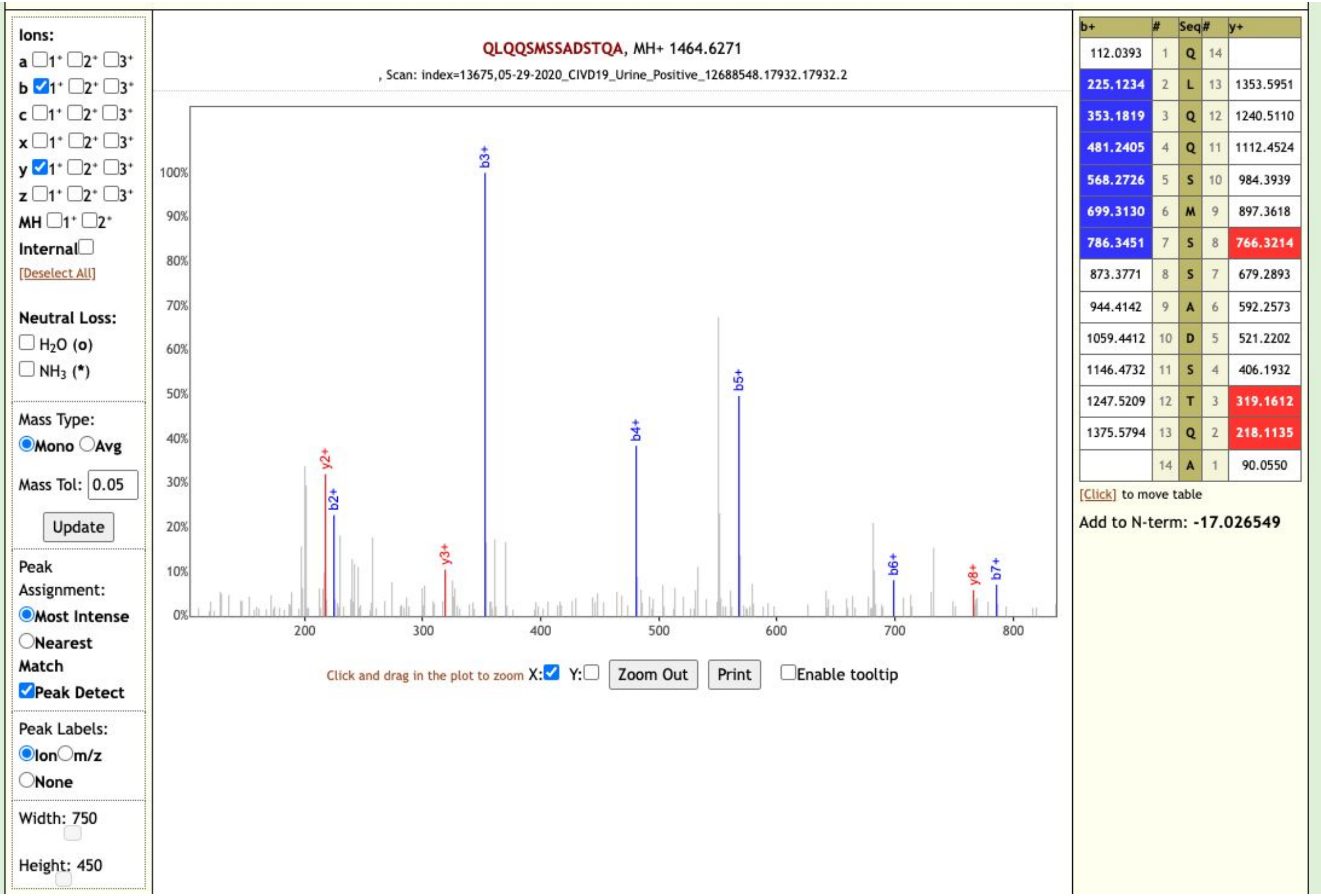

b+	#	Seq	#	y+
112.0393	1	Q	14	
225.1234	2	L	13	1353.5951
353.1819	3	Q	12	1240.5110
481.2405	4	Q	11	1112.4524
568.2726	5	S	10	984.3939
699.3130	6	M	9	897.3618
786.3451	7	S	8	766.3214
873.3771	8	S	7	679.2893
944.4142	9	A	6	592.2573
1059.4412	10	D	5	521.2202
1146.4732	11	S	4	406.1932
1247.5209	12	T	3	319.1612
1375.5794	13	Q	2	218.1135
	14	A	1	90.0550

PXD025214

LDDKDPNFKDQVILLNK

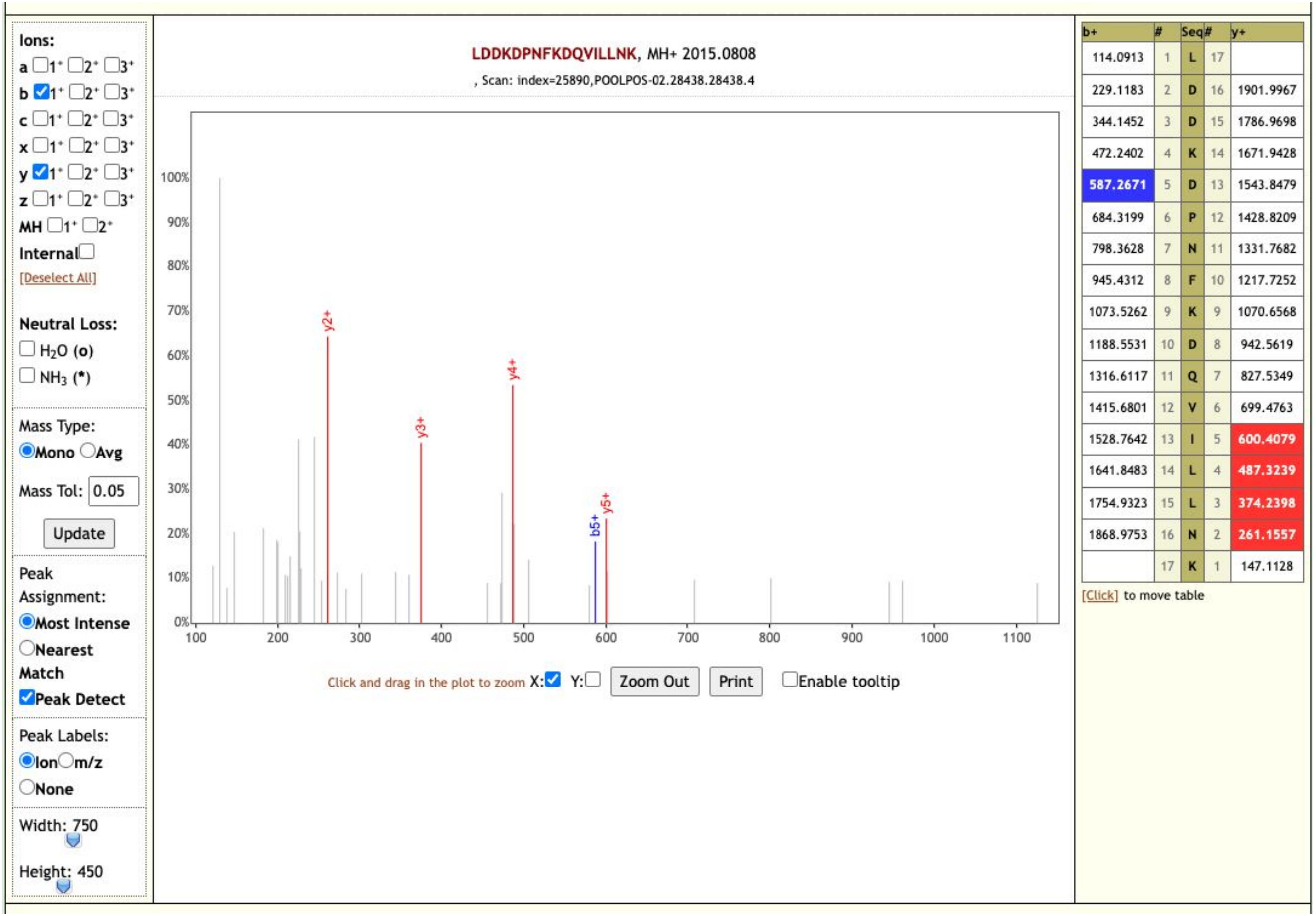

b+	#	Seq	#	y+
114.0913	1	L	17	
229.1183	2	D	16	1901.9967
344.1452	3	D	15	1786.9698
472.2402	4	K	14	1671.9428
587.2671	5	D	13	1543.8479
684.3199	6	P	12	1428.8209
798.3628	7	N	11	1331.7682
945.4312	8	F	10	1217.7252
1073.5262	9	K	9	1070.6568
1188.5531	10	D	8	942.5619
1316.6117	11	Q	7	827.5349
1415.6801	12	V	6	699.4763
1528.7642	13	I	5	600.4079
1641.8483	14	L	4	487.3239
1754.9323	15	L	3	374.2398
1868.9753	16	N	2	261.1557
	17	K	1	147.1128

RPQGLPNNTASWFTALTQHGK

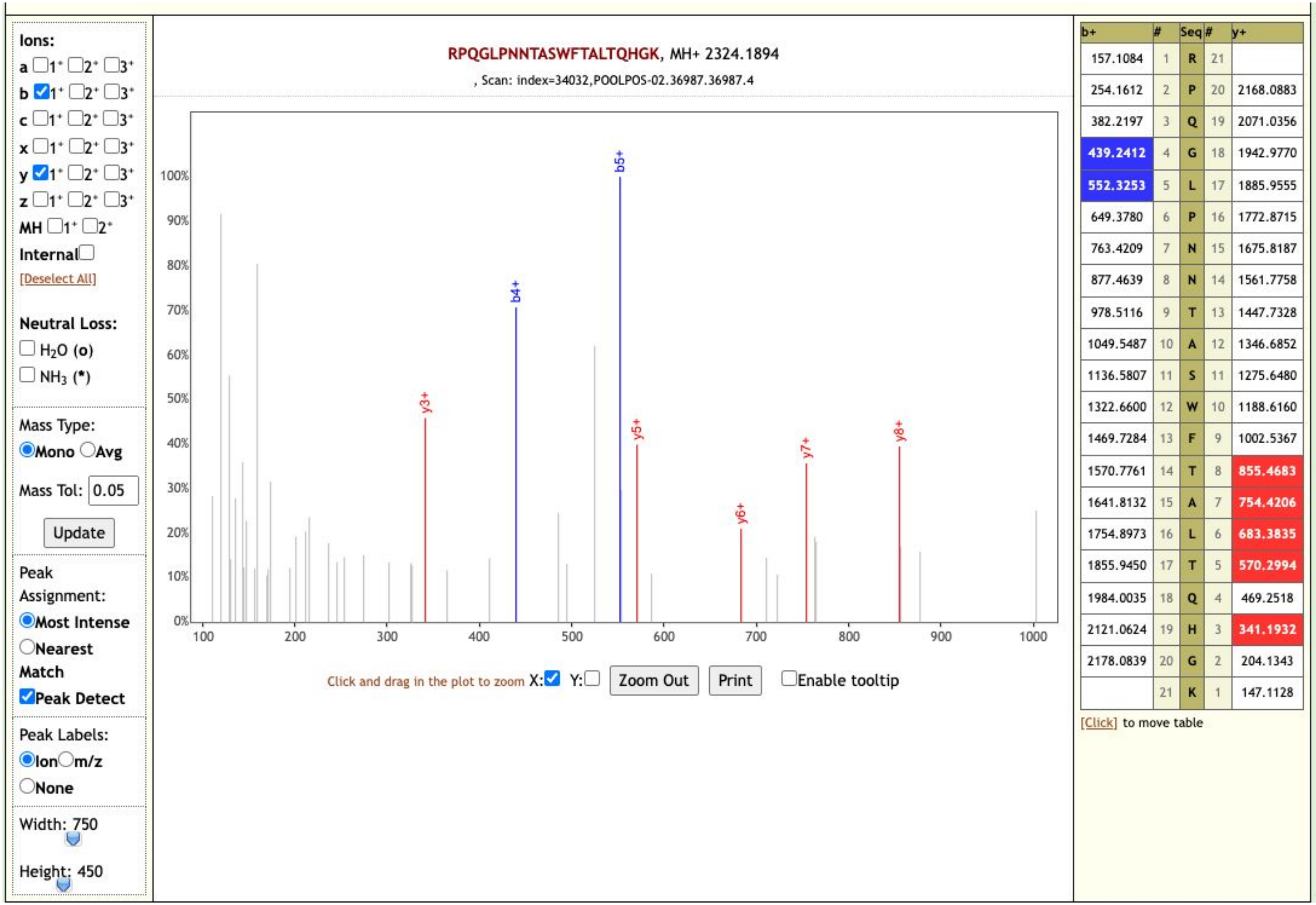

b+	#	Seq	#	y+
157.1084	1	R	21	
254.1612	2	P	20	2168.0883
382.2197	3	Q	19	2071.0356
439.2412	4	G	18	1942.9770
552.3253	5	L	17	1885.9555
649.3780	6	P	16	1772.8715
763.4209	7	N	15	1675.8187
877.4639	8	N	14	1561.7758
978.5116	9	T	13	1447.7328
1049.5487	10	A	12	1346.6852
1136.5807	11	S	11	1275.6480
1322.6600	12	W	10	1188.6160
1469.7284	13	F	9	1002.5367
1570.7761	14	T	8	855.4683
1641.8132	15	A	7	754.4206
1754.8973	16	L	6	683.3835
1855.9450	17	T	5	570.2994
1984.0035	18	Q	4	469.2518
2121.0624	19	H	3	341.1932
2178.0839	20	G	2	204.1343
	21	K	1	147.1128

[Click] to move table

RPQGLPNNTASWFTALTQHGKEDLK

Ions:
a 1+ 2+ 3+
b 1+ 2+ 3+
c 1+ 2+ 3+
x 1+ 2+ 3+
y 1+ 2+ 3+
z 1+ 2+ 3+
MH 1+ 2+
Internal
[Deselect All]

Neutral Loss:
H_2O (o)
NH_3 (*)

Mass Type:
Mono Avg

Mass Tol: 0.05

Update

Peak Assignment:
Most Intense
Nearest Match
Peak Detect

Peak Labels:
Ion m/z
None

Width: 750

Height: 450

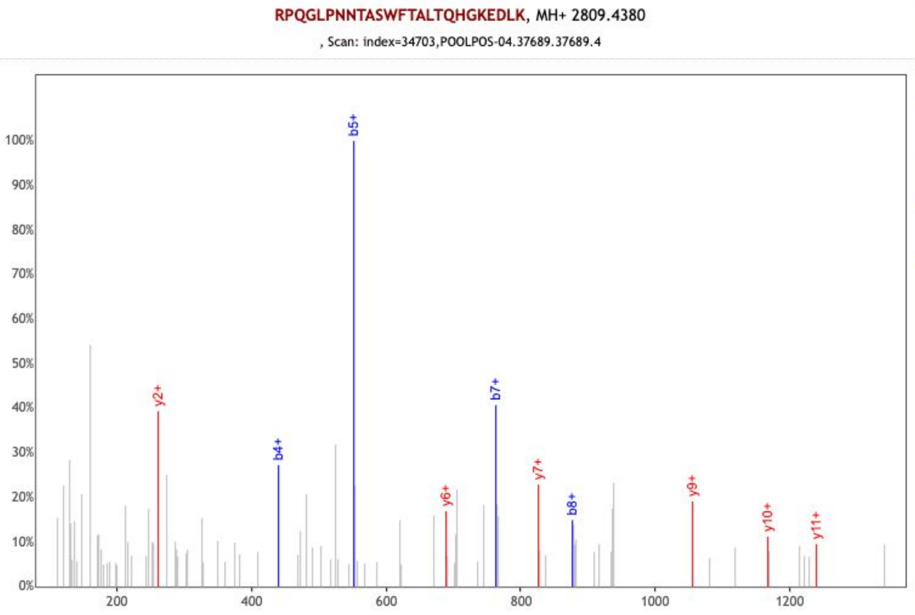

Click and drag in the plot to zoom X: Y: Zoom Out Print Enable tooltip

b+	#	Seq	#	y+
157.1084	1	R	25	
254.1612	2	P	24	2653.3369
382.2197	3	Q	23	2556.2841
439.2412	4	G	22	2428.2255
552.3253	5	L	21	2371.2041
649.3780	6	P	20	2258.1200
763.4209	7	N	19	2161.0673
877.4639	8	N	18	2047.0243
978.5116	9	T	17	1932.9814
1049.5487	10	A	16	1831.9337
1136.5807	11	S	15	1760.8966
1322.6600	12	W	14	1673.8646
1469.7284	13	F	13	1487.7853
1570.7761	14	T	12	1340.7169
1641.8132	15	A	11	1239.6692
1754.8973	16	L	10	1168.6321
1855.9450	17	T	9	1055.5480
1984.0035	18	Q	8	954.5003
2121.0624	19	H	7	826.4417
2178.0839	20	G	6	689.3828
2306.1789	21	K	5	632.3614
2435.2215	22	E	4	504.2664
2550.2484	23	D	3	375.2238
2663.3325	24	L	2	260.1969
	25	K	1	147.1128

[Click] to move table

ITFGGPSDSTGSNQDGER

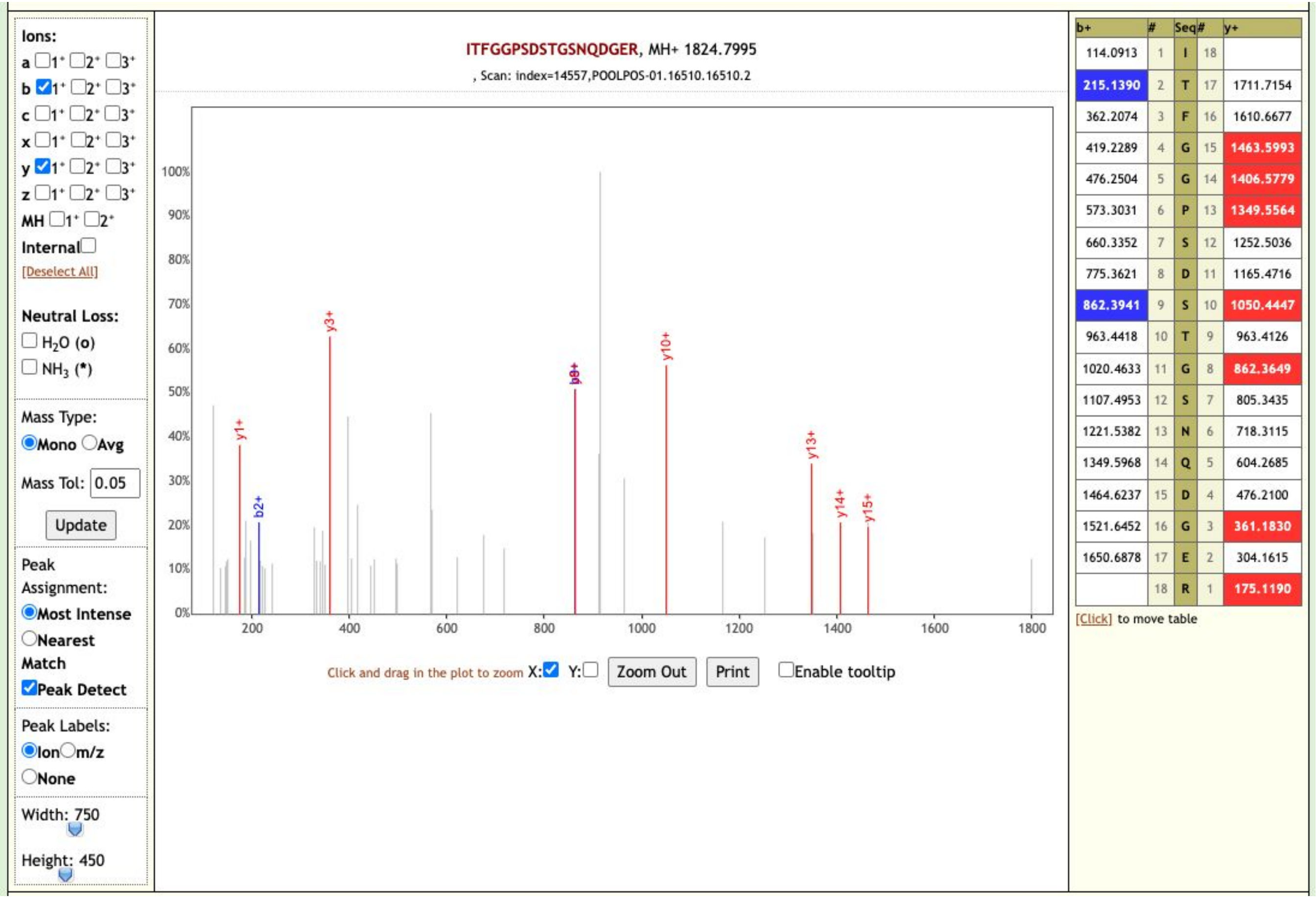

b+	#	Seq	#	y+
114.0913	1	I	18	
215.1390	2	T	17	1711.7154
362.2074	3	F	16	1610.6677
419.2289	4	G	15	1463.5993
476.2504	5	G	14	1406.5779
573.3031	6	P	13	1349.5564
660.3352	7	S	12	1252.5036
775.3621	8	D	11	1165.4716
862.3941	9	S	10	1050.4447
963.4418	10	T	9	963.4126
1020.4633	11	G	8	862.3649
1107.4953	12	S	7	805.3435
1221.5382	13	N	6	718.3115
1349.5968	14	Q	5	604.2685
1464.6237	15	D	4	476.2100
1521.6452	16	G	3	361.1830
1650.6878	17	E	2	304.1615
	18	R	1	175.1190

AYNVTQAFGR

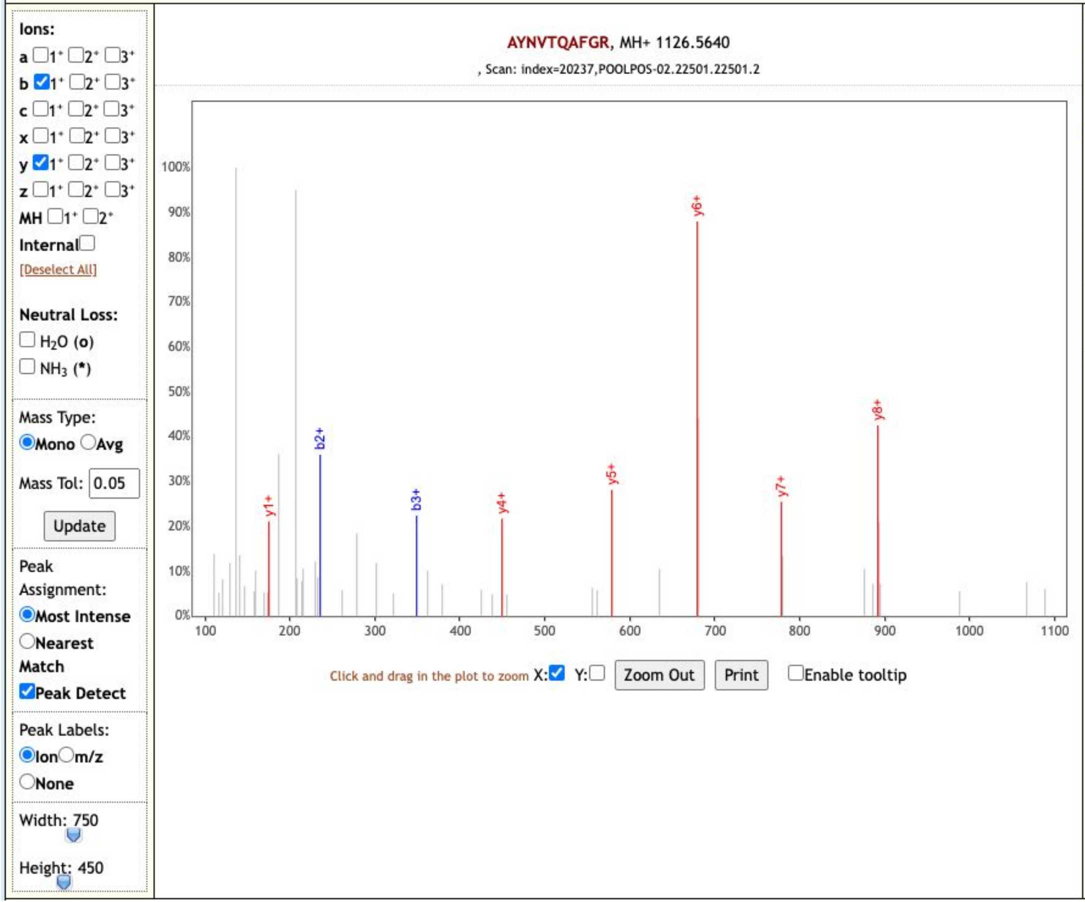

b+	#	Seq	#	y+
72.0444	1	A	10	
235.1077	2	Y	9	1055.5269
349.1506	3	N	8	892.4635
448.2191	4	V	7	778.4206
549.2667	5	T	6	679.3522
677.3253	6	Q	5	578.3045
748.3624	7	A	4	450.2459
895.4308	8	F	3	379.2088
952.4523	9	G	2	232.1404
	10	R	1	175.1190

[Click] to move table

ITFGGPSDSTGSNQNGER

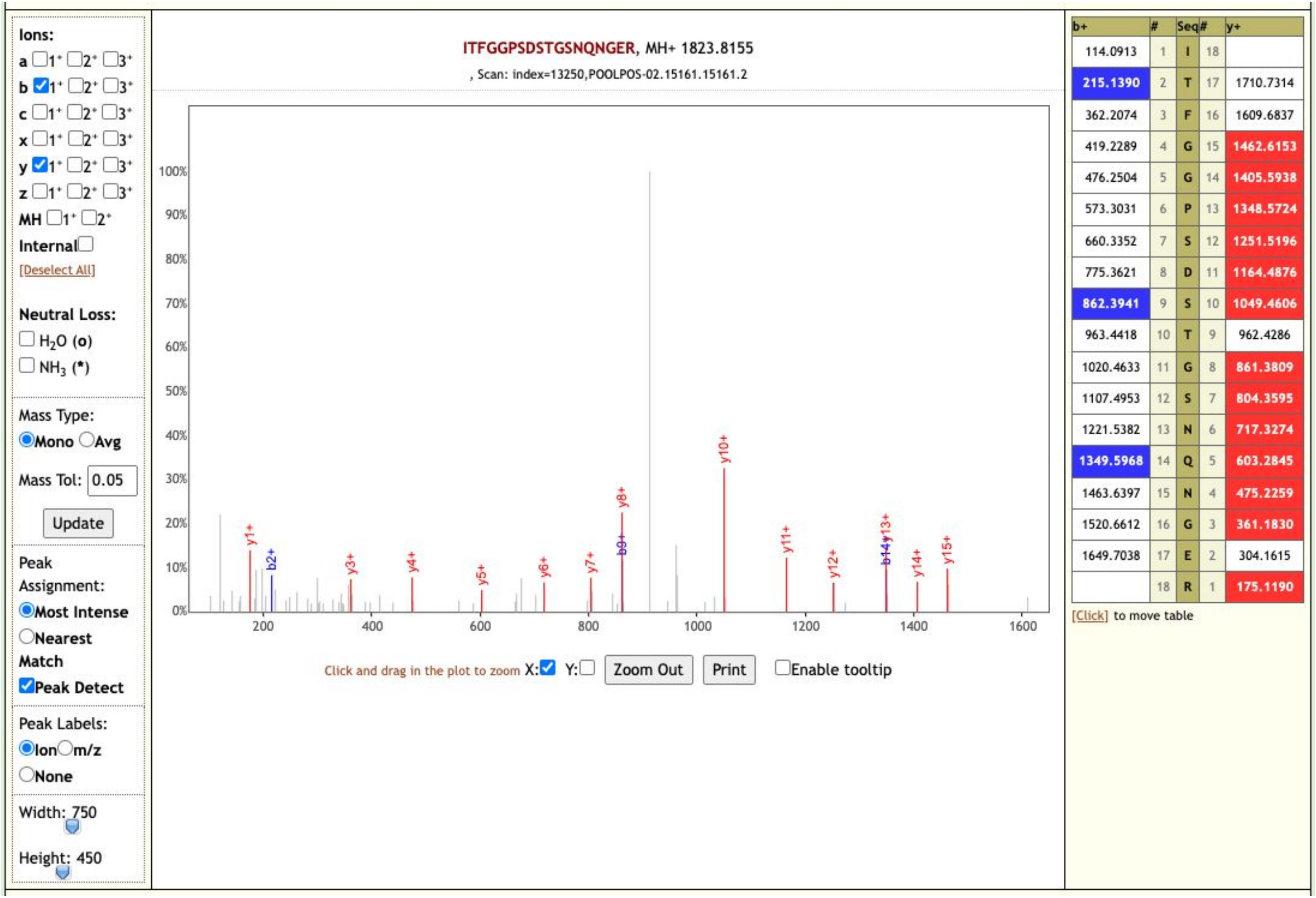

b+	#	Seq	#	y+
114.0913	1	I	18	
215.1390	2	T	17	1710.7314
362.2074	3	F	16	1609.6837
419.2289	4	G	15	1462.6153
476.2504	5	G	14	1405.5938
573.3031	6	P	13	1348.5724
660.3352	7	S	12	1251.5196
775.3621	8	D	11	1164.4876
862.3941	9	S	10	1049.4606
963.4418	10	T	9	962.4286
1020.4633	11	G	8	861.3809
1107.4953	12	S	7	804.3595
1221.5382	13	N	6	717.3274
1349.5968	14	Q	5	603.2845
1463.6397	15	N	4	475.2259
1520.6612	16	G	3	361.1830
1649.7038	17	E	2	304.1615
	18	R	1	175.1190

[Click] to move table

RPQGLPNNTASWF

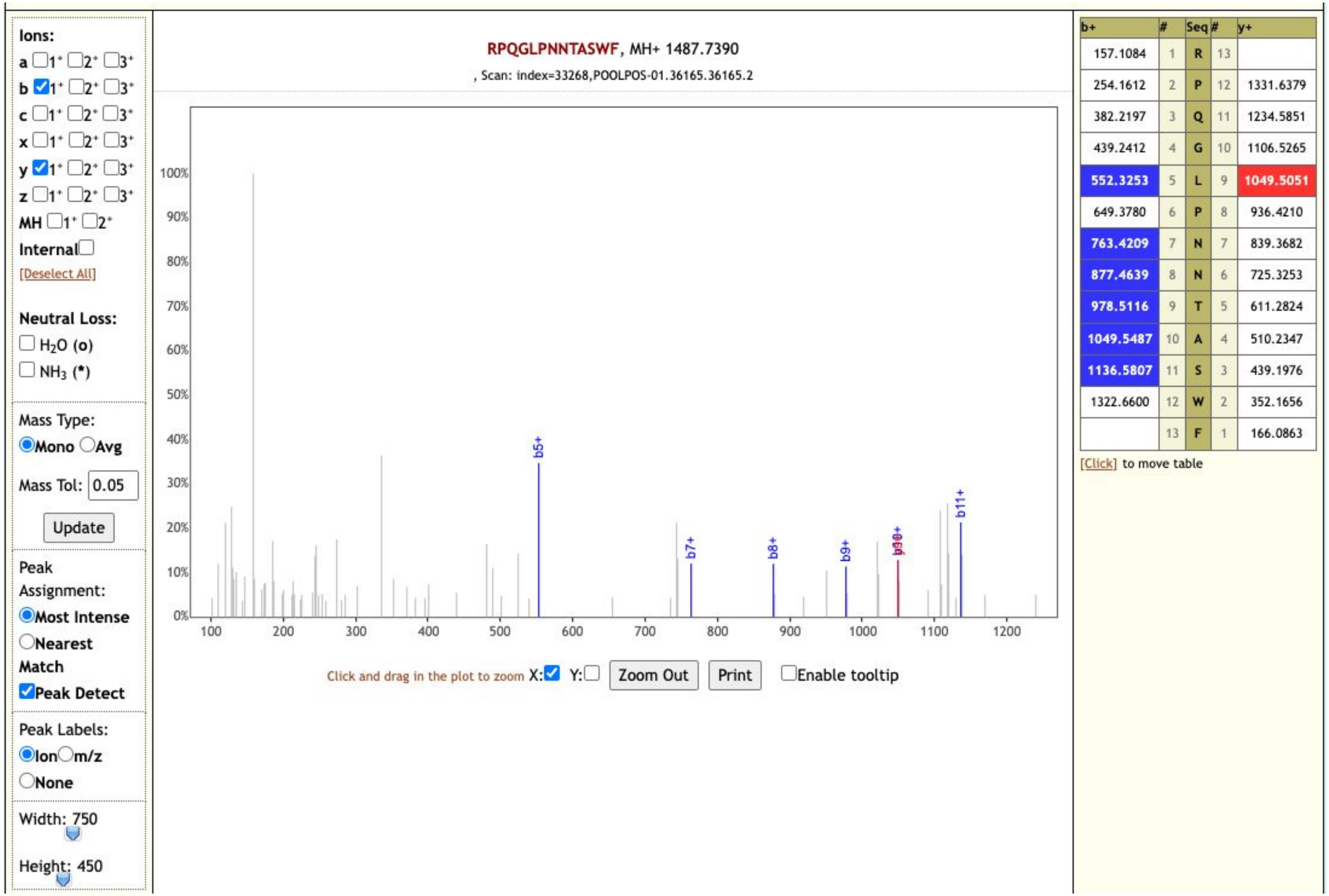

b+	#	Seq	#	y+
157.1084	1	R	13	
254.1612	2	P	12	1331.6379
382.2197	3	Q	11	1234.5851
439.2412	4	G	10	1106.5265
552.3253	5	L	9	1049.5051
649.3780	6	P	8	936.4210
763.4209	7	N	7	839.3682
877.4639	8	N	6	725.3253
978.5116	9	T	5	611.2824
1049.5487	10	A	4	510.2347
1136.5807	11	S	3	439.1976
1322.6600	12	W	2	352.1656
	13	F	1	166.0863

[Click] to move table

HWPQIAQFAPSASAFF

Ions:
a 1+ 2+ 3+
b 1+ 2+ 3+
c 1+ 2+ 3+
x 1+ 2+ 3+
y 1+ 2+ 3+
z 1+ 2+ 3+
MH 1+ 2+
Internal
[Deselect All]

Neutral Loss:
H_2O (o)
NH_3 (*)

Mass Type:
Mono Avg

Mass Tol: 0.05

Update

Peak Assignment:
Most Intense
Nearest Match
Peak Detect

Peak Labels:
Ion m/z
None

Width: 750

Height: 450

HWPQIAQFAPSASAFF, MH+ 1804.8806
, Scan: index=49553,POOLPOS-02.53304.53304.2

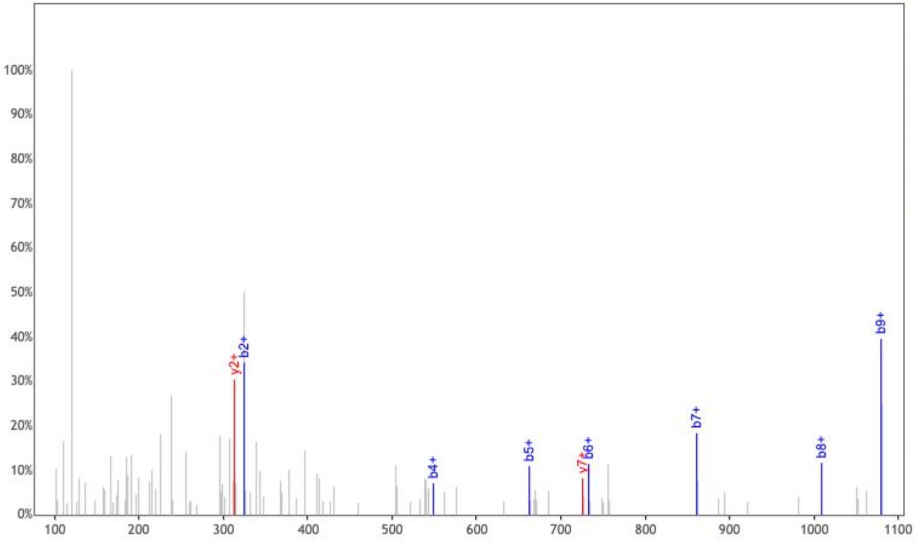

Click and drag in the plot to zoom X: Y: Zoom Out Print Enable tooltip

b+	#	Seq	#	y+
138.0662	1	H	16	
324.1455	2	W	15	1667.8217
421.1983	3	P	14	1481.7423
549.2568	4	Q	13	1384.6896
662.3409	5	I	12	1256.6310
733.3780	6	A	11	1143.5469
861.4366	7	Q	10	1072.5098
1008.5050	8	F	9	944.4512
1079.5421	9	A	8	797.3828
1176.5949	10	P	7	726.3457
1263.6269	11	S	6	629.2930
1334.6640	12	A	5	542.2609
1421.6961	13	S	4	471.2238
1492.7332	14	A	3	384.1918
1639.8016	15	F	2	313.1547
	16	F	1	166.0863

[Click] to move table

MAGNGGDAALALLLLDRLNQLESK

Ions:
a ☐1⁺ ☐2⁺ ☐3⁺
b ☑1⁺ ☐2⁺ ☐3⁺
c ☐1⁺ ☐2⁺ ☐3⁺
x ☐1⁺ ☐2⁺ ☐3⁺
y ☑1⁺ ☐2⁺ ☐3⁺
z ☐1⁺ ☐2⁺ ☐3⁺
MH ☐1⁺ ☐2⁺
Internal ☐
[Deselect All]

Neutral Loss:
☐ H_2O (o)
☐ NH_3 (*)

Mass Type:
◉Mono ○Avg

Mass Tol: 0.05

Update

Peak Assignment:
◉Most Intense
○Nearest Match
☑Peak Detect

Peak Labels:
◉Ion ○m/z
○None

Width: 750

Height: 450

MAGNGGDAALALLLLDRLNQLESK, MH+ 2483.3286

, Scan: index=50511,POOLPOS-04.54308.54308.3

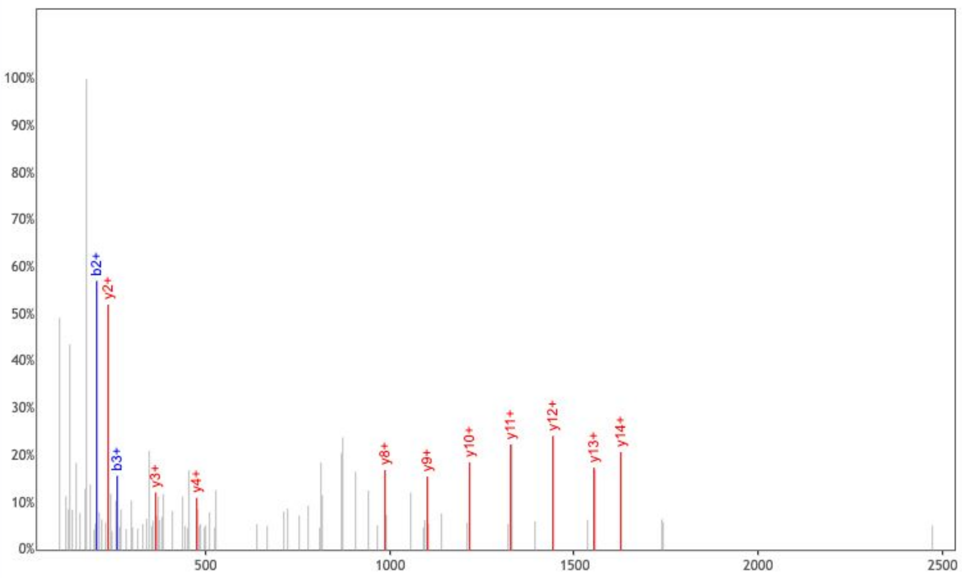

Click and drag in the plot to zoom X:☑ Y:☐ Zoom Out Print ☐Enable tooltip

b+	#	Seq	#	y+
132.0478	1	M	24	
203.0849	2	A	23	2352.2881
260.1063	3	G	22	2281.2510
374.1493	4	N	21	2224.2296
431.1707	5	G	20	2110.1866
488.1922	6	G	19	2053.1652
603.2191	7	D	18	1996.1437
674.2562	8	A	17	1881.1168
745.2934	9	A	16	1810.0797
858.3774	10	L	15	1739.0425
929.4145	11	A	14	1625.9585
1042.4986	12	L	13	1554.9214
1155.5827	13	L	12	1441.8373
1268.6667	14	L	11	1328.7532
1381.7508	15	L	10	1215.6692
1496.7777	16	D	9	1102.5851
1652.8789	17	R	8	987.5582
1765.9629	18	L	7	831.4571
1880.0058	19	N	6	718.3730
2008.0644	20	Q	5	604.3301
2121.1485	21	L	4	476.2715
2250.1911	22	E	3	363.1874
2337.2231	23	S	2	234.1448
	24	K	1	147.1128

[Click] to move table

GQGVPINTNSSR

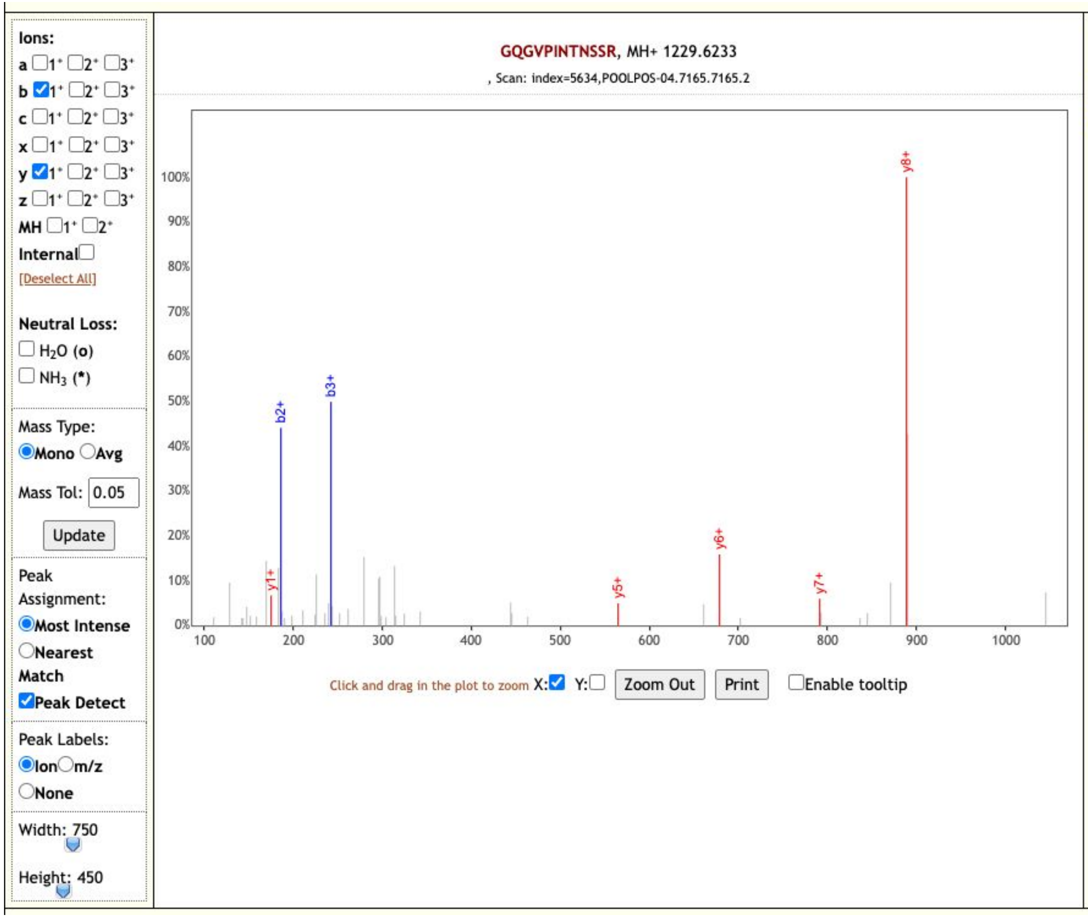

b+	#	Seq	#	y+
58.0287	1	G	12	
186.0873	2	Q	11	1172.6018
243.1088	3	G	10	1044.5432
342.1772	4	V	9	987.5218
439.2300	5	P	8	888.4534
552.3140	6	I	7	791.4006
666.3570	7	N	6	678.3165
767.4046	8	T	5	564.2736
881.4476	9	N	4	463.2259
968.4796	10	S	3	349.1830
1055.5116	11	S	2	262.1510
	12	R	1	175.1190

[Click] to move table

QQTVTLLPAADLDDFSK

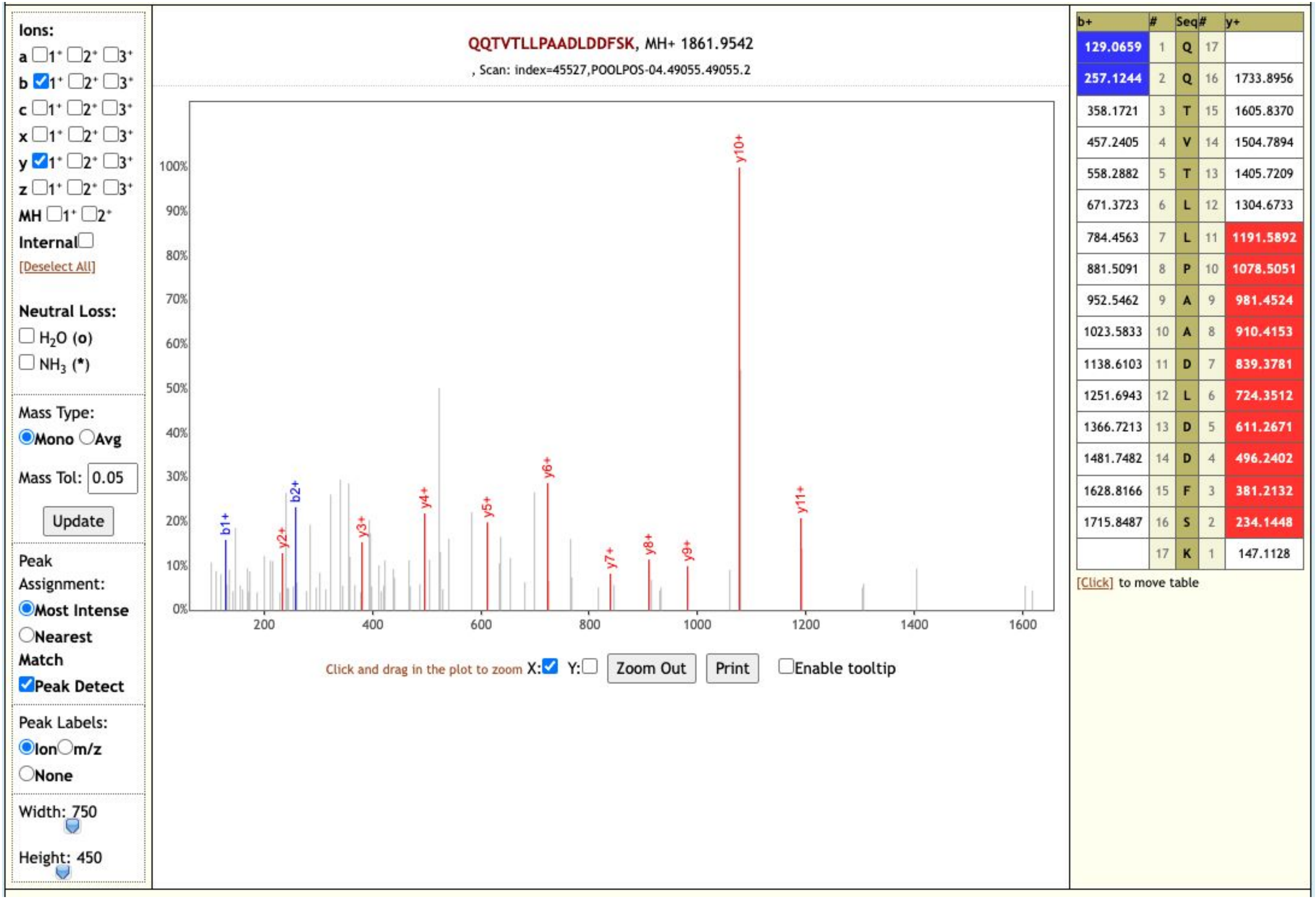

b+	#	Seq	#	y+
129.0659	1	Q	17	
257.1244	2	Q	16	1733.8956
358.1721	3	T	15	1605.8370
457.2405	4	V	14	1504.7894
558.2882	5	T	13	1405.7209
671.3723	6	L	12	1304.6733
784.4563	7	L	11	1191.5892
881.5091	8	P	10	1078.5051
952.5462	9	A	9	981.4524
1023.5833	10	A	8	910.4153
1138.6103	11	D	7	839.3781
1251.6943	12	L	6	724.3512
1366.7213	13	D	5	611.2671
1481.7482	14	D	4	496.2402
1628.8166	15	F	3	381.2132
1715.8487	16	S	2	234.1448
	17	K	1	147.1128

[Click] to move table

TALTQHGKEDLKFPR

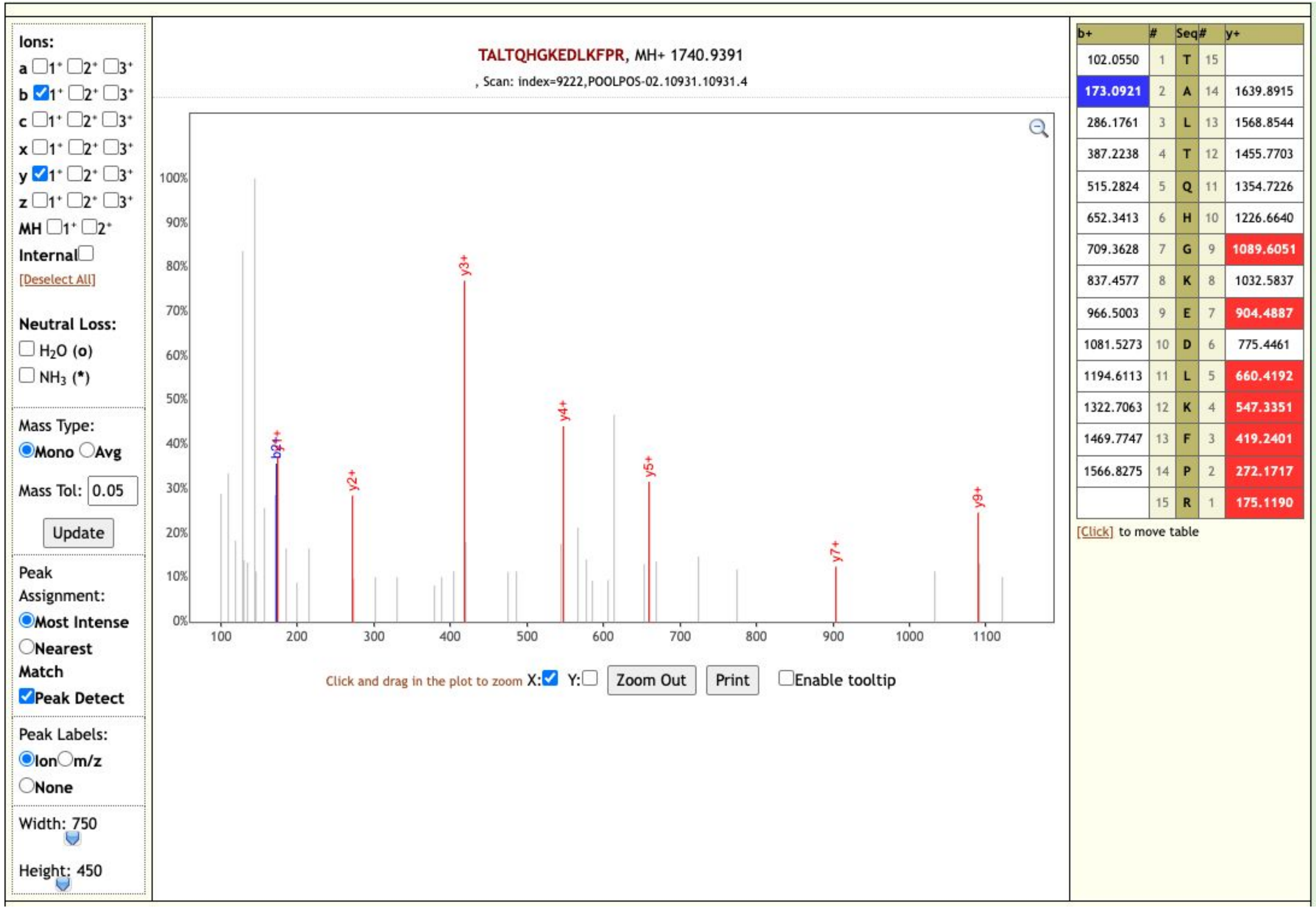

b+	#	Seq	#	y+
102.0550	1	T	15	
173.0921	2	A	14	1639.8915
286.1761	3	L	13	1568.8544
387.2238	4	T	12	1455.7703
515.2824	5	Q	11	1354.7226
652.3413	6	H	10	1226.6640
709.3628	7	G	9	1089.6051
837.4577	8	K	8	1032.5837
966.5003	9	E	7	904.4887
1081.5273	10	D	6	775.4461
1194.6113	11	L	5	660.4192
1322.7063	12	K	4	547.3351
1469.7747	13	F	3	419.2401
1566.8275	14	P	2	272.1717
	15	R	1	175.1190

[Click] to move table

DGIIWVATEGALNTPK

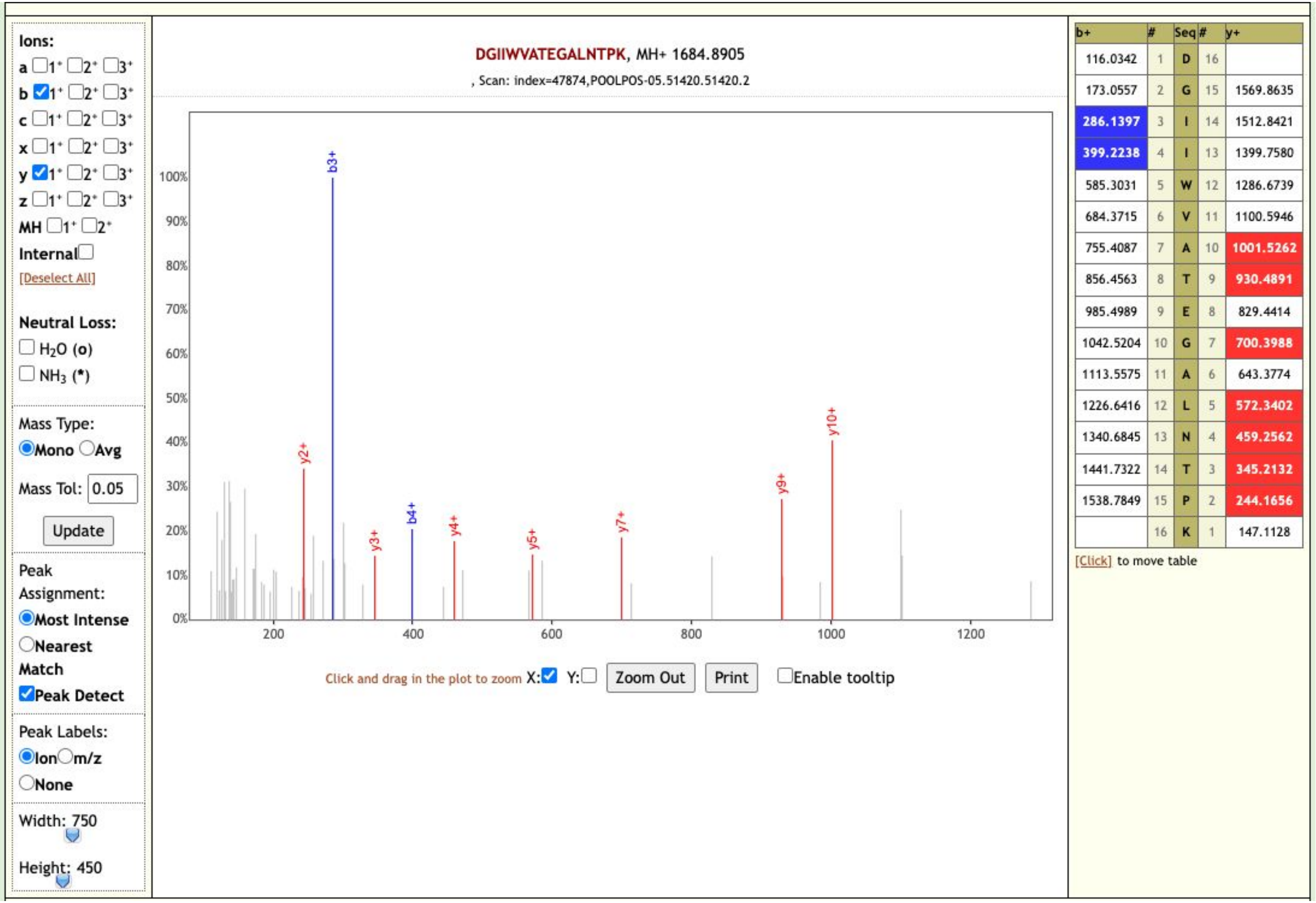

b+	#	Seq	#	y+
116.0342	1	D	16	
173.0557	2	G	15	1569.8635
286.1397	3	I	14	1512.8421
399.2238	4	I	13	1399.7580
585.3031	5	W	12	1286.6739
684.3715	6	V	11	1100.5946
755.4087	7	A	10	1001.5262
856.4563	8	T	9	930.4891
985.4989	9	E	8	829.4414
1042.5204	10	G	7	700.3988
1113.5575	11	A	6	643.3774
1226.6416	12	L	5	572.3402
1340.6845	13	N	4	459.2562
1441.7322	14	T	3	345.2132
1538.7849	15	P	2	244.1656
	16	K	1	147.1128

[Click] to move table

GFYAEGSR

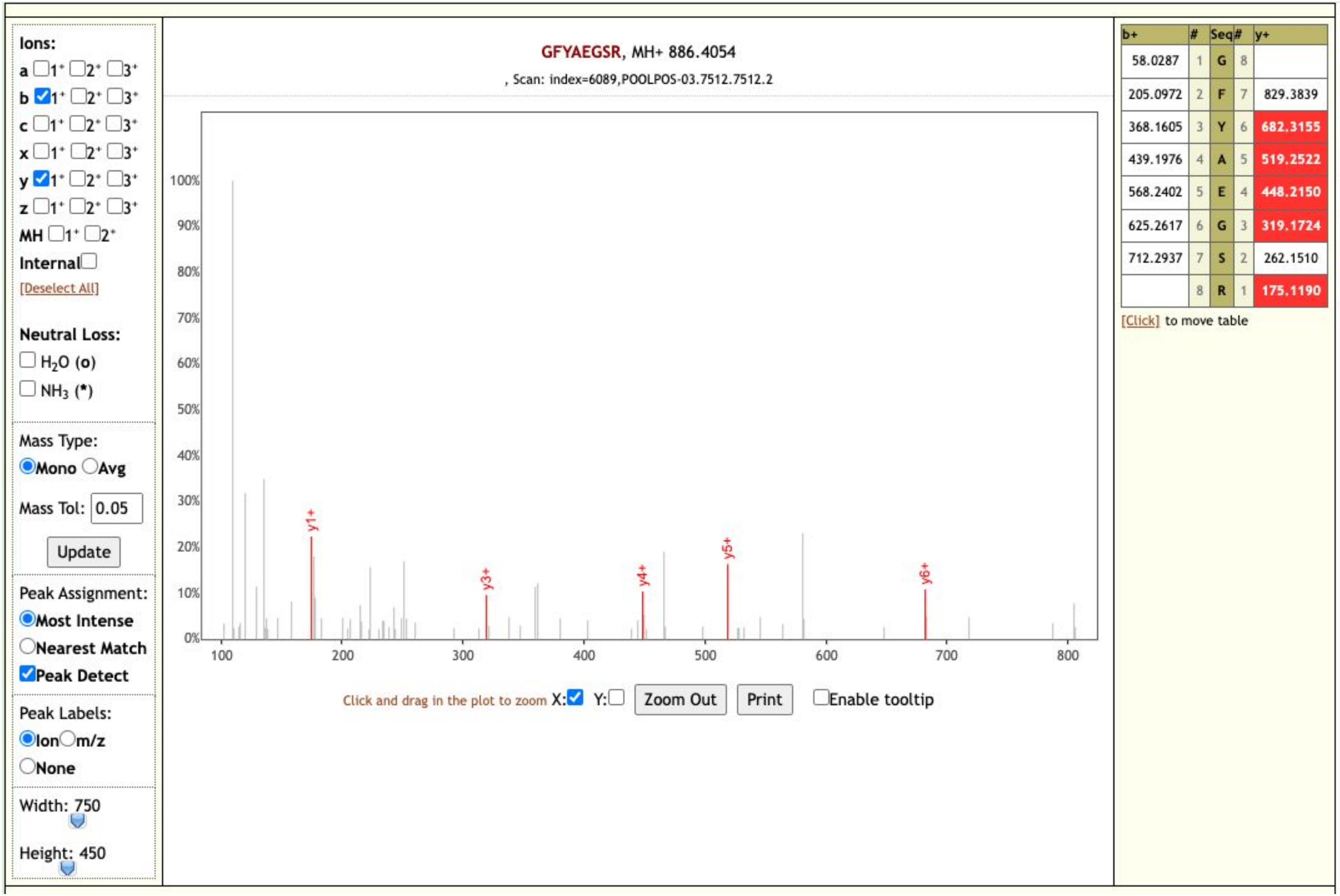
Ions:
a 1+ 2+ 3+
b 1+ 2+ 3+
c 1+ 2+ 3+
x 1+ 2+ 3+
y 1+ 2+ 3+
z 1+ 2+ 3+
MH 1+ 2+
Internal
[Deselect All]
Neutral Loss:
H2O (o)
NH3 (*)
Mass Type:
Mono Avg
Mass Tol: 0.05
Update
Peak Assignment:
Most Intense
Nearest Match
Peak Detect
Peak Labels:
Ion m/z
None
Width: 750
Height: 450
GFYAEGSR, MH+ 886.4054
, Scan: index=6089,POOLPOS-03.7512.7512.2
100%
90%
80%
70%
60%
50%
40%
30%
20%
10%
0%
y1+
y3+
y4+
y5+
y6+
100
200
300
400
500
600
700
800
Click and drag in the plot to zoom X: Y:
Zoom Out
Print
Enable tooltip
b+ # Seq # y+
58.0287 1 G 8
205.0972 2 F 7 829.3839
368.1605 3 Y 6 682.3155
439.1976 4 A 5 519.2522
568.2402 5 E 4 448.2150
625.2617 6 G 3 319.1724
712.2937 7 S 2 262.1510
8 R 1 175.1190
[Click] to move table

GPEQTQGNFGDQELIR

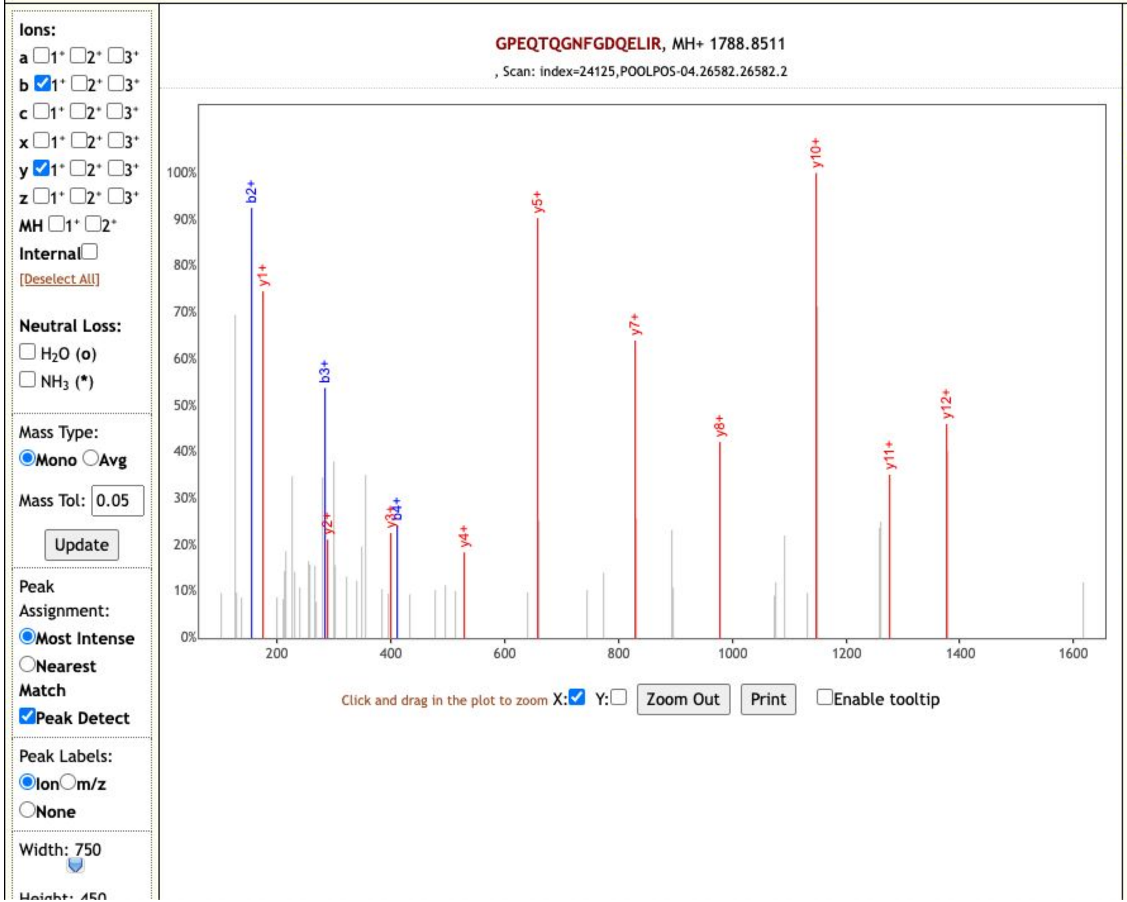

b+	#	Seq	#	y+
58.0287	1	G	16	
155.0815	2	P	15	1731.8297
284.1241	3	E	14	1634.7769
412.1827	4	Q	13	1505.7343
513.2304	5	T	12	1377.6757
641.2889	6	Q	11	1276.6280
698.3104	7	G	10	1148.5695
812.3533	8	N	9	1091.5480
959.4217	9	F	8	977.5051
1016.4432	10	G	7	830.4367
1131.4701	11	D	6	773.4152
1259.5287	12	Q	5	658.3883
1388.5713	13	E	4	530.3297
1501.6554	14	L	3	401.2871
1614.7394	15	I	2	288.2030
	16	R	1	175.1190

[Click] to move table

GPEQTQGNFGDQELIR

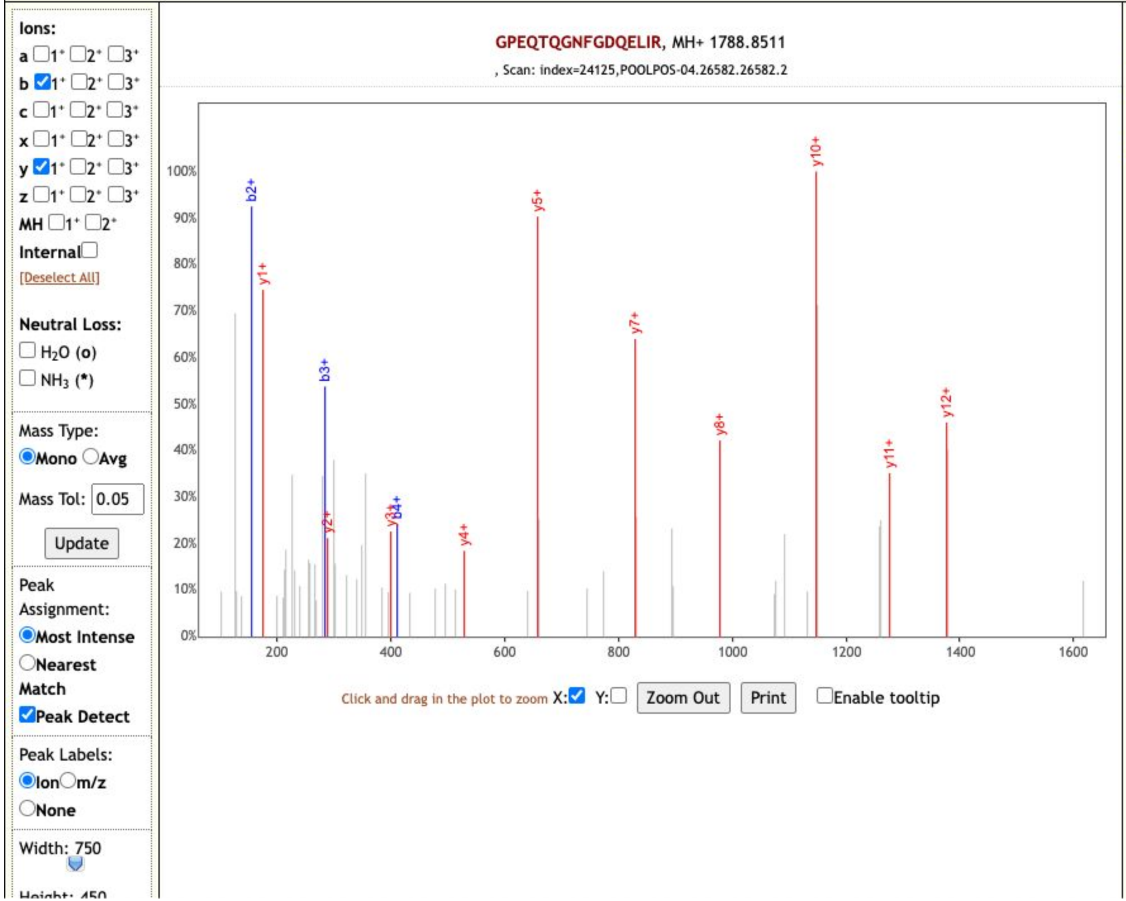

b+	#	Seq	#	y+
58.0287	1	G	16	
155.0815	2	P	15	1731.8297
284.1241	3	E	14	1634.7769
412.1827	4	Q	13	1505.7343
513.2304	5	T	12	1377.6757
641.2889	6	Q	11	1276.6280
698.3104	7	G	10	1148.5695
812.3533	8	N	9	1091.5480
959.4217	9	F	8	977.5051
1016.4432	10	G	7	830.4367
1131.4701	11	D	6	773.4152
1259.5287	12	Q	5	658.3883
1388.5713	13	E	4	530.3297
1501.6554	14	L	3	401.2871
1614.7394	15	I	2	288.2030
	16	R	1	175.1190

[Click] to move table

IGMEVTPSGTWLTYTGAIK

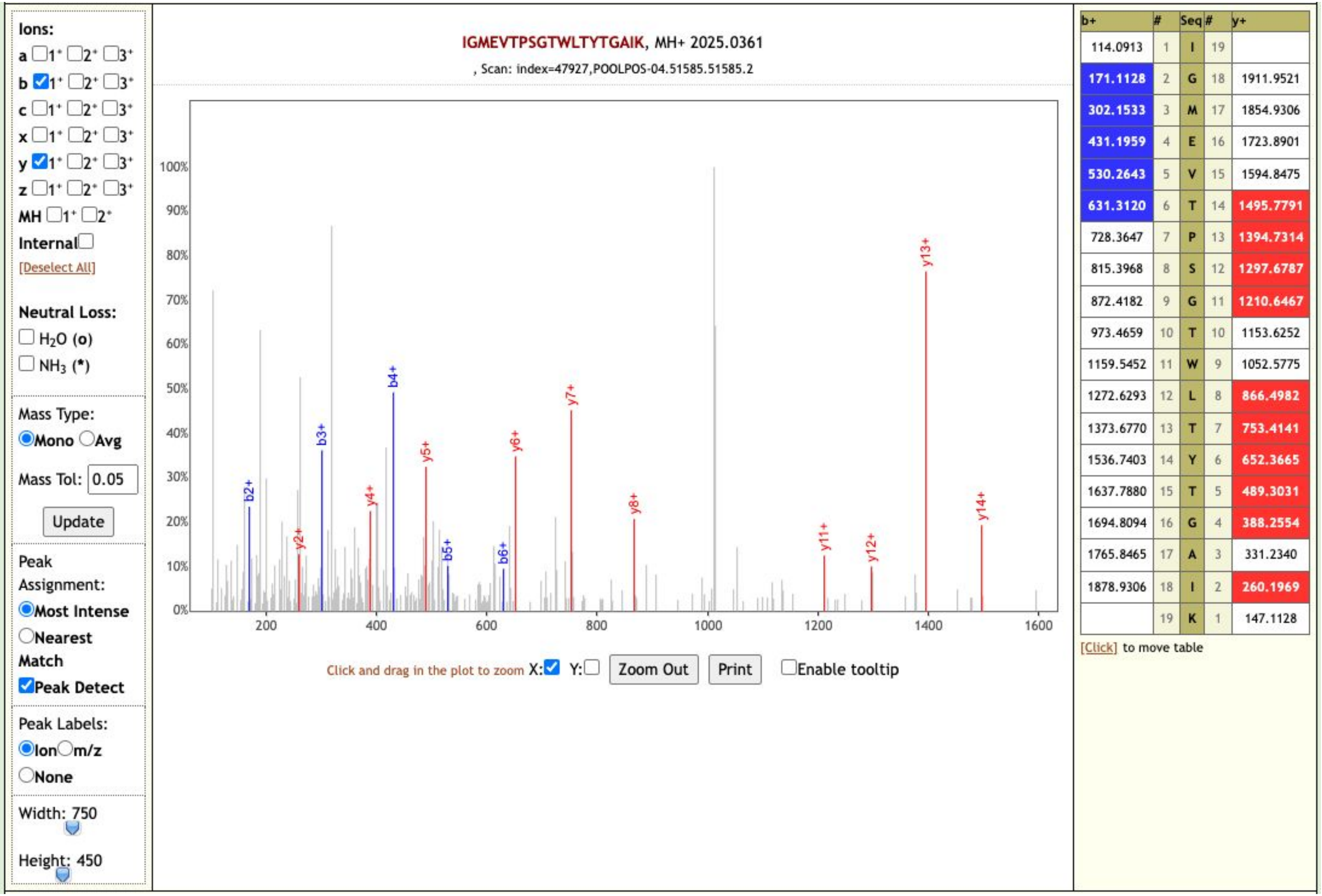

b+	#	Seq	#	y+
114.0913	1	I	19	
171.1128	2	G	18	1911.9521
302.1533	3	M	17	1854.9306
431.1959	4	E	16	1723.8901
530.2643	5	V	15	1594.8475
631.3120	6	T	14	1495.7791
728.3647	7	P	13	1394.7314
815.3968	8	S	12	1297.6787
872.4182	9	G	11	1210.6467
973.4659	10	T	10	1153.6252
1159.5452	11	W	9	1052.5775
1272.6293	12	L	8	866.4982
1373.6770	13	T	7	753.4141
1536.7403	14	Y	6	652.3665
1637.7880	15	T	5	489.3031
1694.8094	16	G	4	388.2554
1765.8465	17	A	3	331.2340
1878.9306	18	I	2	260.1969
	19	K	1	147.1128

[Click] to move table

MAGNGGDAALALLLLDRLNQLESK

Ions:
a ☐1⁺ ☐2⁺ ☐3⁺
b ☑1⁺ ☐2⁺ ☐3⁺
c ☐1⁺ ☐2⁺ ☐3⁺
x ☐1⁺ ☐2⁺ ☐3⁺
y ☑1⁺ ☐2⁺ ☐3⁺
z ☐1⁺ ☐2⁺ ☐3⁺
MH ☐1⁺ ☐2⁺
Internal ☐
[Deselect All]

Neutral Loss:
☐ H_2O (o)
☐ NH_3 (*)

Mass Type:
◉Mono ○Avg

Mass Tol: 0.05

Update

Peak Assignment:
◉Most Intense
○Nearest Match
☑Peak Detect

Peak Labels:
◉Ion ○m/z
○None

Width: 750

Height: 450

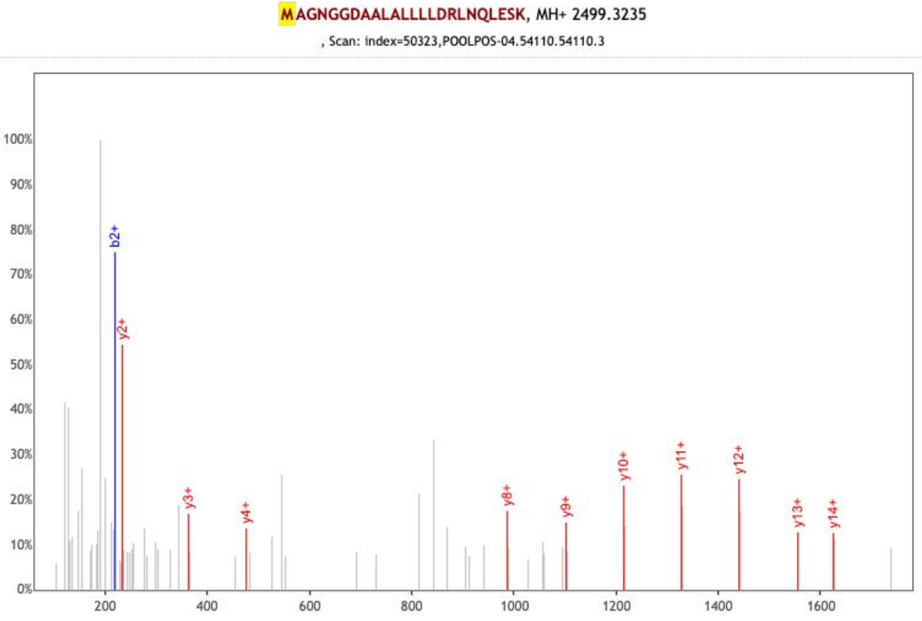

Click and drag in the plot to zoom X:☑ Y:☐ Zoom Out Print ☐Enable tooltip

b+	#	Seq	#	y+
148.0427	1	M	24	
219.0798	2	A	23	2352.2881
276.1013	3	G	22	2281.2510
390.1442	4	N	21	2224.2296
447.1656	5	G	20	2110.1866
504.1871	6	G	19	2053.1652
619.2141	7	D	18	1996.1437
690.2512	8	A	17	1881.1168
761.2883	9	A	16	1810.0797
874.3723	10	L	15	1739.0425
945.4095	11	A	14	1625.9585
1058.4935	12	L	13	1554.9214
1171.5776	13	L	12	1441.8373
1284.6616	14	L	11	1328.7532
1397.7457	15	L	10	1215.6692
1512.7727	16	D	9	1102.5851
1668.8738	17	R	8	987.5582
1781.9578	18	L	7	831.4571
1896.0008	19	N	6	718.3730
2024.0593	20	Q	5	604.3301
2137.1434	21	L	4	476.2715
2266.1860	22	E	3	363.1874
2353.2180	23	S	2	234.1448
	24	K	1	147.1128

[Click] to move table

Variable Modifications:

M: 15.994915 [1]

DRLNQLESK

Ions:
a ☐1+ ☐2+ ☐3+
b ☑1+ ☐2+ ☐3+
c ☐1+ ☐2+ ☐3+
x ☐1+ ☐2+ ☐3+
y ☑1+ ☐2+ ☐3+
z ☐1+ ☐2+ ☐3+
MH ☐1+ ☐2+
Internal ☐
[Deselect All]

Neutral Loss:
☐ H_2O (o)
☐ NH_3 (*)

Mass Type:
◉Mono ○Avg

Mass Tol: 0.05

Update

Peak Assignment:
◉Most Intense
○Nearest Match
☑Peak Detect

Peak Labels:
◉Ion ○m/z
○None

Width: 750

Height: 450

DRLNQLESK, MH+ 1102.5851

, Scan: index=4698,POOLPOS-04.6182.6182.2

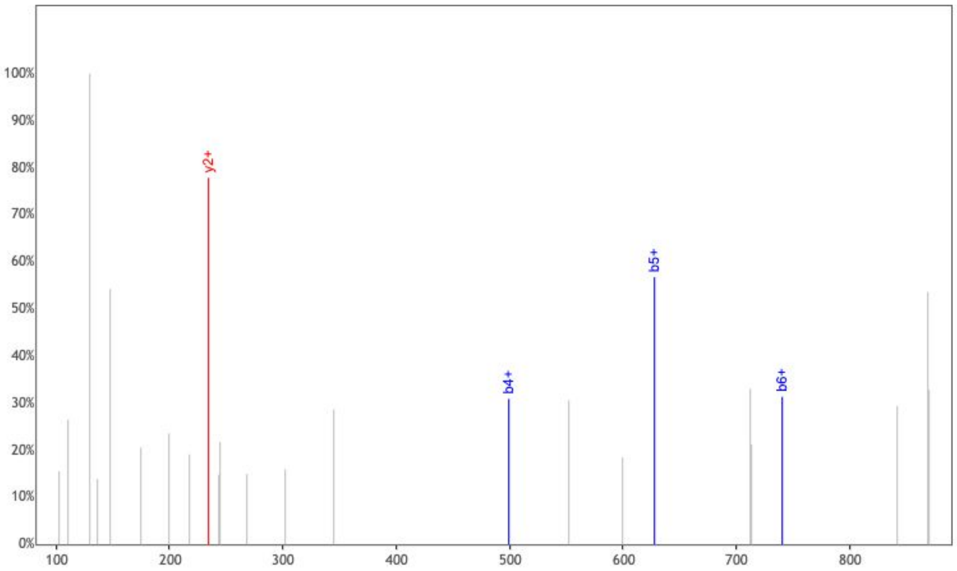

Click and drag in the plot to zoom X:☑ Y:☐ Zoom Out Print ☐Enable tooltip

b+	#	Seq	#	y+
116.0342	1	D	9	
272.1353	2	R	8	987.5582
385.2194	3	L	7	831.4571
499.2623	4	N	6	718.3730
627.3209	5	Q	5	604.3301
740.4050	6	L	4	476.2715
869.4476	7	E	3	363.1874
956.4796	8	S	2	234.1448
	9	K	1	147.1128

[Click] to move table

GQGVPINTNSSPDDQIGYYR

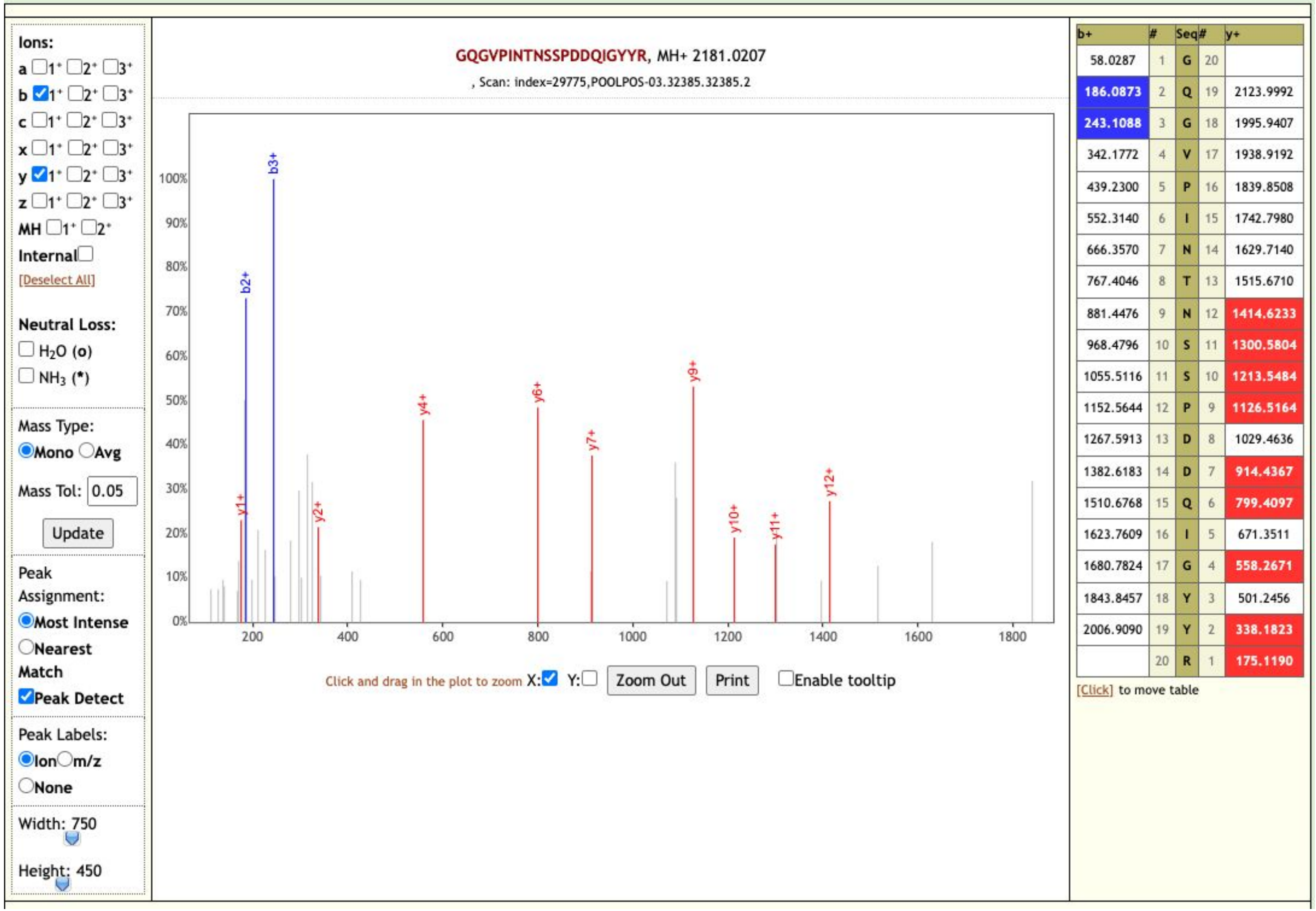

b+	#	Seq	#	y+
58.0287	1	G	20	
186.0873	2	Q	19	2123.9992
243.1088	3	G	18	1995.9407
342.1772	4	V	17	1938.9192
439.2300	5	P	16	1839.8508
552.3140	6	I	15	1742.7980
666.3570	7	N	14	1629.7140
767.4046	8	T	13	1515.6710
881.4476	9	N	12	1414.6233
968.4796	10	S	11	1300.5804
1055.5116	11	S	10	1213.5484
1152.5644	12	P	9	1126.5164
1267.5913	13	D	8	1029.4636
1382.6183	14	D	7	914.4367
1510.6768	15	Q	6	799.4097
1623.7609	16	I	5	671.3511
1680.7824	17	G	4	558.2671
1843.8457	18	Y	3	501.2456
2006.9090	19	Y	2	338.1823
	20	R	1	175.1190

[Click] to move table

KQQTVTLLPAADLDDFSK

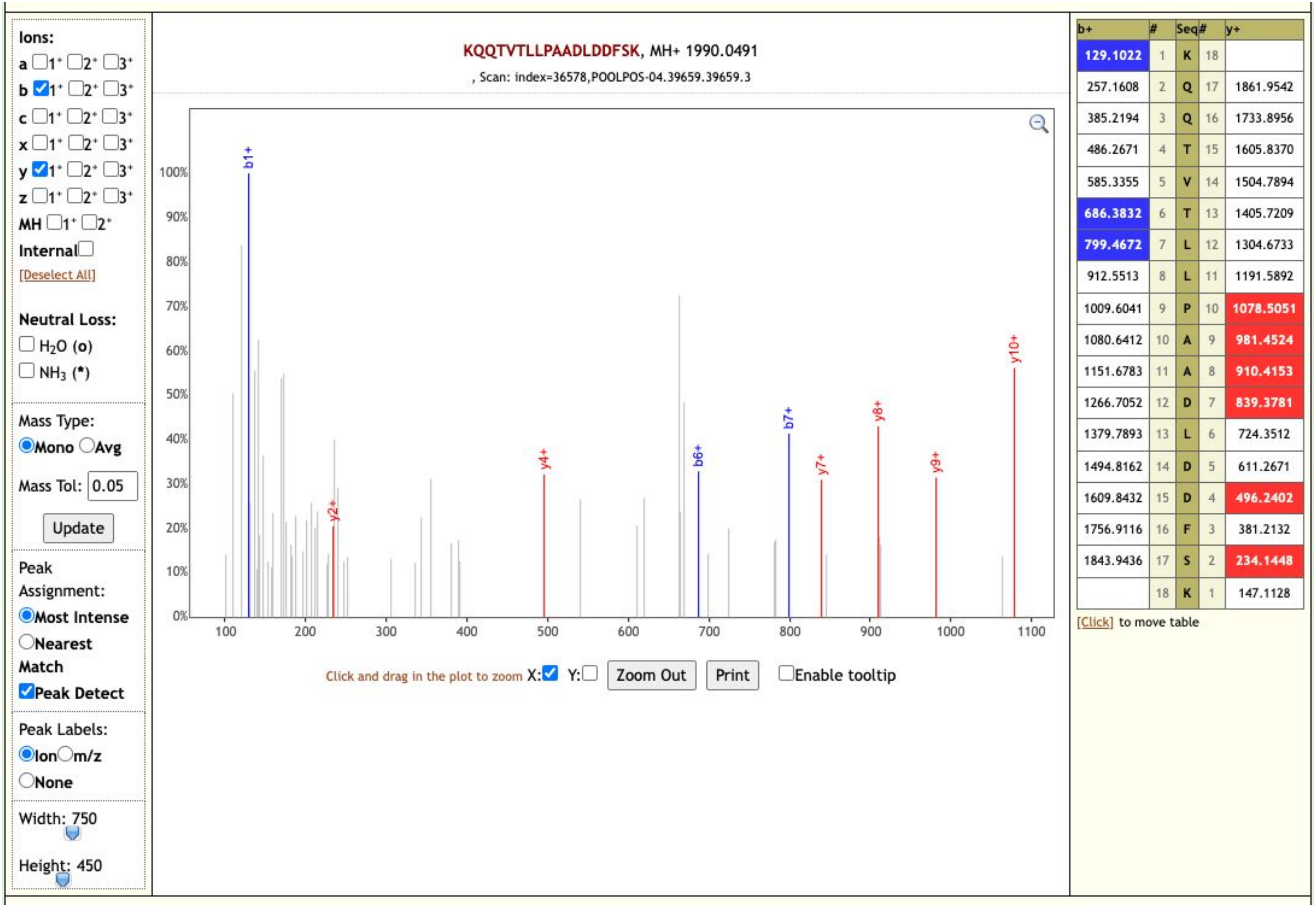

b+	#	Seq	#	y+
129.1022	1	K	18	
257.1608	2	Q	17	1861.9542
385.2194	3	Q	16	1733.8956
486.2671	4	T	15	1605.8370
585.3355	5	V	14	1504.7894
686.3832	6	T	13	1405.7209
799.4672	7	L	12	1304.6733
912.5513	8	L	11	1191.5892
1009.6041	9	P	10	1078.5051
1080.6412	10	A	9	981.4524
1151.6783	11	A	8	910.4153
1266.7052	12	D	7	839.3781
1379.7893	13	L	6	724.3512
1494.8162	14	D	5	611.2671
1609.8432	15	D	4	496.2402
1756.9116	16	F	3	381.2132
1843.9436	17	S	2	234.1448
	18	K	1	147.1128

[Click] to move table

RGPEQTQGNFGDQELTR

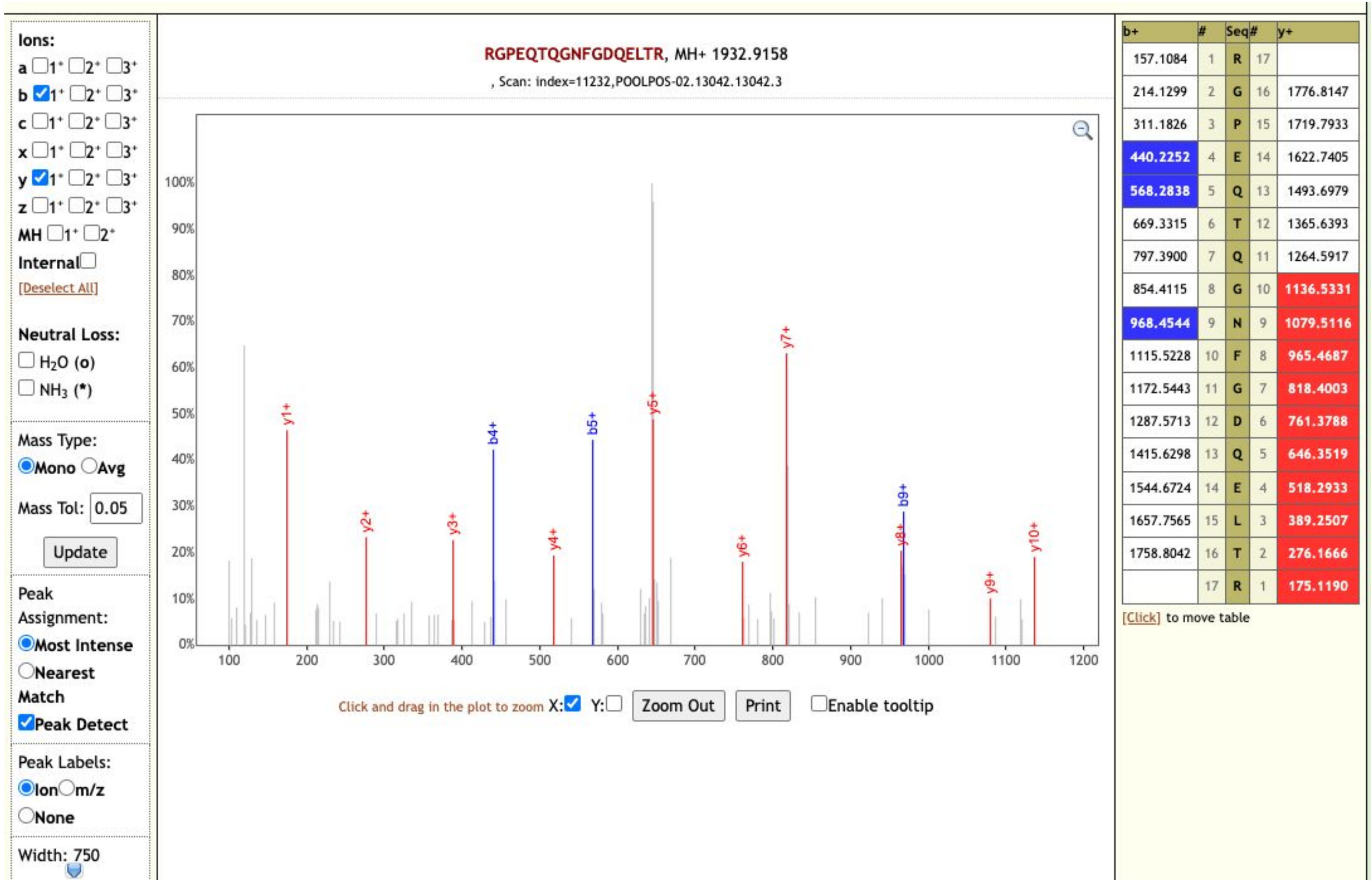

b+	#	Seq	#	y+
157.1084	1	R	17	
214.1299	2	G	16	1776.8147
311.1826	3	P	15	1719.7933
440.2252	4	E	14	1622.7405
568.2838	5	Q	13	1493.6979
669.3315	6	T	12	1365.6393
797.3900	7	Q	11	1264.5917
854.4115	8	G	10	1136.5331
968.4544	9	N	9	1079.5116
1115.5228	10	F	8	965.4687
1172.5443	11	G	7	818.4003
1287.5713	12	D	6	761.3788
1415.6298	13	Q	5	646.3519
1544.6724	14	E	4	518.2933
1657.7565	15	L	3	389.2507
1758.8042	16	T	2	276.1666
	17	R	1	175.1190

[Click] to move table

PGNGCDAALALLLLDR

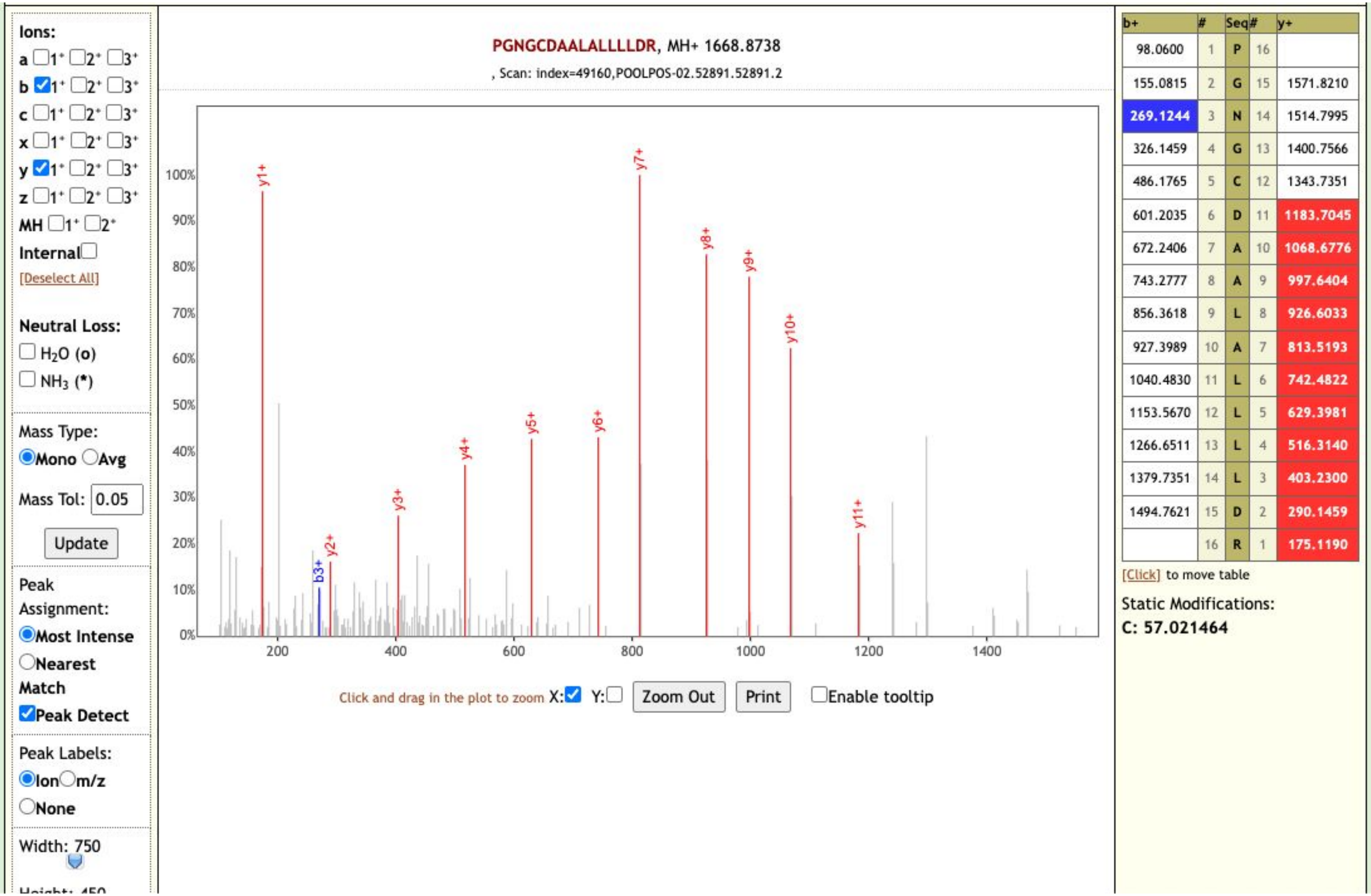

b+	#	Seq	#	y+
98.0600	1	P	16	
155.0815	2	G	15	1571.8210
269.1244	3	N	14	1514.7995
326.1459	4	G	13	1400.7566
486.1765	5	C	12	1343.7351
601.2035	6	D	11	1183.7045
672.2406	7	A	10	1068.6776
743.2777	8	A	9	997.6404
856.3618	9	L	8	926.6033
927.3989	10	A	7	813.5193
1040.4830	11	L	6	742.4822
1153.5670	12	L	5	629.3981
1266.6511	13	L	4	516.3140
1379.7351	14	L	3	403.2300
1494.7621	15	D	2	290.1459
	16	R	1	175.1190

[Click] to move table

Static Modifications:

C: 57.021464

QQTVTLLPAADLDDFSK

Ions:
a 1+ 2+ 3+
b 1+ 2+ 3+
c 1+ 2+ 3+
x 1+ 2+ 3+
y 1+ 2+ 3+
z 1+ 2+ 3+
MH 1+ 2+
Internal
[Deselect All]

Neutral Loss:
H_2O (o)
NH_3 (*)

Mass Type:
Mono Avg

Mass Tol: 0.05

Update

Peak Assignment:
Most Intense
Nearest Match
Peak Detect

Peak Labels:
Ion m/z
None

Width: 750

Height: 450

QQTVTLLPAADLDDFSK, MH+ 1844.9276

, Scan: index=51983,POOLPOS-03.55711.55711.2

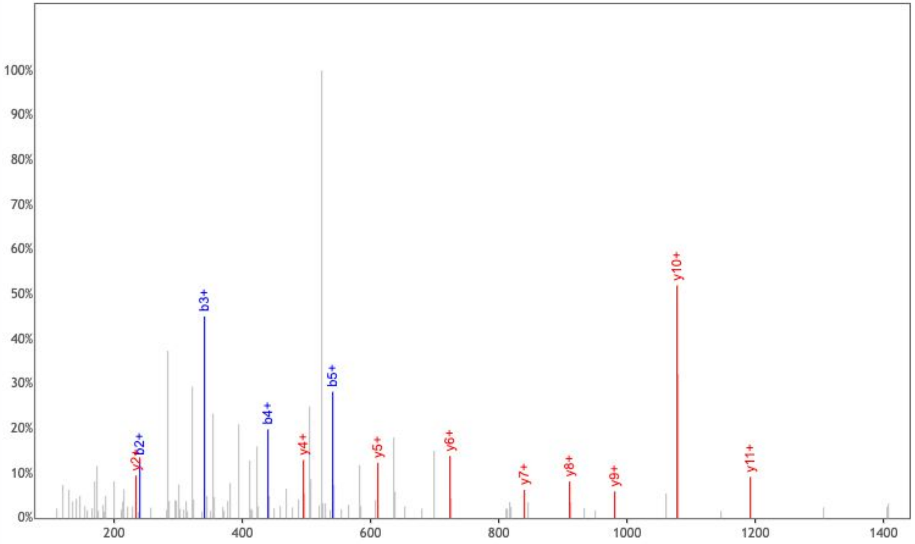

Click and drag in the plot to zoom X: Y: Zoom Out Print Enable tooltip

b+	#	Seq	#	y+
112.0393	1	Q	17	
240.0979	2	Q	16	1733.8956
341.1456	3	T	15	1605.8370
440.2140	4	V	14	1504.7894
541.2617	5	T	13	1405.7209
654.3457	6	L	12	1304.6733
767.4298	7	L	11	1191.5892
864.4825	8	P	10	1078.5051
935.5197	9	A	9	981.4524
1006.5568	10	A	8	910.4153
1121.5837	11	D	7	839.3781
1234.6678	12	L	6	724.3512
1349.6947	13	D	5	611.2671
1464.7217	14	D	4	496.2402
1611.7901	15	F	3	381.2132
1698.8221	16	S	2	234.1448
	17	K	1	147.1128

[Click] to move table

Add to N-term: -17.026549

TQHGKEDLKFPR

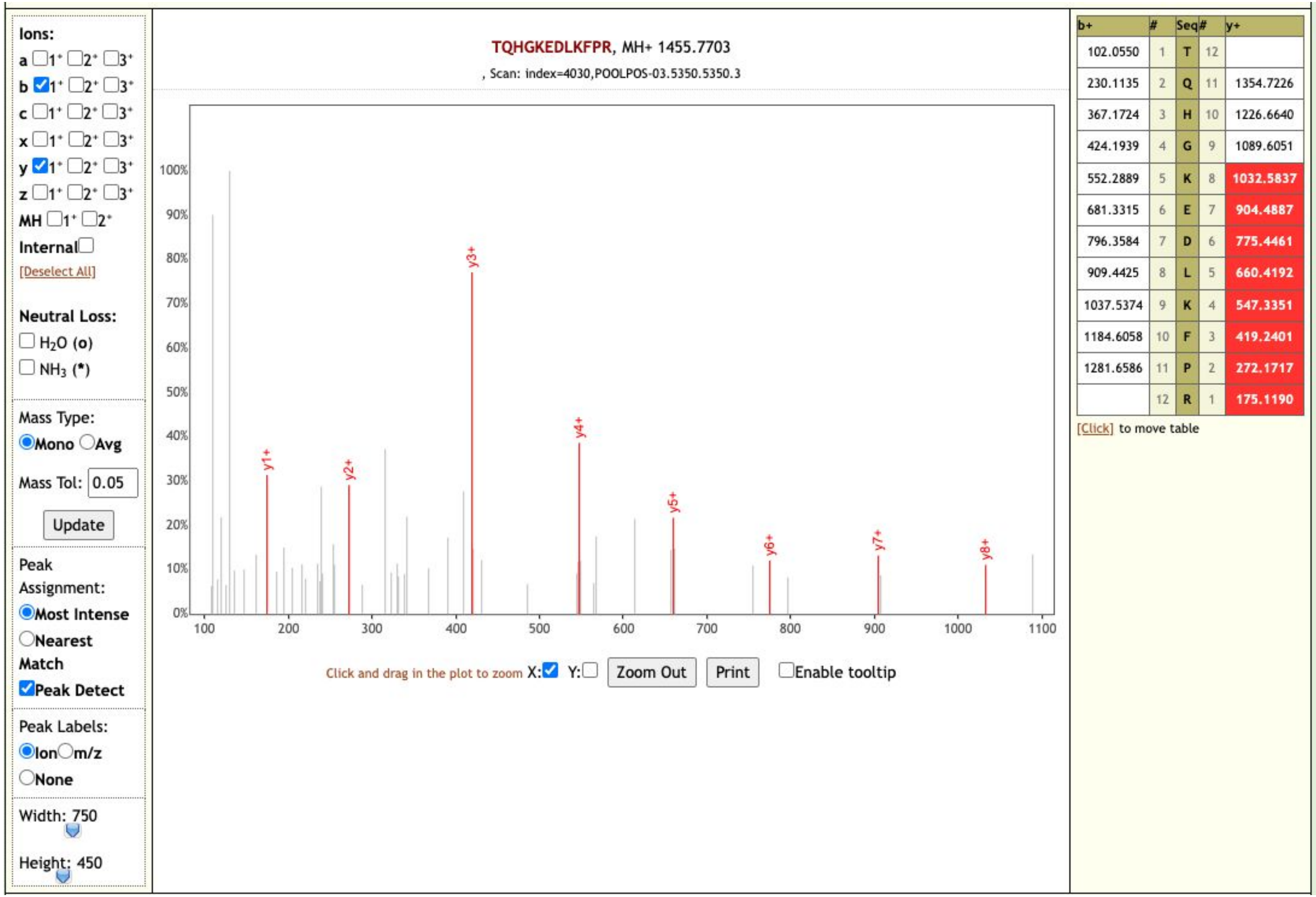

b+	#	Seq	#	y+
102.0550	1	T	12	
230.1135	2	Q	11	1354.7226
367.1724	3	H	10	1226.6640
424.1939	4	G	9	1089.6051
552.2889	5	K	8	1032.5837
681.3315	6	E	7	904.4887
796.3584	7	D	6	775.4461
909.4425	8	L	5	660.4192
1037.5374	9	K	4	547.3351
1184.6058	10	F	3	419.2401
1281.6586	11	P	2	272.1717
	12	R	1	175.1190

YLGTGPEAGLPYGANK

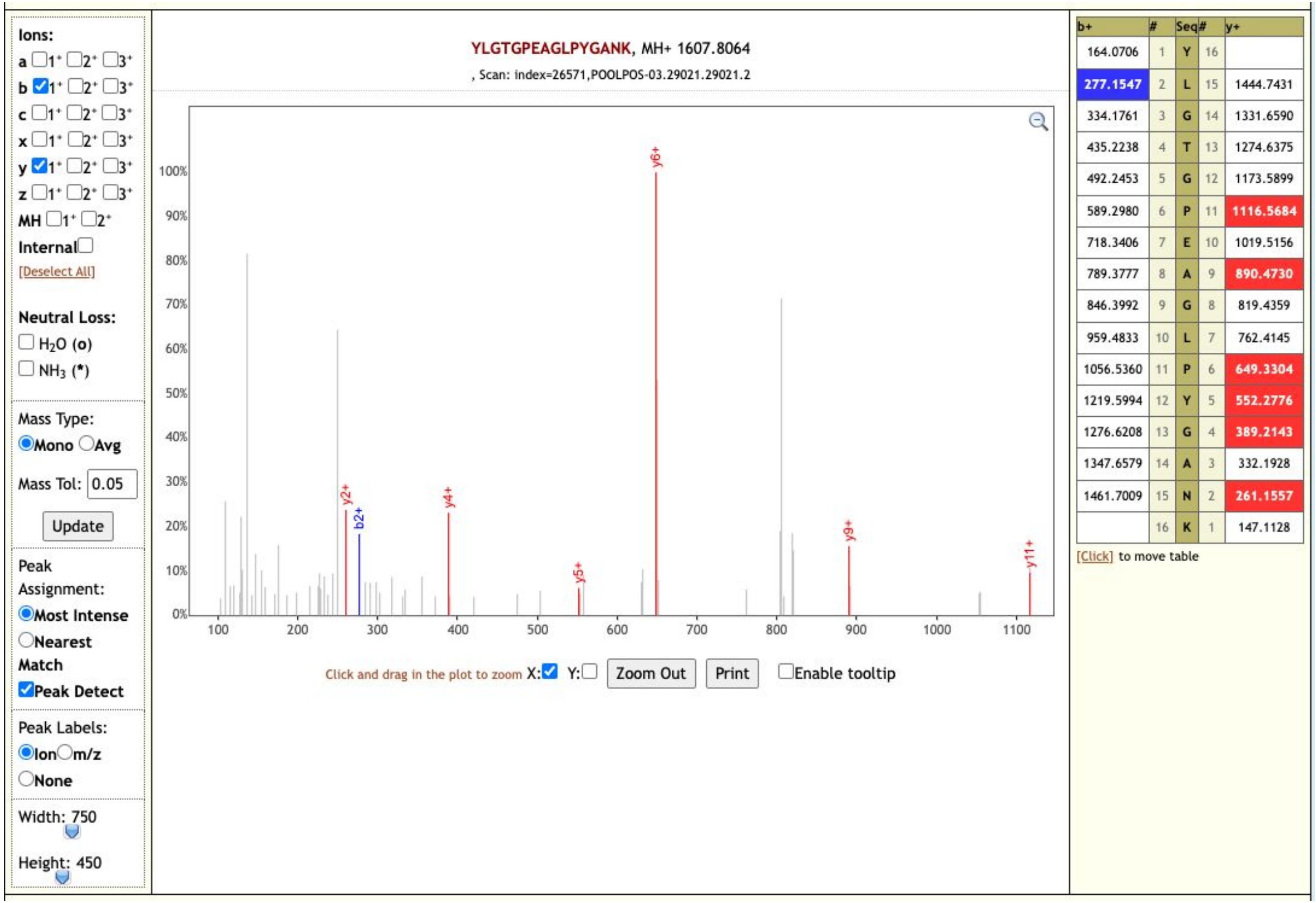

b+	#	Seq	#	y+
164.0706	1	Y	16	
277.1547	2	L	15	1444.7431
334.1761	3	G	14	1331.6590
435.2238	4	T	13	1274.6375
492.2453	5	G	12	1173.5899
589.2980	6	P	11	1116.5684
718.3406	7	E	10	1019.5156
789.3777	8	A	9	890.4730
846.3992	9	G	8	819.4359
959.4833	10	L	7	762.4145
1056.5360	11	P	6	649.3304
1219.5994	12	Y	5	552.2776
1276.6208	13	G	4	389.2143
1347.6579	14	A	3	332.1928
1461.7009	15	N	2	261.1557
	16	K	1	147.1128

YLGTGPEAGLPYGANK

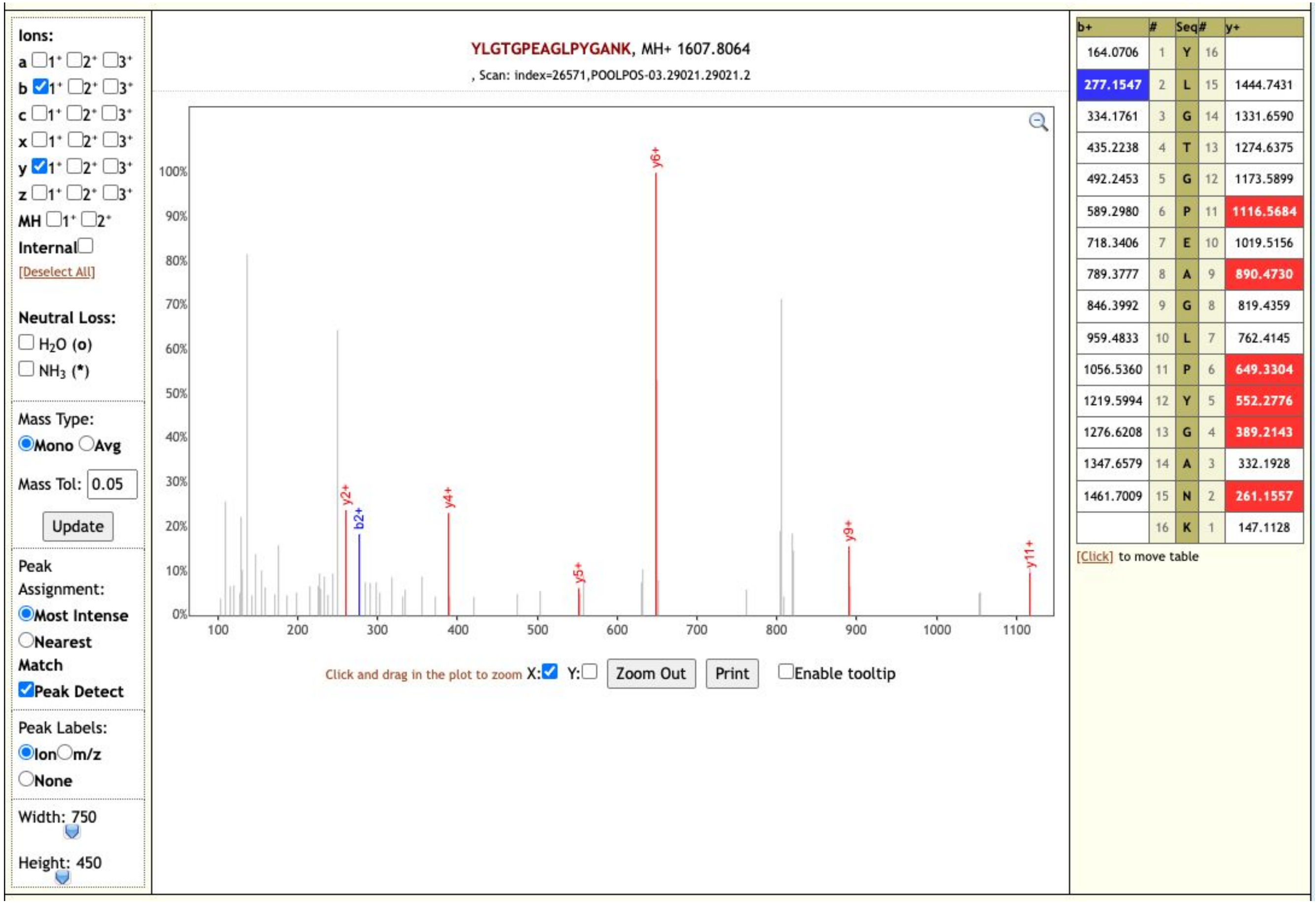

b+	#	Seq	#	y+
164.0706	1	Y	16	
277.1547	2	L	15	1444.7431
334.1761	3	G	14	1331.6590
435.2238	4	T	13	1274.6375
492.2453	5	G	12	1173.5899
589.2980	6	P	11	1116.5684
718.3406	7	E	10	1019.5156
789.3777	8	A	9	890.4730
846.3992	9	G	8	819.4359
959.4833	10	L	7	762.4145
1056.5360	11	P	6	649.3304
1219.5994	12	Y	5	552.2776
1276.6208	13	G	4	389.2143
1347.6579	14	A	3	332.1928
1461.7009	15	N	2	261.1557
	16	K	1	147.1128

[Click] to move table

WYFYYLGTGPEAGLPYGANK

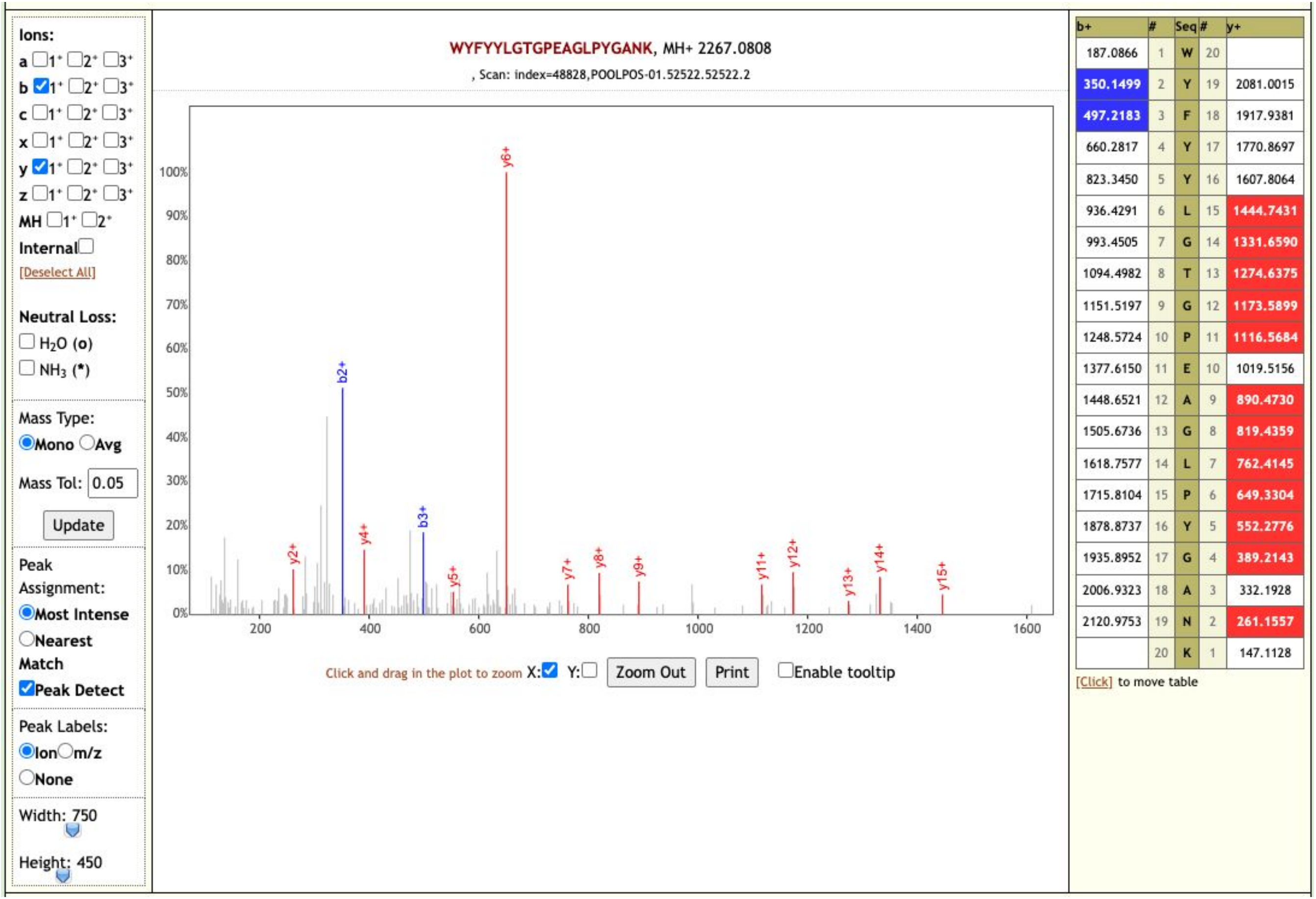

b+	#	Seq	#	y+
187.0866	1	W	20	
350.1499	2	Y	19	2081.0015
497.2183	3	F	18	1917.9381
660.2817	4	Y	17	1770.8697
823.3450	5	Y	16	1607.8064
936.4291	6	L	15	1444.7431
993.4505	7	G	14	1331.6590
1094.4982	8	T	13	1274.6375
1151.5197	9	G	12	1173.5899
1248.5724	10	P	11	1116.5684
1377.6150	11	E	10	1019.5156
1448.6521	12	A	9	890.4730
1505.6736	13	G	8	819.4359
1618.7577	14	L	7	762.4145
1715.8104	15	P	6	649.3304
1878.8737	16	Y	5	552.2776
1935.8952	17	G	4	389.2143
2006.9323	18	A	3	332.1928
2120.9753	19	N	2	261.1557
	20	K	1	147.1128

PXD026795

QYNVTQAFGR

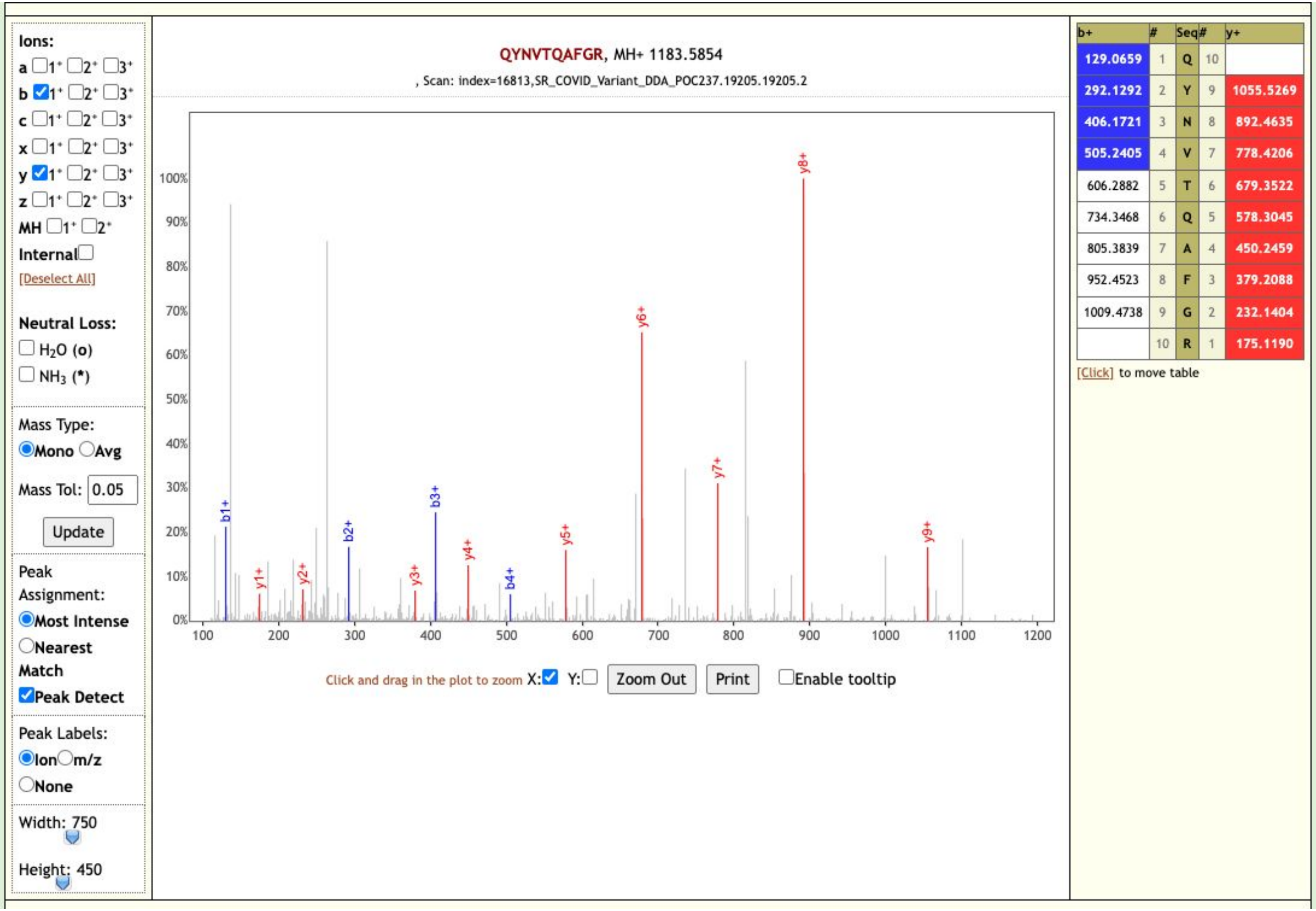

Ions:
a 1+ 2+ 3+
b 1+ 2+ 3+
c 1+ 2+ 3+
x 1+ 2+ 3+
y 1+ 2+ 3+
z 1+ 2+ 3+
MH 1+ 2+
Internal
[Deselect All]
Neutral Loss:
H2O (o)
NH3 (*)
Mass Type:
Mono Avg
Mass Tol: 0.05
Update
Peak Assignment:
Most Intense
Nearest Match
Peak Detect
Peak Labels:
Ion m/z
None
Width: 750
Height: 450
QYNVTQAFGR, MH+ 1183.5854
, Scan: index=16813,SR_COVID_Variant_DDA_POC237.19205.19205.2
b1+ y1+ y2+ b2+ y3+ b3+ y4+ b4+ y5+ y6+ y7+ y8+ y9+
Click and drag in the plot to zoom X: Y: Zoom Out Print Enable tooltip
b+ | # | Seq | # | y+
129.0659 | 1 | Q | 10 |
292.1292 | 2 | Y | 9 | 1055.5269
406.1721 | 3 | N | 8 | 892.4635
505.2405 | 4 | V | 7 | 778.4206
606.2882 | 5 | T | 6 | 679.3522
734.3468 | 6 | Q | 5 | 578.3045
805.3839 | 7 | A | 4 | 450.2459
952.4523 | 8 | F | 3 | 379.2088
1009.4738 | 9 | G | 2 | 232.1404
 | 10 | R | 1 | 175.1190
[Click] to move table

Gamma

AYNVTQAFGR

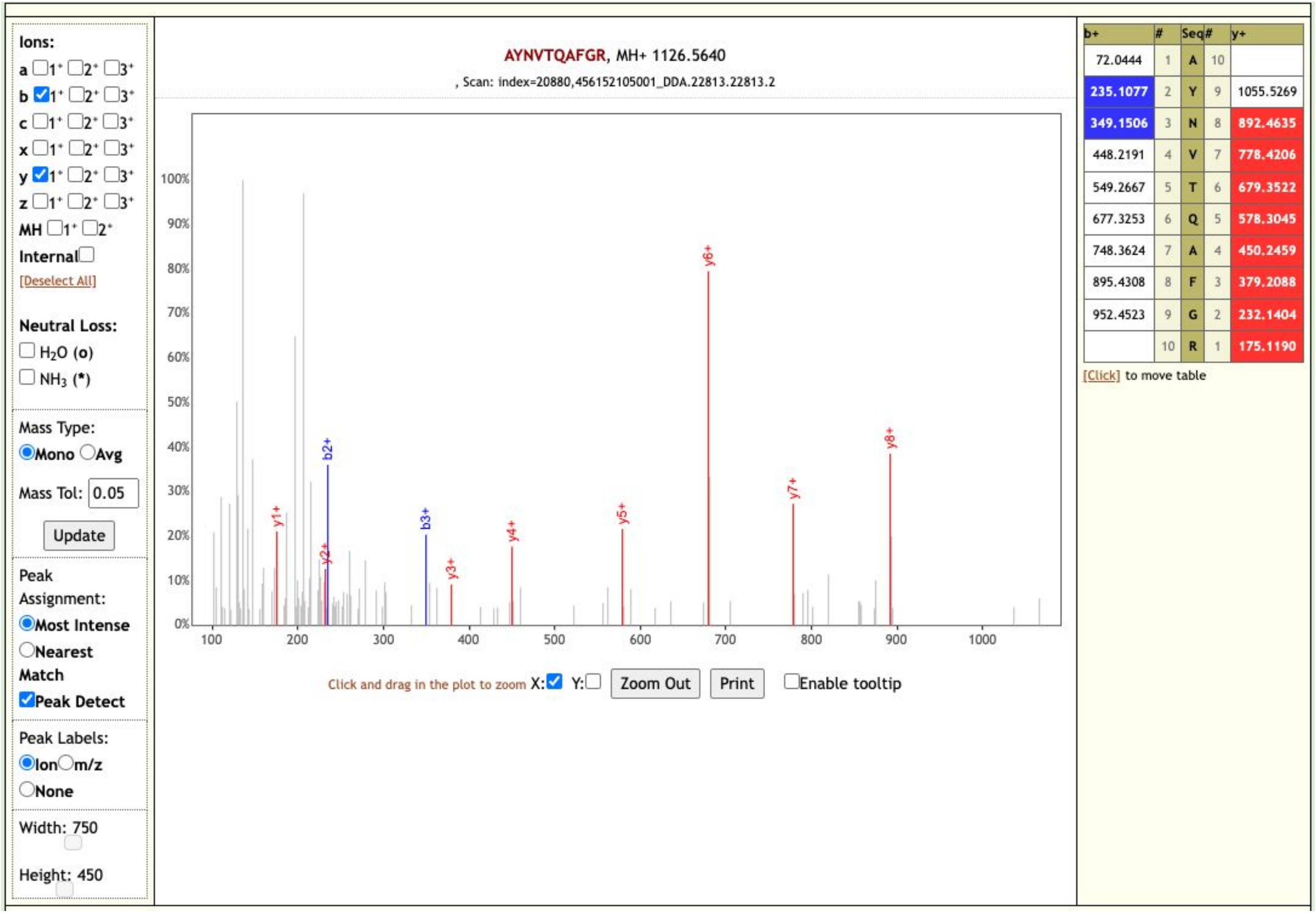
AYNVTQAFGR, MH+ 1126.5640
, Scan: index=20880,456152105001_DDA.22813.22813.2
Ions:
Internal
[Deselect All]
Neutral Loss:
H2O (o)
NH3 (*)
Mass Type:
Mono Avg
Mass Tol: 0.05
Update
Peak Assignment:
Most Intense
Nearest Match
Peak Detect
Peak Labels:
Ion m/z
None
Width: 750
Height: 450
y1+
b2+
y2+
b3+
y3+
y4+
y5+
y6+
y7+
y8+
Click and drag in the plot to zoom X: Y:
Zoom Out
Print
Enable tooltip
b+ # Seq # y+
72.0444 1 A 10
235.1077 2 Y 9 1055.5269
349.1506 3 N 8 892.4635
448.2191 4 V 7 778.4206
549.2667 5 T 6 679.3522
677.3253 6 Q 5 578.3045
748.3624 7 A 4 450.2459
895.4308 8 F 3 379.2088
952.4523 9 G 2 232.1404
10 R 1 175.1190
[Click] to move table

RPQGLPNNTASWFTALTQHGK

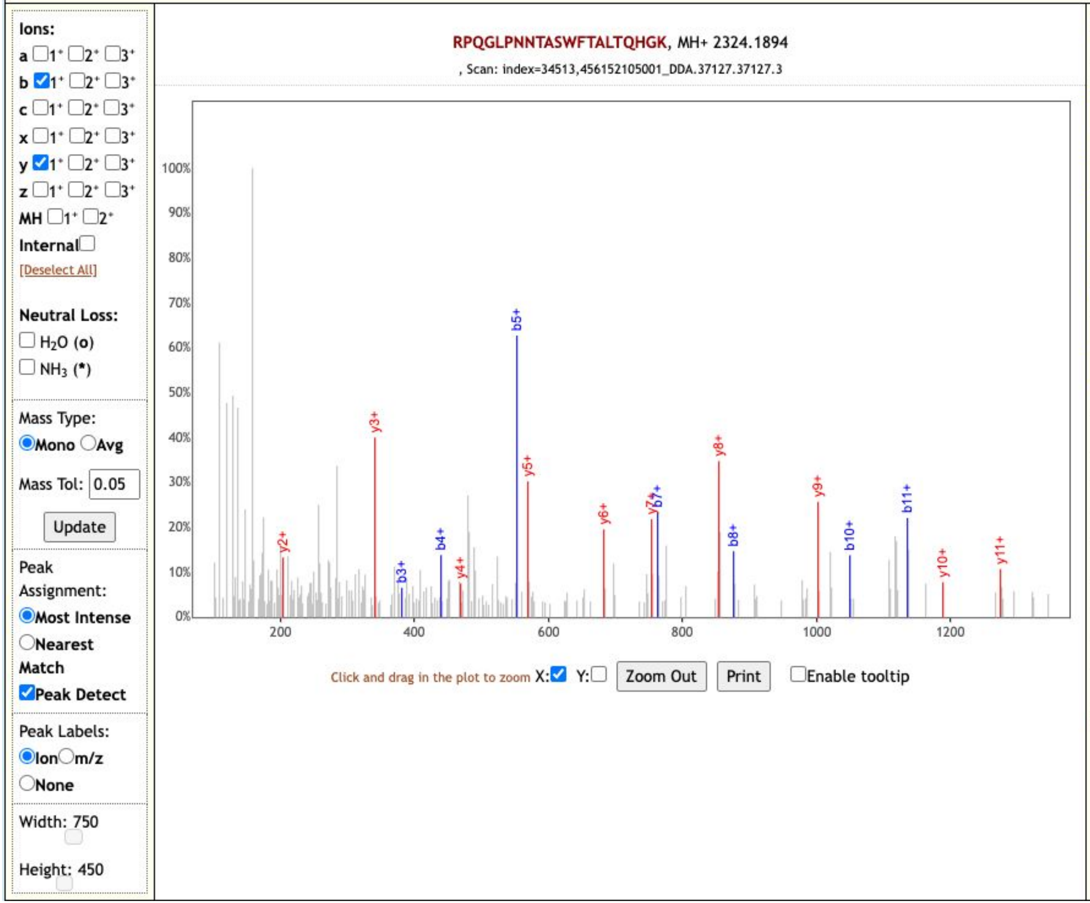

b+	#	Seq	#	y+
157.1084	1	R	21	
254.1612	2	P	20	2168.0883
382.2197	3	Q	19	2071.0356
439.2412	4	G	18	1942.9770
552.3253	5	L	17	1885.9555
649.3780	6	P	16	1772.8715
763.4209	7	N	15	1675.8187
877.4639	8	N	14	1561.7758
978.5116	9	T	13	1447.7328
1049.5487	10	A	12	1346.6852
1136.5807	11	S	11	1275.6480
1322.6600	12	W	10	1188.6160
1469.7284	13	F	9	1002.5367
1570.7761	14	T	8	855.4683
1641.8132	15	A	7	754.4206
1754.8973	16	L	6	683.3835
1855.9450	17	T	5	570.2994
1984.0035	18	Q	4	469.2518
2121.0624	19	H	3	341.1932
2178.0839	20	G	2	204.1343
	21	K	1	147.1128

[Click] to move table

DGIIWVATEGALNTPK

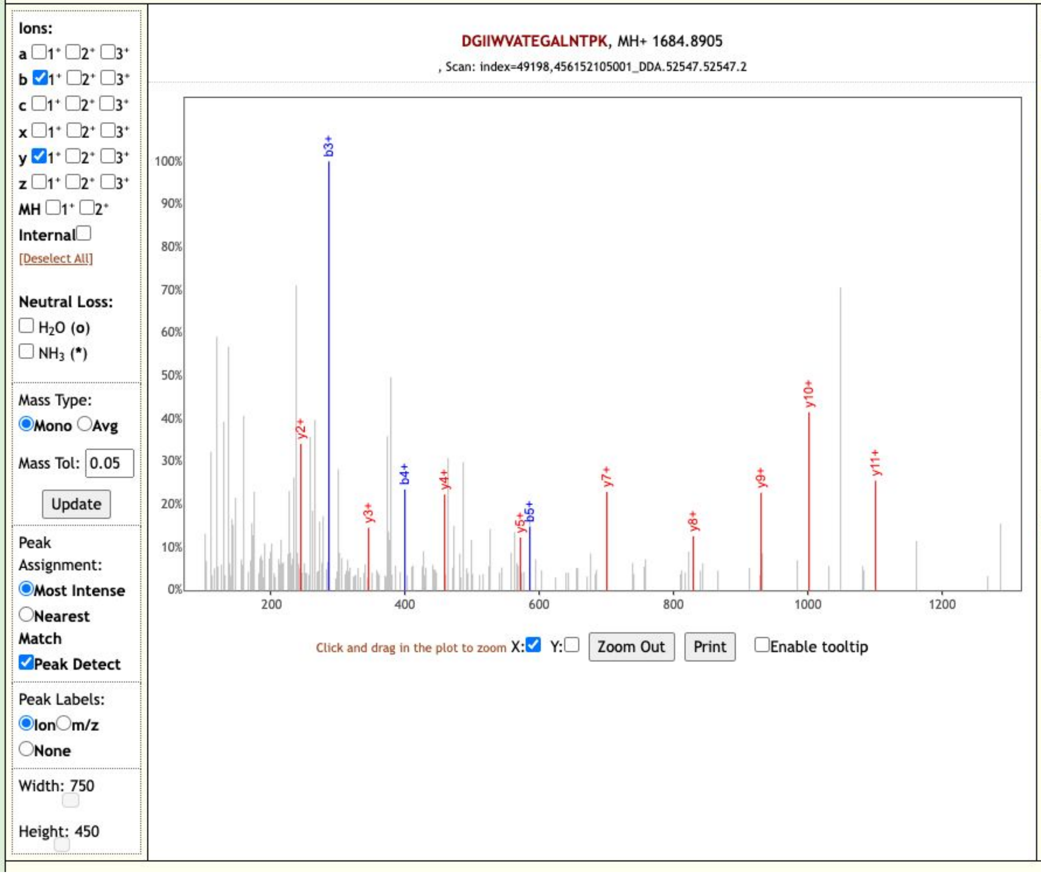

b+	#	Seq	#	y+
116.0342	1	D	16	
173.0557	2	G	15	1569.8635
286.1397	3	I	14	1512.8421
399.2238	4	I	13	1399.7580
585.3031	5	W	12	1286.6739
684.3715	6	V	11	1100.5946
755.4087	7	A	10	1001.5262
856.4563	8	T	9	930.4891
985.4989	9	E	8	829.4414
1042.5204	10	G	7	700.3988
1113.5575	11	A	6	643.3774
1226.6416	12	L	5	572.3402
1340.6845	13	N	4	459.2562
1441.7322	14	T	3	345.2132
1538.7849	15	P	2	244.1656
	16	K	1	147.1128

[Click] to move table

ADETQALPQR

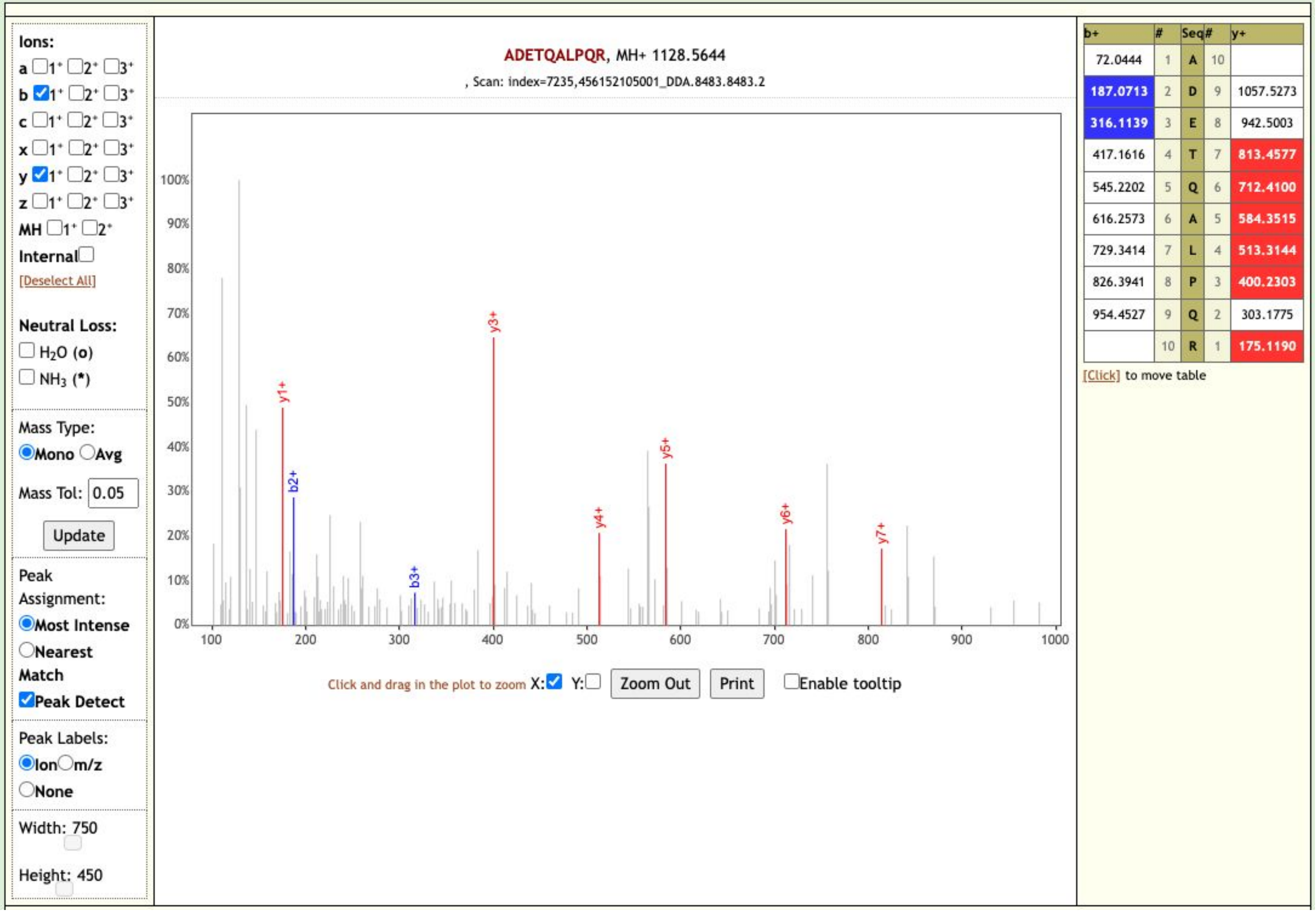

b+	#	Seq	#	y+
72.0444	1	A	10	
187.0713	2	D	9	1057.5273
316.1139	3	E	8	942.5003
417.1616	4	T	7	813.4577
545.2202	5	Q	6	712.4100
616.2573	6	A	5	584.3515
729.3414	7	L	4	513.3144
826.3941	8	P	3	400.2303
954.4527	9	Q	2	303.1775
	10	R	1	175.1190

DDQIGYYR

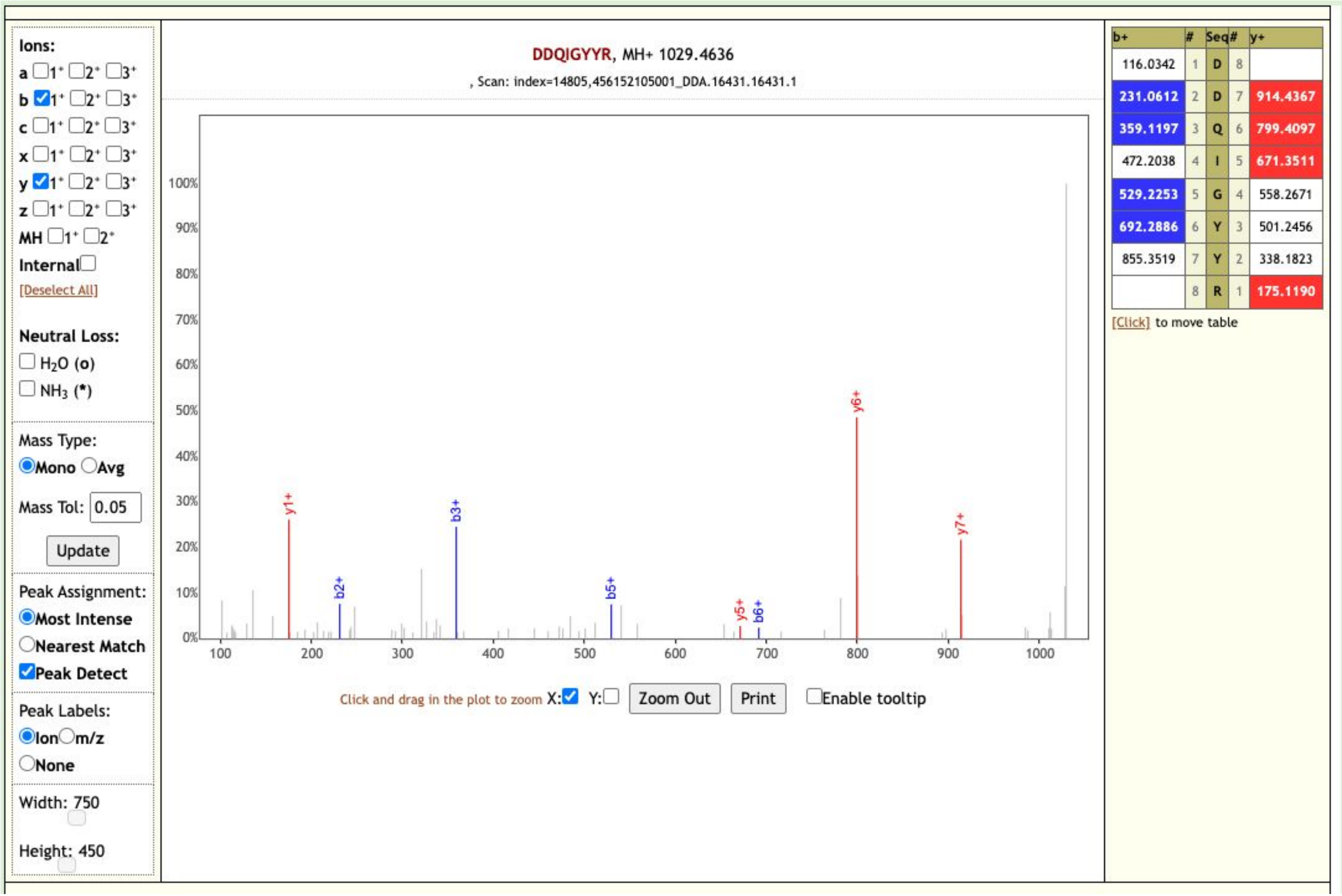
Ions:
a 1+ 2+ 3+
b 1+ 2+ 3+
c 1+ 2+ 3+
x 1+ 2+ 3+
y 1+ 2+ 3+
z 1+ 2+ 3+
MH 1+ 2+
Internal
[Deselect All]
Neutral Loss:
H2O (o)
NH3 (*)
Mass Type:
Mono Avg
Mass Tol: 0.05
Update
Peak Assignment:
Most Intense
Nearest Match
Peak Detect
Peak Labels:
Ion m/z
None
Width: 750
Height: 450
DDQIGYYR, MH+ 1029.4636
, Scan: index=14805,456152105001_DDA.16431.16431.1
100%
90%
80%
70%
60%
50%
40%
30%
20%
10%
0%
100
200
300
400
500
600
700
800
900
1000
y1+
b2+
b3+
b5+
y5+
b6+
y6+
y7+
Click and drag in the plot to zoom X: Y: Zoom Out Print Enable tooltip
b+ # Seq # y+
116.0342 1 D 8
231.0612 2 D 7 914.4367
359.1197 3 Q 6 799.4097
472.2038 4 I 5 671.3511
529.2253 5 G 4 558.2671
692.2886 6 Y 3 501.2456
855.3519 7 Y 2 338.1823
8 R 1 175.1190
[Click] to move table

GEGVPINTNSSPDDQIGYYR

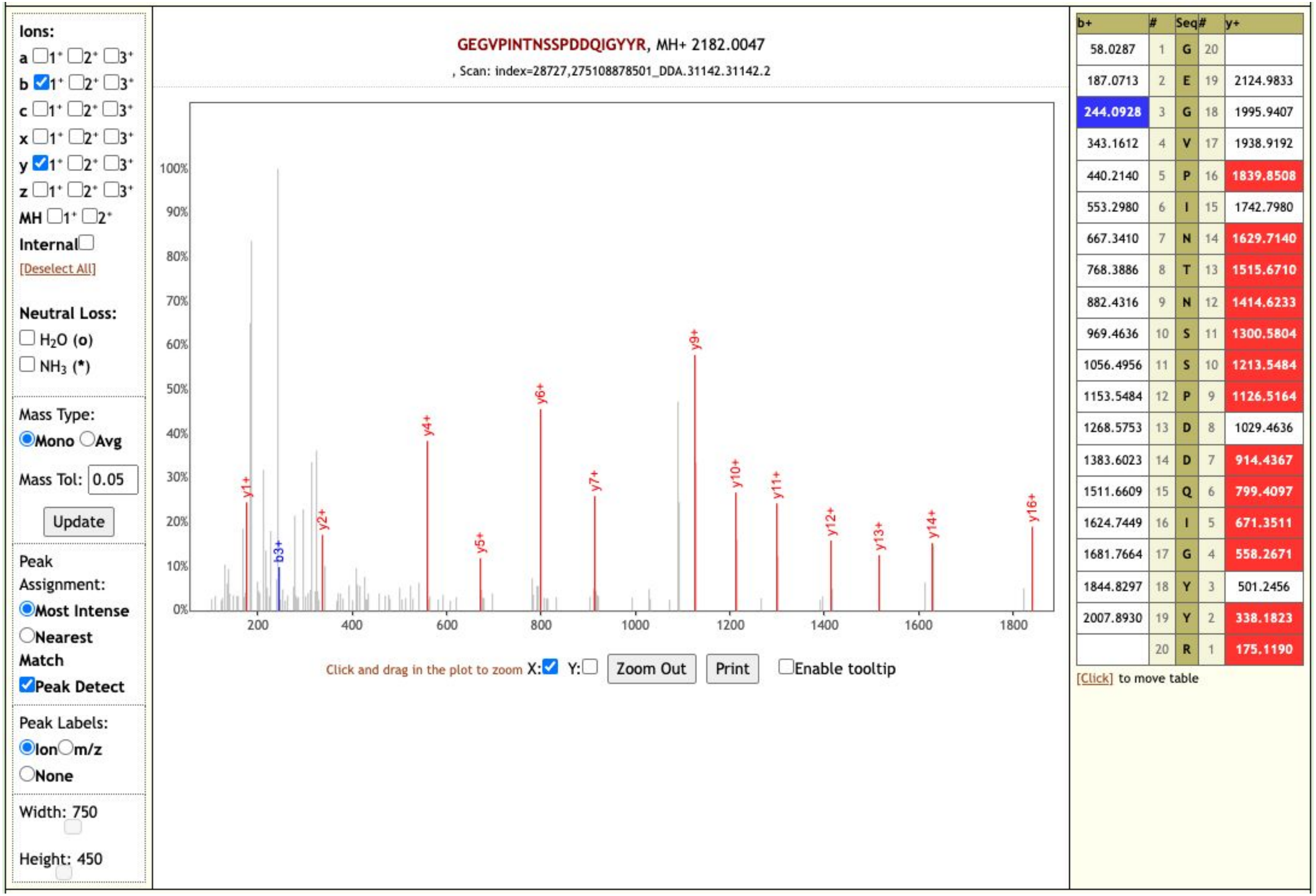

b+	#	Seq	#	y+
58.0287	1	G	20	
187.0713	2	E	19	2124.9833
244.0928	3	G	18	1995.9407
343.1612	4	V	17	1938.9192
440.2140	5	P	16	1839.8508
553.2980	6	I	15	1742.7980
667.3410	7	N	14	1629.7140
768.3886	8	T	13	1515.6710
882.4316	9	N	12	1414.6233
969.4636	10	S	11	1300.5804
1056.4956	11	S	10	1213.5484
1153.5484	12	P	9	1126.5164
1268.5753	13	D	8	1029.4636
1383.6023	14	D	7	914.4367
1511.6609	15	Q	6	799.4097
1624.7449	16	I	5	671.3511
1681.7664	17	G	4	558.2671
1844.8297	18	Y	3	501.2456
2007.8930	19	Y	2	338.1823
	20	R	1	175.1190

[Click] to move table

GPEQTQGNFGDQELIR

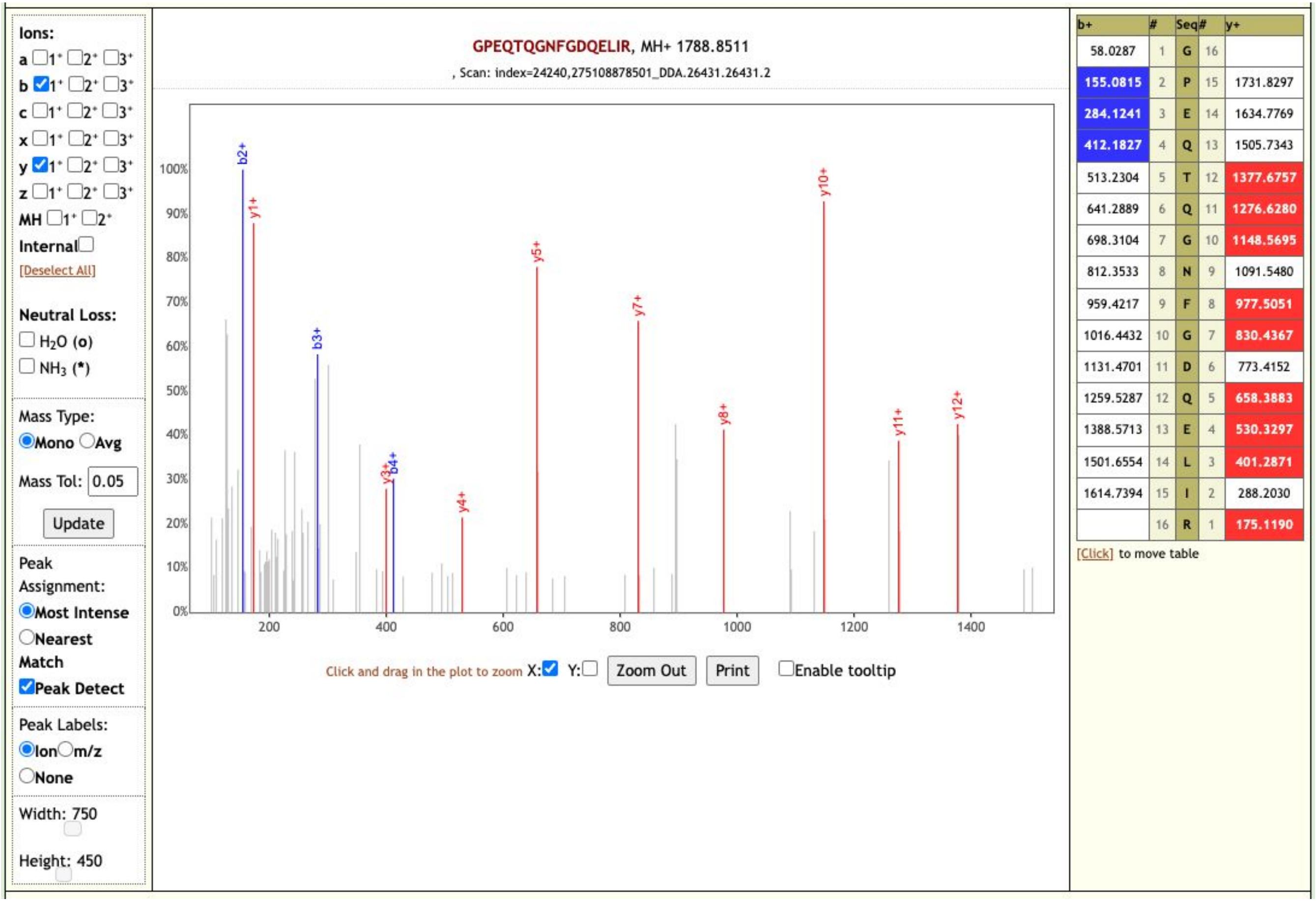

b+	#	Seq	#	y+
58.0287	1	G	16	
155.0815	2	P	15	1731.8297
284.1241	3	E	14	1634.7769
412.1827	4	Q	13	1505.7343
513.2304	5	T	12	1377.6757
641.2889	6	Q	11	1276.6280
698.3104	7	G	10	1148.5695
812.3533	8	N	9	1091.5480
959.4217	9	F	8	977.5051
1016.4432	10	G	7	830.4367
1131.4701	11	D	6	773.4152
1259.5287	12	Q	5	658.3883
1388.5713	13	E	4	530.3297
1501.6554	14	L	3	401.2871
1614.7394	15	I	2	288.2030
	16	R	1	175.1190

[Click] to move table

ITFGGPSDSTGSNQNGER

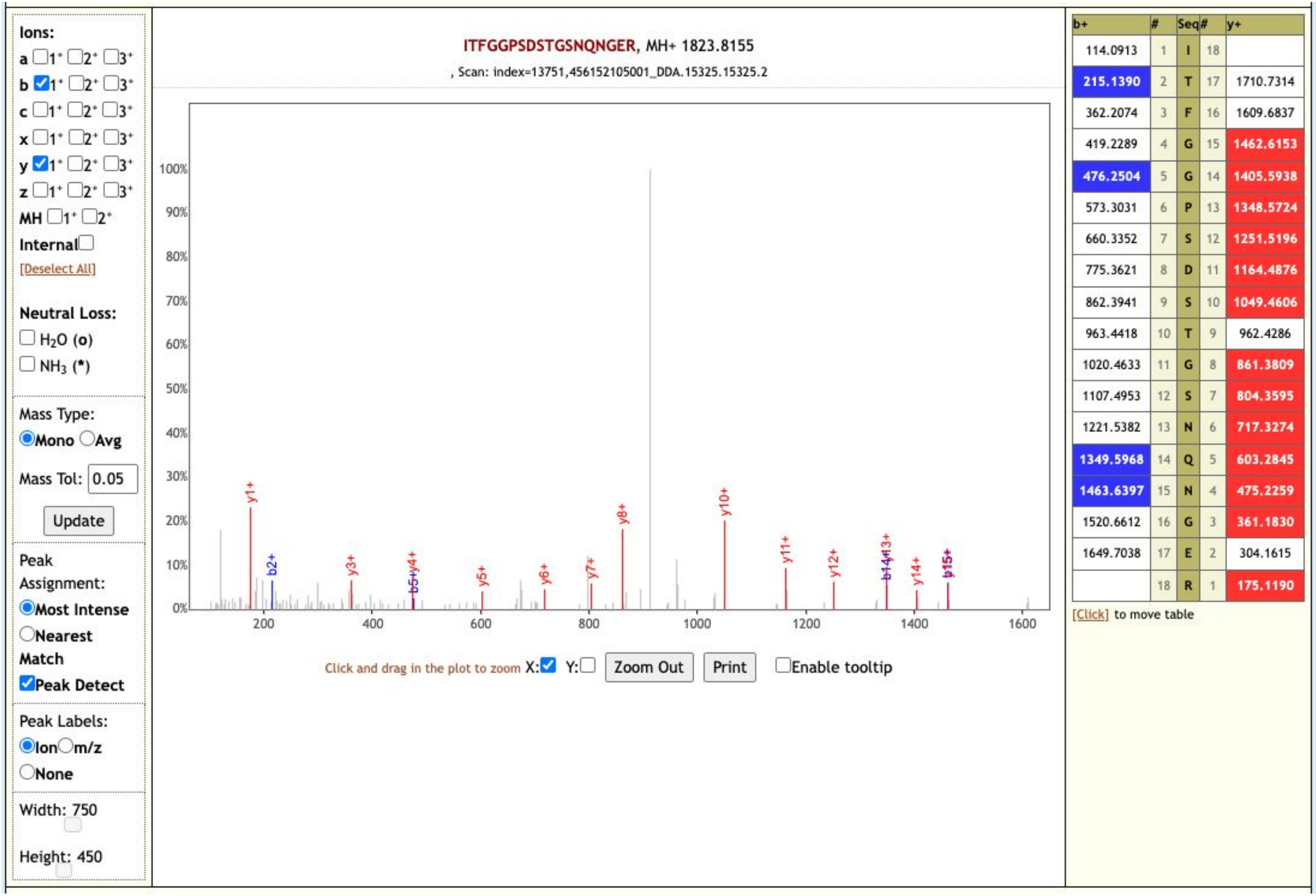

b+	#	Seq	#	y+
114.0913	1	I	18	
215.1390	2	T	17	1710.7314
362.2074	3	F	16	1609.6837
419.2289	4	G	15	1462.6153
476.2504	5	G	14	1405.5938
573.3031	6	P	13	1348.5724
660.3352	7	S	12	1251.5196
775.3621	8	D	11	1164.4876
862.3941	9	S	10	1049.4606
963.4418	10	T	9	962.4286
1020.4633	11	G	8	861.3809
1107.4953	12	S	7	804.3595
1221.5382	13	N	6	717.3274
1349.5968	14	Q	5	603.2845
1463.6397	15	N	4	475.2259
1520.6612	16	G	3	361.1830
1649.7038	17	E	2	304.1615
	18	R	1	175.1190

[Click] to move table

KADETQALPQR

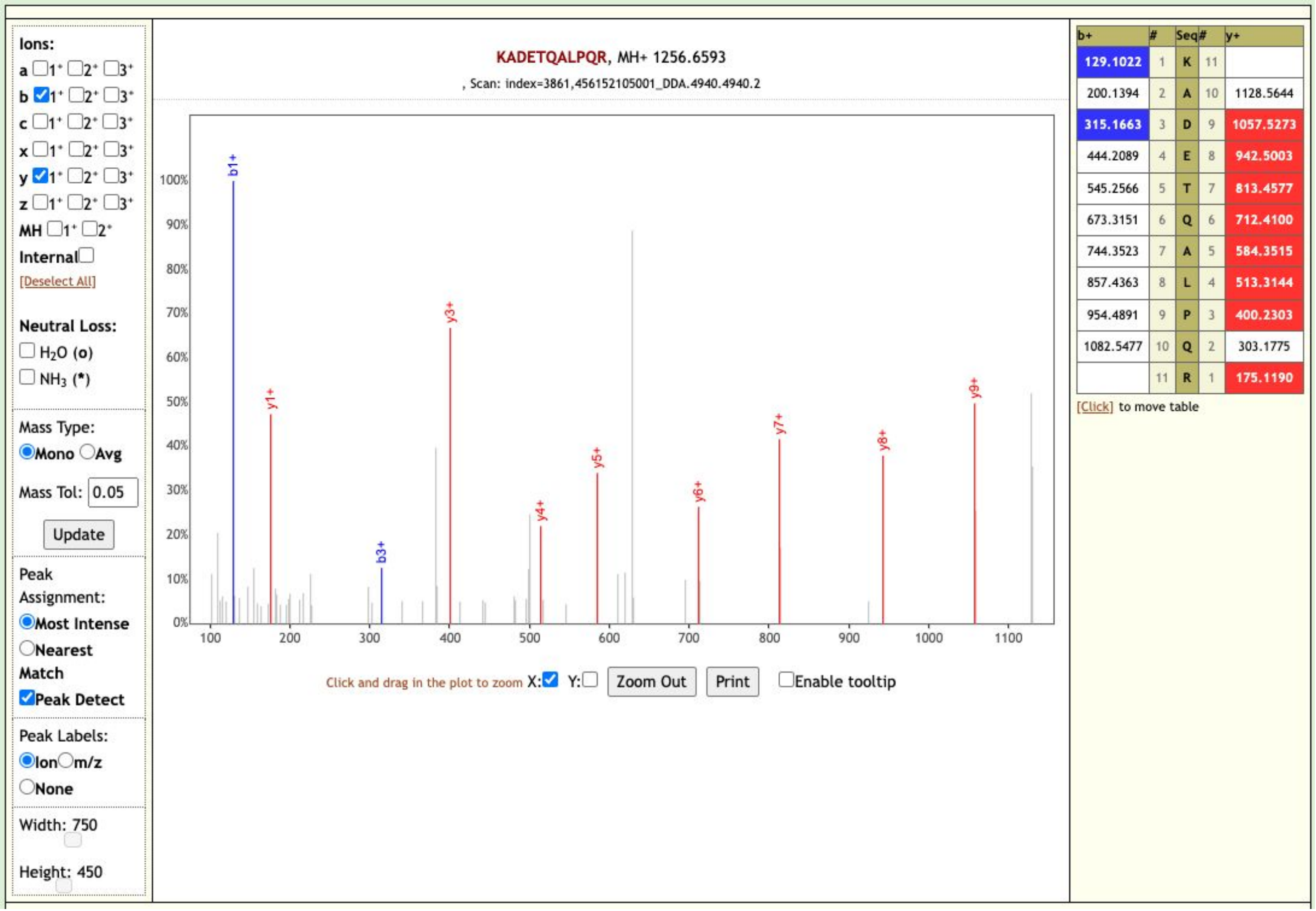

RPQGLPNNTASWF

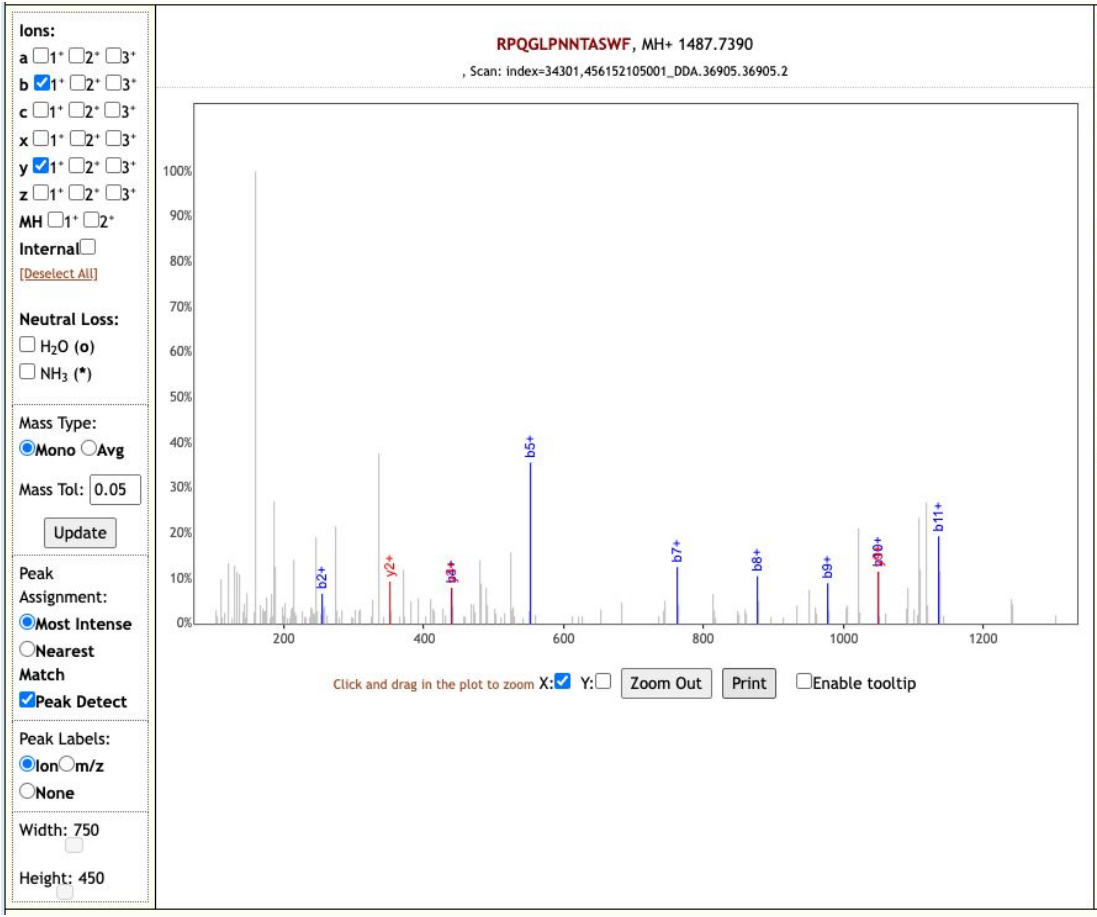

b+	#	Seq	#	y+
157.1084	1	R	13	
254.1612	2	P	12	1331.6379
382.2197	3	Q	11	1234.5851
439.2412	4	G	10	1106.5265
552.3253	5	L	9	1049.5051
649.3780	6	P	8	936.4210
763.4209	7	N	7	839.3682
877.4639	8	N	6	725.3253
978.5116	9	T	5	611.2824
1049.5487	10	A	4	510.2347
1136.5807	11	S	3	439.1976
1322.6600	12	W	2	352.1656
	13	F	1	166.0863

[Click] to move table

Delta

RPQGLPNNTASWFTALTQHGK

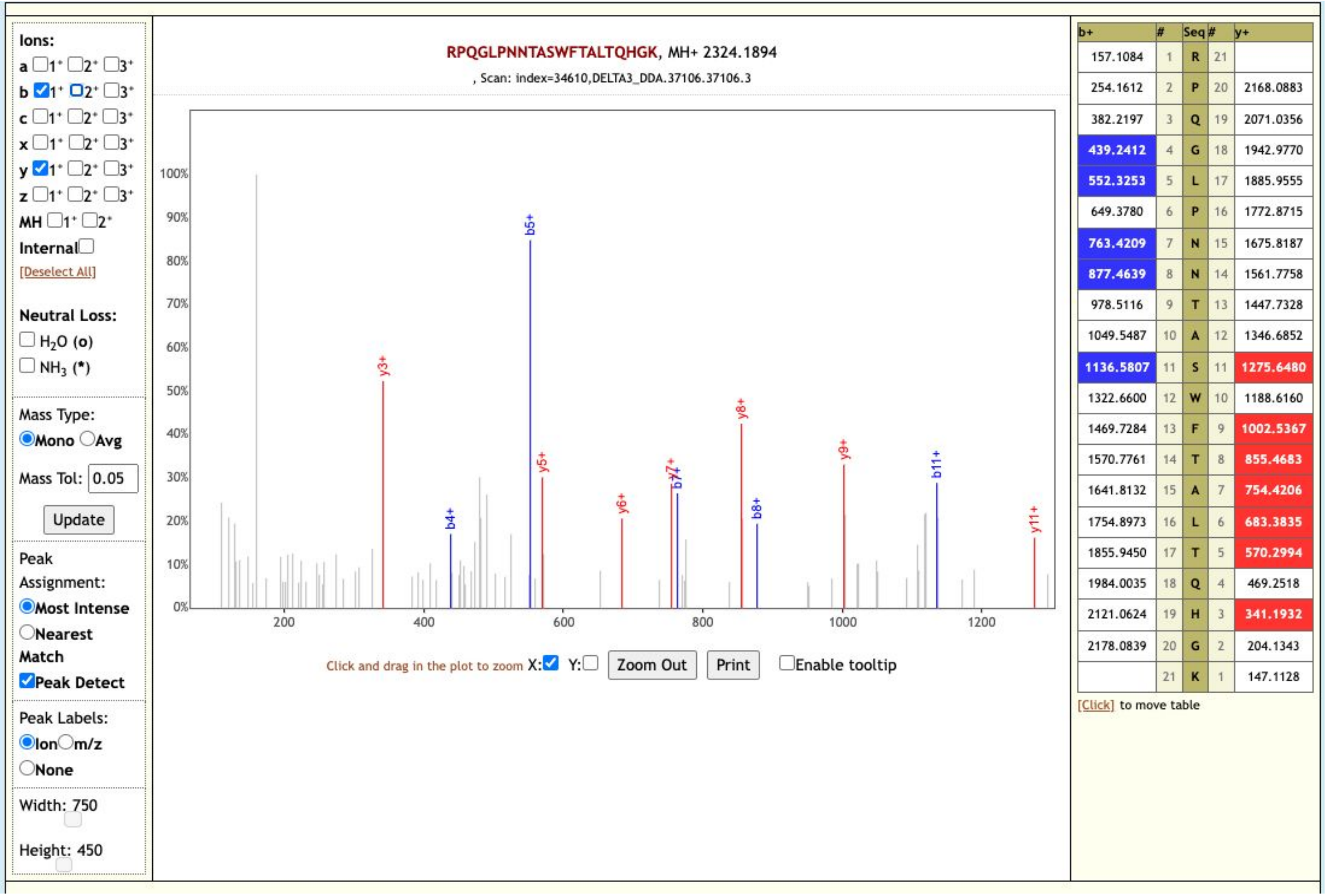

b+	#	Seq	#	y+
157.1084	1	R	21	
254.1612	2	P	20	2168.0883
382.2197	3	Q	19	2071.0356
439.2412	4	G	18	1942.9770
552.3253	5	L	17	1885.9555
649.3780	6	P	16	1772.8715
763.4209	7	N	15	1675.8187
877.4639	8	N	14	1561.7758
978.5116	9	T	13	1447.7328
1049.5487	10	A	12	1346.6852
1136.5807	11	S	11	1275.6480
1322.6600	12	W	10	1188.6160
1469.7284	13	F	9	1002.5367
1570.7761	14	T	8	855.4683
1641.8132	15	A	7	754.4206
1754.8973	16	L	6	683.3835
1855.9450	17	T	5	570.2994
1984.0035	18	Q	4	469.2518
2121.0624	19	H	3	341.1932
2178.0839	20	G	2	204.1343
	21	K	1	147.1128

[Click] to move table

AYNVTQAFGR

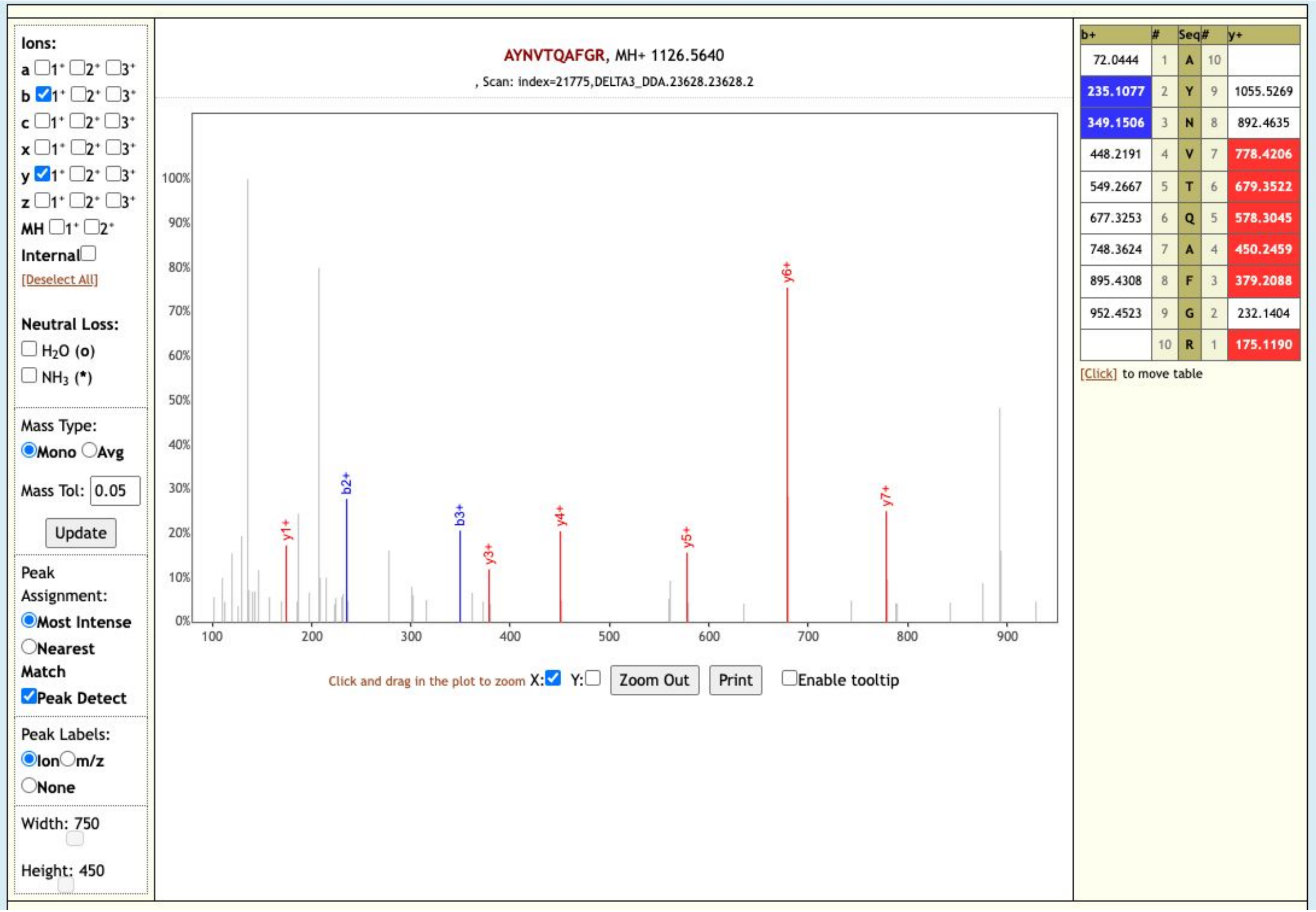

b+	#	Seq	#	y+
72.0444	1	A	10	
235.1077	2	Y	9	1055.5269
349.1506	3	N	8	892.4635
448.2191	4	V	7	778.4206
549.2667	5	T	6	679.3522
677.3253	6	Q	5	578.3045
748.3624	7	A	4	450.2459
895.4308	8	F	3	379.2088
952.4523	9	G	2	232.1404
	10	R	1	175.1190

DGIIWVATEGALNTPK

Ions:
a 1⁺ 2⁺ 3⁺
b 1⁺ 2⁺ 3⁺
c 1⁺ 2⁺ 3⁺
x 1⁺ 2⁺ 3⁺
y 1⁺ 2⁺ 3⁺
z 1⁺ 2⁺ 3⁺
MH 1⁺ 2⁺
Internal
[Deselect All]

Neutral Loss:
H_2O (o)
NH_3 (*)

Mass Type:
Mono Avg

Mass Tol: 0.05

Update

Peak Assignment:
Most Intense
Nearest Match
Peak Detect

Peak Labels:
Ion m/z
None

Width: 750

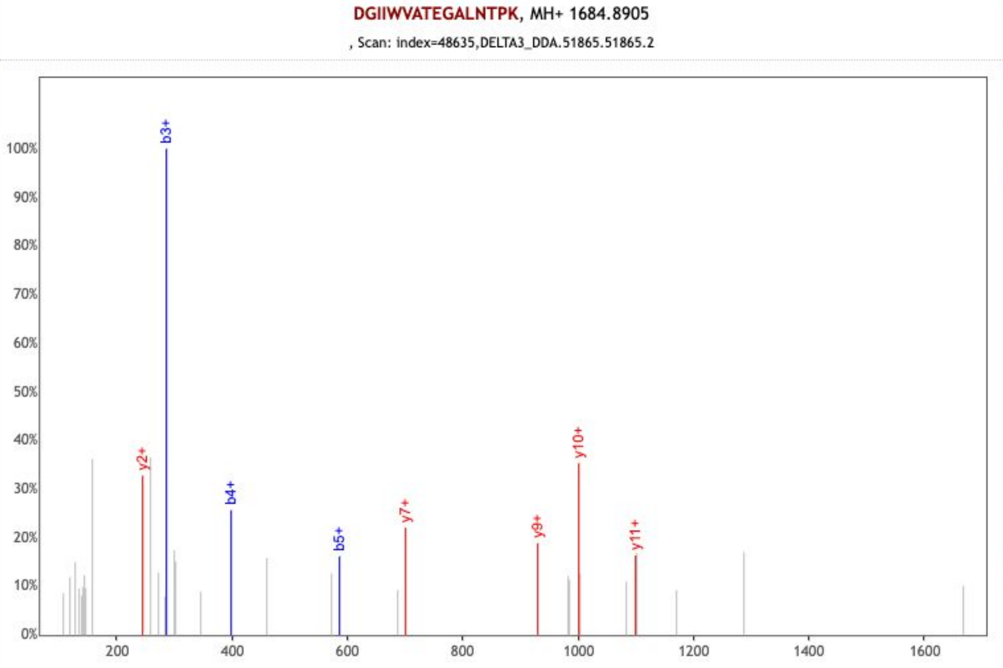

Click and drag in the plot to zoom X: Y: Zoom Out Print Enable tooltip

b+	#	Seq	#	y+
116.0342	1	D	16	
173.0557	2	G	15	1569.8635
286.1397	3	I	14	1512.8421
399.2238	4	I	13	1399.7580
585.3031	5	W	12	1286.6739
684.3715	6	V	11	1100.5946
755.4087	7	A	10	1001.5262
856.4563	8	T	9	930.4891
985.4989	9	E	8	829.4414
1042.5204	10	G	7	700.3988
1113.5575	11	A	6	643.3774
1226.6416	12	L	5	572.3402
1340.6845	13	N	4	459.2562
1441.7322	14	T	3	345.2132
1538.7849	15	P	2	244.1656
	16	K	1	147.1128

[Click] to move table

AYETQALPQR

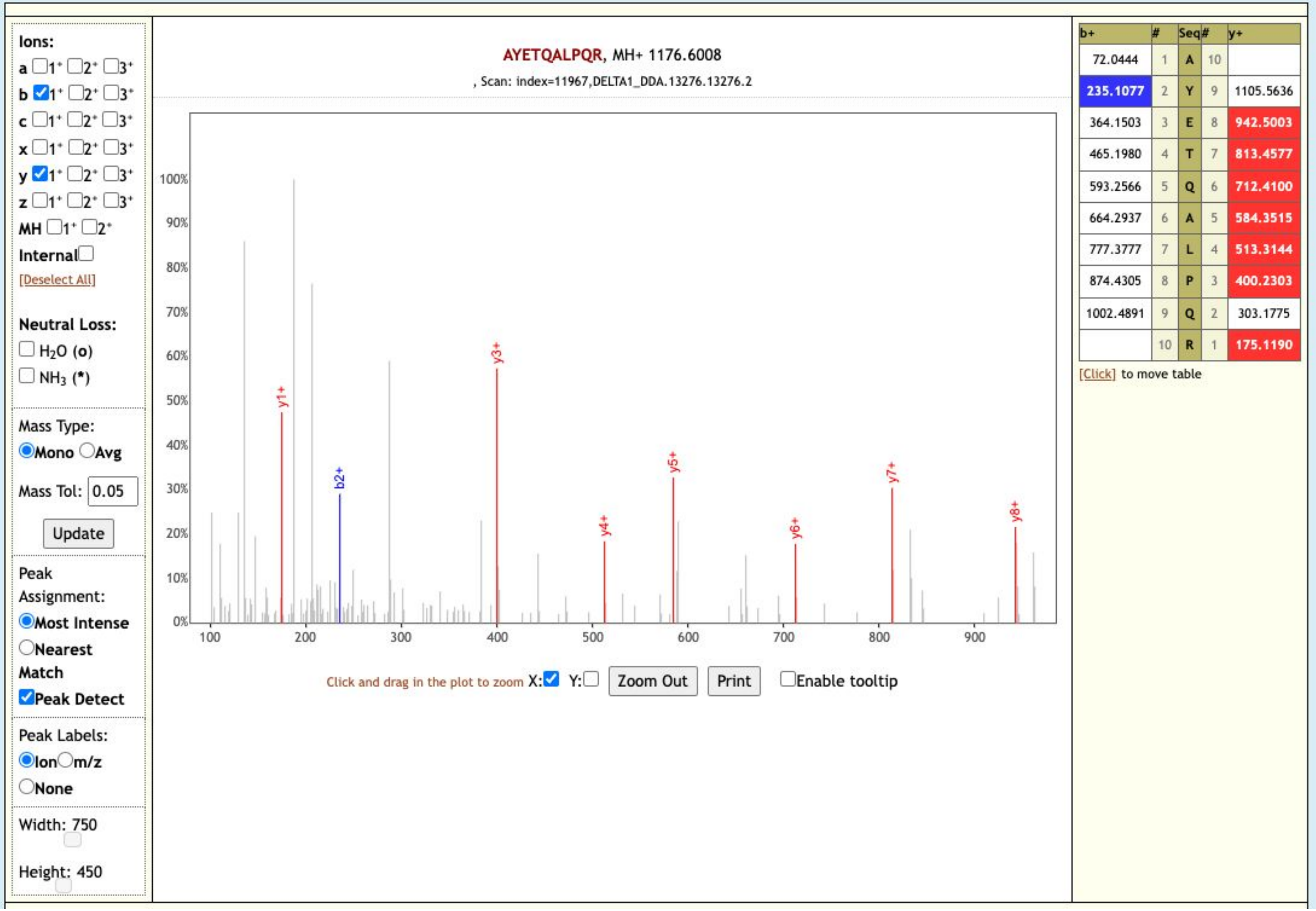

b+	#	Seq	#	y+
72.0444	1	A	10	
235.1077	2	Y	9	1105.5636
364.1503	3	E	8	942.5003
465.1980	4	T	7	813.4577
593.2566	5	Q	6	712.4100
664.2937	6	A	5	584.3515
777.3777	7	L	4	513.3144
874.4305	8	P	3	400.2303
1002.4891	9	Q	2	303.1775
	10	R	1	175.1190

GQGVPINTNSSPDDQIGYYR

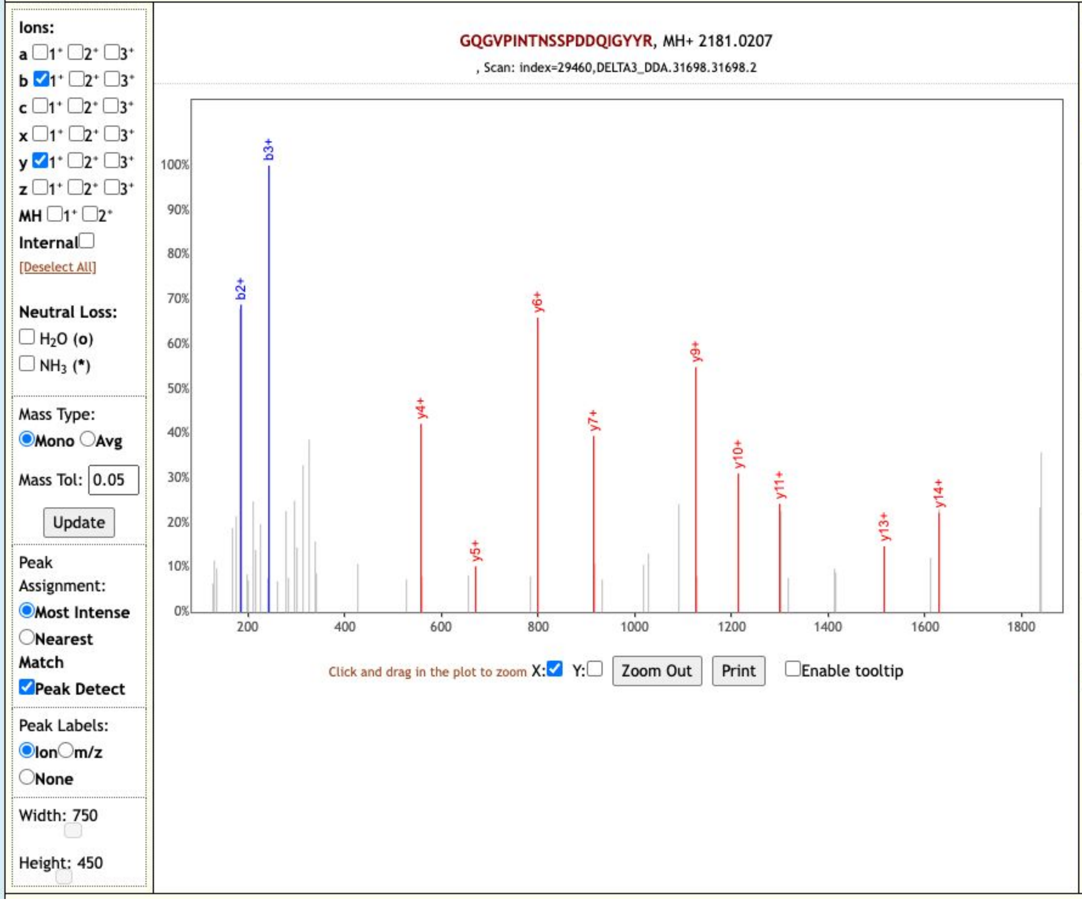

b+	#	Seq	#	y+
58.0287	1	G	20	
186.0873	2	Q	19	2123.9992
243.1088	3	G	18	1995.9407
342.1772	4	V	17	1938.9192
439.2300	5	P	16	1839.8508
552.3140	6	I	15	1742.7980
666.3570	7	N	14	1629.7140
767.4046	8	T	13	1515.6710
881.4476	9	N	12	1414.6233
968.4796	10	S	11	1300.5804
1055.5116	11	S	10	1213.5484
1152.5644	12	P	9	1126.5164
1267.5913	13	D	8	1029.4636
1382.6183	14	D	7	914.4367
1510.6768	15	Q	6	799.4097
1623.7609	16	I	5	671.3511
1680.7824	17	G	4	558.2671
1843.8457	18	Y	3	501.2456
2006.9090	19	Y	2	338.1823
	20	R	1	175.1190

[Click] to move table

LDDKDPNFK

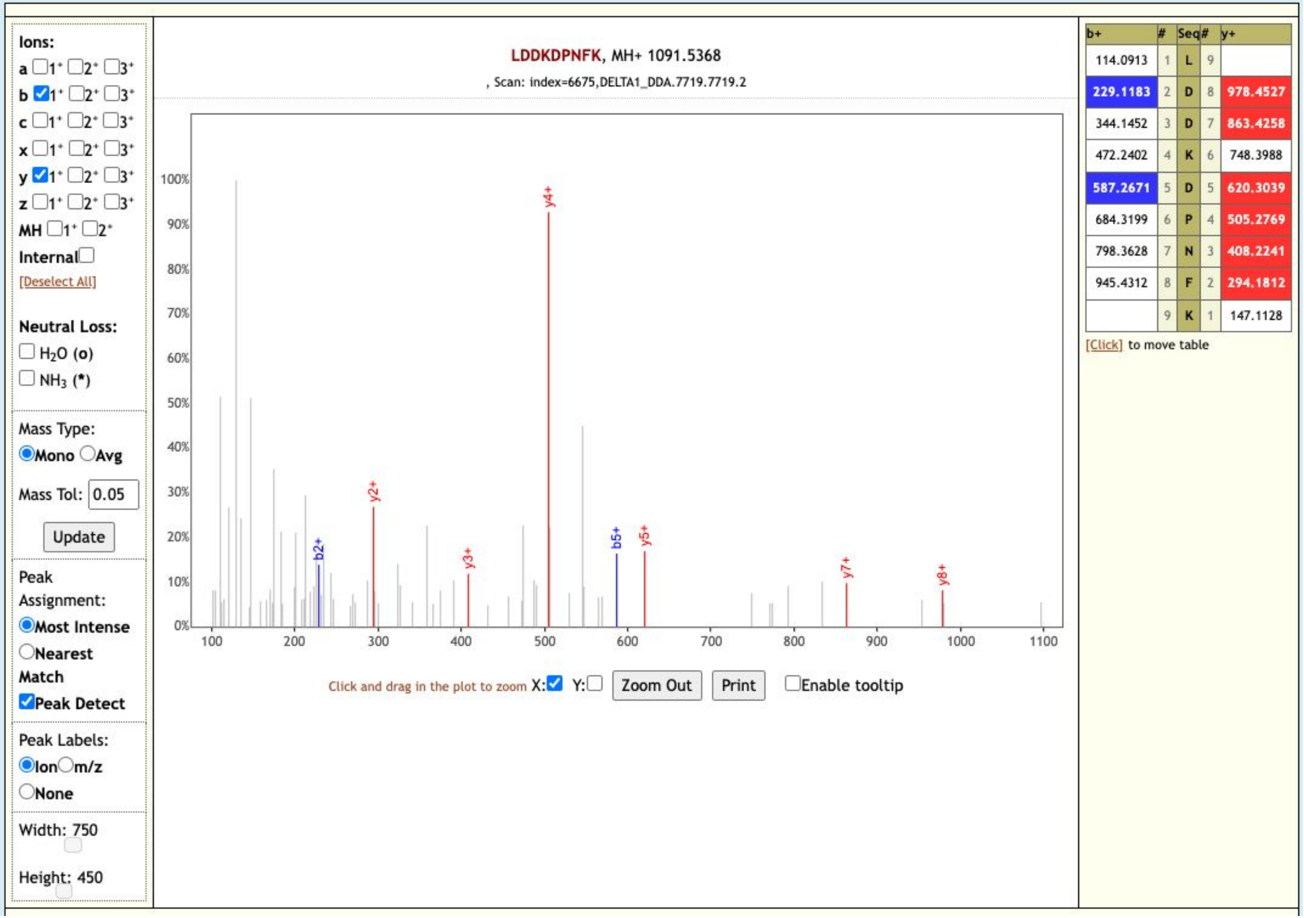

LDDKDPNFK, MH+ 1091.5368
, Scan: index=6675,DELTA1_DDA.7719.7719.2
Ions:
Neutral Loss:
Mass Type:
Mono Avg
Mass Tol: 0.05
Update
Peak Assignment:
Most Intense
Nearest Match
Peak Detect
Peak Labels:
Ion m/z
None
Width: 750
Height: 450
Click and drag in the plot to zoom
Zoom Out
Print
Enable tooltip
b2+
y2+
y3+
y4+
b5+
y5+
y7+
y8+
b+ # Seq # y+
114.0913 1 L 9
229.1183 2 D 8 978.4527
344.1452 3 D 7 863.4258
472.2402 4 K 6 748.3988
587.2671 5 D 5 620.3039
684.3199 6 P 4 505.2769
798.3628 7 N 3 408.2241
945.4312 8 F 2 294.1812
9 K 1 147.1128
[Click] to move table

GFYAEGSR

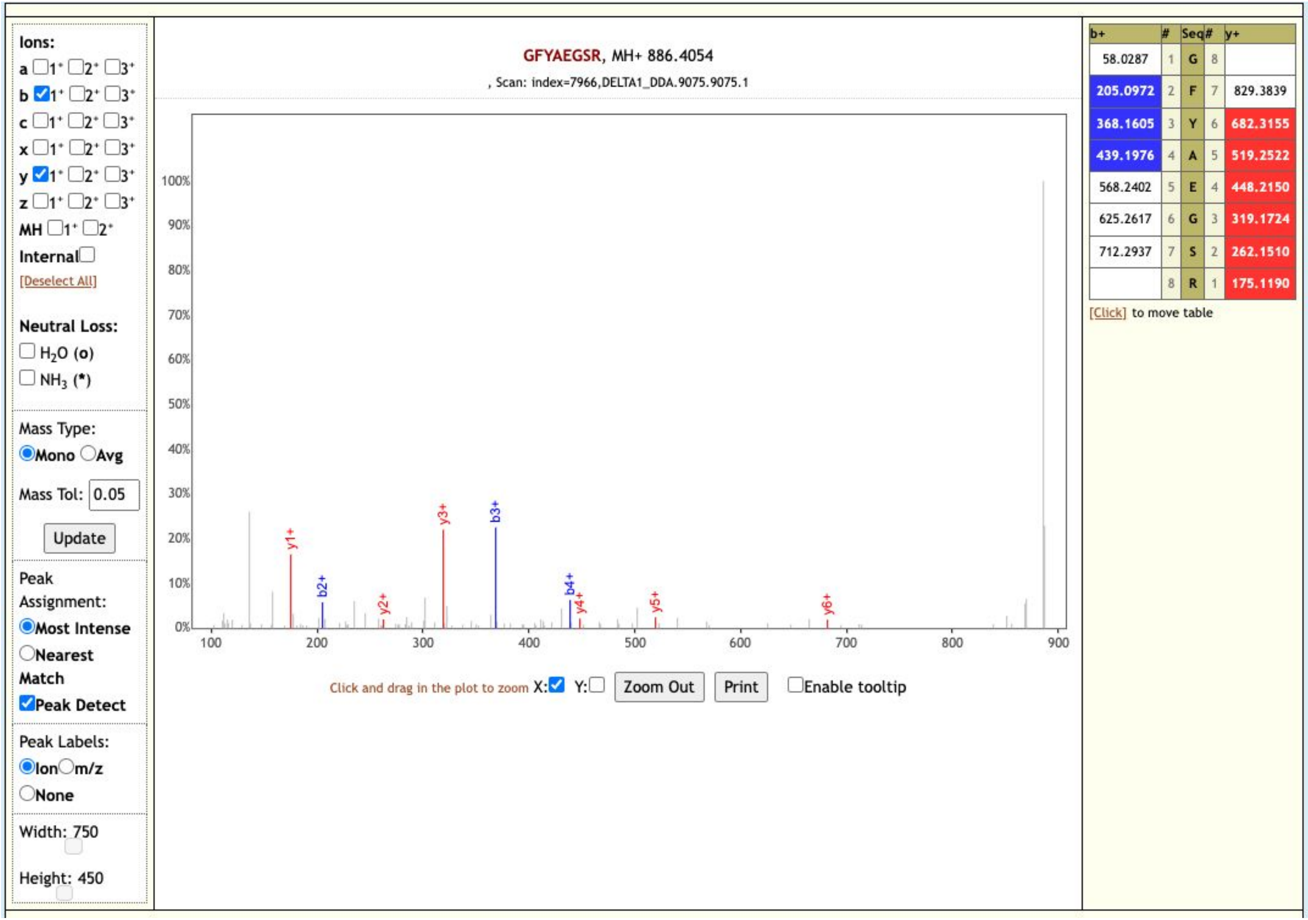

Ions:
a 1+ 2+ 3+
b 1+ 2+ 3+
c 1+ 2+ 3+
x 1+ 2+ 3+
y 1+ 2+ 3+
z 1+ 2+ 3+
MH 1+ 2+
Internal
[Deselect All]
Neutral Loss:
H2O (o)
NH3 (*)
Mass Type:
Mono Avg
Mass Tol: 0.05
Update
Peak Assignment:
Most Intense
Nearest Match
Peak Detect
Peak Labels:
Ion m/z
None
Width: 750
Height: 450
GFYAEGSR, MH+ 886.4054
, Scan: index=7966,DELTA1_DDA.9075.9075.1
100%
90%
80%
70%
60%
50%
40%
30%
20%
10%
0%
100
200
300
400
500
600
700
800
900
y1+
b2+
y2+
y3+
b3+
b4+
y4+
y5+
y6+
Click and drag in the plot to zoom X: Y:
Zoom Out
Print
Enable tooltip
b+ # Seq # y+
58.0287 1 G 8
205.0972 2 F 7 829.3839
368.1605 3 Y 6 682.3155
439.1976 4 A 5 519.2522
568.2402 5 E 4 448.2150
625.2617 6 G 3 319.1724
712.2937 7 S 2 262.1510
8 R 1 175.1190
[Click] to move table

IAGHHLGR

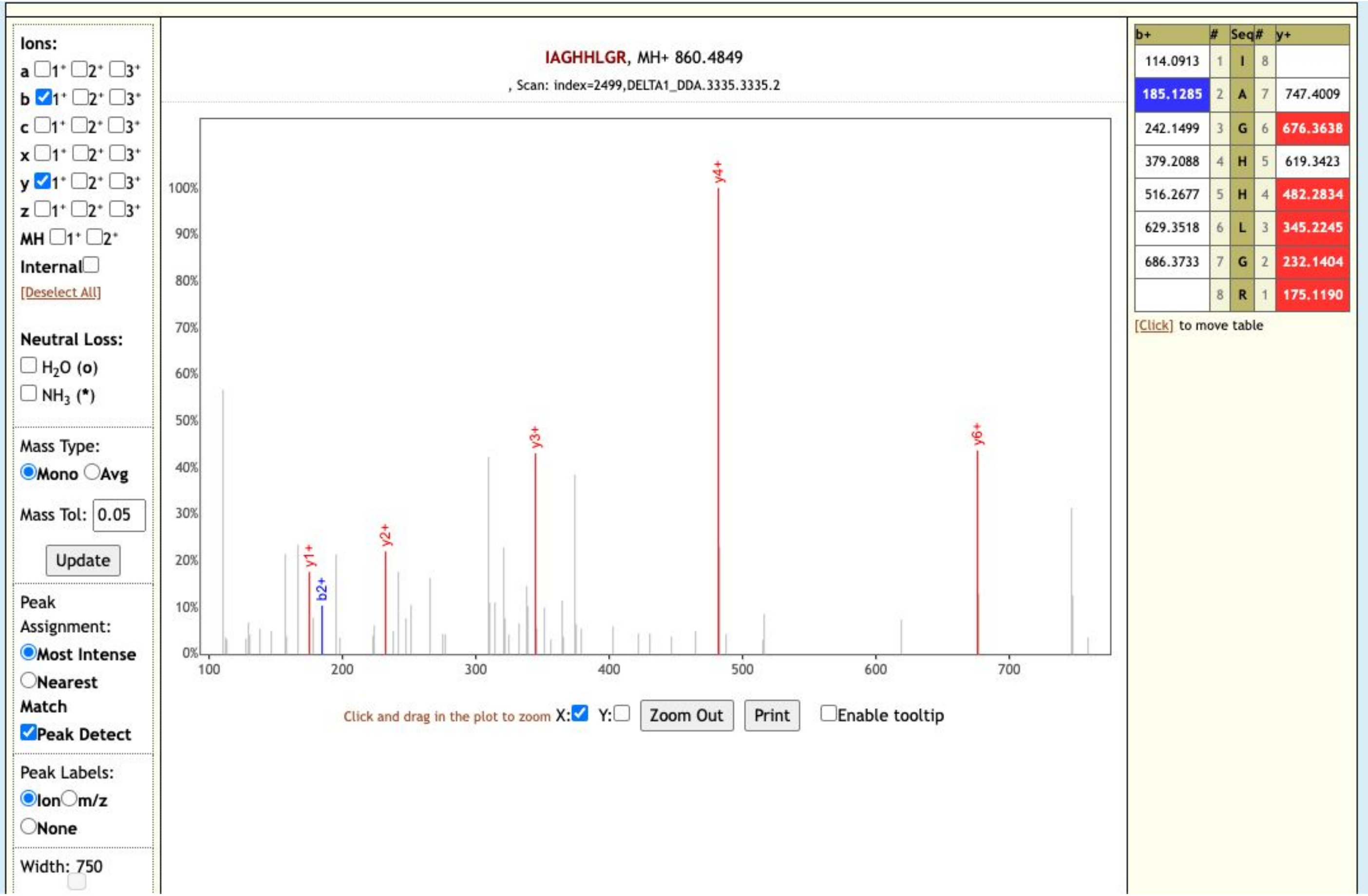
Ions:
a 1+ 2+ 3+
b 1+ 2+ 3+
c 1+ 2+ 3+
x 1+ 2+ 3+
y 1+ 2+ 3+
z 1+ 2+ 3+
MH 1+ 2+
Internal
[Deselect All]
Neutral Loss:
H2O (o)
NH3 (*)
Mass Type:
Mono Avg
Mass Tol: 0.05
Update
Peak Assignment:
Most Intense
Nearest Match
Peak Detect
Peak Labels:
Ion m/z
None
Width: 750
IAGHHLGR, MH+ 860.4849
, Scan: index=2499,DELTA1_DDA.3335.3335.2
100%
90%
80%
70%
60%
50%
40%
30%
20%
10%
0%
100
200
300
400
500
600
700
y1+
b2+
y2+
y3+
y4+
y6+
Click and drag in the plot to zoom X: Y:
Zoom Out
Print
Enable tooltip
b+ | # | Seq | # | y+
114.0913 | 1 | I | 8 |
185.1285 | 2 | A | 7 | 747.4009
242.1499 | 3 | G | 6 | 676.3638
379.2088 | 4 | H | 5 | 619.3423
516.2677 | 5 | H | 4 | 482.2834
629.3518 | 6 | L | 3 | 345.2245
686.3733 | 7 | G | 2 | 232.1404
 | 8 | R | 1 | 175.1190
[Click] to move table

IGMEVTPSGTWLTYTGAIK

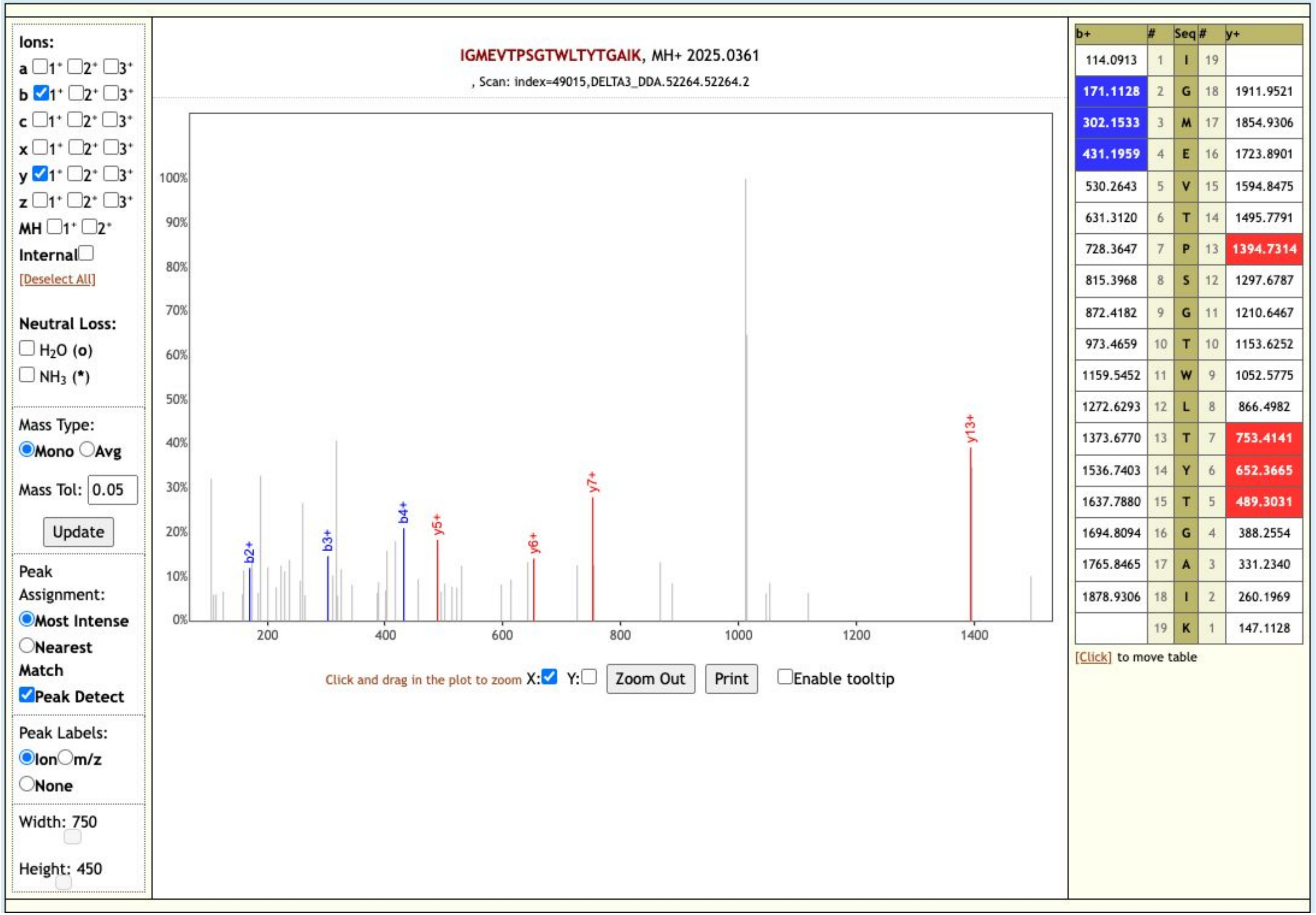

b+	#	Seq	#	y+
114.0913	1	I	19	
171.1128	2	G	18	1911.9521
302.1533	3	M	17	1854.9306
431.1959	4	E	16	1723.8901
530.2643	5	V	15	1594.8475
631.3120	6	T	14	1495.7791
728.3647	7	P	13	1394.7314
815.3968	8	S	12	1297.6787
872.4182	9	G	11	1210.6467
973.4659	10	T	10	1153.6252
1159.5452	11	W	9	1052.5775
1272.6293	12	L	8	866.4982
1373.6770	13	T	7	753.4141
1536.7403	14	Y	6	652.3665
1637.7880	15	T	5	489.3031
1694.8094	16	G	4	388.2554
1765.8465	17	A	3	331.2340
1878.9306	18	I	2	260.1969
	19	K	1	147.1128

[Click] to move table

ITFGGPSDSTGSNQNGER

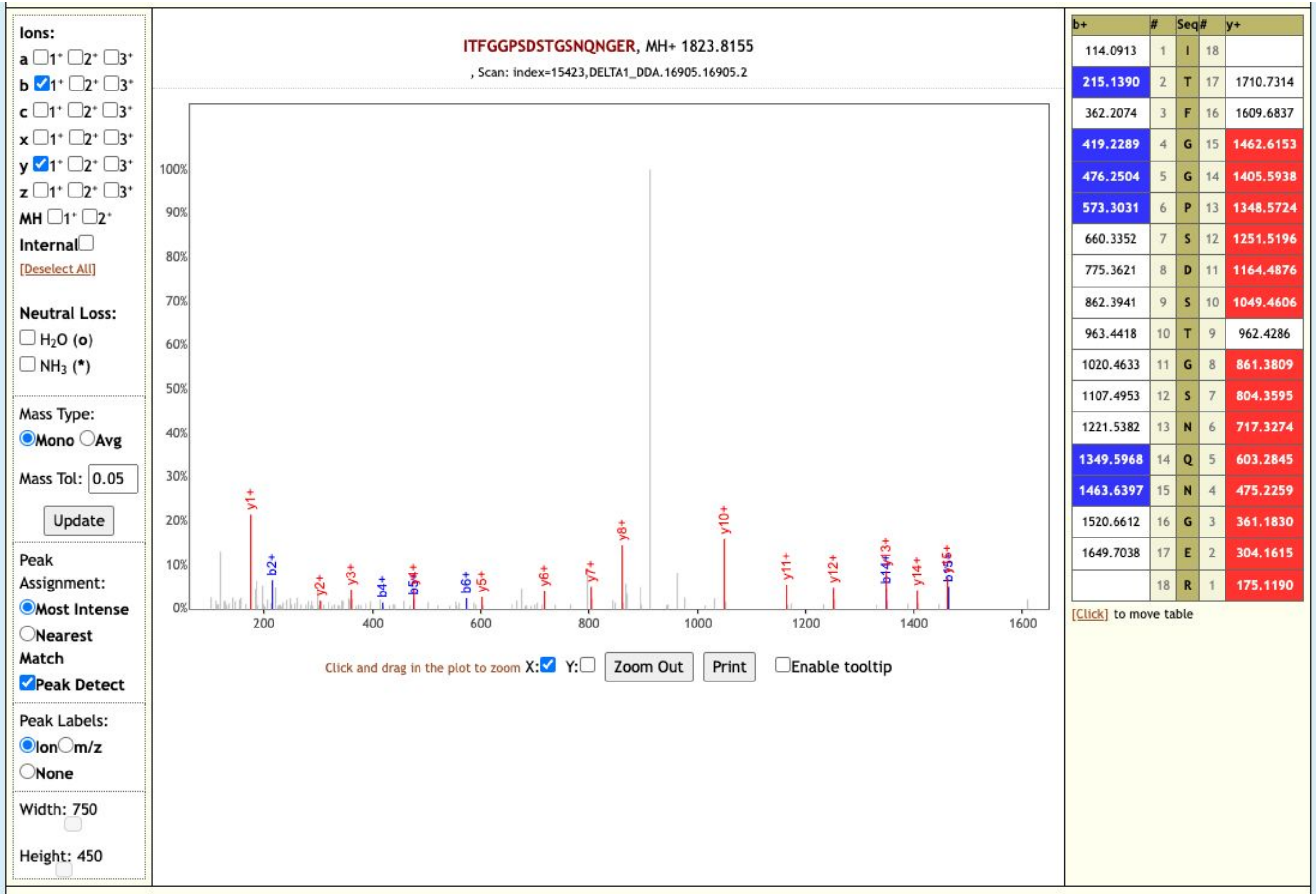

b+	#	Seq	#	y+
114.0913	1	I	18	
215.1390	2	T	17	1710.7314
362.2074	3	F	16	1609.6837
419.2289	4	G	15	1462.6153
476.2504	5	G	14	1405.5938
573.3031	6	P	13	1348.5724
660.3352	7	S	12	1251.5196
775.3621	8	D	11	1164.4876
862.3941	9	S	10	1049.4606
963.4418	10	T	9	962.4286
1020.4633	11	G	8	861.3809
1107.4953	12	S	7	804.3595
1221.5382	13	N	6	717.3274
1349.5968	14	Q	5	603.2845
1463.6397	15	N	4	475.2259
1520.6612	16	G	3	361.1830
1649.7038	17	E	2	304.1615
	18	R	1	175.1190

[Click] to move table

KAYETQALPQR

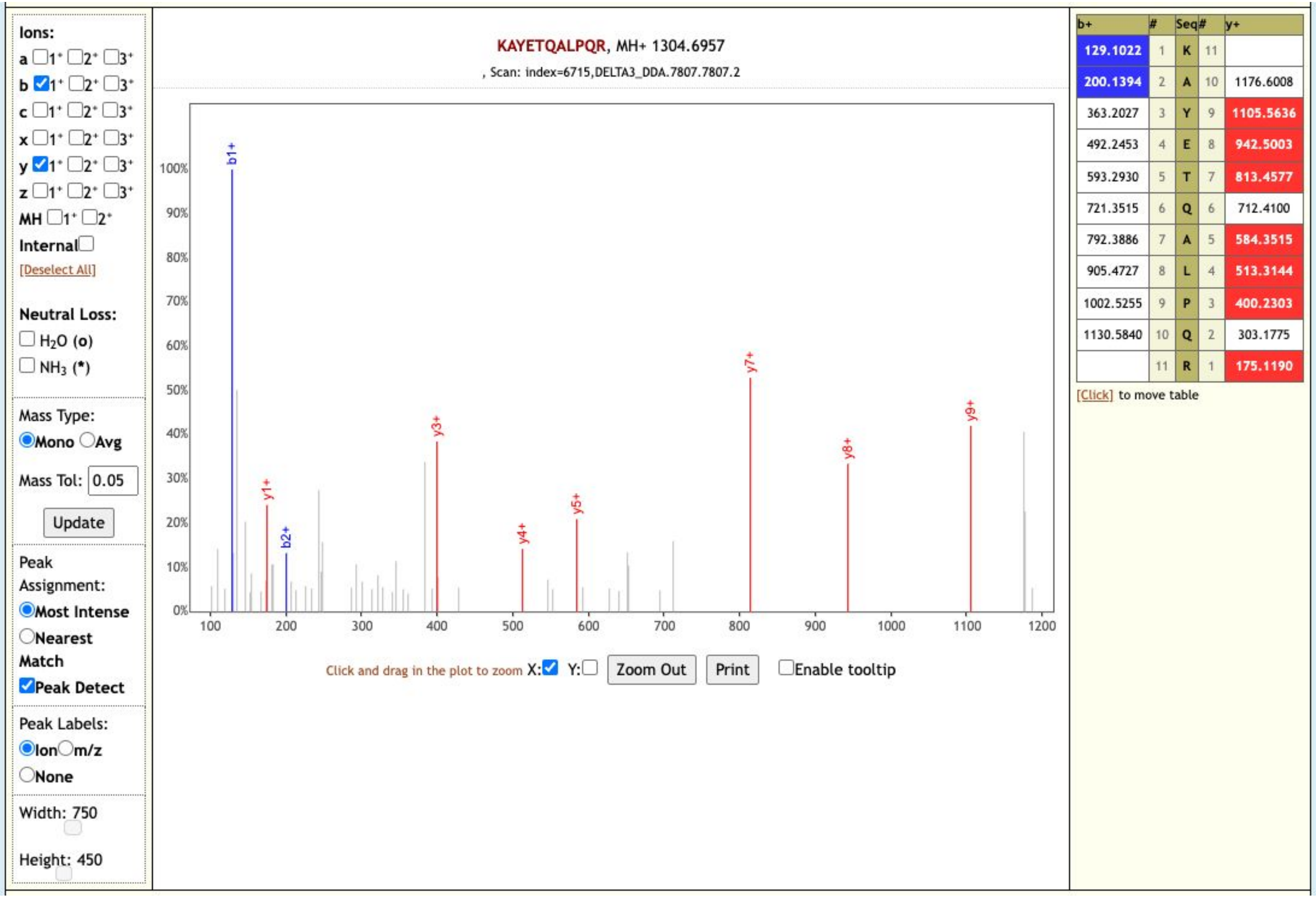

b+	#	Seq	#	y+
129.1022	1	K	11	
200.1394	2	A	10	1176.6008
363.2027	3	Y	9	1105.5636
492.2453	4	E	8	942.5003
593.2930	5	T	7	813.4577
721.3515	6	Q	6	712.4100
792.3886	7	A	5	584.3515
905.4727	8	L	4	513.3144
1002.5255	9	P	3	400.2303
1130.5840	10	Q	2	303.1775
	11	R	1	175.1190

[Click] to move table

HWPQIAQFAPSASAFFGMSR

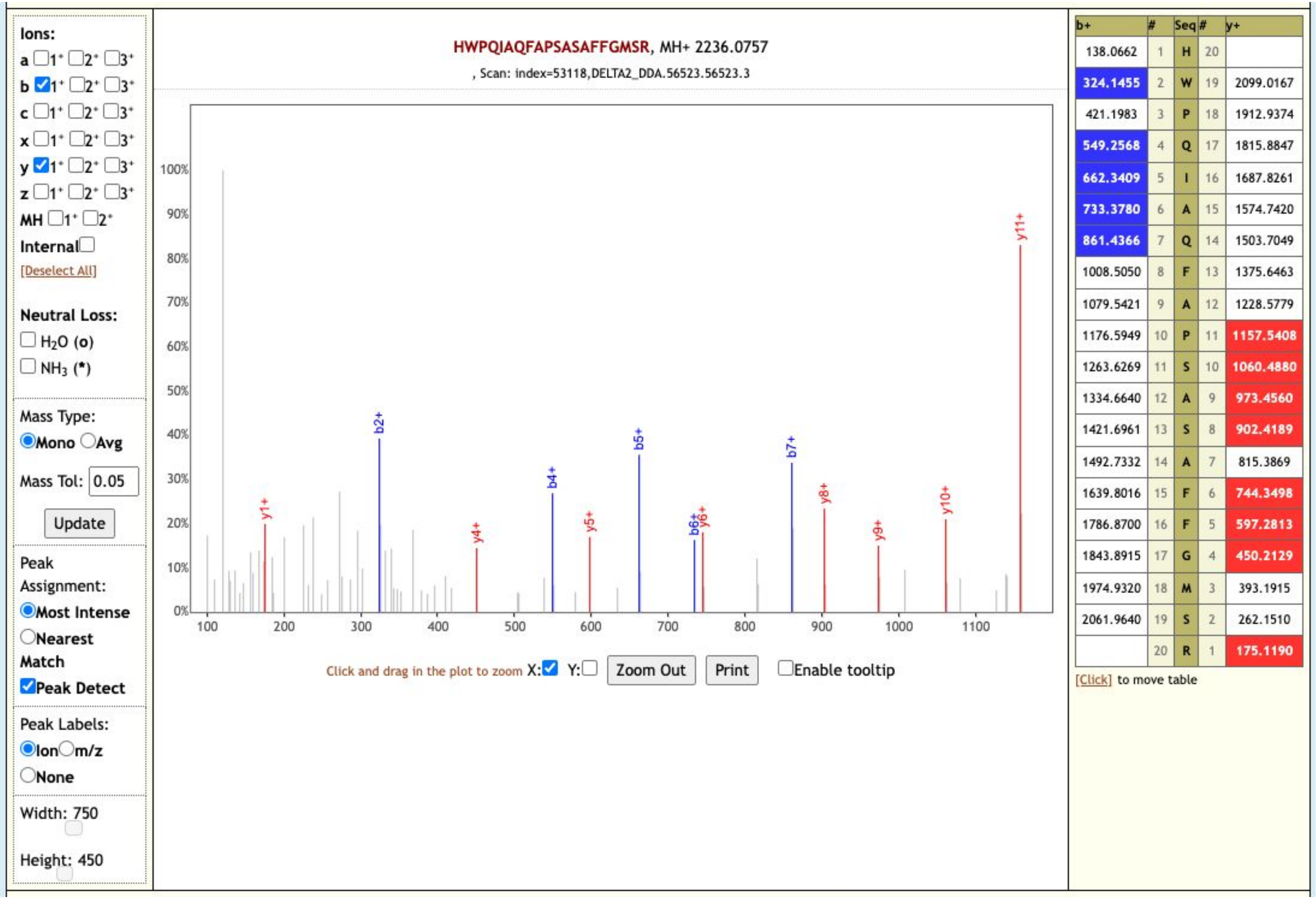

b+	#	Seq	#	y+
138.0662	1	H	20	
324.1455	2	W	19	2099.0167
421.1983	3	P	18	1912.9374
549.2568	4	Q	17	1815.8847
662.3409	5	I	16	1687.8261
733.3780	6	A	15	1574.7420
861.4366	7	Q	14	1503.7049
1008.5050	8	F	13	1375.6463
1079.5421	9	A	12	1228.5779
1176.5949	10	P	11	1157.5408
1263.6269	11	S	10	1060.4880
1334.6640	12	A	9	973.4560
1421.6961	13	S	8	902.4189
1492.7332	14	A	7	815.3869
1639.8016	15	F	6	744.3498
1786.8700	16	F	5	597.2813
1843.8915	17	G	4	450.2129
1974.9320	18	M	3	393.1915
2061.9640	19	S	2	262.1510
	20	R	1	175.1190

[Click] to move table

KSNLKPFER

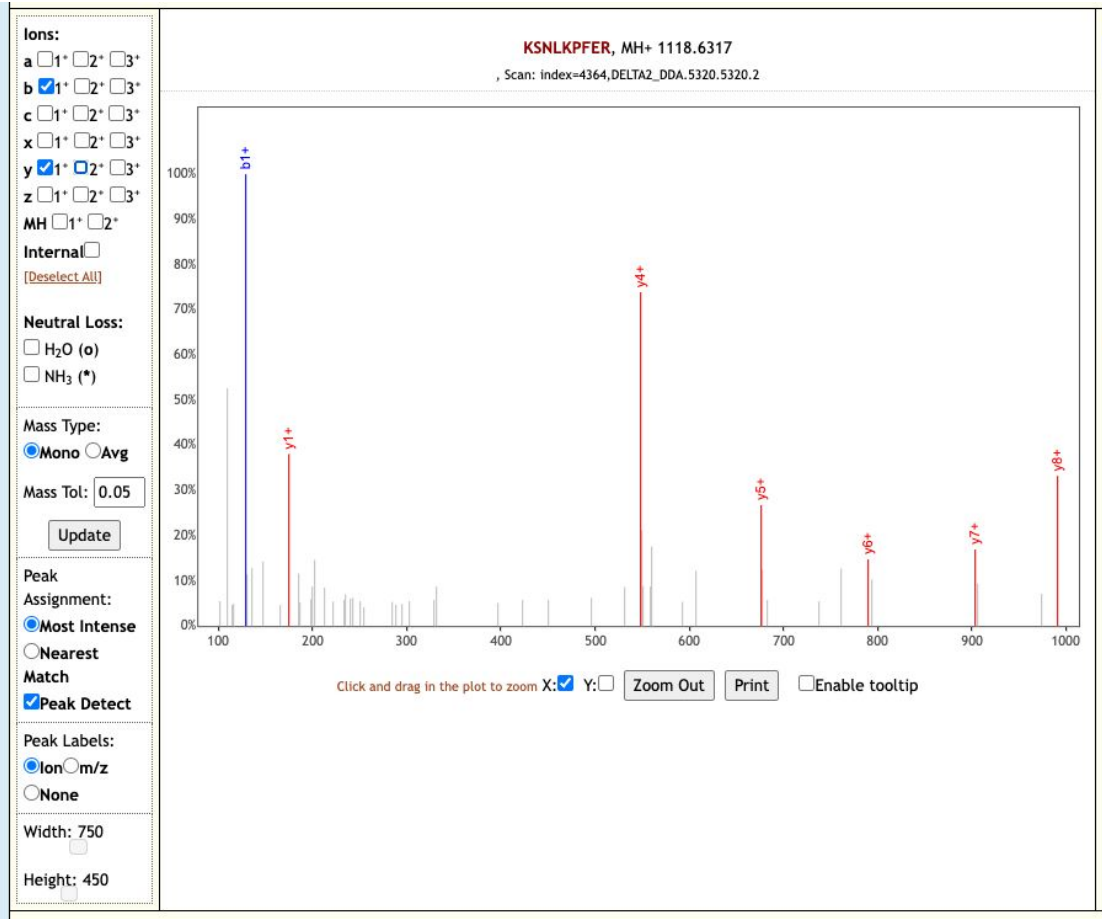

b+	#	Seq	#	y+
129.1022	1	K	9	
216.1343	2	S	8	990.5367
330.1772	3	N	7	903.5047
443.2613	4	L	6	789.4617
571.3562	5	K	5	676.3777
668.4090	6	P	4	548.2827
815.4774	7	F	3	451.2300
944.5200	8	E	2	304.1615
	9	R	1	175.1190

NSTPGSSMGTSPAR

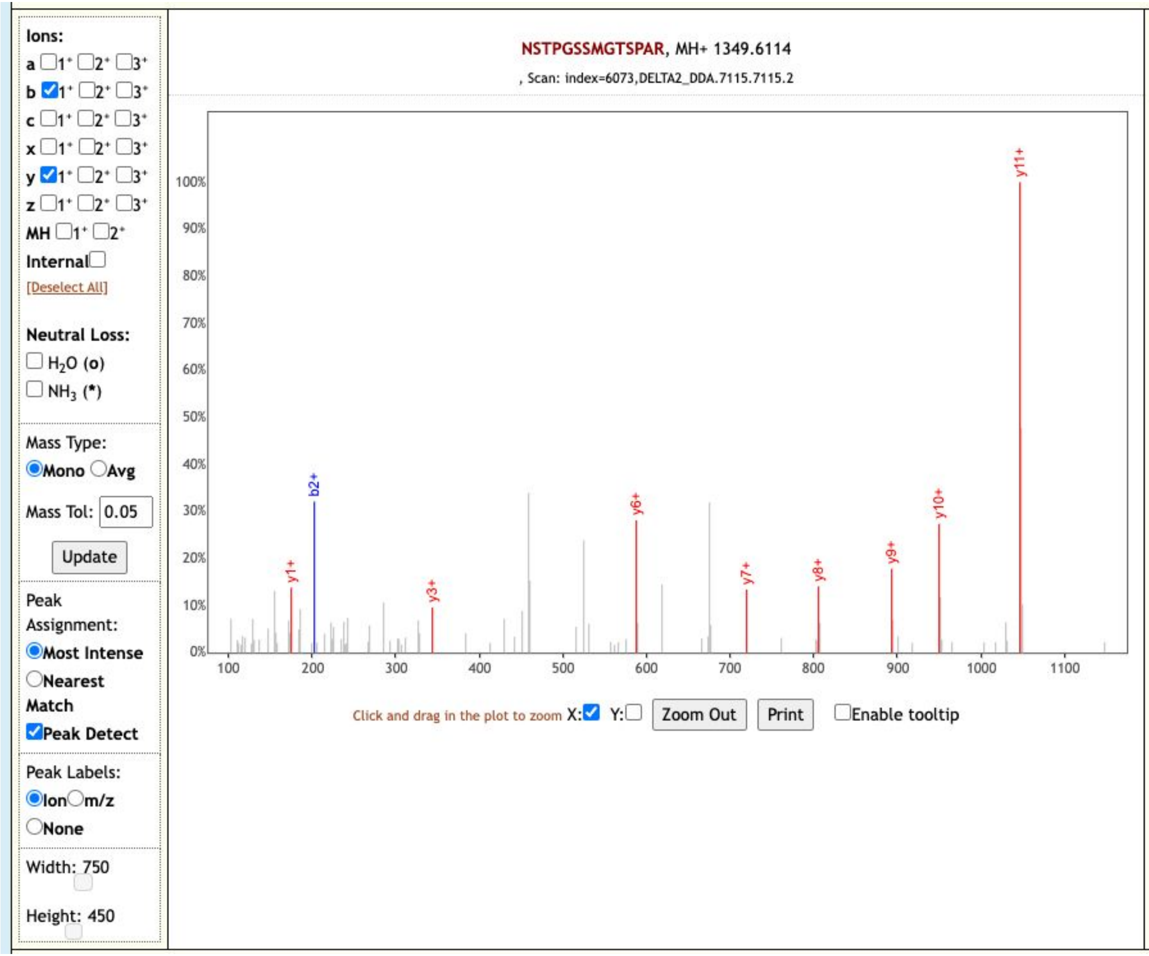

b+	#	Seq	#	y+
115.0502	1	N	14	
202.0822	2	S	13	1235.5685
303.1299	3	T	12	1148.5364
400.1827	4	P	11	1047.4888
457.2041	5	G	10	950.4360
544.2362	6	S	9	893.4145
631.2682	7	S	8	806.3825
762.3087	8	M	7	719.3505
819.3301	9	G	6	588.3100
920.3778	10	T	5	531.2885
1007.4099	11	S	4	430.2409
1104.4626	12	P	3	343.2088
1175.4997	13	A	2	246.1561
	14	R	1	175.1190

[Click] to move table

GPEQTQGNFGDQELIR

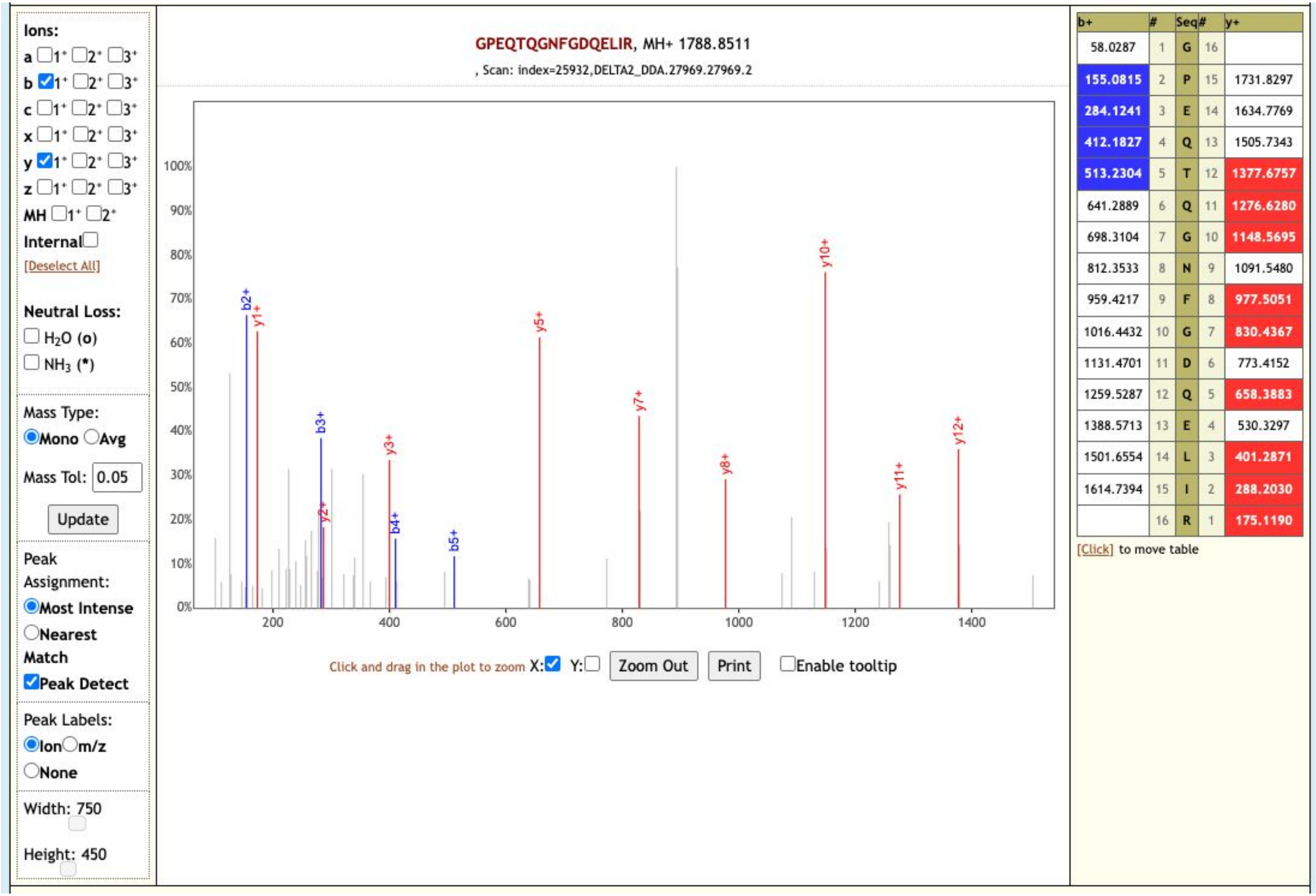

b+	#	Seq	#	y+
58.0287	1	G	16	
155.0815	2	P	15	1731.8297
284.1241	3	E	14	1634.7769
412.1827	4	Q	13	1505.7343
513.2304	5	T	12	1377.6757
641.2889	6	Q	11	1276.6280
698.3104	7	G	10	1148.5695
812.3533	8	N	9	1091.5480
959.4217	9	F	8	977.5051
1016.4432	10	G	7	830.4367
1131.4701	11	D	6	773.4152
1259.5287	12	Q	5	658.3883
1388.5713	13	E	4	530.3297
1501.6554	14	L	3	401.2871
1614.7394	15	I	2	288.2030
	16	R	1	175.1190

[Click] to move table

VAGDSGFAAYSR

Ions:
a 1+ 2+ 3+
b ☑1+ 2+ 3+
c 1+ 2+ 3+
x 1+ 2+ 3+
y ☑1+ 2+ 3+
z 1+ 2+ 3+
MH 1+ 2+
Internal
[Deselect All]

Neutral Loss:
H_2O (o)
NH_3 (*)

Mass Type:
◉Mono ○Avg

Mass Tol: 0.05

Update

Peak Assignment:
◉Most Intense
○Nearest Match
☑Peak Detect

Peak Labels:
◉Ion ○m/z
○None

Width: 750

Height: 450

VAGDSGFAAYSR, MH+ 1200.5644

, Scan: index=16726,DELTA1_DDA.18273.18273.2

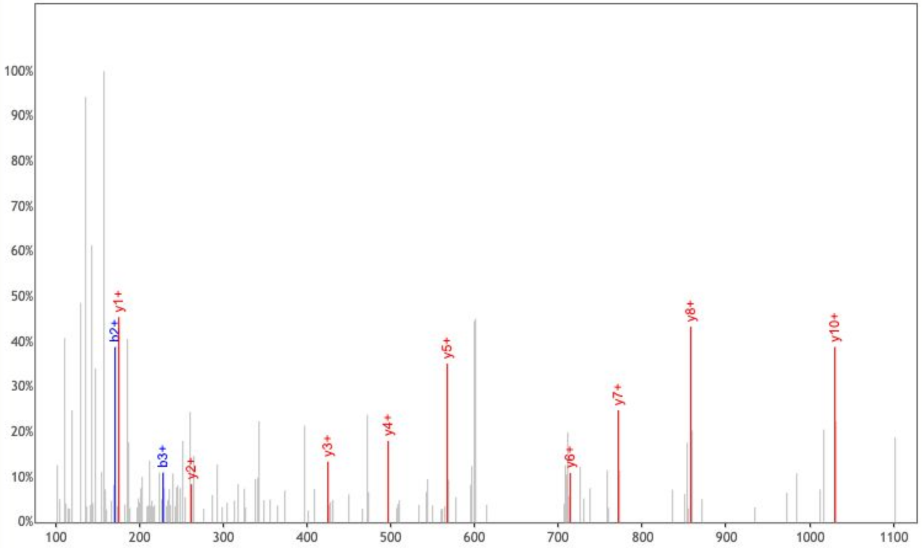

Click and drag in the plot to zoom X:☑ Y: Zoom Out Print Enable tooltip

b+	#	Seq	#	y+
100.0757	1	V	12	
171.1128	2	A	11	1101.4960
228.1343	3	G	10	1030.4588
343.1612	4	D	9	973.4374
430.1932	5	S	8	858.4104
487.2147	6	G	7	771.3784
634.2831	7	F	6	714.3570
705.3202	8	A	5	567.2885
776.3573	9	A	4	496.2514
939.4207	10	Y	3	425.2143
1026.4527	11	S	2	262.1510
	12	R	1	175.1190

[Click] to move table

LVDPQIQLAVTR

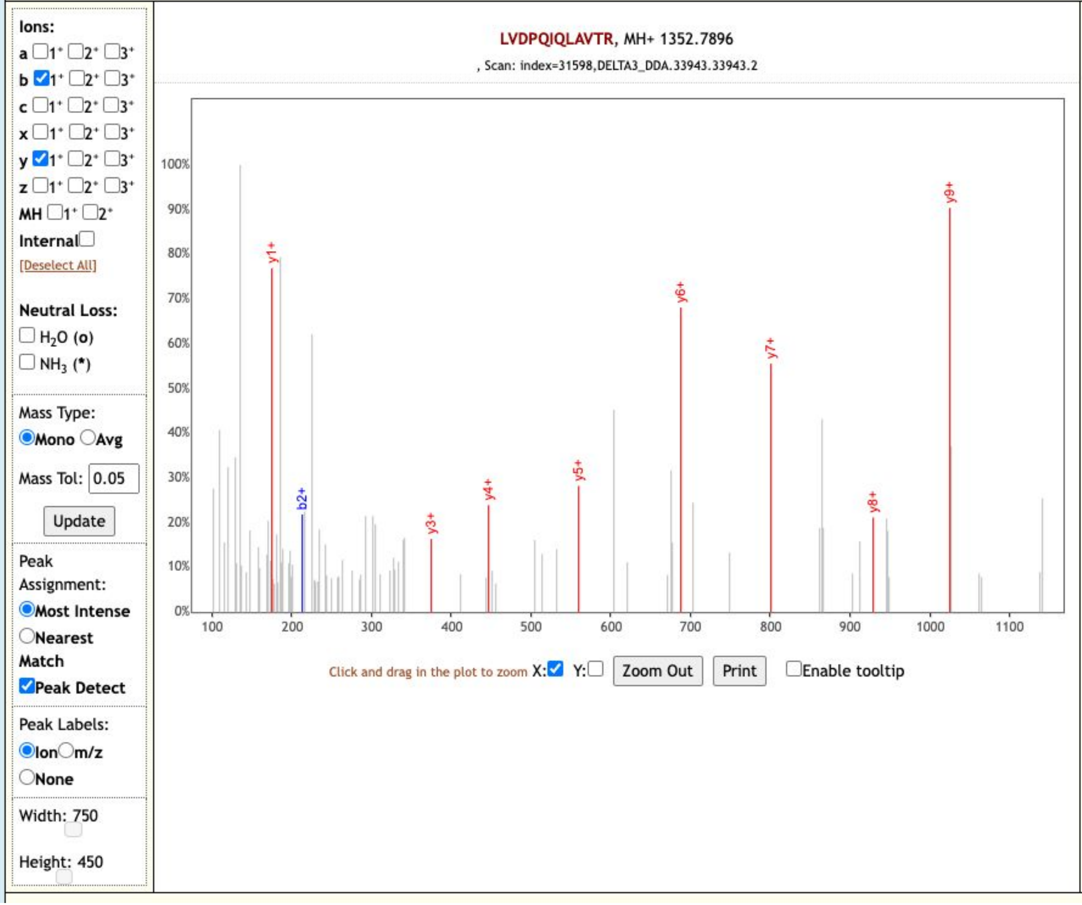

b+	#	Seq	#	y+
114.0913	1	L	12	
213.1598	2	V	11	1239.7056
328.1867	3	D	10	1140.6371
425.2395	4	P	9	1025.6102
553.2980	5	Q	8	928.5574
666.3821	6	I	7	800.4989
794.4407	7	Q	6	687.4148
907.5247	8	L	5	559.3562
978.5619	9	A	4	446.2722
1077.6303	10	V	3	375.2350
1178.6780	11	T	2	276.1666
	12	R	1	175.1190

[Click] to move table

YLGTGPEAGLPYGANK

Ions:
a ☐1⁺ ☐2⁺ ☐3⁺
b ☑1⁺ ☐2⁺ ☐3⁺
c ☐1⁺ ☐2⁺ ☐3⁺
x ☐1⁺ ☐2⁺ ☐3⁺
y ☑1⁺ ☐2⁺ ☐3⁺
z ☐1⁺ ☐2⁺ ☐3⁺
MH ☐1⁺ ☐2⁺
Internal ☐
[Deselect All]

Neutral Loss:
☐ H_2O (o)
☐ NH_3 (*)

Mass Type:
◉Mono ○Avg

Mass Tol: 0.05

Update

Peak Assignment:
◉Most Intense
○Nearest Match
☑Peak Detect

Peak Labels:
◉Ion ○m/z
○None

Width: 750

Height: 450

YLGTGPEAGLPYGANK, MH+ 1607.8064
, Scan: index=26680,DELTA5_DDA.28768.28768.2

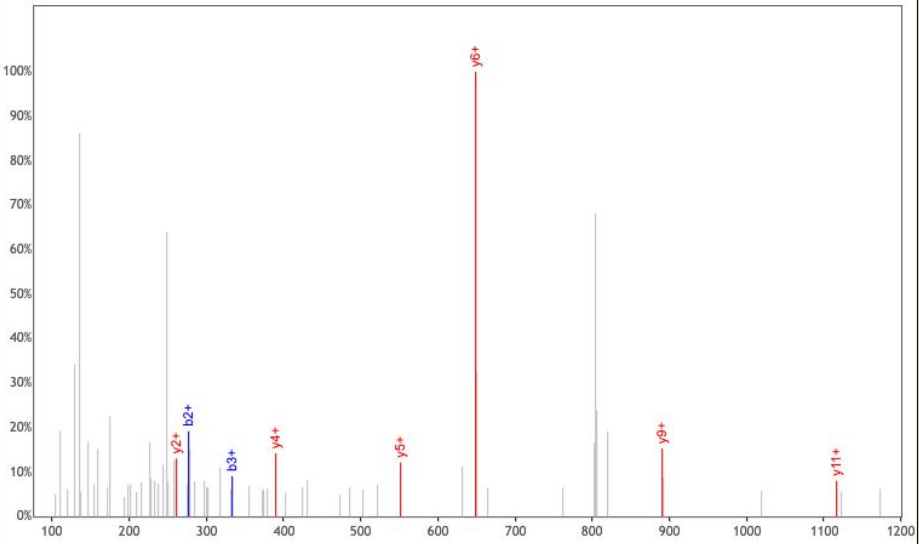

Click and drag in the plot to zoom X:☑ Y:☐ Zoom Out Print ☐Enable tooltip

b+	#	Seq	#	y+
164.0706	1	Y	16	
277.1547	2	L	15	1444.7431
334.1761	3	G	14	1331.6590
435.2238	4	T	13	1274.6375
492.2453	5	G	12	1173.5899
589.2980	6	P	11	1116.5684
718.3406	7	E	10	1019.5156
789.3777	8	A	9	890.4730
846.3992	9	G	8	819.4359
959.4833	10	L	7	762.4145
1056.5360	11	P	6	649.3304
1219.5994	12	Y	5	552.2776
1276.6208	13	G	4	389.2143
1347.6579	14	A	3	332.1928
1461.7009	15	N	2	261.1557
	16	K	1	147.1128

[Click] to move table

VYSTGSNVFQTR

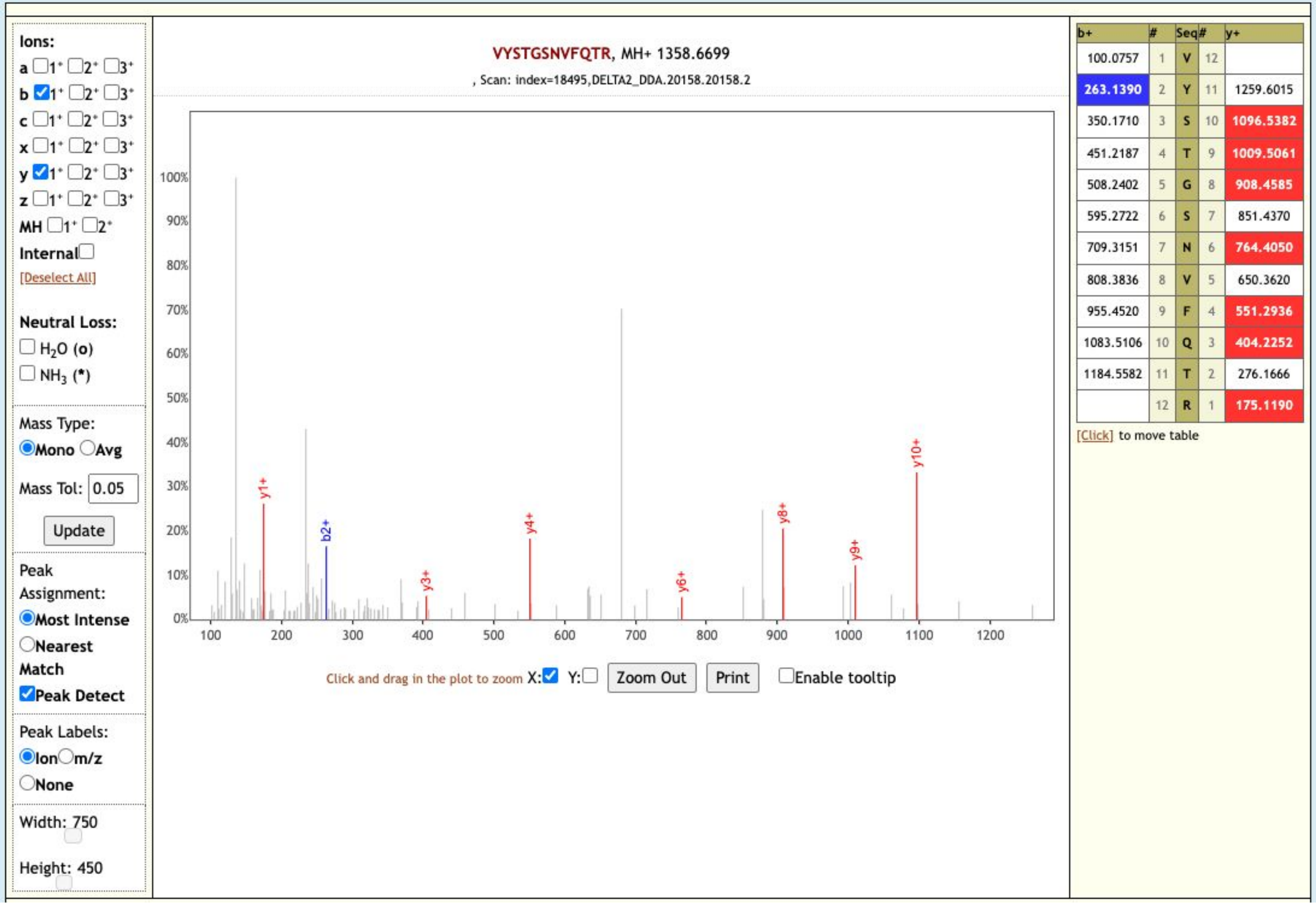

b+	#	Seq	#	y+
100.0757	1	V	12	
263.1390	2	Y	11	1259.6015
350.1710	3	S	10	1096.5382
451.2187	4	T	9	1009.5061
508.2402	5	G	8	908.4585
595.2722	6	S	7	851.4370
709.3151	7	N	6	764.4050
808.3836	8	V	5	650.3620
955.4520	9	F	4	551.2936
1083.5106	10	Q	3	404.2252
1184.5582	11	T	2	276.1666
	12	R	1	175.1190

VTLADAGFIK

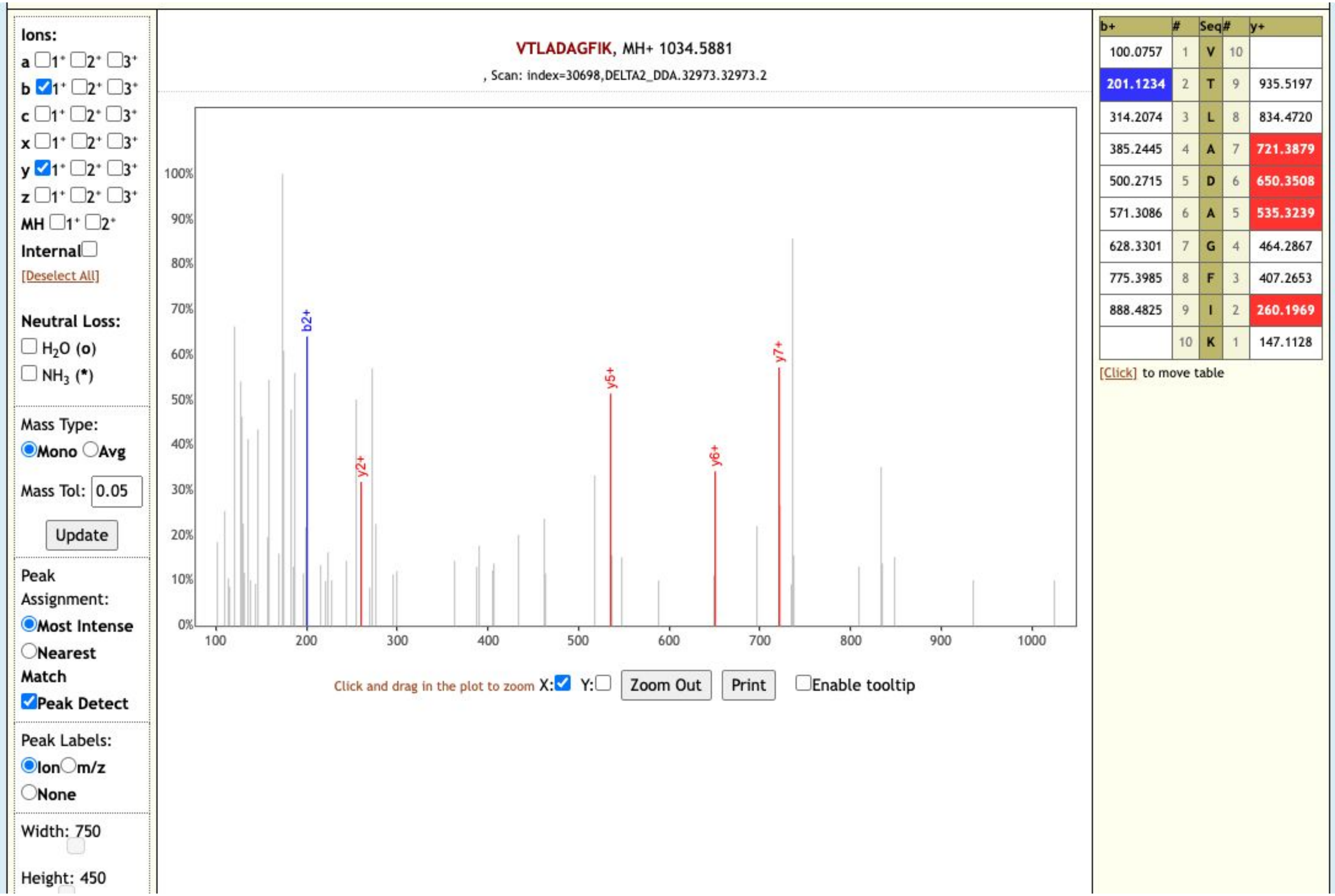

b+	#	Seq	#	y+
100.0757	1	V	10	
201.1234	2	T	9	935.5197
314.2074	3	L	8	834.4720
385.2445	4	A	7	721.3879
500.2715	5	D	6	650.3508
571.3086	6	A	5	535.3239
628.3301	7	G	4	464.2867
775.3985	8	F	3	407.2653
888.4825	9	I	2	260.1969
	10	K	1	147.1128

[Click] to move table

LGSPLSLNMAR

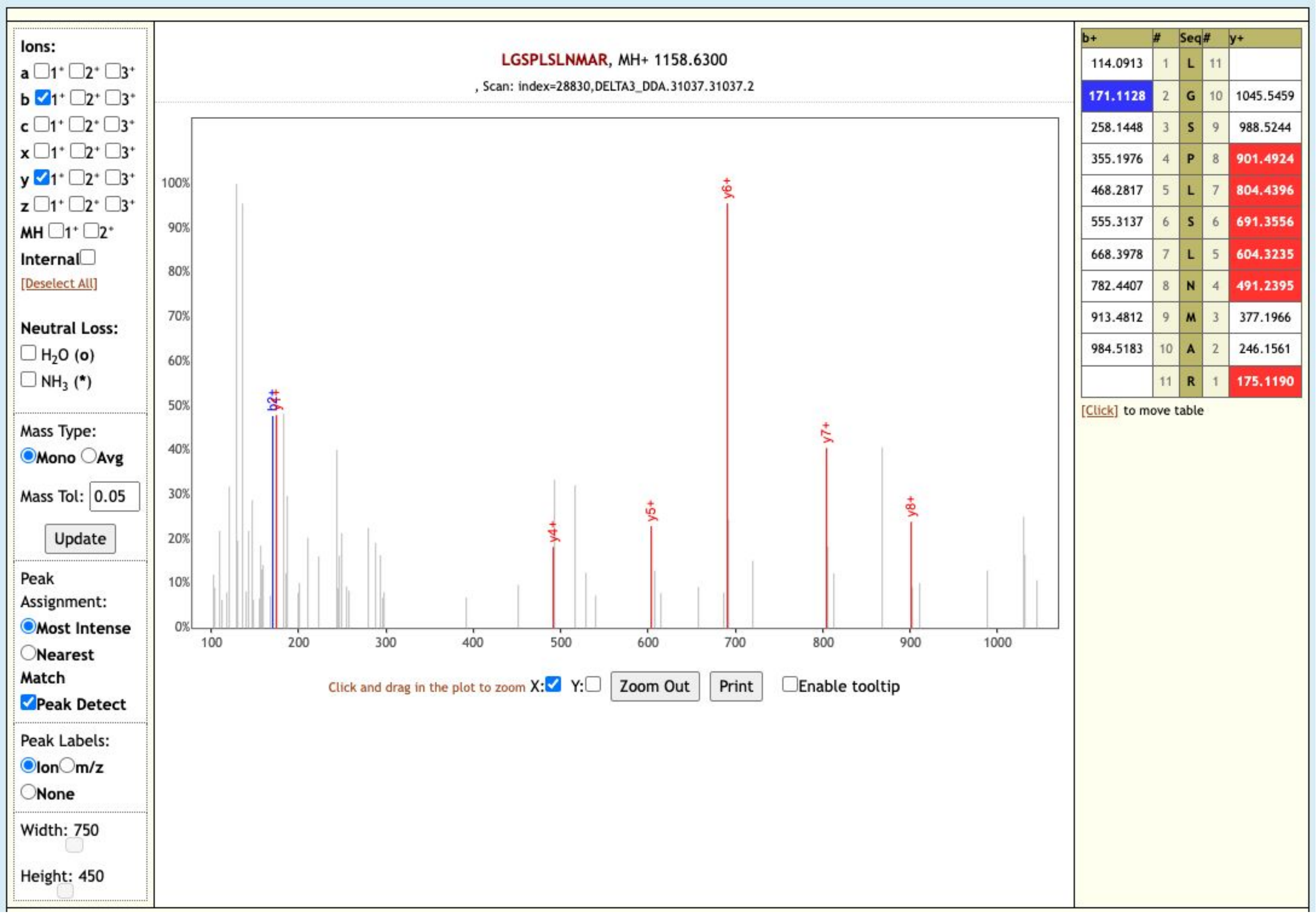

Omicron

AYNVTQAFGR

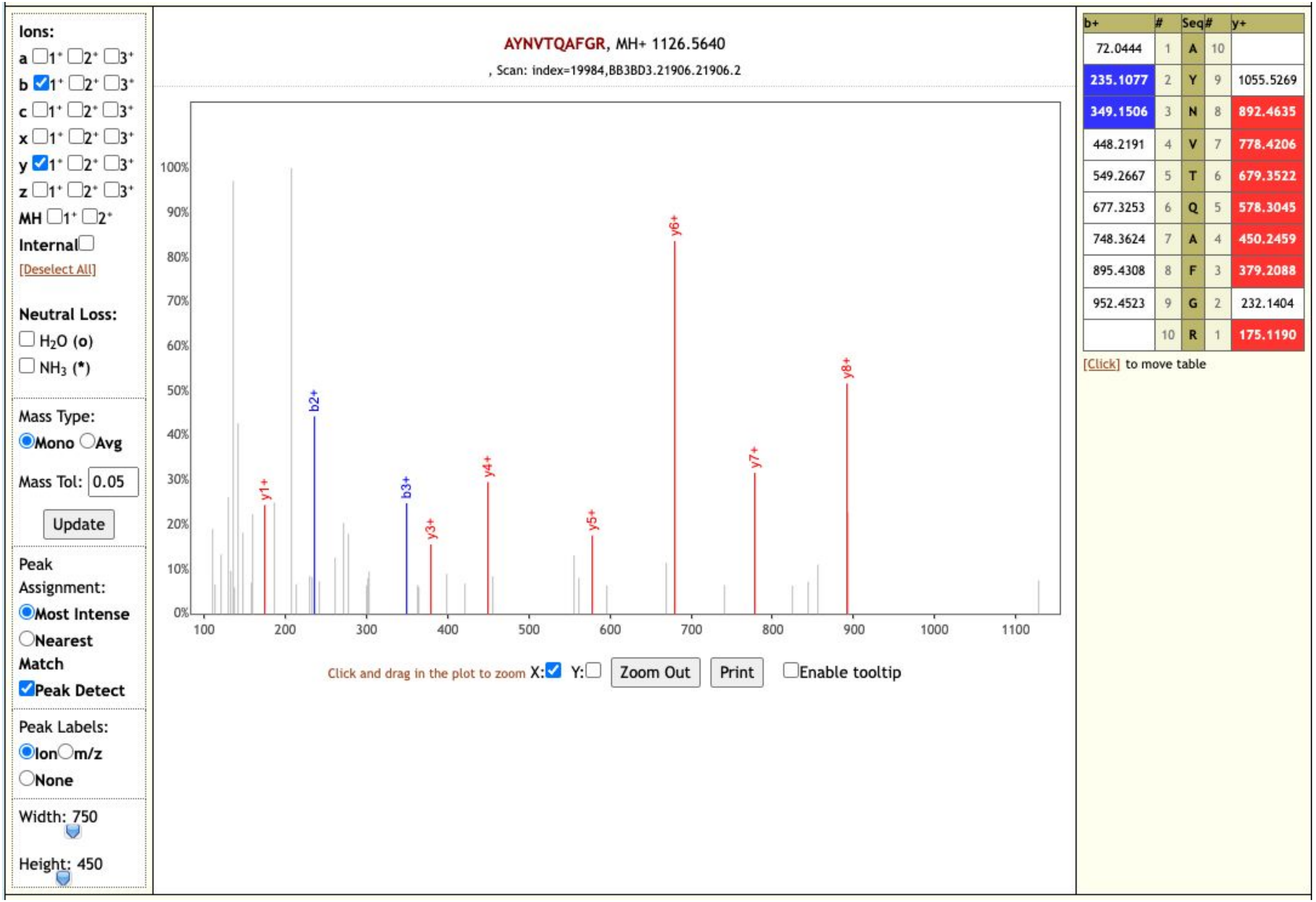

b+	#	Seq	#	y+
72.0444	1	A	10	
235.1077	2	Y	9	1055.5269
349.1506	3	N	8	892.4635
448.2191	4	V	7	778.4206
549.2667	5	T	6	679.3522
677.3253	6	Q	5	578.3045
748.3624	7	A	4	450.2459
895.4308	8	F	3	379.2088
952.4523	9	G	2	232.1404
	10	R	1	175.1190

[Click] to move table

NPANNAAIVIQLPQGTTLPK

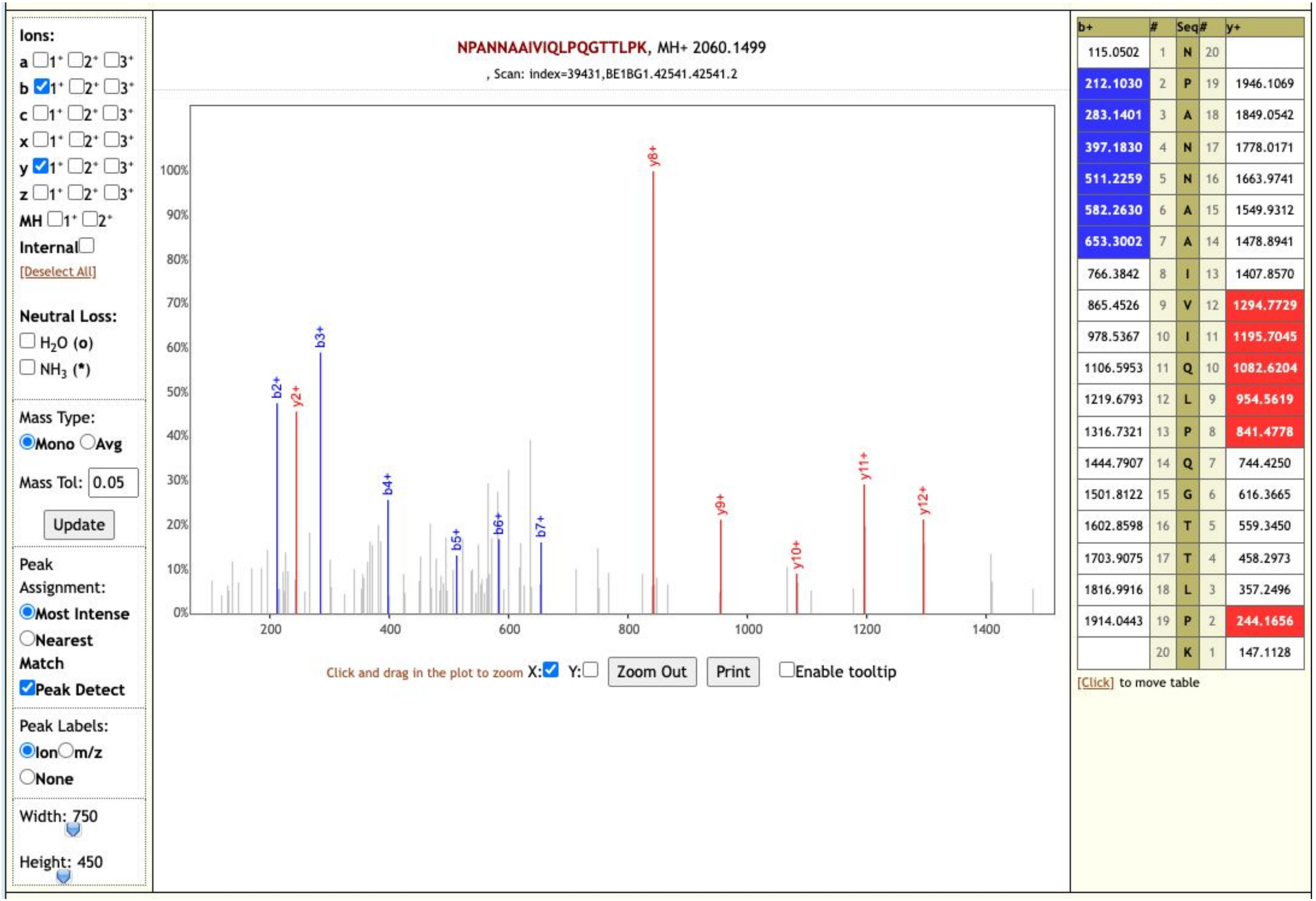

b+	#	Seq	#	y+
115.0502	1	N	20	
212.1030	2	P	19	1946.1069
283.1401	3	A	18	1849.0542
397.1830	4	N	17	1778.0171
511.2259	5	N	16	1663.9741
582.2630	6	A	15	1549.9312
653.3002	7	A	14	1478.8941
766.3842	8	I	13	1407.8570
865.4526	9	V	12	1294.7729
978.5367	10	I	11	1195.7045
1106.5953	11	Q	10	1082.6204
1219.6793	12	L	9	954.5619
1316.7321	13	P	8	841.4778
1444.7907	14	Q	7	744.4250
1501.8122	15	G	6	616.3665
1602.8598	16	T	5	559.3450
1703.9075	17	T	4	458.2973
1816.9916	18	L	3	357.2496
1914.0443	19	P	2	244.1656
	20	K	1	147.1128

[Click] to move table

DGIIWVATEGALNTPK

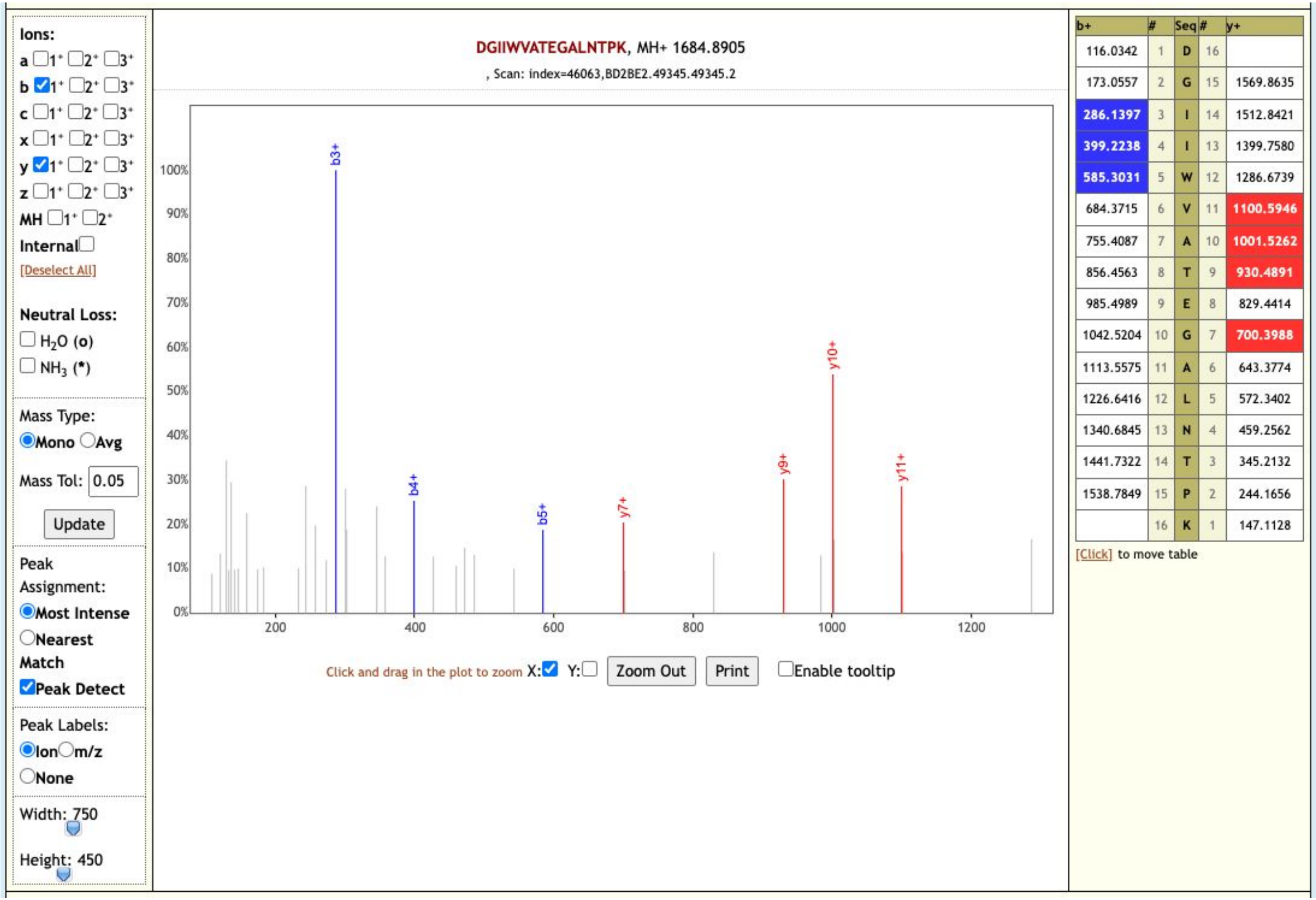

b+	#	Seq	#	y+
116.0342	1	D	16	
173.0557	2	G	15	1569.8635
286.1397	3	I	14	1512.8421
399.2238	4	I	13	1399.7580
585.3031	5	W	12	1286.6739
684.3715	6	V	11	1100.5946
755.4087	7	A	10	1001.5262
856.4563	8	T	9	930.4891
985.4989	9	E	8	829.4414
1042.5204	10	G	7	700.3988
1113.5575	11	A	6	643.3774
1226.6416	12	L	5	572.3402
1340.6845	13	N	4	459.2562
1441.7322	14	T	3	345.2132
1538.7849	15	P	2	244.1656
	16	K	1	147.1128

[Click] to move table

KADETQALPQR

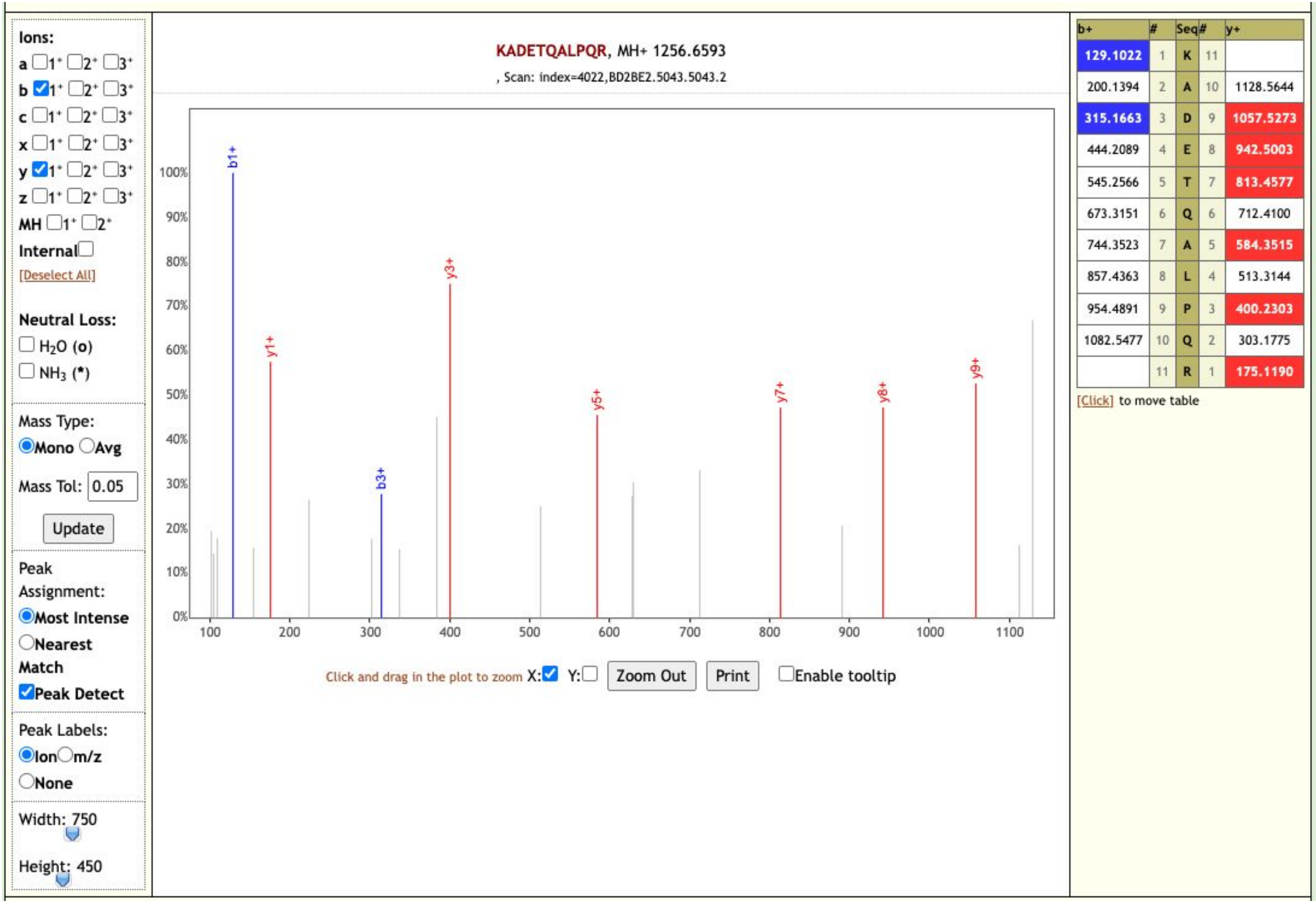

b+	#	Seq	#	y+
129.1022	1	K	11	
200.1394	2	A	10	1128.5644
315.1663	3	D	9	1057.5273
444.2089	4	E	8	942.5003
545.2566	5	T	7	813.4577
673.3151	6	Q	6	712.4100
744.3523	7	A	5	584.3515
857.4363	8	L	4	513.3144
954.4891	9	P	3	400.2303
1082.5477	10	Q	2	303.1775
	11	R	1	175.1190

ITFGGPSDSTGSNQNGGAR

Ions:
a 1+ 2+ 3+
b 1+ 2+ 3+
c 1+ 2+ 3+
x 1+ 2+ 3+
y 1+ 2+ 3+
z 1+ 2+ 3+
MH 1+ 2+
Internal
[Deselect All]

Neutral Loss:
H_2O (o)
NH_3 (*)

Mass Type:
Mono Avg

Mass Tol: 0.05

Update

Peak Assignment:
Most Intense
Nearest Match
Peak Detect

Peak Labels:
Ion m/z
None

Width: 750

Height: 450

ITFGGPSDSTGSNQNGGAR, MH+ 1822.8314

, Scan: index=12269,BE1BG1.13906.13906.2

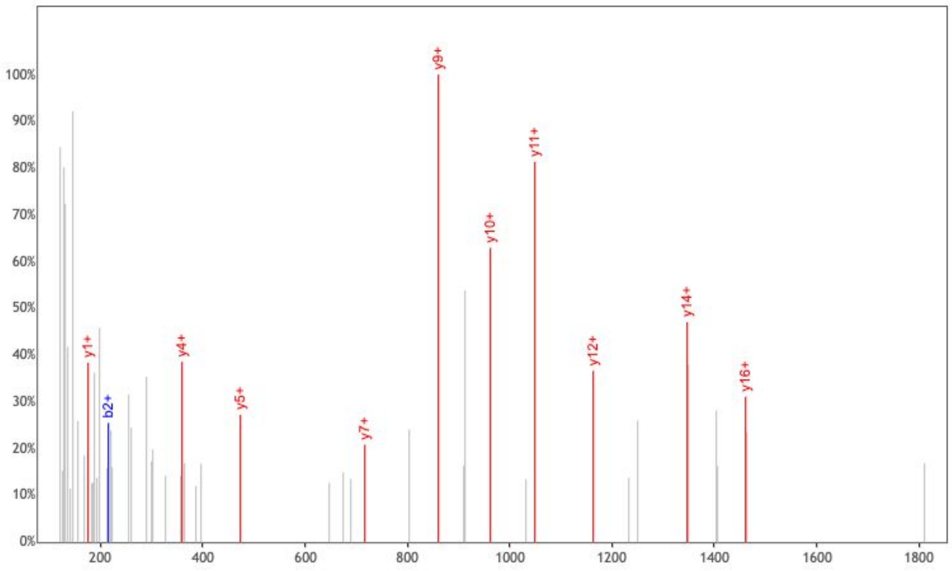

Click and drag in the plot to zoom X: Y: Zoom Out Print Enable tooltip

b+	#	Seq	#	y+
114.0913	1	I	19	
215.1390	2	T	18	1709.7474
362.2074	3	F	17	1608.6997
419.2289	4	G	16	1461.6313
476.2504	5	G	15	1404.6098
573.3031	6	P	14	1347.5884
660.3352	7	S	13	1250.5356
775.3621	8	D	12	1163.5036
862.3941	9	S	11	1048.4766
963.4418	10	T	10	961.4446
1020.4633	11	G	9	860.3969
1107.4953	12	S	8	803.3755
1221.5382	13	N	7	716.3434
1349.5968	14	Q	6	602.3005
1463.6397	15	N	5	474.2419
1520.6612	16	G	4	360.1990
1577.6827	17	G	3	303.1775
1648.7198	18	A	2	246.1561
	19	R	1	175.1190

[Click] to move table

QQTVTLLPAADLDDFSK

Ions:
a 1+ 2+ 3+
b 1+ 2+ 3+
c 1+ 2+ 3+
x 1+ 2+ 3+
y 1+ 2+ 3+
z 1+ 2+ 3+
MH 1+ 2+
Internal
[Deselect All]

Neutral Loss:
H_2O (o)
NH_3 (*)

Mass Type:
Mono Avg

Mass Tol: 0.05

Update

Peak Assignment:
Most Intense
Nearest Match
Peak Detect

Peak Labels:
Ion m/z
None

Width: 750

Height: 450

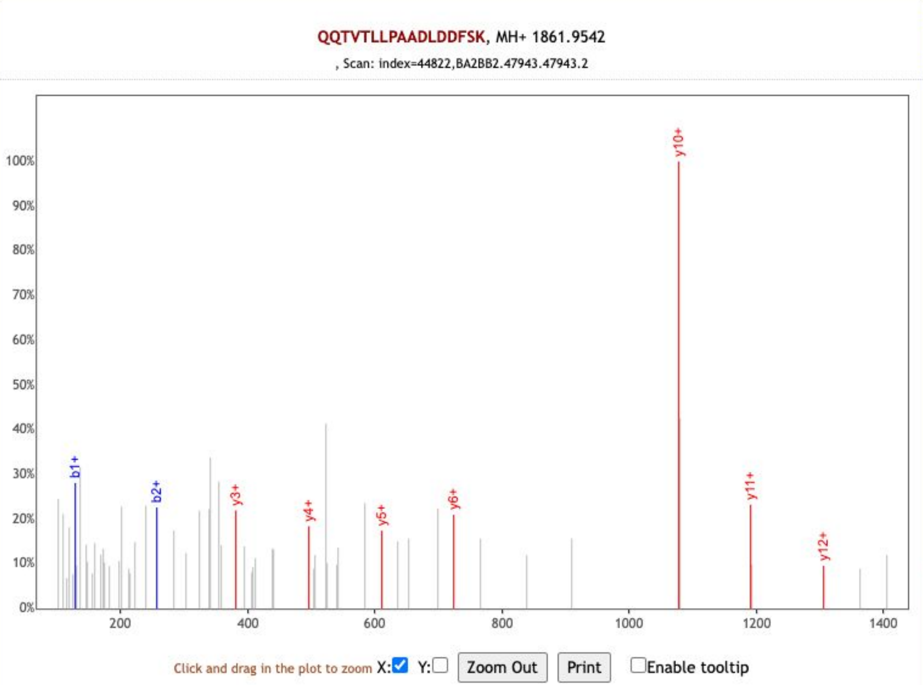

b+	#	Seq	#	y+
129.0659	1	Q	17	
257.1244	2	Q	16	1733.8956
358.1721	3	T	15	1605.8370
457.2405	4	V	14	1504.7894
558.2882	5	T	13	1405.7209
671.3723	6	L	12	1304.6733
784.4563	7	L	11	1191.5892
881.5091	8	P	10	1078.5051
952.5462	9	A	9	981.4524
1023.5833	10	A	8	910.4153
1138.6103	11	D	7	839.3781
1251.6943	12	L	6	724.3512
1366.7213	13	D	5	611.2671
1481.7482	14	D	4	496.2402
1628.8166	15	F	3	381.2132
1715.8487	16	S	2	234.1448
	17	K	1	147.1128

[Click] to move table

HWPQIAQFAPSASAFF

Ions:
a 1+ 2+ 3+
b ☑1+ 2+ 3+
c 1+ 2+ 3+
x 1+ 2+ 3+
y ☑1+ 2+ 3+
z 1+ 2+ 3+
MH 1+ 2+
Internal
[Deselect All]

Neutral Loss:
H_2O (o)
NH_3 (*)

Mass Type:
Mono Avg

Mass Tol: 0.05

Update

Peak Assignment:
Most Intense
Nearest Match
☑Peak Detect

Peak Labels:
Ion m/z
None

Width: 750

Height: 450

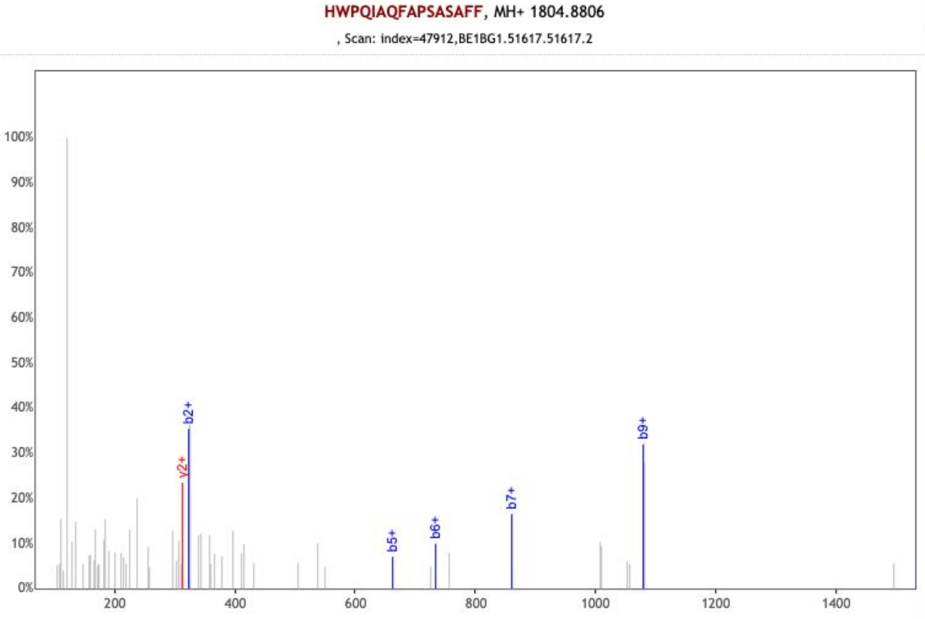

Click and drag in the plot to zoom X:☑ Y: Zoom Out Print Enable tooltip

b+	#	Seq	#	y+
138.0662	1	H	16	
324.1455	2	W	15	1667.8217
421.1983	3	P	14	1481.7423
549.2568	4	Q	13	1384.6896
662.3409	5	I	12	1256.6310
733.3780	6	A	11	1143.5469
861.4366	7	Q	10	1072.5098
1008.5050	8	F	9	944.4512
1079.5421	9	A	8	797.3828
1176.5949	10	P	7	726.3457
1263.6269	11	S	6	629.2930
1334.6640	12	A	5	542.2609
1421.6961	13	S	4	471.2238
1492.7332	14	A	3	384.1918
1639.8016	15	F	2	313.1547
	16	F	1	166.0863

[Click] to move table

GQGVPINTNSSPDDQIGYYR

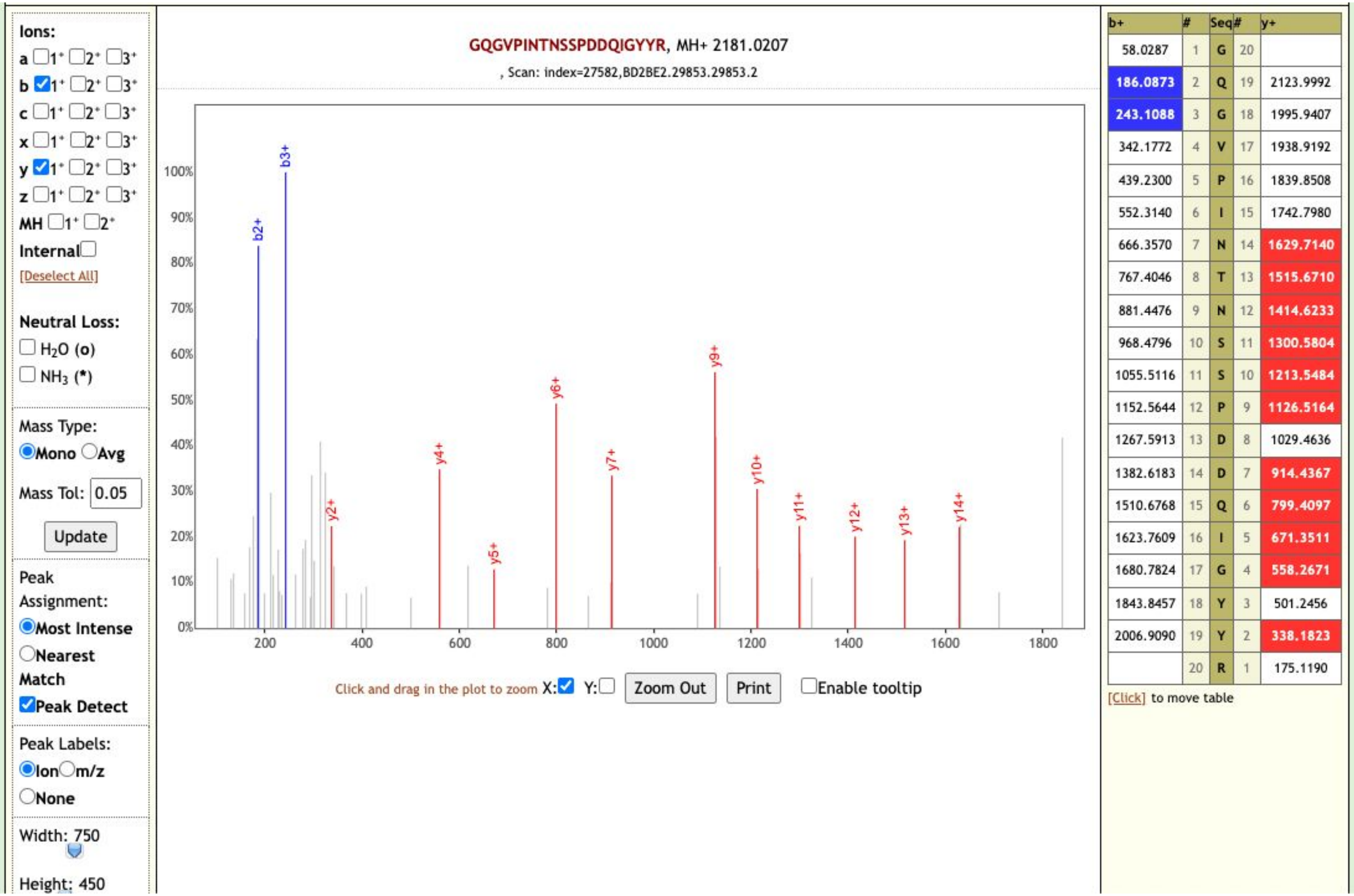

b+	#	Seq	#	y+
58.0287	1	G	20	
186.0873	2	Q	19	2123.9992
243.1088	3	G	18	1995.9407
342.1772	4	V	17	1938.9192
439.2300	5	P	16	1839.8508
552.3140	6	I	15	1742.7980
666.3570	7	N	14	1629.7140
767.4046	8	T	13	1515.6710
881.4476	9	N	12	1414.6233
968.4796	10	S	11	1300.5804
1055.5116	11	S	10	1213.5484
1152.5644	12	P	9	1126.5164
1267.5913	13	D	8	1029.4636
1382.6183	14	D	7	914.4367
1510.6768	15	Q	6	799.4097
1623.7609	16	I	5	671.3511
1680.7824	17	G	4	558.2671
1843.8457	18	Y	3	501.2456
2006.9090	19	Y	2	338.1823
	20	R	1	175.1190

[Click] to move table

MAGNGGDAALALLLLDRLNQLESK

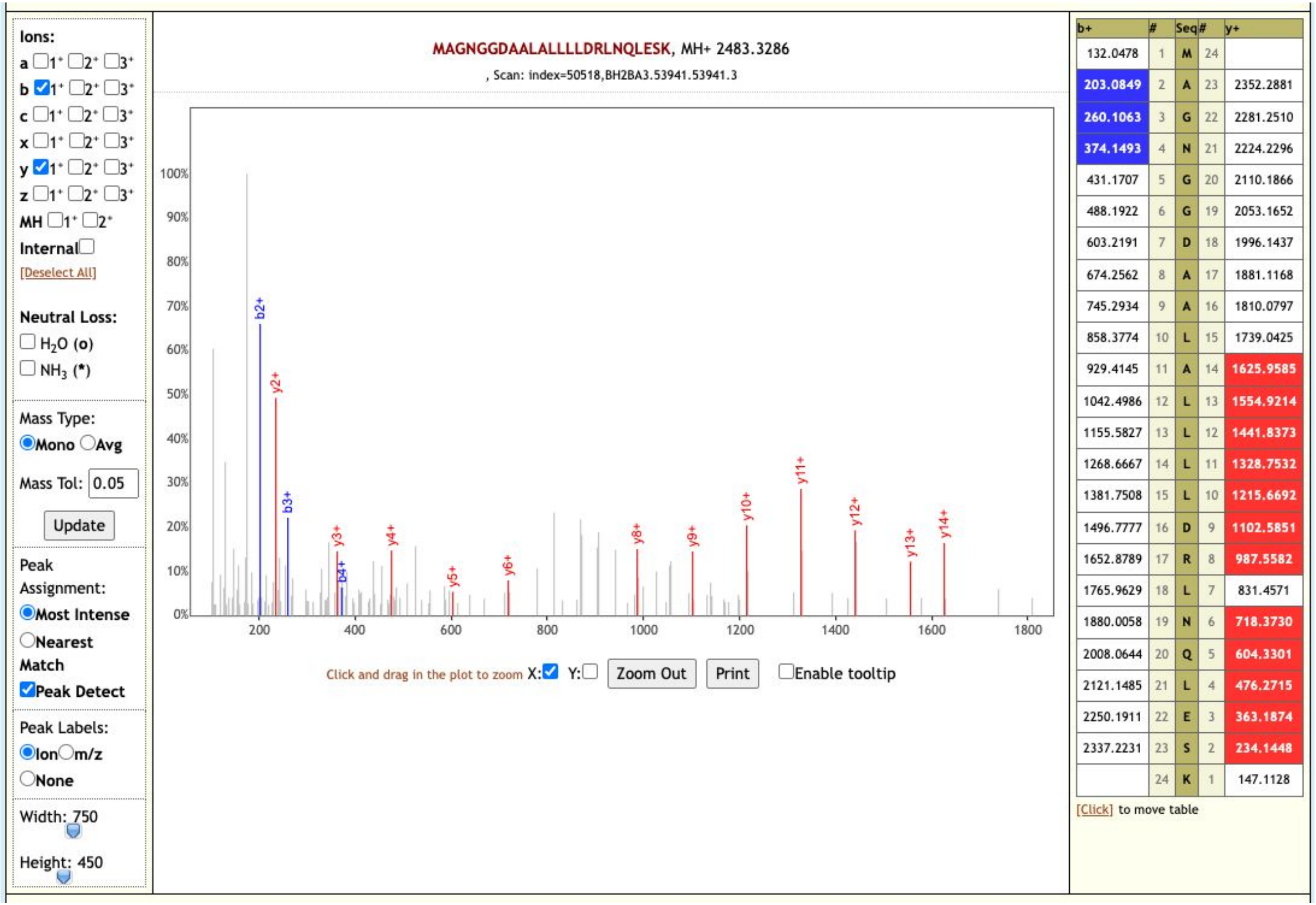

b+	#	Seq	#	y+
132.0478	1	M	24	
203.0849	2	A	23	2352.2881
260.1063	3	G	22	2281.2510
374.1493	4	N	21	2224.2296
431.1707	5	G	20	2110.1866
488.1922	6	G	19	2053.1652
603.2191	7	D	18	1996.1437
674.2562	8	A	17	1881.1168
745.2934	9	A	16	1810.0797
858.3774	10	L	15	1739.0425
929.4145	11	A	14	1625.9585
1042.4986	12	L	13	1554.9214
1155.5827	13	L	12	1441.8373
1268.6667	14	L	11	1328.7532
1381.7508	15	L	10	1215.6692
1496.7777	16	D	9	1102.5851
1652.8789	17	R	8	987.5582
1765.9629	18	L	7	831.4571
1880.0058	19	N	6	718.3730
2008.0644	20	Q	5	604.3301
2121.1485	21	L	4	476.2715
2250.1911	22	E	3	363.1874
2337.2231	23	S	2	234.1448
	24	K	1	147.1128

[Click] to move table

TALTQHGKEDLK

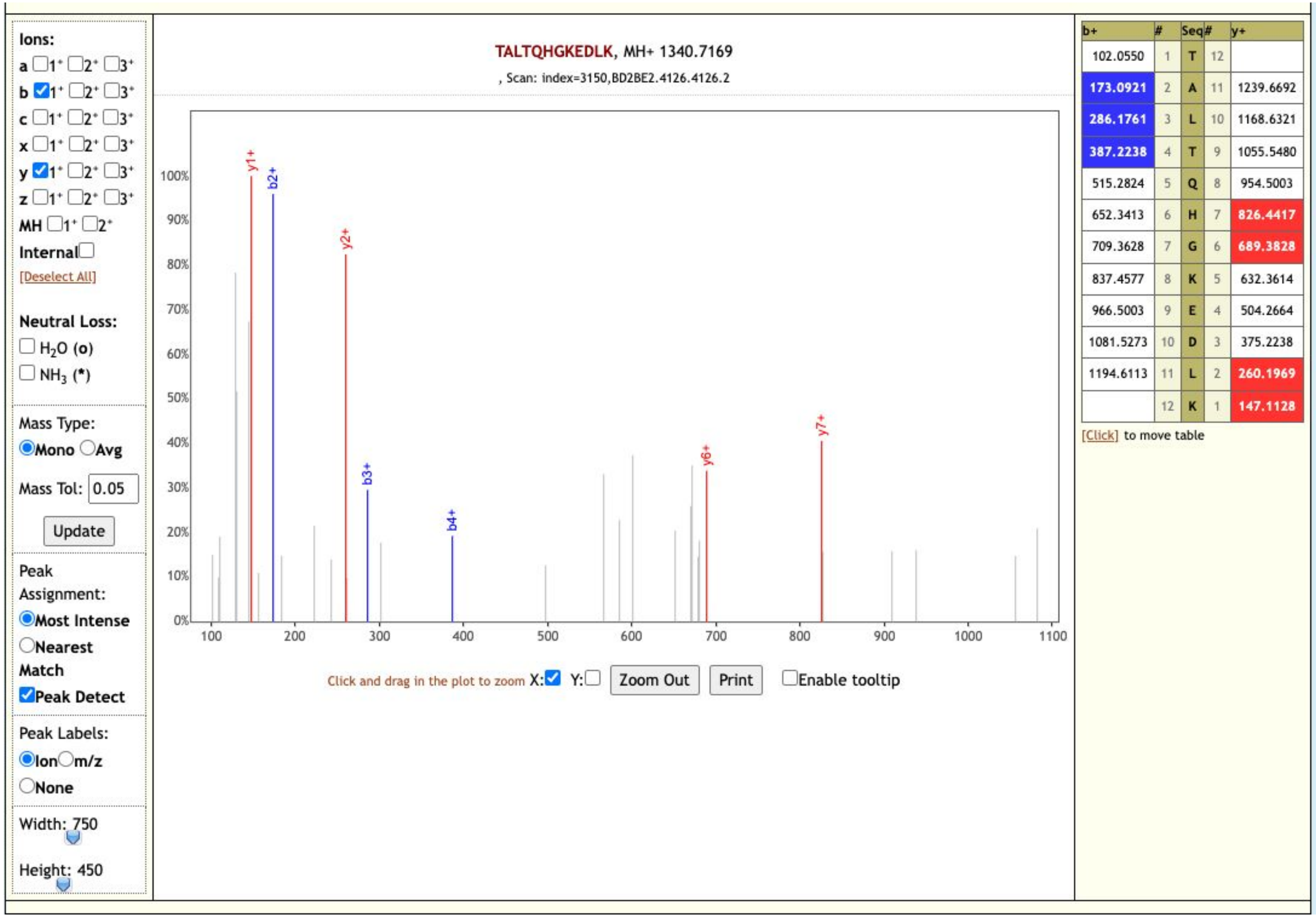

b+	#	Seq	#	y+
102.0550	1	T	12	
173.0921	2	A	11	1239.6692
286.1761	3	L	10	1168.6321
387.2238	4	T	9	1055.5480
515.2824	5	Q	8	954.5003
652.3413	6	H	7	826.4417
709.3628	7	G	6	689.3828
837.4577	8	K	5	632.3614
966.5003	9	E	4	504.2664
1081.5273	10	D	3	375.2238
1194.6113	11	L	2	260.1969
	12	K	1	147.1128

[Click] to move table

KQQTVTLLPAADLDDFSK

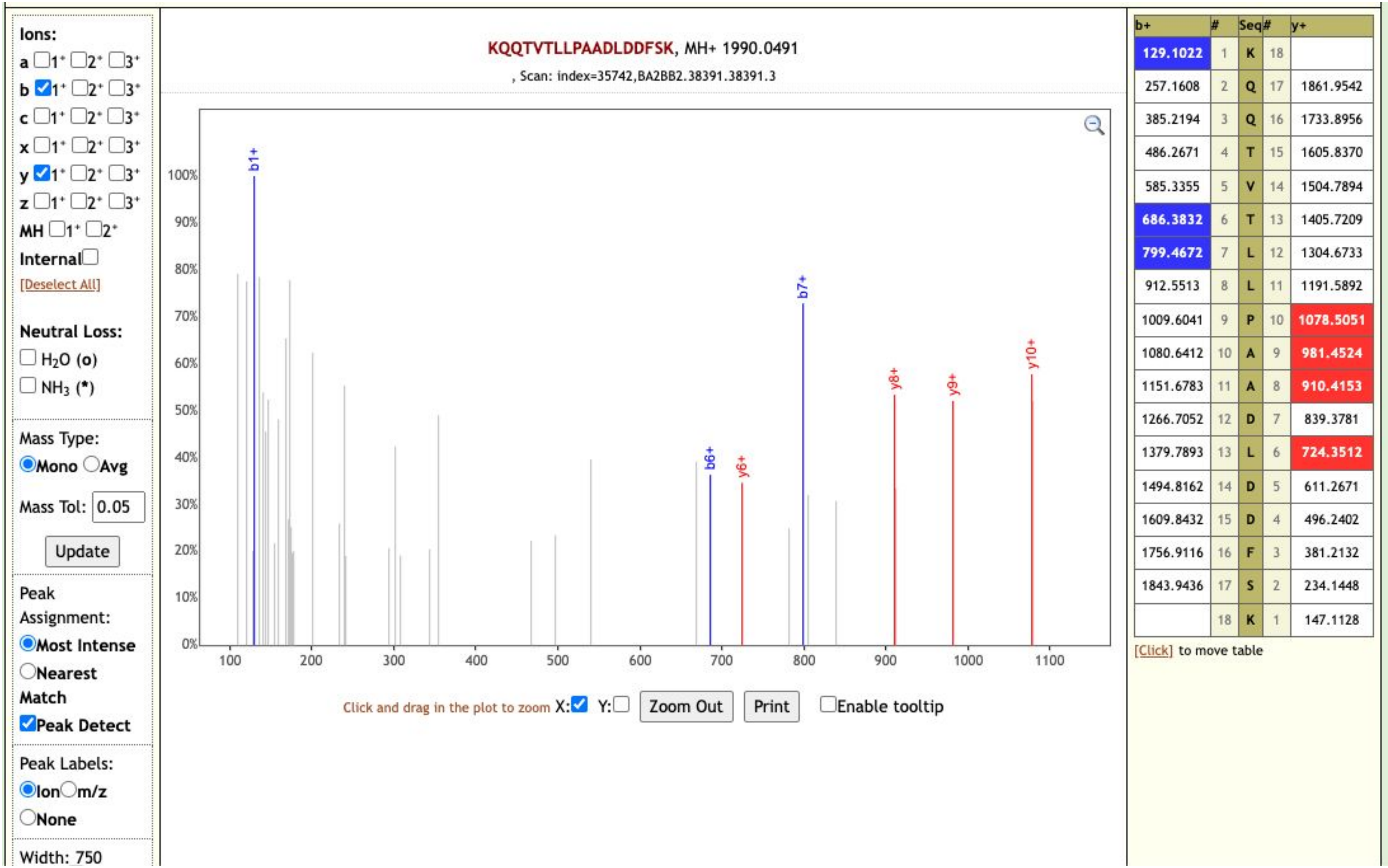

b+	#	Seq	#	y+
129.1022	1	K	18	
257.1608	2	Q	17	1861.9542
385.2194	3	Q	16	1733.8956
486.2671	4	T	15	1605.8370
585.3355	5	V	14	1504.7894
686.3832	6	T	13	1405.7209
799.4672	7	L	12	1304.6733
912.5513	8	L	11	1191.5892
1009.6041	9	P	10	1078.5051
1080.6412	10	A	9	981.4524
1151.6783	11	A	8	910.4153
1266.7052	12	D	7	839.3781
1379.7893	13	L	6	724.3512
1494.8162	14	D	5	611.2671
1609.8432	15	D	4	496.2402
1756.9116	16	F	3	381.2132
1843.9436	17	S	2	234.1448
	18	K	1	147.1128

MAGNGGDAALALLLLDRLNQLESK

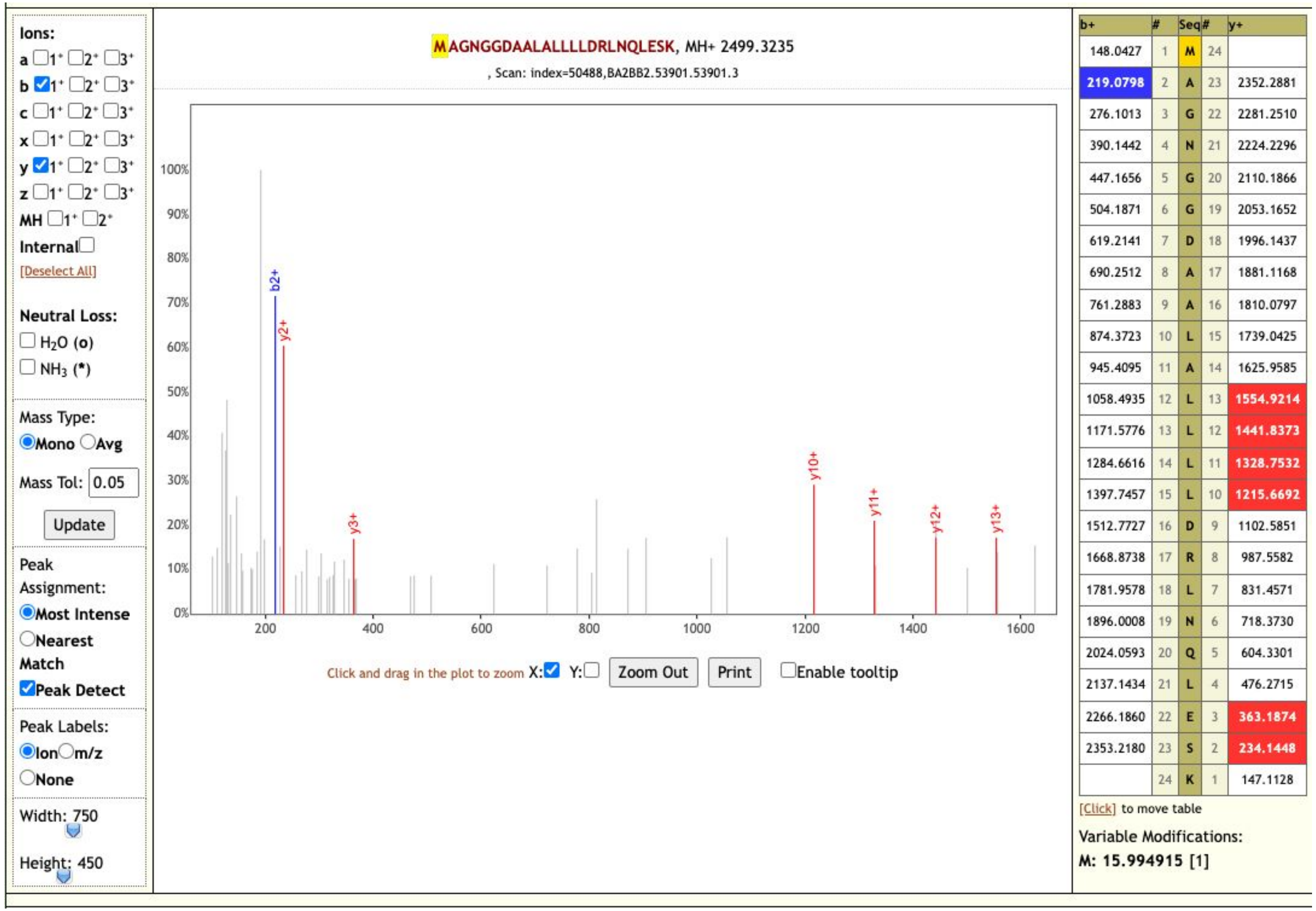

b+	#	Seq	#	y+
148.0427	1	M	24	
219.0798	2	A	23	2352.2881
276.1013	3	G	22	2281.2510
390.1442	4	N	21	2224.2296
447.1656	5	G	20	2110.1866
504.1871	6	G	19	2053.1652
619.2141	7	D	18	1996.1437
690.2512	8	A	17	1881.1168
761.2883	9	A	16	1810.0797
874.3723	10	L	15	1739.0425
945.4095	11	A	14	1625.9585
1058.4935	12	L	13	1554.9214
1171.5776	13	L	12	1441.8373
1284.6616	14	L	11	1328.7532
1397.7457	15	L	10	1215.6692
1512.7727	16	D	9	1102.5851
1668.8738	17	R	8	987.5582
1781.9578	18	L	7	831.4571
1896.0008	19	N	6	718.3730
2024.0593	20	Q	5	604.3301
2137.1434	21	L	4	476.2715
2266.1860	22	E	3	363.1874
2353.2180	23	S	2	234.1448
	24	K	1	147.1128

[Click] to move table

Variable Modifications:

M: 15.994915 [1]

IGMEVTPSGTWLTYTGAIK

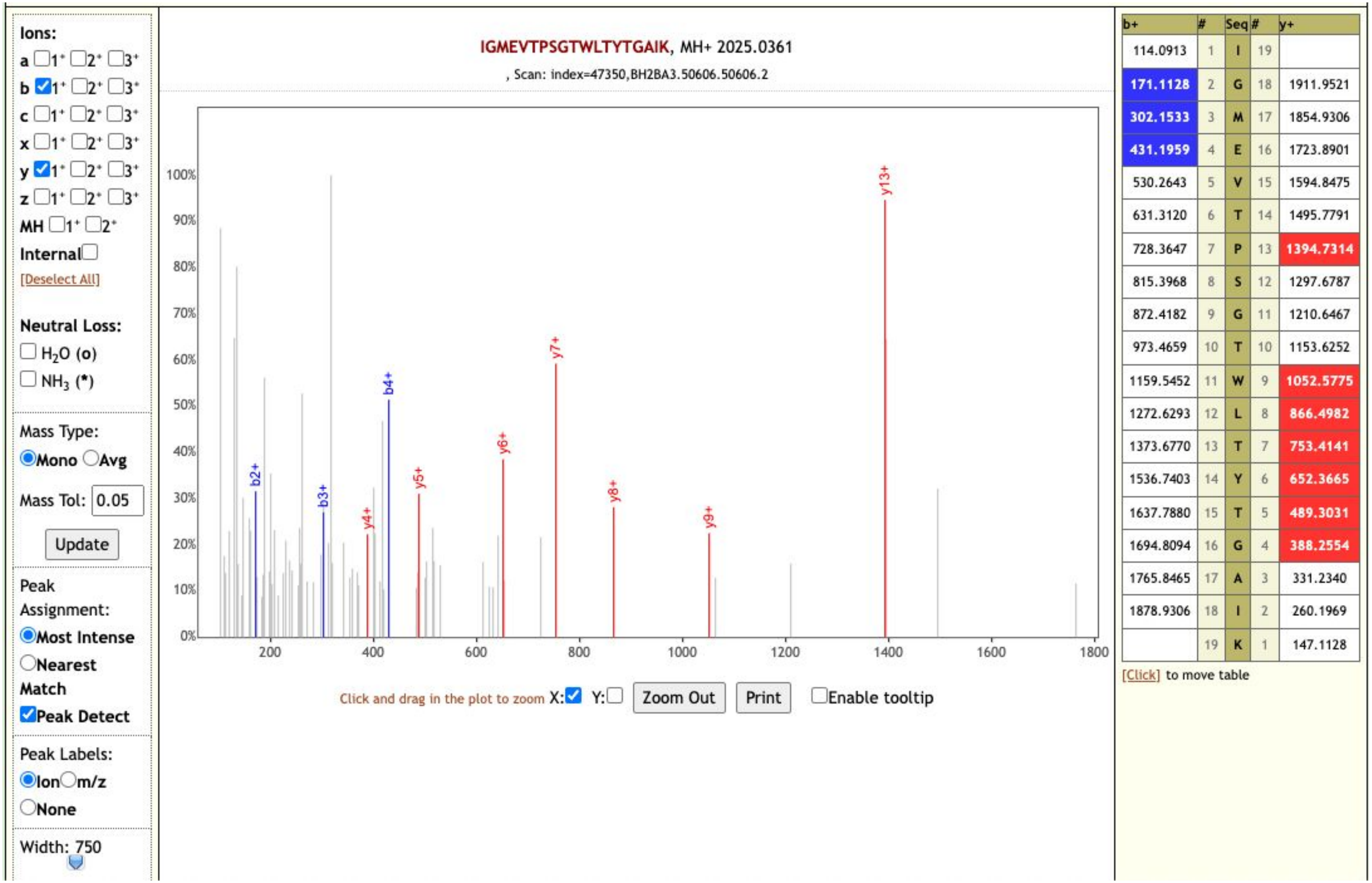

b+	#	Seq	#	y+
114.0913	1	I	19	
171.1128	2	G	18	1911.9521
302.1533	3	M	17	1854.9306
431.1959	4	E	16	1723.8901
530.2643	5	V	15	1594.8475
631.3120	6	T	14	1495.7791
728.3647	7	P	13	1394.7314
815.3968	8	S	12	1297.6787
872.4182	9	G	11	1210.6467
973.4659	10	T	10	1153.6252
1159.5452	11	W	9	1052.5775
1272.6293	12	L	8	866.4982
1373.6770	13	T	7	753.4141
1536.7403	14	Y	6	652.3665
1637.7880	15	T	5	489.3031
1694.8094	16	G	4	388.2554
1765.8465	17	A	3	331.2340
1878.9306	18	I	2	260.1969
	19	K	1	147.1128

[Click] to move table

MAGDGGDAALALLLLDR

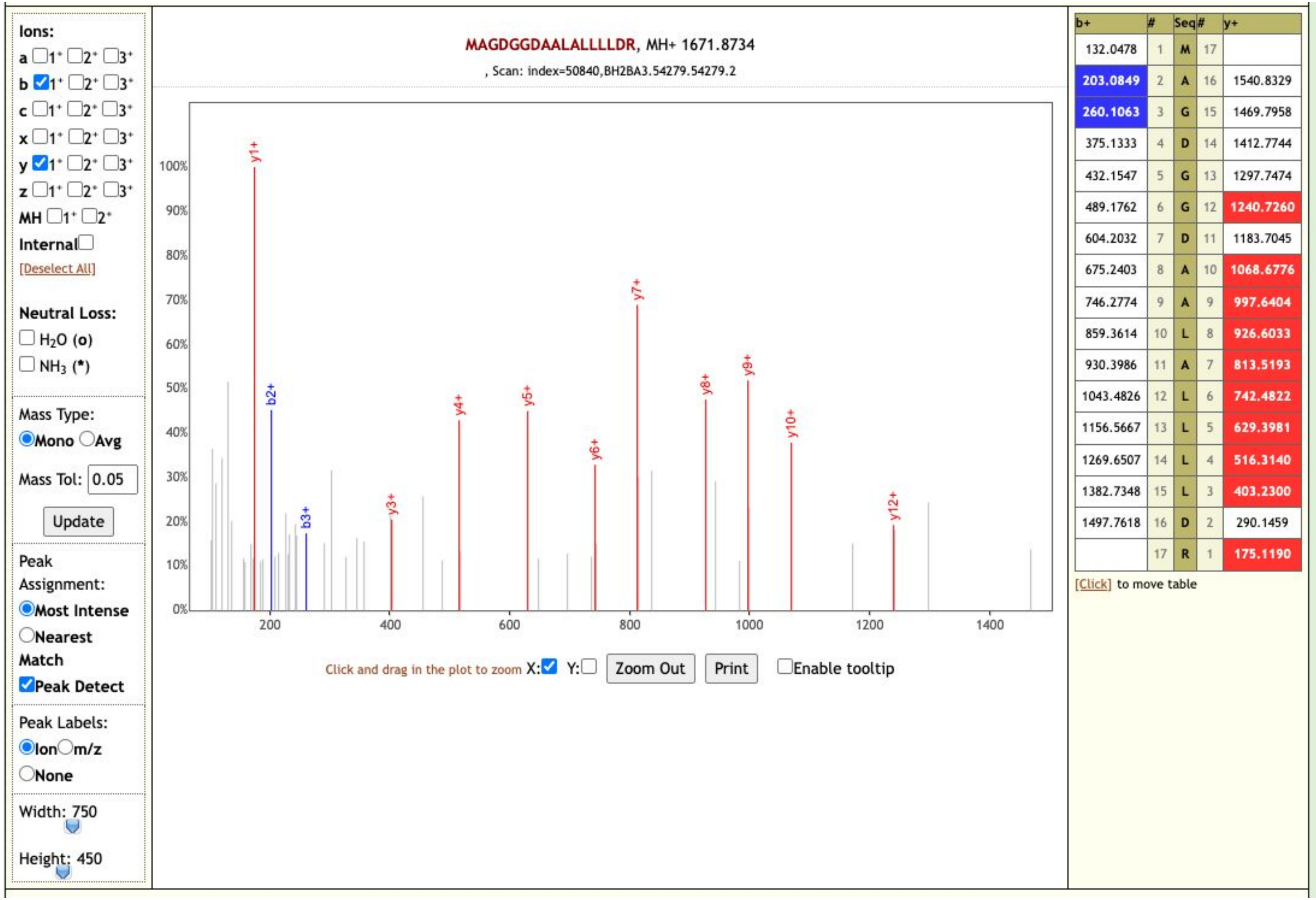

WYFYYLGTGPEAGLPYGANK

Ions:

a ☐1⁺ ☐2⁺ ☐3⁺
b ☑1⁺ ☐2⁺ ☐3⁺
c ☐1⁺ ☐2⁺ ☐3⁺
x ☐1⁺ ☐2⁺ ☐3⁺
y ☑1⁺ ☐2⁺ ☐3⁺
z ☐1⁺ ☐2⁺ ☐3⁺
MH ☐1⁺ ☐2⁺
Internal ☐

[Deselect All]

Neutral Loss:

☐ H_2O (o)
☐ NH_3 (*)

Mass Type: ◉Mono ○Avg

Mass Tol: 0.05

Update

Peak Assignment: ◉Most Intense ○Nearest Match ☑Peak Detect

Peak Labels: ◉Ion ○m/z ○None

WYFYYLGTGPEAGLPYGANK, MH+ 2267.0808

, Scan: index=49118,BH2BA3.52471.52471.2

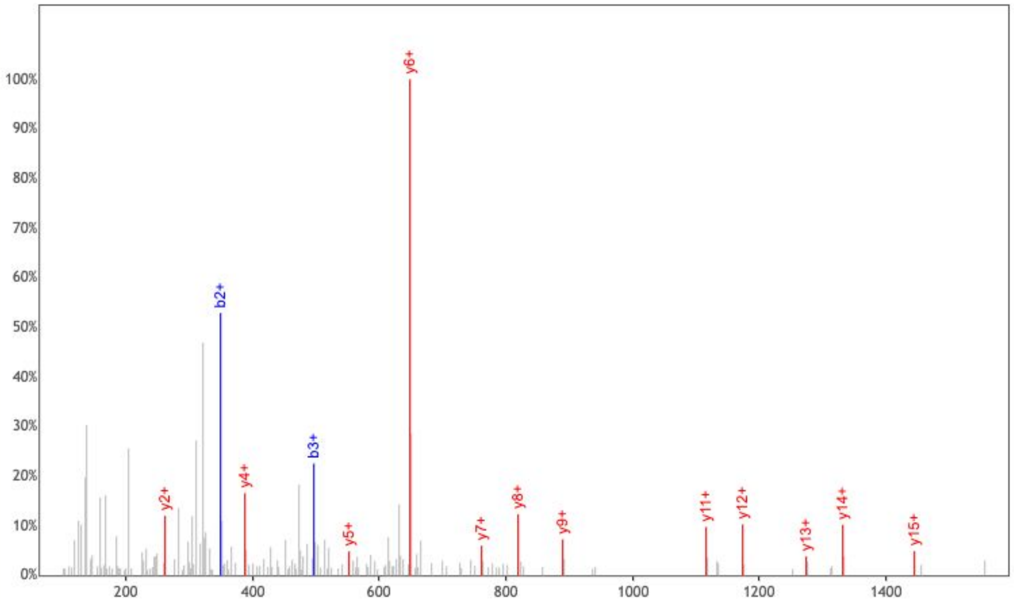

Click and drag in the plot to zoom X:☑ Y:☐ Zoom Out Print ☐Enable tooltip

b+	#	Seq	#	y+
187.0866	1	W	20	
350.1499	2	Y	19	2081.0015
497.2183	3	F	18	1917.9381
660.2817	4	Y	17	1770.8697
823.3450	5	Y	16	1607.8064
936.4291	6	L	15	1444.7431
993.4505	7	G	14	1331.6590
1094.4982	8	T	13	1274.6375
1151.5197	9	G	12	1173.5899
1248.5724	10	P	11	1116.5684
1377.6150	11	E	10	1019.5156
1448.6521	12	A	9	890.4730
1505.6736	13	G	8	819.4359
1618.7577	14	L	7	762.4145
1715.8104	15	P	6	649.3304
1878.8737	16	Y	5	552.2776
1935.8952	17	G	4	389.2143
2006.9323	18	A	3	332.1928
2120.9753	19	N	2	261.1557
	20	K	1	147.1128

[Click] to move table
