# Supplementary material for: Catching the Wave: Detecting Strain-Specific SARS-CoV-2 Peptides in Clinical Samples Collected during Infection Waves from Diverse Geographical Locations
Source: Viruses. 2022 Oct 7;14(10):2205. doi: 10.3390/v14102205 (PMC9609567; doi:10.3390/v14102205)
Supplement: Supplementary file 1 [file viruses-14-02205-s001.zip › Supplementary Data S3.pdf]

PXD019423	PXD019686	PXD020394	PXD021328	PXD022085	PXD023016	PXD024967	PXD025214	PXD026795	Gamma (PXD034582)	Delta (PXD034582)	Omicron (PXD034582)	All Peptides
GQGVPINTNSSPDDQIGYYRR	ADETQALPQR	AYNVTQAFGR	TFPPTEPK		DGIIWVATEGALNTPK		AYNVTQAFGR	ADETQALPQR	ADETQALPQR	WYFYYLGTGPEAGLPYGANK	AYNVTQAFGR	GQGVPINTNSSPDDQIGYYRR
YLGTGPEAGLPYGANK	ASANLAATK	AYNVTQAFGRRGPEQTQGNFGDQELIR	NPANNAAIVLQLPQGTTLPK		DGIIWVATEGALNTPKG		DDQIGYYR	ANNAAIVLQLPQGTTLPK	AYNVTQAFGR	HWPQIAQFAPSASAFF	DGIIWVATEGALNTPK	YLGTGPEAGLPYGANK
AYNVTQAFGR	CDIKDLPK	DGIIWVATEGALNTPK	RPQGLPNNTASWFTALTQHGKEDLK		GIIWVATEGALNTPK		DDQIGYYRR	AYNVTQAFGGR	DDQIGYYR	IGAGICASYQTQTNSPR	DGILWVATEGALNTPK	AYNVTQAFGR
RGPEQTQGNFGDQELIR	CYGVSPTK	HIDAYKTFPPTEPK	GFYAEGSR		IIWVATEGALNTPK		DGILWVATEGALNTPK	AYNVTQAFGR	DGIIWVATEGALNTPK	LDDKDPNFKDQVILLNK	FPQGQGVPINTNSSR	RGPEQTQGNFGDQELIR
AGGNYNYRYR	EIDRLNEVAK	MAGDGGDAALALLLLDR	ITFGGPSDSTGSNQDGER		IWVATEGALNTPK		DGILWVATEGALNTPKDHIGTR	DGILWVATEGALNTPK	DLPIEITVATSRTLSYYK	IGMEVTPSGTWLTYTGAIK	GFYAEGSR	AGGNYNYRYR
CEFQFCNDPFLDVYYHKNNK	EINRLNEVAK	MAGNGGDAALALLLLDR	AYNVTQAFGR		MAGNGCDAALALLLLNR		GFYAEGSR	GFYAEGSR	GEGVPINTNSSPDDQIGYYR	NLNESLIDLQVL	GPEQTQGNFGDQELIR	CEFQFCNDPFLDVYYHKNNK
MADSNGTVEELKK	EITVATSR	QGTDYKHWPQIAQFAPSASAFFGMSR	HWPQIAQFAPSASAFF		VATEGALNTPK		GIYQTSNFR	GPEQTQGNFGDQELIRR	GPEQTQGNFGDQELIR	NSTPGSSMGTSPAR	GQGVPINTNSSPDDQIGYYR	MADSNGTVEELKK
RPQGLPNNTASWFTALTQHGK	ELTVATSR	QKRTATKAYNVTQAFGR	GQGVPINTNSSPDDQIGYYR		WVATEGALNTPK		GPEQTQGNFGDQELLR	GPEQTQGNFGDQELLR	GQGVPINTNSSR	DGIIWVATEGALNTPK	HWPQIAQFAPSASAFF	RPQGLPNNTASWFTALTQHGK
KQQTVTLLPAADLDDFSK	GFYAEGSR	RGPEQTQGNFGDQELIR	RPQGLPNNTASWF		DGILWVATEGALNTPK		GQGVPINTNSSPDDQIGYYR	GQGVPIDTNSSPDDQIGYYR	IAGHHLGR	NPANNAAIVLQLPQGTTLPK	IGMEVTPSGTWLTYTGAIK	KQQTVTLLPAADLDDFSK
ITFVEPSDSTGSNQNGER	HTPINLVR	RTATKAYNVTQAFGR	RGPEQTQGNFGDQELIR				GQGVPINTNSSPDDQIGYYRR	GQGVPINTNSSPDDQIGYYR	ITFGGPSDSTGSNQNGER	GQGVPINTNSSPDDQIGYYR	ITFGGPSDSTGSNQNGER	ITFVEPSDSTGSNQNGER
ITFVGPSDSTDSNQNGER	IAGHHLGR	TATKAYNVTQAFGR	QQTVTLLPAADLDDFSK				GQGVPINTNSSR	IGMEVTPSGTWLTYTGAIK	KADETQALPQR	RPQGLPNNTASWFTALTQHGK	ITFGGPSDSTGSNQNGGAR	ITFVGPSDSTDSNQNGER
IGMEVTPSGTWLTYTGAIK	ICASHQTQTNSPR	AYNVTQAFGRRGPEQTQGNFGDQELLR	RPQGLPDNTASWF				GQGVPINTNSSRDDQIGYYR	ITFGGPSDSTGSNQDGER	LDDKDPNFK	AYETQALPQR	KADETQALPQR	IGMEVTPSGTWLTYTGAIK
MAGNGGDAALALLLLDR	KADETQALPQR	DGILWVATEGALNTPK	MAGNGGDAALALLLLDR				HWPQIAQFAPSASAFF	ITFGGPSDSTGSNQNGER	LDDKDPNFKDQVILLNK	KAYETQALPQR	KKADETQALPQR	MAGNGGDAALALLLLDR
NKCINFNFNGLTGTGVLTESNKK	KANETQALPQR	RGPEQTQGNFGDQELLR	DGIIWVATEGALNTPK				IGMEVTPSGTWLTYTGAIK	KADETQALPQR	LLPAADLDDFFK	QQTVTLLPAADLDDFSK	KQQTVTLLPAADLDDFSK	NKCINFNFNGLTGTGVLTESNKK
	KKADETQALPQR		IAGHHLGR				ITFGGPSDSTGSNENGER	KQLTVTLLPAADLDDFSK	LQDVVNQNAQALNTLVK	RPQGLPNNTASWF	LDDKDPNFKDQVILLNK	ADETQALPQR
	LDDKDPNFK		QLPQGTTLPK				ITFGGPSDSTGSNQDGER	LDDKDPNFK	MAGDAALALL	RGPEQTQGNFGDQELIR	LGSPLSLNMAR	ASANLAATK
	MSECVLGQSK		LDDKDPNFK				ITFGGPSDSTGSNQNGER	LNTDHSSSSDNIALLVQ	NPANNAAIVIQLPQGTTLPK	TFPPTEPK	MAGDGGDAALALLLLDR	CDIKDLPK
	NNISWMESE		SWMESEFR				KADETQALPQR	LQEVVNQNAQALNTLVK	NPANNAAIVL	IAGHHLGR	MAGNGGDAAIALLLLDRLNQLESK	CYGVSPTK
	QLQQSMSSADSTQA		LDDKDPNFKDQVILLNK				KQQTVTLLPAADLDDFSK	LVDPQIQLAVTR	NPANNAAIVLQIPQGTTLPK	AYNVTQAFGR	MAGNGGDAALALLLLDR	EIDRLNEVAK
	SNLKPFER		LTYTGAIK				LDDKDPNFKDQVLLLNK	MAGDGGDAALALLLLDR	NSIPGSSMGTSPARMA	GFYAEGSR	MAGNGGDAALALLLLDRLNQLESK	EINRLNEVAK
	TFGAGAAL		GPEQTQGNFGDQELIR				LNTDHSSSSDNIALLVQ	MAGNGNDAALALLLLDR	NSTPGSSKPTSPARMAGNGGDAA	VAGDSGFAAYSR	NPANNAAIVIQLPQGTTLPK	EITVATSR
	TFPPTEPK		VATEGALNTPK				LQEVVNQNAQALNTLVK	NPANNAALVLQLPQGTTLPK	RGPEQTQGNFGDQELIR	KSNLKPFER	NPANNAAIVLQIPQGTTLPK	ELTVATSR
	TNQFNSAIGK		ADETQALPQR				MAGDGGDAALALLLLDR	NSSRTSTPGSSK	RPQGLPNNTASWF	YLGTGPEAGLPYGANK	NPANNAAIVLQLPQGTTLPK	GFYAEGSR
	ADETQALPQR		GWIFGTTLDSK				MAGNGGDAALALLLLDR	QLQQSMSSADSTQA	RPQGLPNNTASWFTALTQHGK	ITFGGPSDSTGSNQNGER	QLPQGTTLPK	HTPINLVR
	ASANLAATK		ITFGGPSDSTGSNQNGER				MAGNGGDAALALLLLDRLNQLESK	QLTVTLLPAADLDDFSK	RPQGLPNNTASWFTALTQHGKEDLK	HTPINLVR	QQTVTLLPAADLDDFSK	IAGHHLGR
	CDIKDLPK		WYFYYLGTGPEAGLPY				NPANNAAIVLQLPEGTTLPK	RGPEQTQGNFGDQELLR	SDNGPQNLPNAPR	DGILWVATEGALNTPK	RATRRIPGGDGK	ICASHQTQTNSPR
	CDIKNLPK		YLGTGPEAGLPYGANK				NPANNAALVLQLPQGTTLPK	RPQGLPNNTASWFTALTQHGK	SILSPLYAFASEAAR	LDDKDPNFK	RGPEQTQGNFGDQELIR	KADETQALPQR
	CYGVSPTK		KKADETQALPQR				NSSRNSTPGSSIRTSPGMAGNGGDAALALLLLDR	SDNGPQNQRDAPR	SLVPGFNEK	GQGVPINTNSSPDDQIGYYRR	RPQGLPNNTASWF	KANETQALPQR
	EIDRLNQVAK		RPQGLPNNTASW				QQTVTLLPAADLDDFSK	VAGDSGFAAYSR	TALTQHGKEDLK	LNQLGSKMSG	RPQGLPNNTASWFTALTQHGK	KKADETQALPQR
	ELTVATSR		LTQHGKEDLK				RGPEQTQGNFGDEELIR	VGGNYNYLYR	YLGTGPEAGLPYGANK	GPEQTQGNFGDQELIR	TALTQHGKEDLK	LDDKDPNFK
	GFYAEGSR		GQGVPINTNSSPDDQIGYY				RGPEQTQGNFGDQELLR	VYSTGSNVFQTR	DGILWVATEGALNTPK	FPQGQGVPINTNSSR	TALTQHGKEDLKFPR	MSECVLGQSK
	GVYYPDK		KQQTVTLLPAADLDDFSK				RGPEQTQGNFGDQELTR	WYFYYLGTGPEAGLPYGANK	GPEQTQGNFGDQELLR	DQVILLNK	TFPPTEPK	NNISWMESE
	HTPINLVR		RPQGLPNNTASWFTALTQHGK				RPQGLPNNTASWFTALTQHGK		GQGVPINTNSSPDDQIGYYR	HWPQIAQFAPSASAFFGMSR	VATEGALNTPK	QLQQSMSSADSTQA
	IAGHHLGR		LGTGPEAGLPYGANK				RPQGLPNNTASWFTALTQHGKEDLK		HWPQIAQFAPSASAFF	GWIFGTTLDSK	VTSPARMAGN	SNLKPFER
	LDDKDPNFK		MAGNGGDAALALLLLDRLNQLESK				VGGNYNYLYR		IGMEVTPSGTWLTYTGAIK	NGIIWVATEGALNTPK	WYFYYLGTGPEAGLPYGANK	TFGAGAAL
	LDDKNPNFK		PAADLDDFSK				VTLADAGFIK		LNTDHSSSSDNIALLVQ	ITFGGPSDSTGSNQDGER	YLGTGPEAGLPYGANK	TFPPTEPK
	QLQQSMSSSDSTQA		HWPQIAQFAPSASAF				WYFYYLGTGPEAGLPYGANK		LQEVVNQNAQALNTLVK	VYSTGSNVFQTR	ADETQALPQR	TNQFNSAIGK
	RVDFCGK		QLQQSMSSADSTQA				WYFYYLGTGPESGLPYGANK		NPANNAALVLQLPQGTTLPK	LNQLESKMAGK	GPEQTQGNFGDQELLR	CDIKNLPK
	SNLKPFER		IGMEVTPSGTWLTYTGAIK				PGNGCDAALALLLLDR		RGPEQTQGNFGDQELLR	VTLADAGFIK	ITFGGPSDSTGSNQNGQR	EIDRLNQVAK
			IGMEVTPSGTWLTY				NPANNAAIVLQLPQGTTLPK		SLNVAKSEFDRDAAMQR	FLPFQQFGR	LDDKDPNFKDQVLLLNK	GVYYPDK
			GFYAEGSRGGSEASSR				TFPPTEPK		WYFYYLGTGPEAGLPYGANK	LVDPQIQLAVTR	LNKVEAEVQIDR	LDDKNPNFK
			KADETQALPQR				YLGTGPESGLPYGANK			GWLFGTTLDSK	LQDVVNQNAQALNTLVK	QLQQSMSSSDSTQA
			RPQGLPNNTASWFTAL				PNFKDQVILLNK			LGSPLSLNMAR	LQEVVNQNAQALNTLVK	RVDFCGK
			KSNLKPFER				RGPEQTQGNFGDQELIR			AFQLTPIAVQMTK	NPANNAALVLQLPQGTTLPK	DGIIWVATEGALNTPK
			VGGNYNYLYR				LDDKDPNFKDQVILLNK				RGPEQTQGNFGDQELLR	DGIIWVATEGALNTPKG
			LNTDHSSSSDNIALLVQ				VAGDSGFAAYSR					GIIWVATEGALNTPK
			WYFYYLGTGPEAGLPYGANK				HTPINLVR					IIWVATEGALNTPK
			RGPEQTQGNFGDQELTR				TALTQHGKEDLKFPR					IWVATEGALNTPK
			NPANNAAIVLQLPQGT				RPQGLPNNTASWF					MAGNGCDAALALLLLNR
			GGDAALALLLLDR				KKADETQALPQR					VATEGALNTPK
			TQLPPAYTNSFTR				GPEQTQGNFGDQELIR					WVATEGALNTPK
			VTLADAGFIK				RPQGLPNNTASW					DGILWVATEGALNTPK
			NTNSSPDDQIGYYR				FTALTQHGK					NPANNAAIVLQLPQGTTLPK
			FDNPVLPFNDGVYFASTEK				DGIIWVATEGALNTPK					RPQGLPNNTASWFTALTQHGKEDLK
			FLPFQQFSR				TLLPAADLDDFSK					ITFGGPSDSTGSNQDGER
			GWIFGTTLDPK				YYLGTGPEAGLPYGANK					HWPQIAQFAPSASAFF
			AGDGGDAALALLLLDR				LGTGPEAGLPYGANK					GQGVPINTNSSPDDQIGYYR
			AYIVTQAFGR				NPANNAAIVL					RPQGLPNNTASWF
			FNGIGVTQNVLYENQK				DQVILLNK					QQTVTLLPAADLDDFSK
			LIANQFNSAIGK				YLGTGPEAGLPYGANK					RPQGLPDNTASWF
			SSPDDQIGYYR				QQTVTLLPAADLDYFSK					QLPQGTTLPK
			HWPQIAQF				VATEGALNTPK					SWMESEFR
			MAGDGGDAALALLLLDRLNQLESK				RGPEQTQGNFGDQEL					LDDKDPNFKDQVILLNK
			TLLPAADLDDFSK				NPANDAAIVLQLPQGTTLPK					LTYTGAIK
			YYLGTGPEAGLPYGANK				GTGPEAGLPYGANK					GPEQTQGNFGDQELIR
			NSSPDDQIGYYR				RPQGLPNNTASWFTALTQHGKEDLKFPR					GWIFGTTLDSK
			SMGTSPTRMAGNGGDAALALLLLDR				DRLNQLESK					ITFGGPSDSTGSNQNGER
			HWPQIAQFAPSASAFFGMSR				TQHGKEDLKFPR					WYFYYLGTGPEAGLPY
			FPQGQGVPINTNSSR				LDDKDPNFK					RPQGLPNNTASW
			LLDRLNQLESK				HWPQIAQFAPSASAF					LTQHGKEDLK
			RPQGLPNNTASWFTALTQHGKEDLKFPR									GQGVPINTNSSPDDQIGYY
			RFDNPVLPFNDGVYFASTEK									LGTGPEAGLPYGANK
			VQPTESIVR									MAGNGGDAALALLLLDRLNQLESK
			FTALTQHGKEDLK									PAADLDDFSK
			GQGVPINTNSSPDDQIGY									HWPQIAQFAPSASAF
			GPEQTQGNFGDQELTR									IGMEVTPSGTWLTY
			QGTDYKHWPQIAQFAPSASAFF									GFYAEGSRGGSEASSR
			ALALLLLDR									RPQGLPNNTASWFTAL
			VAGDSGFAAYSR									KSNLKPFER
			LQDVVNQNAQALNTLVK									VGGNYNYLYR
			ITFGGPSDSTGSNQDGERSGAR									LNTDHSSSSDNIALLVQ
			RPQGLPNNTASWFT									WYFYYLGTGPEAGLPYGANK
			MAGNGGDAAIALLLLDRLNQLESK									RGPEQTQGNFGDQELTR
			DDKDPNFKDQVILLNK									NPANNAAIVLQLPQGT
			LQSLQTYVTQQLIR									GGDAALALLLLDR
			ALTGISVEQDKNTQEVFAQVK									TQLPPAYTNSFTR
			FLPFQQFGR									VTLADAGFIK
			EITVATSR									NTNSSPDDQIGYYR

			HWPQIAQFAPSASAFFGMSRICMEVTPSGT									FDNPVLPFNDGVYFASTEK
			SNLKPFER									FLPFQQFSR
			SFIEDLLFNK									GWIFGTTLDPK
			TALTQHGKEDLK									AGDGGDAALALLLLDR
			AGNGGDAALALLLLDRLNQLESK									AYIVTQAFGR
			YWPQIAQFAPSASAFF									FNGIGVTQNVLYENQK
			VYSTGSNVFQTR									LIANQFNSAIGK
			DQVILLNK									SSPDDQIGYYR
			TLVATAEAELAK									HWPQIAQF
			GFQPTNGVGYQPYR									MAGDGGDAALALLLLDRLNQLESK
			FTALTQHGK									TLLPAADLDDFSK
			AGNGGDAALALLLLDR									YYLGTGPEAGLPYGANK
			ALTGIAVEQDKNTQEVFAQVK									NSSPDDQIGYYR
			DGILWVATEGALNTPK									SMGTSPTRMAGNGGDAALALLLLDR
			ELTVATSR									HWPQIAQFAPSASAFFGMSR
			FDNPVLPFNDGVYFSATEK									FPQGQGVPINTNSSR
			GPEQTQGNFGDQELLR									LLDRLNQLESK
			GWLFGTTLDSK									RPQGLPNNTASWFTALTQHGKEDLKFPR
			HTPINLVR									RFDNPVLPFNDGVYFASTEK
			ITFGGPSDSTGSNENGER									VQPTESIVR
			LDDKDPNFKDQVLLLNK									FTALTQHGKEDLK
			LQDSLSSTASALGK									GQGVPINTNSSPDDQIGY
			MAGDGGDAALALLLLDR									GPEQTQGNFGDQELTR
			NPANNAALVLQLPQGTTLPK									QGTDYKHWPQIAQFAPSASAFF
			RGPEQTQGNFGDEELIR									ALALLLLDR
			RGPEQTQGNFGDQELLR									VAGDSGFAAYSR
			SDNGPQNQRDAPR									LQDVVNQNAQALNTLVK
			SFLEDLLFNK									ITFGGPSDSTGSNQDGERSGAR
												RPQGLPNNTASWFT
												MAGNGGDAAIALLLLDRLNQLESK
												DDKDPNFKDQVILLNK
												LQSLQTYVTQQLIR
												ALTGISVEQDKNTQEVFAQVK
												FLPFQQFGR
												HWPQIAQFAPSASAFFGMSRICMEVTPSGT
												SFIEDLLFNK
												TALTQHGKEDLK
												AGNGGDAALALLLLDRLNQLESK
												YWPQIAQFAPSASAFF
												VYSTGSNVFQTR
												DQVILLNK
												TLVATAEAELAK
												GFQPTNGVGYQPYR
												FTALTQHGK
												AGNGGDAALALLLLDR
												ALTGIAVEQDKNTQEVFAQVK
												FDNPVLPFNDGVYFSATEK
												GPEQTQGNFGDQELLR
												GWLFGTTLDSK
												ITFGGPSDSTGSNENGER
												LDDKDPNFKDQVLLLNK
												LQDSLSSTASALGK
												MAGDGGDAALALLLLDR
												NPANNAALVLQLPQGTTLPK
												RGPEQTQGNFGDEELIR
												RGPEQTQGNFGDQELLR
												SDNGPQNQRDAPR
												SFLEDLLFNK
												DDQIGYYR
												DDQIGYYRR
												DGILWVATEGALNTPKDHIGTR
												GIYQTSNFR
												GQGVPINTNSSR
												GQGVPINTNSSRDDQIGYYR
												LQEVVNQNAQALNTLVK
												NPANNAAIVLQLPEGTTLPK
												NSSRNSTPGSSIRTSPGMAGNGGDAALALLLLDR
												WYFYYLGTGPESGLPYGANK
												PGNGCDAALALLLLDR
												YLGTGPESGLPYGANK
												PNFKDQVILLNK
												TALTQHGKEDLKFPR
												NPANNAAIVL
												QQTVTLLPAADLDYFSK
												RGPEQTQGNFGDQEL
												NPANDAAIVLQLPQGTTLPK
												GTGPEAGLPYGANK
												DRLNQLESK
												TQHGKEDLKFPR
												ANNAAIVLQLPQGTTLPK
												AYNVTQAFGGR
												GPEQTQGNFGDQELIRR
												GQGVPIDTNSSPDDQIGYYR
												KQLTVTLLPAADLDDFSK
												LVDPQIQLAVTR
												MAGNGNDAALALLLLDR
												NSSRTSTPGSSK
												QLTVTLLPAADLDDFSK
												DLPIEITVATSRTLSYYK

												GEGVPINTNSSPDDQIGYYR
												LLPAADLDDFFK
												MAGDAALALL
												NPANNAAIVIQLPQGTTLPK
												NPANNAAIVLQIPQGTTLPK
												NSIPGSSMGTSPARMA
												NSTPGSSKPTSPARMAGNGGDAA
												SDNGPQNLPNAPR
												SILSPLYAFASEAAR
												SLVPGFNEK
												SLNVAKSEFDRDAAMQR
												IGAGICASYQTQTNSPR
												NLNESLIDLQVL
												NSTPGSSMGTSPAR
												AYETQALPQR
												KAYETQALPQR
												LNQLGSKMSG
												NGIIWVATEGALNTPK
												LNQLESKMAGK
												LGSPLSLNMAR
												AFQLTPIAVQMTK
												ITFGGPSDSTGSNQNGGAR
												RATRRIPGGDGK
												VTSPARMAGN
												ITFGGPSDSTGSNQNGQR
												LNKVEAEVQIDR
