# Supplementary material for: Catching the Wave: Detecting Strain-Specific SARS-CoV-2 Peptides in Clinical Samples Collected during Infection Waves from Diverse Geographical Locations
Source: Viruses. 2022 Oct 7;14(10):2205. doi: 10.3390/v14102205 (PMC9609567; doi:10.3390/v14102205)
Supplement: Supplementary file 1 [file viruses-14-02205-s001.zip › Supplementary Data S4.pdf]

Peptides after SGPS and MaxQuant(203)	803 Peptides	Total Unique peptides (906)	82 Confident Peptides
GQGVPINTNSSPDDQIGYYRR	AAITILDGISQYSLR	AAITILDGISQYSLR	ADETQALPQR
YLGTGPEAGLPYGANK	AAIVLQLPQGTTLPK	AAIVLQLPQGTTLPK	ASANLAATK
AYNVTQAFGR	AALALLLLDR	AALALLLLDR	AYETQALPQR
RGPEQTQGNFGDQELIR	AALALLLLDRLNQLESK	AALALLLLDRLNQLESK	AYNVTQAFGR
AGGNYNYRYR	AALLADKFPVLHDIGNPK	AALLADKFPVLHDIGNPK	CDIKDLPK
CEFQFCNDPFLDVYYHKNNK	ACPLIAAVITR	ACPLIAAVITR	DDKDPNFKDQVILLNK
MADSNGTVEELKK	ACVEEVTTTLEETK	ACVEEVTTTLEETK	DDQIGYYR
RPQGLPNNTASWFTALTQHGK	ADETQALPQR	ADETQALPQR	DGIIWVATEGALNTPK
KQQTVTLLPAADLDDFSK	ADETQALPQRQ	ADETQALPQRQ	DGIIWVATEGALNTPKG
ITFVEPSDSTGSNQNGER	ADETQALPQRQK	ADETQALPQRQK	DRLNQLESK
ITFVGPSDSTDSNQNGER	ADETQALPQRQKK	ADETQALPQRQKK	EITVATSR
IGMEVTPSGTWLTYTGAIK	ADSNGTITVEELK	ADSNGTITVEELK	FDNPVLPFNDGVYFASTEK
MAGNGGDAALALLLLDR	ADSNGTITVEELKK	ADSNGTITVEELKK	FNGIGVTQNVLYENQK
NKCINFNFNGLTGTGVLTESNKK	AENVTGLFK	AENVTGLFK	GEGVPINTNSSPDDQIGYYR
ADETQALPQR	AFDIYNDK	AFDIYNDK	GFQPTNGVGYQPYR
ASANLAATK	AFDIYNDKVAGFAK	AFDIYNDKVAGFAK	GFYAEGSR
CDIKDLPK	AFQLTPIAVQMTK	AFQLTPIAVQMTK	GGDAALALLLLDR
CYGVSPTK	AFQLTPIAVQMTKLATTEELPDEFVVVTVK	AFQLTPIAVQMTKLATTEELPDEFVVVTVK	GIIWVATEGALNTPK
EIDRLNEVAK	AGEAANFCALILAYCNK	AGEAANFCALILAYCNK	GPEQTQGNFGDQELIR
EINRLNEVAK	AGGTTEMLAK	AGGTTEMLAK	GPEQTQGNFGDQELTR
EITVATSR	AGNATEVPANSTVLSFCAFAVDAAK	AGNATEVPANSTVLSFCAFAVDAAK	GQGVPINTNSSPDDQIGY
ELTVATSR	AGNGGDAALALLLLDR	AGNGGDAALALLLLDR	GQGVPINTNSSPDDQIGYYR
GFYAEGSR	AGNGGDAALALLLLDRLNQLESK	AGNGGDAALALLLLDRLNQLESK	GQGVPINTNSSR
HTPINLVR	AISSVLNDILSR	AISSVLNDILSR	HWPQIAQFAPSASAFF
IAGHHLGR	AIVSTIQR	AIVSTIQR	HWPQIAQFAPSASAFFGMSR
ICASHQTQTNSPR	ALNDFSNSGSDVLYQPPQTSITSAVLQ	ALNDFSNSGSDVLYQPPQTSITSAVLQ	IAGHHLGR
KADETQALPQR	ALNDFSNSGSDVLYQPPQTSITSAVLQSGFR	ALNDFSNSGSDVLYQPPQTSITSAVLQSGFR	IGMEVTPSGTWLTYTGAIK
KANETQALPQR	ALNLGETFVTHSK	ALNLGETFVTHSK	IIWVATEGALNTPK
KKADETQALPQR	ALTAESHVDTDLTKPYIK	ALTAESHVDTDLTKPYIK	ITFGGPSDSTGSNQDGER
LDDKDPNFK	ALTGIAVEQDK	ALTGIAVEQDK	ITFGGPSDSTGSNQNGER
MSECVLGQSK	ALTGIAVEQDKNTQEVFAQVK	ALTGIAVEQDKNTQEVFAQVK	ITFGGPSDSTGSNQNGGAR
NNISWMESE	ALTQHGKEDLK	ALTQHGKEDLK	KADETQALPQR
QLQQSMSSADSTQA	ANNAAIVLQLPQGTTLPK	ANNAAIVLQLPQGTTLPK	KAYETQALPQR
SNLKPFER	APSASAFFGMSR	APSASAFFGMSR	KKADETQALPQR
TFGAGAAL	ASANIGCNHTGVVGEGSEGLNDNLLEILQK	ASANIGCNHTGVVGEGSEGLNDNLLEILQK	KQQTVTLLPAADLDDFSK
TFPPTEPK	ASANLAATK	ASANLAATK	KSNLKPFER

TNQFNSAIGK	ASCTLSEQLDFIDTK	ASCTLSEQLDFIDTK	LDDKDPNFK
CDIKNLPK	ASCTLSEQLDFIDTKR	ASCTLSEQLDFIDTKR	LDDKDPNFKDQVILLNK
EIDRLNQVAK	ASMPTTIAK	ASMPTTIAK	LGSPLSLNMAR
GVYYPDK	ASWFTALTQHGK	ASWFTALTQHGK	LGTGPEAGLPYGANK
LDDKNPNFK	ASWFTALTQHGKEDLK	ASWFTALTQHGKEDLK	LIANQFNSAIGK
QLQQSMSSSDSTQA	ASYQTQTNSPR	ASYQTQTNSPR	LQDVVNQNAQALNTLVK
RVDFCGK	ATCEFCGTENLTK	ATCEFCGTENLTK	LQSLQTYVTQQLIR
DGIIWVATEGALNTPK	ATNNAMQVESDDYIATNGPLK	ATNNAMQVESDDYIATNGPLK	LTYTGAIK
DGIIWVATEGALNTPKG	AVDCALDPLSETK	AVDCALDPLSETK	LVDPQIQLAVTR
GIIWVATEGALNTPK	AVFISPYNSQNAVASK	AVFISPYNSQNAVASK	MAGDGGDAALALLLLDR
IIWVATEGALNTPK	AVGACVLCNSQTSLR	AVGACVLCNSQTSLR	MAGNGGDAALALLLLDR
IWVATEGALNTPK	AYKDYLASGGQPITNCVK	AYKDYLASGGQPITNCVK	MAGNGGDAALALLLLDRLNQLESK
MAGNGCDAALALLLLNR	AYNVTQAFGR	AYNVTQAFGR	MSECVLGQSK
VATEGALNTPK	AYNVTQAFGRR	AYNVTQAFGRR	NPANNAAIVIQLPQGTTLPK
WVATEGALNTPK	CAGSTFISDEVAR	CAGSTFISDEVAR	NPANNAAIVLQLPQGT
DGILWVATEGALNTPK	CDHCGETSWQTGDFVK	CDHCGETSWQTGDFVK	NSSPDDQIGYYR
NPANNAAIVLQLPQGTTLPK	CDIKDLPK	CDIKDLPK	NSTPGSSMGTSPAR
RPQGLPNNTASWFTALTQHGKEDLK	CDIKDLPKEITVATSR	CDIKDLPKEITVATSR	NTNSSPDDQIGYYR
ITFGGPSDSTGSNQDGER	CDLQNYGDSATLPK	CDLQNYGDSATLPK	PAADLDDFSK
HWPQIAQFAPSASAFF	CLWSTKPVETSNSFDVLK	CLWSTKPVETSNSFDVLK	PGNGCDAALALLLLDR
GQGVPINTNSSPDDQIGYYR	CPAEIVDTVSALVYDNK	CPAEIVDTVSALVYDNK	QGTDYKHWPQIAQFAPSASAFFGMSR
RPQGLPNNTASWF	CSFYEDFLEYHDVR	CSFYEDFLEYHDVR	QKRTATKAYNVTQAFGR
QQTVTLLPAADLDDFSK	CTSVVLLSVLQQLR	CTSVVLLSVLQQLR	QLQQSMSSADSTQA
RPQGLPDNTASWF	CVNFNFNGLTGTGVLTESNK	CVNFNFNGLTGTGVLTESNK	QQTVTLLPAADLDDFSK
QLPQGTTLPK	CVNFNFNGLTGTGVLTESNKK	CVNFNFNGLTGTGVLTESNKK	RGPEQTQGNFGDQELTR
SWMESEFR	CYGVSPTK	CYGVSPTK	RPQGLPNNTASW
LDDKDPNFKDQVILLNK	DAPAHISTIGVCSMTDIAK	DAPAHISTIGVCSMTDIAK	RPQGLPNNTASWF
LTYTGAIK	DAPYIVGDVVQEGVLTAVVIPTK	DAPYIVGDVVQEGVLTAVVIPTK	RPQGLPNNTASWFTALTQHGK
GPEQTQGNFGDQELIR	DAPYIVGDVVQEGVLTAVVIPTKK	DAPYIVGDVVQEGVLTAVVIPTKK	RPQGLPNNTASWFTALTQHGKEDLK
GWIFGTTLDSK	DASGKPVPYCYDTNVLEGSVAYESLRPDTR	DASGKPVPYCYDTNVLEGSVAYESLRPDTR	SFIEDLLFNK
ITFGGPSDSTGSNQNGER	DATPSDFVR	DATPSDFVR	SMGTSPTRMAGNGGDAALALLLLDR
WYFYYLGTGPEAGLPY	DDKDPNFKDQVILLNK	DDKDPNFKDQVILLNK	SNLKPFER
RPQGLPNNTASW	DFGGFNFSQILPDPSKPSK	DFGGFNFSQILPDPSKPSK	TALTQHGKEDLK
LTQHGKEDLK	DFMSLSEQLR	DFMSLSEQLR	TALTQHGKEDLKFPR
GQGVPINTNSSPDDQIGYY	DFMSLSEQLRK	DFMSLSEQLRK	TATKAYNVTQAFGR
LGTGPEAGLPYGANK	DFYDFAVSK	DFYDFAVSK	TQHGKEDLKFPR
MAGNGGDAALALLLLDRLNQLESK	DGCVPLNIIPLTTAAK	DGCVPLNIIPLTTAAK	VAGDSGFAAYSR

PAADLDDFSK	DGHVETFYPK	DGHVETFYPK	VATEGALNTPK
HWPQIAQFAPSASAF	DGIIWVATEGALNTPK	DGIIWVATEGALNTPK	VGGNYNYLYR
IGMEVTPSGTWLTY	DGIIWVATEGALNTPKDHIGTR	DGIIWVATEGALNTPKDHIGTR	VTLADAGFIK
GFYAEGSRGGSEASSR	DGIIWVATEGALNTPKDHIGTRNPANNAAIVLQLPC	DGIIWVATEGALNTPKDHIGTRNPANNAAIVLQLPQGTT	VYSTGSNVFQTR
RPQGLPNNTASWFTAL	DGTCGLVEVEK	DGTCGLVEVEK	WVATEGALNTPK
KSNLKPFER	DHIGTRNPANNAAIVLQLPQGTTLPK	DHIGTRNPANNAAIVLQLPQGTTLPK	WYFYYLGTGPEAGLPYGANK
VGGNYNYLYR	DIADTTDAVR	DIADTTDAVR	YLGTGPEAGLPYGANK
LNTDHSSSSDNIALLVQ	DIADTTDAVRD	DIADTTDAVRD	YYLGTGPEAGLPYGANK
WYFYYLGTGPEAGLPYGANK	DIADTTDAVRDPQ	DIADTTDAVRDPQ	
RGPEQTQGNFGDQELTR	DIADTTDAVRDPQTLEILDITPC	DIADTTDAVRDPQTLEILDITPC	
NPANNAAIVLQLPQGT	DIASTDTCFANK	DIASTDTCFANK	
GGDAALALLLLDR	DLGACIDCSAR	DLGACIDCSAR	
TQLPPAYTNSFTR	DLPKEITVATSR	DLPKEITVATSR	
VTLADAGFIK	DLPQGFSALEPLVDLPIGINITR	DLPQGFSALEPLVDLPIGINITR	
NTNSSPDDQIGYYR	DLSPRWYFYYLGTGPEAGLPYGANK	DLSPRWYFYYLGTGPEAGLPYGANK	
FDNPVLPFNDGVYFASTEK	DLSPRWYFYYLGTGPEAGLPYGANKDGIIWVATE	DLSPRWYFYYLGTGPEAGLPYGANKDGIIWVATEGALNTPK	
FLPFQQFSR	DLYDKLQFTSLEIPR	DLYDKLQFTSLEIPR	
GWIFGTTLDPK	DNSYFTEQPIDLVPNQPYPNASFDNFK	DNSYFTEQPIDLVPNQPYPNASFDNFK	
AGDGGDAALALLLLDR	DPNFKDQVILLNK	DPNFKDQVILLNK	
AYIVTQAFGR	DPQTLEILDITPC	DPQTLEILDITPC	
FNGIGVTQNVLYENQK	DQNNVGPKVYPIILR	DQNNVGPKVYPIILR	
LIANQFNSAIGK	DQVILLNK	DQVILLNK	
SSPDDQIGYYR	DQVILLNKHIDAYK	DQVILLNKHIDAYK	
HWPQIAQF	DSNGTITVEELKK	DSNGTITVEELKK	
MAGDGGDAALALLLLDRLNQLESK	DVDTDFVNEFYAYLR	DVDTDFVNEFYAYLR	
TLLPAADLDDFSK	DWSYSGQSTQLGIEFLK	DWSYSGQSTQLGIEFLK	
YYLGTGPEAGLPYGANK	DWYDFVENPDILR	DWYDFVENPDILR	
NSSPDDQIGYYR	DYLASGGQPITNCVK	DYLASGGQPITNCVK	
SMGTSPTRMAGNGGDAALALLLLDR	EAPAHVSTIGVCTMTDIAK	EAPAHVSTIGVCTMTDIAK	
HWPQIAQFAPSASAFFGMSR	EETGLLMPLKAPK	EETGLLMPLKAPK	
FPQGQGVPINTNSSR	EEVKPFITESKPSVEQR	EEVKPFITESKPSVEQR	
LLDRLNQLESK	EFVFKNIDGYFK	EFVFKNIDGYFK	
RPQGLPNNTASWFTALTQHGKEDLKFPR	EGATTCGYLPQNAVVK	EGATTCGYLPQNAVVK	
RFDNPVLPFNDGVYFASTEK	EGFFTYICGFIQQK	EGFFTYICGFIQQK	
VQPTESIVR	EGIVWVATEGALNTPK	EGIVWVATEGALNTPK	
FTALTQHGKEDLK	EGIVWVATEGALNTPKDHIGTR	EGIVWVATEGALNTPKDHIGTR	
GQGVPINTNSSPDDQIGY	EGQINDMILSLLSK	EGQINDMILSLLSK	

GPEQTQGNFGDQELTR	EGSSVELK	EGSSVELK	
QGTDYKHWPQIAQFAPSASAFF	EGVFVSNGTHWFVTQR	EGVFVSNGTHWFVTQR	
ALALLLLDR	EHEHEIAWYTER	EHEHEIAWYTER	
VAGDSGFAAYSR	EIDRLNEVAK	EIDRLNEVAK	
LQDVVNQNAQALNTLVK	EIIFLEGETLPTEVLTEEVVLK	EIIFLEGETLPTEVLTEEVVLK	
ITFGGPSDSTGSNQDGERSGAR	EITVATSR	EITVATSR	
RPQGLPNNTASWFT	EITVATSRTLSYYK	EITVATSRTLSYYK	
MAGNGGDAAIALLLLDRLNQLESK	EITVATSRTLSYYKLGASQR	EITVATSRTLSYYKLGASQR	
DDKDPNFKDQVILLNK	EKVNINIVGDFK	EKVNINIVGDFK	
LQSLQTYVTQQLIR	ELGVVHNQDVNLHSSR	ELGVVHNQDVNLHSSR	
ALTGISVEQDKNTQEVFAQVK	ELLQNGMNGR	ELLQNGMNGR	
FLPFQQFGR	ELLVYAADPAMHAASGNLLLDKR	ELLVYAADPAMHAASGNLLLDKR	
HWPQIAQFAPSASAFFGMSRICMEVTPSGT	ELYHYQECVR	ELYHYQECVR	
SFIEDLLFNK	EMLAHAEETR	EMLAHAEETR	
TALTQHGKEDLK	EMLAHAEETRK	EMLAHAEETRK	
AGNGGDAALALLLLDRLNQLESK	ENDSKEGFFTYICGFIQQK	ENDSKEGFFTYICGFIQQK	
YWPQIAQFAPSASAFF	ENSYTTTIKPVTYK	ENSYTTTIKPVTYK	
VYSTGSNVFQTR	EPCSSGTYEGNSPFHPLADNK	EPCSSGTYEGNSPFHPLADNK	
DQVILLNK	EQIDGYVMHANYIFWR	EQIDGYVMHANYIFWR	
TLVATAEAELAK	ESPFELEDFIPMDSTVK	ESPFELEDFIPMDSTVK	
GFQPTNGVGYQPYR	ESVQTFFK	ESVQTFFK	
FTALTQHGK	ETLYCIDGALLTK	ETLYCIDGALLTK	
AGNGGDAALALLLLDR	ETMSYLFQHANLDSCK	ETMSYLFQHANLDSCK	
ALTGIAVEQDKNTQEVFAQVK	ETMSYLFQHANLDSCKR	ETMSYLFQHANLDSCKR	
FDNPVLPFNDGVYFSATEK	EVGFVVPGLPGTILR	EVGFVVPGLPGTILR	
GPEQTQGNFGDQELLR	FADDLNQLTGYK	FADDLNQLTGYK	
GWLFGTTLDSK	FADDLNQLTGYKKPASR	FADDLNQLTGYKKPASR	
ITFGGPSDSTGSNENGER	FALTCFSTQFAFACPDGVK	FALTCFSTQFAFACPDGVK	
LDDKDPNFKDQVLLLNK	FAPSASAFFGMSR	FAPSASAFFGMSR	
LQDSLSSTASALGK	FASVYAWNR	FASVYAWNR	
MAGDGGDAALALLLLDR	FCLEASFNYLK	FCLEASFNYLK	
NPANNAALVLQLPQGTTLPK	FDEDDSEPVLK	FDEDDSEPVLK	
RGPEQTQGNFGDEELIR	FDEDDSEPVLKG	FDEDDSEPVLKG	
RGPEQTQGNFGDQELLR	FDEDDSEPVLKGVK	FDEDDSEPVLKGVK	
SDNGPQNQRDAPR	FDNPVLPFNDGVYFASTEK	FDNPVLPFNDGVYFASTEK	
SFLEDLLFNK	FDTFNGECPNFVFPLNSIIK	FDTFNGECPNFVFPLNSIIK	
DDQIGYYR	FGDQELIR	FGDQELIR	

DDQIGYYRR	FGGPSDSTGSNQNGER	FGGPSDSTGSNQNGER	
DGILWVATEGALNTPKDHIGTR	FISTCACEIVGGQIVTCAK	FISTCACEIVGGQIVTCAK	
GIYQTSNFR	FKEGVEFLR	FKEGVEFLR	
GQGVPINTNSSR	FKESPFELEDFIPMDSTVK	FKESPFELEDFIPMDSTVK	
GQGVPINTNSSRDDQIGYYR	FKTEGLCVDIPGIPK	FKTEGLCVDIPGIPK	
LQEVVNQNAQALNTLVK	FLALCADSIIIGGAK	FLALCADSIIIGGAK	
NPANNAAIVLQLPEGTTLPK	FLPFQQFGR	FLPFQQFGR	
NSSRNSTPGSSIRTSPGMAGNGGDAALALLLLDR	FNFNGLTGTGVLTESNK	FNFNGLTGTGVLTESNK	
WYFYYLGTGPESGLPYGANK	FNGIGVTQNVLYENQK	FNGIGVTQNVLYENQK	
PGNGCDAALALLLLDR	FNGLTGTGVLTESNK	FNGLTGTGVLTESNK	
YLGTGPESGLPYGANK	FNGLTVLPPLLTD	FNGLTVLPPLLTD	
PNFKDQVILLNK	FNPPALQDAYYR	FNPPALQDAYYR	
TALTQHGKEDLKFPR	FPNITNLCPFGEVFNATR	FPNITNLCPFGEVFNATR	
NPANNAAIVL	FQEKDEDDNLIDSYFVVK	FQEKDEDDNLIDSYFVVK	
QQTVTLLPAADLDYFSK	FQPTNGVGYQPYR	FQPTNGVGYQPYR	
RGPEQTQGNFGDQEL	FQTLLALHR	FQTLLALHR	
NPANDAAIVLQLPQGTTLPK	FTALTQHGK	FTALTQHGK	
GTGPEAGLPYGANK	FTALTQHGKEDLK	FTALTQHGKEDLK	
DRLNQLESK	FTALTQHGKEDLKFPR	FTALTQHGKEDLKFPR	
TQHGKEDLKFPR	FTTTLNDFNLVAMK	FTTTLNDFNLVAMK	
ANNAAIVLQLPQGTTLPK	FVLALLSDLQDLK	FVLALLSDLQDLK	
AYNVTQAFGGR	FVSLAIDAYPLTK	FVSLAIDAYPLTK	
GPEQTQGNFGDQELIRR	FYDAQPCSDK	FYDAQPCSDK	
GQGVPIDTNSSPDDQIGYYR	GAGGHSYGADLK	GAGGHSYGADLK	
KQLTVTLLPAADLDDFSK	GANKDGIIWVATEGALNTPK	GANKDGIIWVATEGALNTPK	
LVDPQIQLAVTR	GAWNIGEQK	GAWNIGEQK	
MAGNGNDAALALLLLDR	GCCSCGSCCKFDEDDSEPVLK	GCCSCGSCCKFDEDDSEPVLK	
NSSRTSTPGSSK	GDAALALLLLDR	GDAALALLLLDR	
QLTVTLLPAADLDDFSK	GDYGDAVVYR	GDYGDAVVYR	
DLPIEITVATSRTLSYYK	GEDIQLLK	GEDIQLLK	
GEGVPINTNSSPDDQIGYYR	GFGDSVEEVLSEAR	GFGDSVEEVLSEAR	
LLPAADLDDFFK	GFQPTNGVGYQPYR	GFQPTNGVGYQPYR	
MAGDAALALL	GFYAEGSR	GFYAEGSR	
NPANNAAIVIQLPQGTTLPK	GFYAEGSRG	GFYAEGSRG	
NPANNAAIVLQIPQGTTLPK	GFYAEGSRGG	GFYAEGSRGG	
NSIPGSSMGTSPARMA	GFYAEGSRGGSQA	GFYAEGSRGGSQA	
NSTPGSSKPTSPARMAGNGGDAA	GFYAEGSRGGSQAS	GFYAEGSRGGSQAS	

SDNGPQNLPNAPR	GFYAEGSRGGSQASSR	GFYAEGSRGGSQASSR	
SILSPLYAFASEAAR	GGDAALALLLLDR	GGDAALALLLLDR	
SLVPGFNEK	GGDAALALLLLDRLNQLESK	GGDAALALLLLDRLNQLESK	
SLNVAKSEFDRDAAMQR	GGDGKMKDLSPR	GGDGKMKDLSPR	
IGAGICASYQTQTNSPR	GGSYTNDKACPLIAAVITR	GGSYTNDKACPLIAAVITR	
NLNESLIDLQVL	GHFDGQQGEVPVSIINNTVYTK	GHFDGQQGEVPVSIINNTVYTK	
NSTPGSSMGTSPAR	GIGVTQNVLYENQK	GIGVTQNVLYENQK	
AYETQALPQR	GIIWVATEGALNTPK	GIIWVATEGALNTPK	
KAYETQALPQR	GIYQTSNFR	GIYQTSNFR	
LNQLGSKMSG	GLPNNTASWFTALTQHGK	GLPNNTASWFTALTQHGK	
NGIIWVATEGALNTPK	GLPWNVVR	GLPWNVVR	
LNQLESKMAGK	GLTGTGVLTESNK	GLTGTGVLTESNK	
LGSPLSLNMAR	GMVLGSLAATVR	GMVLGSLAATVR	
AFQLTPIAVQMTK	GNGGDAALALLLLDR	GNGGDAALALLLLDR	
ITFGGPSDSTGSNQNGGAR	GPEQTQGNFGDQELIR	GPEQTQGNFGDQELIR	
RATRRIPGGDGK	GPHEFCSQHTMLVK	GPHEFCSQHTMLVK	
VTSPARMAGN	GPITDVFYK	GPITDVFYK	
ITFGGPSDSTGSNQNGGQR	GPITDVFYKENSYTTTIKPVTYK	GPITDVFYKENSYTTTIKPVTYK	
LNKVEAEVQIDR	GQGVPINTNSSPDDQIGY	GQGVPINTNSSPDDQIGY	
	GQGVPINTNSSPDDQIGYY	GQGVPINTNSSPDDQIGYY	
	GQGVPINTNSSPDDQIGYYR	GQGVPINTNSSPDDQIGYYR	
	GQGVPINTNSSPDDQIGYYRR	GQGVPINTNSSPDDQIGYYRR	
	GQGVPINTNSSPDDQIGYYRRATR	GQGVPINTNSSPDDQIGYYRRATR	
	GQQQQGQTVTK	GQQQQGQTVTK	
	GQQQQGQTVTKK	GQQQQGQTVTKK	
	GQQQQGQTVTKKSAAEASK	GQQQQGQTVTKKSAAEASK	
	GQQQQGQTVTKKSAAEASKKPR	GQQQQGQTVTKKSAAEASKKPR	
	GSLPINVIVFDGK	GSLPINVIVFDGK	
	GTGPEAGLPYGANK	GTGPEAGLPYGANK	
	GTLEPEYFNSVCR	GTLEPEYFNSVCR	
	GTTVLLKEPCSSGTYEGNSPFHPLADNK	GTTVLLKEPCSSGTYEGNSPFHPLADNK	
	GVAPGTAVLR	GVAPGTAVLR	
	GVEAVMYMGTLSYEQFK	GVEAVMYMGTLSYEQFK	
	GVEAVMYMGTLSYEQFKK	GVEAVMYMGTLSYEQFKK	
	GVITHDVSSAINRPQIGVVR	GVITHDVSSAINRPQIGVVR	
	GVLPQLEQPYVFIK	GVLPQLEQPYVFIK	
	GVLPQLEQPYVFIKR	GVLPQLEQPYVFIKR	

	GVQIPCTCGK	GVQIPCTCGK	
	GVVFLHVTYVPAQEK	GVVFLHVTYVPAQEK	
	GVYFASTEK	GVYFASTEK	
	GVYYPDK	GVYYPDK	
	GVYYPDKVFR	GVYYPDKVFR	
	GWIFGTTLDSK	GWIFGTTLDSK	
	GYHLMSFPQSAPH	GYHLMSFPQSAPH	
	GYHLMSFPQSAPHGVVFLHVTYVPAQEK	GYHLMSFPQSAPHGVVFLHVTYVPAQEK	
	HADFDTWFSQR	HADFDTWFSQR	
	HCLHVVGPNVNK	HCLHVVGPNVNK	
	HCLHVVGPNVNKGEDIQLLK	HCLHVVGPNVNKGEDIQLLK	
	HFDEGNCDTLK	HFDEGNCDTLK	
	HGGGVAGALNK	HGGGVAGALNK	
	HGTFTCASEYTGNYQCGHYK	HGTFTCASEYTGNYQCGHYK	
	HIDAYKTFPPTEPK	HIDAYKTFPPTEPK	
	HSLSHFVNLDNLR	HSLSHFVNLDNLR	
	HTDFSSEIIGYK	HTDFSSEIIGYK	
	HTFSNYQHEETIYNLLK	HTFSNYQHEETIYNLLK	
	HTPINLVR	HTPINLVR	
	HVICTSEDMLNPNYEDLLIR	HVICTSEDMLNPNYEDLLIR	
	HVICTSEDMLNPNYEDLLIRK	HVICTSEDMLNPNYEDLLIRK	
	HWPQIAQF	HWPQIAQF	
	HWPQIAQFAPSASAF	HWPQIAQFAPSASAF	
	HWPQIAQFAPSASAFF	HWPQIAQFAPSASAFF	
	HWPQIAQFAPSASAFFGM	HWPQIAQFAPSASAFFGM	
	HWPQIAQFAPSASAFFGMSR	HWPQIAQFAPSASAFFGMSR	
	HYVYIGDPAQLPAPR	HYVYIGDPAQLPAPR	
	IADYNYK	IADYNYK	
	IADYNYKLPDDFTGCVIAWNSNNLDSK	IADYNYKLPDDFTGCVIAWNSNNLDSK	
	IAEIPKEEVKPFITESKPSVEQR	IAEIPKEEVKPFITESKPSVEQR	
	IAGHHLGR	IAGHHLGR	
	IAQFAPSASAFFGMSR	IAQFAPSASAFFGMSR	
	IFTIGTVTLK	IFTIGTVTLK	
	IFVDGVPFVVSTGYHFR	IFVDGVPFVVSTGYHFR	
	IGMEVTPSGTWLTY	IGMEVTPSGTWLTY	
	IGMEVTPSGTWLTYTGAIK	IGMEVTPSGTWLTYTGAIK	
	IGMEVTPSGTWLTYTGAIKLDDKDPNFK	IGMEVTPSGTWLTYTGAIKLDDKDPNFK	

	IGMEVTPSGTWLTYTGAIKLDDKDPNFKDQVILLNK	IGMEVTPSGTWLTYTGAIKLDDKDPNFKDQVILLNK	
	IGNYKLNTDHSSSSDNIALLVQ	IGNYKLNTDHSSSSDNIALLVQ	
	ILGAGCFVDDIVK	ILGAGCFVDDIVK	
	IMASLVLAR	IMASLVLAR	
	IMTWLDMVDTSLSGFK	IMTWLDMVDTSLSGFK	
	IQDSLSSTASALGK	IQDSLSSTASALGK	
	IQEGVVDYGAR	IQEGVVDYGAR	
	IRGGDGKMKDLSPR	IRGGDGKMKDLSPR	
	ISEMHPALR	ISEMHPALR	
	ISNCVADYSVLYNSASFSTFK	ISNCVADYSVLYNSASFSTFK	
	ITEEVGHTDLMAAYVDNSSLTIK	ITEEVGHTDLMAAYVDNSSLTIK	
	ITEHSWNADLYK	ITEHSWNADLYK	
	ITFGGPSDSTGSNQNGER	ITFGGPSDSTGSNQNGER	
	ITFGGPSDSTGSNQNGERSGAR	ITFGGPSDSTGSNQNGERSGAR	
	ITGLYPTLNISDEFSSNVANYQK	ITGLYPTLNISDEFSSNVANYQK	
	IVDEPEEHVQIH	IVDEPEEHVQIH	
	IVDEPEEHVQIHTI	IVDEPEEHVQIHTI	
	IVDEPEEHVQIHTID	IVDEPEEHVQIHTID	
	IVDEPEEHVQIHTIDG	IVDEPEEHVQIHTIDG	
	IVDEPEEHVQIHTIDGSSGVVNPVMEPIYDEPTTTT	IVDEPEEHVQIHTIDGSSGVVNPVMEPIYDEPTTTTSVPL	
	IVITSGDGTTSPISEHDYQIGGYTEK	IVITSGDGTTSPISEHDYQIGGYTEK	
	IVLQLPQGTTLPK	IVLQLPQGTTLPK	
	IVQLSEISMDNSPNLAWPLIVTALR	IVQLSEISMDNSPNLAWPLIVTALR	
	IVYTACSHAAVDALCEK	IVYTACSHAAVDALCEK	
	IWVATEGALNTPK	IWVATEGALNTPK	
	IYCPACHNSEVGPEHSLAEYHNESGLK	IYCPACHNSEVGPEHSLAEYHNESGLK	
	IYSKHTPINLVR	IYSKHTPINLVR	
	KADETQALPQ	KADETQALPQ	
	KADETQALPQR	KADETQALPQR	
	KADETQALPQRQ	KADETQALPQRQ	
	KADETQALPQRQK	KADETQALPQRQK	
	KADETQALPQRQKK	KADETQALPQRQKK	
	KADETQALPQRQR	KADETQALPQRQR	
	KADETQALPQRQRQK	KADETQALPQRQRQK	
	KDAPYIVGDVVQEGVLTAVVIPTKK	KDAPYIVGDVVQEGVLTAVVIPTKK	
	KDGIIWVATEGALNTPK	KDGIIWVATEGALNTPK	
	KDKKKKADETQALPQR	KDKKKKADETQALPQR	

	KDNSYFTEQPIDLVPNQPYPNASFDNFK	KDNSYFTEQPIDLVPNQPYPNASFDNFK	
	KFDTFNGECPNFVFPLNSIIK	KFDTFNGECPNFVFPLNSIIK	
	KKADETQALPQR	KKADETQALPQR	
	KKADETQALPQRQK	KKADETQALPQRQK	
	KKKADETQALPQR	KKKADETQALPQR	
	KLDNDALNNIINNAR	KLDNDALNNIINNAR	
	KLMPVCVETK	KLMPVCVETK	
	KPTETICAPLTVFFDGR	KPTETICAPLTVFFDGR	
	KQQTVTLLPAADLDD	KQQTVTLLPAADLDD	
	KQQTVTLLPAADLDDFSK	KQQTVTLLPAADLDDFSK	
	KQQTVTLLPAADLDDFSKQLQQSMSSADSTQA	KQQTVTLLPAADLDDFSKQLQQSMSSADSTQA	
	KSAAEASK	KSAAEASK	
	KSAPLIELCVDEAGSK	KSAPLIELCVDEAGSK	
	KSNHNFLVQAGNVQLR	KSNHNFLVQAGNVQLR	
	KSNLKPFER	KSNLKPFER	
	KTLNSLEDK	KTLNSLEDK	
	KTLNSLEDKAFQLTPIAVQMTK	KTLNSLEDKAFQLTPIAVQMTK	
	KVDGVVQQLPETYFTQSR	KVDGVVQQLPETYFTQSR	
	KVKPTVVVNAANVYLK	KVKPTVVVNAANVYLK	
	KVPTDNYITTYPGQGLNGYTVEEAK	KVPTDNYITTYPGQGLNGYTVEEAK	
	LATTEELPDEFVVVTVK	LATTEELPDEFVVVTVK	
	LCEEMLDNR	LCEEMLDNR	
	LDDKDPNFK	LDDKDPNFK	
	LDDKDPNFKDQVILLNK	LDDKDPNFKDQVILLNK	
	LDDKDPNFKDQVILLNKHIDAYK	LDDKDPNFKDQVILLNKHIDAYK	
	LDDKDPQFKDNVILLNK	LDDKDPQFKDNVILLNK	
	LDDKDPQFKDNVILLNKHIDAYK	LDDKDPQFKDNVILLNKHIDAYK	
	LDGVVCTEIDPK	LDGVVCTEIDPK	
	LDKVEAEVQIDR	LDKVEAEVQIDR	
	LDNDALNNIINNAR	LDNDALNNIINNAR	
	LGASQRVAGDSGFAAYSR	LGASQRVAGDSGFAAYSR	
	LGSPLSLNMAR	LGSPLSLNMAR	
	LGTGPEAGLPYGANK	LGTGPEAGLPYGANK	
	LHNWNCVNCDTFCAGSTFISDEVAR	LHNWNCVNCDTFCAGSTFISDEVAR	
	LHVTYVPAQEK	LHVTYVPAQEK	
	LIANQFNSAIGK	LIANQFNSAIGK	
	LIANQFNSAIGKIQDSLSSTASALGK	LIANQFNSAIGKIQDSLSSTASALGK	

	LKPVLDWLEEK	LKPVLDWLEEK	
	LKTLVATAEAELAK	LKTLVATAEAELAK	
	LKVDTANPK	LKVDTANPK	
	LLDRLNQLESK	LLDRLNQLESK	
	LLHKPIVW	LLHKPIVW	
	LLHKPIVWHVNNATNK	LLHKPIVWHVNNATNK	
	LLPAADLDDFSK	LLPAADLDDFSK	
	LMPVCVETK	LMPVCVETK	
	LMVVIPDYNTYK	LMVVIPDYNTYK	
	LNDLCFTNVYADSFVIR	LNDLCFTNVYADSFVIR	
	LNDLCFTNVYADSFVIRGDEVR	LNDLCFTNVYADSFVIRGDEVR	
	LNQLESKMSGK	LNQLESKMSGK	
	LNTDHSSSSDNIALLVQ	LNTDHSSSSDNIALLVQ	
	LPDDFTGCVIAWNSNNLDSK	LPDDFTGCVIAWNSNNLDSK	
	LQAGNATEVPANSTVLSFCAFAVDAAK	LQAGNATEVPANSTVLSFCAFAVDAAK	
	LQDVVNQNAQALN	LQDVVNQNAQALN	
	LQDVVNQNAQALNTLVK	LQDVVNQNAQALNTLVK	
	LQFTSLEIPR	LQFTSLEIPR	
	LQNNELSPVALR	LQNNELSPVALR	
	LQSLENVAFNVVNK	LQSLENVAFNVVNK	
	LQSLQTYVTQQLIR	LQSLQTYVTQQLIR	
	LRSDVLLPLTQY	LRSDVLLPLTQY	
	LSHQSDIEVTGDSCNNYMLTYNK	LSHQSDIEVTGDSCNNYMLTYNK	
	LSYGIATVR	LSYGIATVR	
	LTDNVYIK	LTDNVYIK	
	LTPCGTGTSTDVVYR	LTPCGTGTSTDVVYR	
	LTQHGKEDLK	LTQHGKEDLK	
	LVDPQIQLAVTR	LVDPQIQLAVTR	
	LVSSFLEMK	LVSSFLEMK	
	MADQAMTQMYK	MADQAMTQMYK	
	MADSNGTITVEELKK	MADSNGTITVEELKK	
	MAGNGGDAALALLLLDR	MAGNGGDAALALLLLDR	
	MAGNGGDAALALLLLDRLN	MAGNGGDAALALLLLDRLN	
	MAGNGGDAALALLLLDRLNQLESK	MAGNGGDAALALLLLDRLNQLESK	
	MAGNGGDAALALLLLDRLNQLESKMSGK	MAGNGGDAALALLLLDRLNQLESKMSGK	
	MASGGGETALALLLLDR	MASGGGETALALLLLDR	
	MASGGGETALALLLLDRLNQLESK	MASGGGETALALLLLDRLNQLESK	

	MENAVGRDQNNVGPK	MENAVGRDQNNVGPK	
	MESLVPGFNEK	MESLVPGFNEK	
	MFDAYVNTFSSTFNVPMEK	MFDAYVNTFSSTFNVPMEK	
	MFVFLVLLPLVSSQCVNLTTR	MFVFLVLLPLVSSQCVNLTTR	
	MKDLSPRWYFYYLGTGPEAGLPYGANK	MKDLSPRWYFYYLGTGPEAGLPYGANK	
	MNYQVNGYPNMFITR	MNYQVNGYPNMFITR	
	MSECVLGQSK	MSECVLGQSK	
	MSECVLGQSKR	MSECVLGQSKR	
	MSGKGQQQQGQTVTK	MSGKGQQQQGQTVTK	
	MSGKGQQQQGQTVTKK	MSGKGQQQQGQTVTKK	
	NAAIVLQLPQGTTLPK	NAAIVLQLPQGTTLPK	
	NADIVEEAK	NADIVEEAK	
	NADIVEEAKK	NADIVEEAKK	
	NAPRITFGGPSDSTGSNQNGER	NAPRITFGGPSDSTGSNQNGER	
	NDGVYFASTEK	NDGVYFASTEK	
	NFNGLTGTGVLTESNKK	NFNGLTGTGVLTESNKK	
	NFTTAPAICHDGK	NFTTAPAICHDGK	
	NFTTAPAICHEGK	NFTTAPAICHEGK	
	NGSIHLYFDK	NGSIHLYFDK	
	NGVLITEGSVK	NGVLITEGSVK	
	NHTSPDVDLGDISGINASVVNIQK	NHTSPDVDLGDISGINASVVNIQK	
	NIDGYFK	NIDGYFK	
	NIKPVPEVK	NIKPVPEVK	
	NLNESLIDLQELGK	NLNESLIDLQELGK	
	NLNSSRVPDLLV	NLNSSRVPDLLV	
	NLYDKLVSSFLEMK	NLYDKLVSSFLEMK	
	NNELSPVALR	NNELSPVALR	
	NPANNAAIVLQLPQGT	NPANNAAIVLQLPQGT	
	NPANNAAIVLQLPQGTTLPK	NPANNAAIVLQLPQGTTLPK	
	NPANNAAIVLQLPQGTTLPKG	NPANNAAIVLQLPQGTTLPKG	
	NPANNAAIVLQLPQGTTLPKGFYAEGSR	NPANNAAIVLQLPQGTTLPKGFYAEGSR	
	NPANNAAIVLQLPQGTTLPKGFYAEGSRG	NPANNAAIVLQLPQGTTLPKGFYAEGSRG	
	NPLLYDANYFL	NPLLYDANYFL	
	NPLLYDANYFLCWH	NPLLYDANYFLCWH	
	NSIDAFKLNIK	NSIDAFKLNIK	
	NSSPDDQIGYYR	NSSPDDQIGYYR	
	NSTPGSSR	NSTPGSSR	

	NSTPGSSRGTSPA	NSTPGSSRGTSPA	
	NTASWFTALTQHGK	NTASWFTALTQHGK	
	NTASWFTALTQHGKEDLK	NTASWFTALTQHGKEDLK	
	NTNPIQLSSYSLFDMSK	NTNPIQLSSYSLFDMSK	
	NTNSSPDDQIGYYR	NTNSSPDDQIGYYR	
	NTQEVFAQVK	NTQEVFAQVK	
	NTVCTVCGMWK	NTVCTVCGMWK	
	NVATLQAENVTGLFK	NVATLQAENVTGLFK	
	NVIPTITQMNLK	NVIPTITQMNLK	
	NVSLDNVLSTFISAAR	NVSLDNVLSTFISAAR	
	NVTQAFGR	NVTQAFGR	
	NYFITDAQTGSSK	NYFITDAQTGSSK	
	NYVFTGYR	NYVFTGYR	
	PAADLDDFSK	PAADLDDFSK	
	PANNAAIVLQLPQGTTLPK	PANNAAIVLQLPQGTTLPK	
	PDDQIGYYR	PDDQIGYYR	
	PINTNSSPDDQIGYYRR	PINTNSSPDDQIGYYRR	
	PLLESELVIGAVILR	PLLESELVIGAVILR	
	PNFKDQVILLNK	PNFKDQVILLNK	
	PNNTASWFTALTQHGK	PNNTASWFTALTQHGK	
	PPAYTNSFTR	PPAYTNSFTR	
	PQGLPNNTASWFTALTQHGK	PQGLPNNTASWFTALTQHGK	
	PQGLPNNTASWFTALTQHGKEDLK	PQGLPNNTASWFTALTQHGKEDLK	
	PQIAQFAPSASAFFGMSR	PQIAQFAPSASAFFGMSR	
	PSASAFFGMSR	PSASAFFGMSR	
	PSFYVYSR	PSFYVYSR	
	PSGTWLTYTGAIK	PSGTWLTYTGAIK	
	PSSKRFQPFQQFGR	PSSKRFQPFQQFGR	
	PVETSNSFDVLK	PVETSNSFDVLK	
	QASLNGVTLIGEAVK	QASLNGVTLIGEAVK	
	QEILGTVSWNLR	QEILGTVSWNLR	
	QFDTYNLWNTFTR	QFDTYNLWNTFTR	
	QGDDYVYLPYPDPSR	QGDDYVYLPYPDPSR	
	QGEIKDATPSDFVR	QGEIKDATPSDFVR	
	QGFVDSDVETK	QGFVDSDVETK	
	QGFVDSDVETKDVVECLK	QGFVDSDVETKDVVECLK	
	QGTDYKHWPQIAQFAPSASAFFGMSR	QGTDYKHWPQIAQFAPSASAFFGMSR	

	QHLKDGTCGLVEVEK	QHLKDGTCGLVEVEK	
	QIAPGQTGK	QIAPGQTGK	
	QIVESCGNFK	QIVESCGNFK	
	QKKQQTVTLLPAADLDDFSK	QKKQQTVTLLPAADLDDFSK	
	QKRTATKAYNVTQAFGR	QKRTATKAYNVTQAFGR	
	QLPFFYYSDSPCESHGK	QLPFFYYSDSPCESHGK	
	QLPQGTTLPK	QLPQGTTLPK	
	QLQQSMSSADSTQA	QLQQSMSSADSTQA	
	QLSSNFGAISSVLNDILSR	QLSSNFGAISSVLNDILSR	
	QPTNGVGYQPYR	QPTNGVGYQPYR	
	QQQGQTVTKK	QQQGQTVTKK	
	QQTVTLLPAADLDDFSK	QQTVTLLPAADLDDFSK	
	QSYGFQPTNGVGYQPYR	QSYGFQPTNGVGYQPYR	
	QVVNVVTTK	QVVNVVTTK	
	QVVSDIDYVPLK	QVVSDIDYVPLK	
	QYGDCLGDIAAR	QYGDCLGDIAAR	
	QYNVTQAFGR	QYNVTQAFGR	
	RCPAEIVDTVSALVYDNK	RCPAEIVDTVSALVYDNK	
	RFDNPVLPFNDGVYFASTEK	RFDNPVLPFNDGVYFASTEK	
	RGPEQTQGNFGDQDLIR	RGPEQTQGNFGDQDLIR	
	RGPEQTQGNFGDQELIR	RGPEQTQGNFGDQELIR	
	RGPEQTQGNFGDQELIRQ	RGPEQTQGNFGDQELIRQ	
	RGQGVPINTNSSPDDQIGYYR	RGQGVPINTNSSPDDQIGYYR	
	RITFGGPSDSTGSNQNGER	RITFGGPSDSTGSNQNGER	
	RNPANNAAIVLQLPQGTTLPK	RNPANNAAIVLQLPQGTTLPK	
	RPINPTDQSSYIVDSVTVK	RPINPTDQSSYIVDSVTVK	
	RPQGLPNNTASW	RPQGLPNNTASW	
	RPQGLPNNTASWF	RPQGLPNNTASWF	
	RPQGLPNNTASWFT	RPQGLPNNTASWFT	
	RPQGLPNNTASWFTAL	RPQGLPNNTASWFTAL	
	RPQGLPNNTASWFTALTQH	RPQGLPNNTASWFTALTQH	
	RPQGLPNNTASWFTALTQHGK	RPQGLPNNTASWFTALTQHGK	
	RPQGLPNNTASWFTALTQHGKEDLK	RPQGLPNNTASWFTALTQHGKEDLK	
	RPQGLPNNTASWFTALTQHGKEDLKFPR	RPQGLPNNTASWFTALTQHGKEDLKFPR	
	RPQGLPNNTASWFTALTQHGKEELR	RPQGLPNNTASWFTALTQHGKEELR	
	RSFIEDLLFNK	RSFIEDLLFNK	
	RTATKAYNVTQAFGR	RTATKAYNVTQAFGR	

	RWQLALSK	RWQLALSK	
	SADAQSFLNR	SADAQSFLNR	
	SAFYILPSIISNEK	SAFYILPSIISNEK	
	SAGFPFNK	SAGFPFNK	
	SALEPLVDLPIGINITR	SALEPLVDLPIGINITR	
	SAPLIELCVDEAGSK	SAPLIELCVDEAGSK	
	SAYENFNQHEVLLAPLLSAGIFGADPIHSLR	SAYENFNQHEVLLAPLLSAGIFGADPIHSLR	
	SDVLLPLTQYNR	SDVLLPLTQYNR	
	SEAGVCVSTSGR	SEAGVCVSTSGR	
	SEDAQGMDNLACEDLKPVSEEVVENPTIQK	SEDAQGMDNLACEDLKPVSEEVVENPTIQK	
	SFIEDLLFNK	SFIEDLLFNK	
	SFIEDLLFNKVTLADAGFIK	SFIEDLLFNKVTLADAGFIK	
	SFNPETNILLNVPLHGTILTRPLLESELVIGAVILR	SFNPETNILLNVPLHGTILTRPLLESELVIGAVILR	
	SFYVYANGGK	SFYVYANGGK	
	SGDGTTSPISEHDYQIGGYTEK	SGDGTTSPISEHDYQIGGYTEK	
	SGETLGVLVPHVGEIPVAYR	SGETLGVLVPHVGEIPVAYR	
	SGETLGVLVPHVGEIPVAYRK	SGETLGVLVPHVGEIPVAYRK	
	SHKPPISFPLCANGQVFGLYK	SHKPPISFPLCANGQVFGLYK	
	SHNIALIWNVK	SHNIALIWNVK	
	SILSPLYAFASEAAR	SILSPLYAFASEAAR	
	SIVITSGDGTTSPISEHDYQIGGYTEK	SIVITSGDGTTSPISEHDYQIGGYTEK	
	SLENVAFNVVNK	SLENVAFNVVNK	
	SLKVPATVSVSSPDAVTAYNGYLTSSSK	SLKVPATVSVSSPDAVTAYNGYLTSSSK	
	SLTENKYSQLDEEQPMEID	SLTENKYSQLDEEQPMEID	
	SMWSFNPETNILLNVPLHGTILTR	SMWSFNPETNILLNVPLHGTILTR	
	SNGTITVEELKK	SNGTITVEELKK	
	SNHNFLVQAGNVQLR	SNHNFLVQAGNVQLR	
	SNLKPFER	SNLKPFER	
	SPDDQIGYYR	SPDDQIGYYR	
	SQDLSVVSK	SQDLSVVSK	
	SREETGLLMPLK	SREETGLLMPLK	
	SREETGLLMPLKAPK	SREETGLLMPLKAPK	
	SSEYKGPITDVFYK	SSEYKGPITDVFYK	
	SSEYKGPITDVFYKENSYTTTIKPVTYK	SSEYKGPITDVFYKENSYTTTIKPVTYK	
	SSGTYEGNSPFHPLADNK	SSGTYEGNSPFHPLADNK	
	SSPDDQIGYYR	SSPDDQIGYYR	
	SSPDDQIGYYRR	SSPDDQIGYYRR	

	SSVLHSTQDLFLPF	SSVLHSTQDLFLPF	
	STDTGVEHVTFFIYNK	STDTGVEHVTFFIYNK	
	SVLYNSASFSTFK	SVLYNSASFSTFK	
	SVLYYQNNVFMSEAK	SVLYYQNNVFMSEAK	
	SVNITFELDER	SVNITFELDER	
	SVTSSIVITSGDGTTSPISEHDYQIGGYTEK	SVTSSIVITSGDGTTSPISEHDYQIGGYTEK	
	SVYPVASPNECNQMCLSTLMK	SVYPVASPNECNQMCLSTLMK	
	SVYYTSNPTTFHLDGEVITFDNLK	SVYYTSNPTTFHLDGEVITFDNLK	
	SWFTALTQHGKEDLK	SWFTALTQHGKEDLK	
	SWMESEFR	SWMESEFR	
	SYELQTPFEIK	SYELQTPFEIK	
	SYLTPGDSSSGW	SYLTPGDSSSGW	
	SYLTPGDSSSGWTAGAAAYYVGYLQPR	SYLTPGDSSSGWTAGAAAYYVGYLQPR	
	SYYKLGASQR	SYYKLGASQR	
	TAGAAAYYVGYLQPR	TAGAAAYYVGYLQPR	
	TALTQHGKEDLK	TALTQHGKEDLK	
	TALTQHGKEDLKFPR	TALTQHGKEDLKFPR	
	TASWFTALTQHGK	TASWFTALTQHGK	
	TATKAYNVTQAFGR	TATKAYNVTQAFGR	
	TATKQYNVTQAFGR	TATKQYNVTQAFGR	
	TCGQQQTTLK	TCGQQQTTLK	
	TDGTLMIER	TDGTLMIER	
	TFPPTEPK	TFPPTEPK	
	TFPPTEPKK	TFPPTEPKK	
	TFPPTEPKKDK	TFPPTEPKKDK	
	TFPPTEPKKDKK	TFPPTEPKKDKK	
	TFPPTEPKKDKKK	TFPPTEPKKDKKK	
	TFYVLPNDDTLR	TFYVLPNDDTLR	
	THVQLSLPVLQVR	THVQLSLPVLQVR	
	TIAFGGCVFSYVGCHNK	TIAFGGCVFSYVGCHNK	
	TIGPDMFLGTCR	TIGPDMFLGTCR	
	TILGSALLEDEFTPFDVVR	TILGSALLEDEFTPFDVVR	
	TLADAGFIK	TLADAGFIK	
	TLATHGLAAVNSVPWDTIANYAK	TLATHGLAAVNSVPWDTIANYAK	
	TLETAQNSVR	TLETAQNSVR	
	TLLPAADLDDFSK	TLLPAADLDDFSK	
	TLNSLEDK	TLNSLEDK	

	TLNSLEDKAFQLTPIAVQMTK	TLNSLEDKAFQLTPIAVQMTK	
	TLNSLEDKAFQLTPIAVQMTKLATTEELPDEFVVVT	TLNSLEDKAFQLTPIAVQMTKLATTEELPDEFVVVTVK	
	TLSYYKLGASQR	TLSYYKLGASQR	
	TLSYYKLGASQRVAGDSGFAAYSR	TLSYYKLGASQRVAGDSGFAAYSR	
	TLVATAEAELAK	TLVATAEAELAK	
	TNSSPDDQIGYYR	TNSSPDDQIGYYR	
	TNSSPDDQIGYYRR	TNSSPDDQIGYYRR	
	TNVYADSFVIR	TNVYADSFVIR	
	TNVYLAVFDK	TNVYLAVFDK	
	TNVYLAVFDKNLYDK	TNVYLAVFDKNLYDK	
	TPEEHFIETISLAGSYK	TPEEHFIETISLAGSYK	
	TPPIKDFGGFNFSQILPDPSKPSK	TPPIKDFGGFNFSQILPDPSKPSK	
	TQGNFGDQELIR	TQGNFGDQELIR	
	TQLPPAYTNSFTR	TQLPPAYTNSFTR	
	TQSLLIVNNATNVVIK	TQSLLIVNNATNVVIK	
	TTEVVGDIILKPANNSLK	TTEVVGDIILKPANNSLK	
	TTLPVNVAFELWAK	TTLPVNVAFELWAK	
	TTNGDFLHFLPR	TTNGDFLHFLPR	
	TVAGVSICSTMTNR	TVAGVSICSTMTNR	
	TVGELGDVR	TVGELGDVR	
	TVYSDVENPHLMGWDYPK	TVYSDVENPHLMGWDYPK	
	VAGDSGFAAY	VAGDSGFAAY	
	VAGDSGFAAYSR	VAGDSGFAAYSR	
	VAGDSGFAAYSRYR	VAGDSGFAAYSRYR	
	VATEGALNTPK	VATEGALNTPK	
	VCEFQFCNDPFLGVYYHK	VCEFQFCNDPFLGVYYHK	
	VCGVSAAR	VCGVSAAR	
	VDGQVDLFR	VDGQVDLFR	
	VDGVDVELFENK	VDGVDVELFENK	
	VDGVVQQLPETYFTQSR	VDGVVQQLPETYFTQSR	
	VEAEVQIDR	VEAEVQIDR	
	VEAFEYYHTTDPSFLGR	VEAFEYYHTTDPSFLGR	
	VECTTIVNGVR	VECTTIVNGVR	
	VFSAVGNICYTPSK	VFSAVGNICYTPSK	
	VGGNYNYLYR	VGGNYNYLYR	
	VGGSCLLSGHNLAK	VGGSCLLSGHNLAK	
	VGGSCVLSGHNLAK	VGGSCVLSGHNLAK	

	VIGHSMQNCVLK	VIGHSMQNCVLK	
	VIHFGAGSDK	VIHFGAGSDK	
	VKPTVVVNAANVYLK	VKPTVVVNAANVYLK	
	VNINIVGDFK	VNINIVGDFK	
	VPATVSVSSPDAVTAYNGYLTSSSK	VPATVSVSSPDAVTAYNGYLTSSSK	
	VPTDNYITTYPGQGLNGYTVEEAK	VPTDNYITTYPGQGLNGYTVEEAK	
	VQIGEYTFEK	VQIGEYTFEK	
	VQPTESIVR	VQPTESIVR	
	VTFFPDLNGDVVAIDYK	VTFFPDLNGDVVAIDYK	
	VTFGDDTVIEVQGYK	VTFGDDTVIEVQGYK	
	VTLADAGFIK	VTLADAGFIK	
	VTSAMQTMLFTMLR	VTSAMQTMLFTMLR	
	VVNQNAQALNTLVK	VVNQNAQALNTLVK	
	VVSTTTNIVTR	VVSTTTNIVTR	
	VVTTFDSEYCR	VVTTFDSEYCR	
	VVVLSFELLHAPATVCGPK	VVVLSFELLHAPATVCGPK	
	VYANLGER	VYANLGER	
	VYPIILRL	VYPIILRL	
	VYSSANNCTFEYVSQPFLMDLEGK	VYSSANNCTFEYVSQPFLMDLEGK	
	VYSTGSNVFQTR	VYSTGSNVFQTR	
	WKYPQVNGLTSIK	WKYPQVNGLTSIK	
	WVATEGALNTPK	WVATEGALNTPK	
	WVLNNDYYR	WVLNNDYYR	
	WYFYYLGTGPEAGLPYGAN	WYFYYLGTGPEAGLPYGAN	
	WYFYYLGTGPEAGLPYGANK	WYFYYLGTGPEAGLPYGANK	
	WYFYYLGTGPEAGLPYGANKDGIIWVATEGALNT	WYFYYLGTGPEAGLPYGANKDGIIWVATEGALNTPK	
	WYFYYLGTGPEAGLPYGANKDGIIWVATEGALNT	WYFYYLGTGPEAGLPYGANKDGIIWVATEGALNTPKDHIGTR	
	WYFYYLGTGPEASLPYGANK	WYFYYLGTGPEASLPYGANK	
	YCALAPNMMVTNNTFTLK	YCALAPNMMVTNNTFTLK	
	YFSGAMDTTSYR	YFSGAMDTTSYR	
	YLGTGPEAGLPYGANK	YLGTGPEAGLPYGANK	
	YLVQQESPFVMMSAPPAQYELK	YLVQQESPFVMMSAPPAQYELK	
	YMNSQGLLPPK	YMNSQGLLPPK	
	YMSALNHTK	YMSALNHTK	
	YNENGTITDAVDCALDPLSETK	YNENGTITDAVDCALDPLSETK	
	YNLPTMCDIR	YNLPTMCDIR	
	YNSASFSTFK	YNSASFSTFK	

	YPANSIVCR	YPANSIVCR	
	YPQVNGLTSIK	YPQVNGLTSIK	
	YSQLDEEQPMEID	YSQLDEEQPMEID	
	YSTLQGPPGTGK	YSTLQGPPGTGK	
	YTMADLVYALR	YTMADLVYALR	
	YTQLCQYLNTLTLAVPYNMR	YTQLCQYLNTLTLAVPYNMR	
	YVDNNFCGPDGYPLECIK	YVDNNFCGPDGYPLECIK	
	YVDNNFCGPDGYPLECIKDLLAR	YVDNNFCGPDGYPLECIKDLLAR	
	YVLMDGSIIQFPNTYLEGSVR	YVLMDGSIIQFPNTYLEGSVR	
	YVQIPTTCANDPVGFTLK	YVQIPTTCANDPVGFTLK	
	YYLGTGPEAGLPYGANK	YYLGTGPEAGLPYGANK	
	AIASEFSSLPSYAAFATAQEAYEQAVANGDSEVVL	AIASEFSSLPSYAAFATAQEAYEQAVANGDSEVVLK	
	AIASEFSSLPSYAAFATAQEAYEQAVANGDSEVVL	AIASEFSSLPSYAAFATAQEAYEQAVANGDSEVVLKK	
	ALTGIAVEQDKNTQEVFAQVKQIYK	ALTGIAVEQDKNTQEVFAQVKQIYK	
	AMPNMLR	AMPNMLR	
	ATYKPNTWCIR	ATYKPNTWCIR	
	AWIGFDVEGCHATR	AWIGFDVEGCHATR	
	AYKIEELFYSYATHSDK	AYKIEELFYSYATHSDK	
	CAYWVPR	CAYWVPR	
	CEESSAKSASVYYSQLMCQPILLLDQALVSDVGDS	CEESSAKSASVYYSQLMCQPILLLDQALVSDVGDSAEVAVK	
	CSAYTVELGTEVNEFACVVADAVIK	CSAYTVELGTEVNEFACVVADAVIK	
	CVPQADVEWK	CVPQADVEWK	
	CYGVSPTKLNDLCFTNVYADSFVIR	CYGVSPTKLNDLCFTNVYADSFVIR	
	CYGVSPTKLNDLCFTNVYADSFVIRGDEVR	CYGVSPTKLNDLCFTNVYADSFVIRGDEVR	
	DAPAHISTIGVCSMTDIAKKPTETICAPLTVFFDGR	DAPAHISTIGVCSMTDIAKKPTETICAPLTVFFDGR	
	DEDDNLIDSYFVVK	DEDDNLIDSYFVVK	
	DISTEIYQAGSTPCNGVEGFNCYFPLQSYGFQPTN	DISTEIYQAGSTPCNGVEGFNCYFPLQSYGFQPTNGVGYQPYR	
	DLSLQFK	DLSLQFK	
	DTTEAFEK	DTTEAFEK	
	DVLECNVK	DVLECNVK	
	DWSYSGQSTQLGIEFLKR	DWSYSGQSTQLGIEFLKR	
	EAVGTNLPLQLGFSTGVNLVAVPTGYVDTPNNTDF	EAVGTNLPLQLGFSTGVNLVAVPTGYVDTPNNTDFSR	
	EDLKFPR	EDLKFPR	
	EETGLLMPLK	EETGLLMPLK	
	EGQINDMILSLLSKGR	EGQINDMILSLLSKGR	
	EGVEFLR	EGVEFLR	
	EIKESVQTFFK	EIKESVQTFFK	

	EILVTYNCCDDDYFNK	EILVTYNCCDDDYFNK	
	EILVTYNCCDDDYFNKK	EILVTYNCCDDDYFNKK	
	ELHLSWEVGKPRPPLNR	ELHLSWEVGKPRPPLNR	
	ELLVYAADPAMHAASGNLLLDK	ELLVYAADPAMHAASGNLLLDK	
	FASVYAWNRK	FASVYAWNRK	
	FNGIGVTQNVLYENQKLIANQFNSAIGK	FNGIGVTQNVLYENQKLIANQFNSAIGK	
	FNVAITR	FNVAITR	
	FPVLHDIGNPK	FPVLHDIGNPK	
	FRIDGDMVPHISR	FRIDGDMVPHISR	
	FTDGVCLFWNCNVDR	FTDGVCLFWNCNVDR	
	FTDGVCLFWNCNVDRYPANSIVCR	FTDGVCLFWNCNVDRYPANSIVCR	
	FVTDTPK	FVTDTPK	
	FYFYTSK	FYFYTSK	
	FYGGWHNMLK	FYGGWHNMLK	
	GATVVIGTSK	GATVVIGTSK	
	GLQPSVGPK	GLQPSVGPK	
	GPEQTQGNFGDQELIRQGTDYK	GPEQTQGNFGDQELIRQGTDYK	
	GPHEFCSQHTMLVKQGDDYVYLPYPDPSR	GPHEFCSQHTMLVKQGDDYVYLPYPDPSR	
	GTAVMSLK	GTAVMSLK	
	GTAVMSLKEGQINDMILSLLSK	GTAVMSLKEGQINDMILSLLSK	
	GVYCCREHEHEIAWYTER	GVYCCREHEHEIAWYTER	
	GYGCSCDQLREPMLQ	GYGCSCDQLREPMLQ	
	HAFHTPAFDK	HAFHTPAFDK	
	HFFFAQDGNAAISDYDYYR	HFFFAQDGNAAISDYDYYR	
	HLIPLMYK	HLIPLMYK	
	HLIPLMYKGLPWNVVR	HLIPLMYKGLPWNVVR	
	HPNQEYADVFHLYLQYIR	HPNQEYADVFHLYLQYIR	
	HWPQIAQFAPSASAFFGMSRIGMEVTPSGTWLTY	HWPQIAQFAPSASAFFGMSRIGMEVTPSGTWLTYTGAIK	
	IDGDMVPHISR	IDGDMVPHISR	
	IEELFYSYATHSDK	IEELFYSYATHSDK	
	IEELFYSYATHSDKFTDGVCLFWNCNVDR	IEELFYSYATHSDKFTDGVCLFWNCNVDR	
	IKIVQMLSDTLK	IKIVQMLSDTLK	
	ILNNLGVDIAANTVIWDYK	ILNNLGVDIAANTVIWDYK	
	ILNNLGVDIAANTVIWDYKR	ILNNLGVDIAANTVIWDYKR	
	IQDSLSSTASALGKLQDVVNQNAQALNTLVK	IQDSLSSTASALGKLQDVVNQNAQALNTLVK	
	IQPGQTFSVLACYNGSPSGVYQCAMRPNFTIK	IQPGQTFSVLACYNGSPSGVYQCAMRPNFTIK	
	IVQMLSDTLK	IVQMLSDTLK	

	KAVFISPYNSQNAVASK	KAVFISPYNSQNAVASK	
	KDAPYIVGDVVQEGVLTAVVIPTK	KDAPYIVGDVVQEGVLTAVVIPTK	
	KDWYDFVENPDILR	KDWYDFVENPDILR	
	KFLPFQQFGR	KFLPFQQFGR	
	KGAWNIGEQK	KGAWNIGEQK	
	KLDGFMGR	KLDGFMGR	
	KLHDELTGHMLDMYSVMLTNDNTSR	KLHDELTGHMLDMYSVMLTNDNTSR	
	LALGGSVAIK	LALGGSVAIK	
	LDGFMGR	LDGFMGR	
	LEGYAFEHIVYGDFSHSQLGGLHLLIGLAK	LEGYAFEHIVYGDFSHSQLGGLHLLIGLAK	
	LFAAETLK	LFAAETLK	
	LHDELTGHMLDMYSVMLTNDNTSR	LHDELTGHMLDMYSVMLTNDNTSR	
	LIEYTDFATSACVLAAECTIFK	LIEYTDFATSACVLAAECTIFK	
	LISMMGFK	LISMMGFK	
	LITGRLQSLQTYVTQQLIR	LITGRLQSLQTYVTQQLIR	
	LLGVGGKPCIK	LLGVGGKPCIK	
	LNDLCFTNVYADSFVIRGDEVRQIAPGQTGK	LNDLCFTNVYADSFVIRGDEVRQIAPGQTGK	
	LNEEIAIILASFSASTSAFVETVK	LNEEIAIILASFSASTSAFVETVK	
	LNVGDYFVLTSHTVMPLSAPTLVPQEHYVR	LNVGDYFVLTSHTVMPLSAPTLVPQEHYVR	
	LQSLQTYVTQQLIRAAEIRASANLAATK	LQSLQTYVTQQLIRAAEIRASANLAATK	
	LRSDVLLPLTQYNR	LRSDVLLPLTQYNR	
	LSFKELLVYAADPAMHAASGNLLLDK	LSFKELLVYAADPAMHAASGNLLLDK	
	LTLGVYDYLVSTQEFR	LTLGVYDYLVSTQEFR	
	LYYDSMSYEDQDALFAYTK	LYYDSMSYEDQDALFAYTK	
	LYYDSMSYEDQDALFAYTKR	LYYDSMSYEDQDALFAYTKR	
	MVSLLSVLLSMQGAVDINK	MVSLLSVLLSMQGAVDINK	
	NKCVNFNFNGLTGTGVLTESNKK	NKCVNFNFNGLTGTGVLTESNKK	
	NLQEFKPR	NLQEFKPR	
	NRDVDTDFVNEFYAYLR	NRDVDTDFVNEFYAYLR	
	NSIDAFK	NSIDAFK	
	NTCDGTTFTYASALWEIQQVVDADSK	NTCDGTTFTYASALWEIQQVVDADSK	
	QLLFVVEVVDK	QLLFVVEVVDK	
	QLSSNFGAISSVLNDILSRLDK	QLSSNFGAISSVLNDILSRLDK	
	QLSSNFGAISSVLNDILSRLDKVEAEVQIDR	QLSSNFGAISSVLNDILSRLDKVEAEVQIDR	
	QMSCAAGTTQTACTDDNALAYYNTTK	QMSCAAGTTQTACTDDNALAYYNTTK	
	QRRPQGLPNNTASWFTALTQHGK	QRRPQGLPNNTASWFTALTQHGK	
	QRRPQGLPNNTASWFTALTQHGKEDLK	QRRPQGLPNNTASWFTALTQHGKEDLK	

	QWLPTGTLLVDSDLNDFVSDADSTLIGDCATVHTA	QWLPTGTLLVDSDLNDFVSDADSTLIGDCATVHTANK	
	QYGDCLGDIAARDLICAQK	QYGDCLGDIAARDLICAQK	
	RFDNPVLPFNDGVYFASTEKSNIIR	RFDNPVLPFNDGVYFASTEKSNIIR	
	RGPEQTQGNFGDQELIRQGTDYK	RGPEQTQGNFGDQELIRQGTDYK	
	RHTFSNYQHEETIYNLLK	RHTFSNYQHEETIYNLLK	
	RISNCVADYSVLYNSASFSTFK	RISNCVADYSVLYNSASFSTFK	
	RLISMMGFK	RLISMMGFK	
	RNIKPVPEVK	RNIKPVPEVK	
	RNVIPTITQMNLK	RNVIPTITQMNLK	
	RSFIEDLLFNKVTLADAGFIK	RSFIEDLLFNKVTLADAGFIK	
	RSFYVYANGGK	RSFYVYANGGK	
	RVDWTIEYPIIGDELK	RVDWTIEYPIIGDELK	
	SAFYILPSIISNEKQEILGTVSWNLR	SAFYILPSIISNEKQEILGTVSWNLR	
	SASVYYSQLMCQPILLLDQALVSDVGDSAEVAVK	SASVYYSQLMCQPILLLDQALVSDVGDSAEVAVK	
	SDGTGTIYTELEPPCR	SDGTGTIYTELEPPCR	
	SFDLGDELGTDPYEDFQENWNTK	SFDLGDELGTDPYEDFQENWNTK	
	SFIEDLLFNKVTLADAGFIKQYGDCLGDIAAR	SFIEDLLFNKVTLADAGFIKQYGDCLGDIAAR	
	SFTVEKGIYQTSNFR	SFTVEKGIYQTSNFR	
	SHFAIGLALYYPSAR	SHFAIGLALYYPSAR	
	SKCEESSAKSASVYYSQLMCQPILLLDQALVSDVG	SKCEESSAKSASVYYSQLMCQPILLLDQALVSDVGDSAEVAVK	
	SMWSFNPETNILLNVPLHGTILTRPLLESELVIGAVI	SMWSFNPETNILLNVPLHGTILTRPLLESELVIGAVILR	
	SMWSFNPETNILLNVPLHGTILTRPLLESELVIGAVI	SMWSFNPETNILLNVPLHGTILTRPLLESELVIGAVILRGHLR	
	SQMEIDFLELAMDEFIER	SQMEIDFLELAMDEFIER	
	SSQAWQPGVAMPNLYK	SSQAWQPGVAMPNLYK	
	SYLTPGDSSSGWTAGAAAYYVGYLQPRTFLLK	SYLTPGDSSSGWTAGAAAYYVGYLQPRTFLLK	
	TEGLCVDIPGIPK	TEGLCVDIPGIPK	
	TFYVLPNDDTLRVEAFEYYHTTDPSFLGR	TFYVLPNDDTLRVEAFEYYHTTDPSFLGR	
	TGDLQPLEQPTSEAVEAPLVGTPVCINGLMLLEIK	TGDLQPLEQPTSEAVEAPLVGTPVCINGLMLLEIK	
	TGDLQPLEQPTSEAVEAPLVGTPVCINGLMLLEIKD	TGDLQPLEQPTSEAVEAPLVGTPVCINGLMLLEIKDTEK	
	TIGPDMFLGTCRRCPAEIVDTVSALVYDNK	TIGPDMFLGTCRRCPAEIVDTVSALVYDNK	
	TLATHGLAAVNSVPWDTIANYAKPFLNK	TLATHGLAAVNSVPWDTIANYAKPFLNK	
	TNCCRFQEKDEDDNLIDSYFVVK	TNCCRFQEKDEDDNLIDSYFVVK	
	TNVYLAVFDKNLYDKLVSSFLEMK	TNVYLAVFDKNLYDKLVSSFLEMK	
	TQFNYYK	TQFNYYK	
	TSVDCTMYICGDSTECSNLLLQYGSFCTQLNR	TSVDCTMYICGDSTECSNLLLQYGSFCTQLNR	
	TTVASLINTLNDLNETLVTMPLGYVTHGLNLEEAAR	TTVASLINTLNDLNETLVTMPLGYVTHGLNLEEAAR	
	VCVDTVR	VCVDTVR	

	VDWTIEYPIIGDELK	VDWTIEYPIIGDELK	
	VEGCMVQVTCGTTTLNGLWLDDVVYCPR	VEGCMVQVTCGTTTLNGLWLDDVVYCPR	
	VFTTVDNINLHTQVVDMSMTYGQQFGPTYLDGAD	VFTTVDNINLHTQVVDMSMTYGQQFGPTYLDGADVTK	
	VGILCIMSDR	VGILCIMSDR	
	VIHFGAGSDKGVAPGTAVLR	VIHFGAGSDKGVAPGTAVLR	
	VITGLHPTQAPTHLSVDTK	VITGLHPTQAPTHLSVDTK	
	VLSNLNLPGCDGGSLYVNK	VLSNLNLPGCDGGSLYVNK	
	VSAKPPPGDQFK	VSAKPPPGDQFK	
	VTIDYTEISFMLWCK	VTIDYTEISFMLWCK	
	VTLADAGFIKQYGDCLGDIAAR	VTLADAGFIKQYGDCLGDIAAR	
	VTLADAGFIKQYGDCLGDIAARDLICAQK	VTLADAGFIKQYGDCLGDIAARDLICAQK	
	VVFVLWAHGFELTSMK	VVFVLWAHGFELTSMK	
	VVISSDVLVNN	VVISSDVLVNN	
	VVVLSFELLHAPATVCGPKK	VVVLSFELLHAPATVCGPKK	
	VWTLMNVLTLVYK	VWTLMNVLTLVYK	
	VYPIILR	VYPIILR	
	WADNNCYLATALLTLQQIELK	WADNNCYLATALLTLQQIELK	
	WDLIISDMYDPK	WDLIISDMYDPK	
	WQLALSK	WQLALSK	
	YDFTEER	YDFTEER	
	YFDCYDGGCINANQVIVNNLDK	YFDCYDGGCINANQVIVNNLDK	
	YKLEGYAFEHIVYGDFSHSQLGGLHLLIGLAK	YKLEGYAFEHIVYGDFSHSQLGGLHLLIGLAK	
	YLALYNK	YLALYNK	
	YNYEPLTQDHVDILGPLSAQTGIAVLDMCASLK	YNYEPLTQDHVDILGPLSAQTGIAVLDMCASLK	
	YRIGNYKLNTDHSSSSDNIALLVQ	YRIGNYKLNTDHSSSSDNIALLVQ	
	YWDQTYHPNCVNCLDDR	YWDQTYHPNCVNCLDDR	
	YWEPEFYEAMYTPHTVLQ	YWEPEFYEAMYTPHTVLQ	
		AGGNYNYRYR	
		CEFQFCNDPFLDVYYHKNNK	
		MADSNGTVEELKK	
		ITFVEPSDSTGSNQNGER	
		ITFVGPSDSTDSNQNGER	
		NKCINFNFNGLTGTGVLTESNKK	
		EINRLNEVAK	
		ELTVATSR	
		ICASHQTQTNSPR	
		KANETQALPQR	

		NNISWMESE	
		TFGAGAAL	
		TNQFNSAIGK	
		CDIKNLPK	
		EIDRLNQVAK	
		LDDKNPNFK	
		QLQQSMSSSDSTQA	
		RVDFCGK	
		DGIIWVATEGALNTPKG	
		IIWVATEGALNTPK	
		MAGNGCDAALALLLLNR	
		DGILWVATEGALNTPK	
		ITFGGPSDSTGSNQDGER	
		RPQGLPDNTASWF	
		LTYTGAIK	
		WYFYYLGTGPEAGLPY	
		GFYAEGSRGGSEASSR	
		RGPEQTQGNFGDQELTR	
		FLPFQQFSR	
		GWIFGTTLDPK	
		AGDGGDAALALLLLDR	
		AYIVTQAFGR	
		MAGDGGDAALALLLLDRLNQLESK	
		SMGTSPTRMAGNGGDAALALLLLDR	
		FPQGQGVPINTNSSR	
		GPEQTQGNFGDQELTR	
		QGTDYKHWPQIAQFAPSASAFF	
		ALALLLLDR	
		ITFGGPSDSTGSNQDGERSGAR	
		MAGNGGDAAIALLLLDRLNQLESK	
		ALTGISVEQDKNTQEVFAQVK	
		HWPQIAQFAPSASAFFGMSRICMEVTPSGT	
		YWPQIAQFAPSASAFF	
		FDNPVLPFNDGVYFSATEK	
		GPEQTQGNFGDQELLR	
		GWLFGTTLDSK	
		ITFGGPSDSTGSNENGER	

		LDDKDPNFKDQVLLLNK	
		LQDSLSSTASALGK	
		MAGDGGDAALALLLLDR	
		NPANNAALVLQLPQGTTLPK	
		RGPEQTQGNFGDEELIR	
		RGPEQTQGNFGDQELLR	
		SDNGPQNQRDAPR	
		SFLEDLLFNK	
		DDQIGYYR	
		DDQIGYYRR	
		DGILWVATEGALNTPKDHIGTR	
		GQGVPINTNSSR	
		GQGVPINTNSSRDDQIGYYR	
		LQEVVNQNAQALNTLVK	
		NPANNAAIVLQLPEGTTLPK	
		NSSRNSTPGSSIRTSPGMAGNGGDAALALLLLDR	
		WYFYYLGTGPESGLPYGANK	
		PGNGCDAALALLLLDR	
		YLGTGPESGLPYGANK	
		NPANNAAIVL	
		QQTVTLLPAADLDYFSK	
		RGPEQTQGNFGDQEL	
		NPANDAAIVLQLPQGTTLPK	
		DRLNQLESK	
		TQHGKEDLKFPR	
		AYNVTQAFGGR	
		GPEQTQGNFGDQELIRR	
		GQGVPIDTNSSPDDQIGYYR	
		KQLTVTLLPAADLDDFSK	
		MAGNGNDAALALLLLDR	
		NSSRTSTPGSSK	
		QLTVTLLPAADLDDFSK	
		DLPIEITVATSRTLSYYK	
		GEGVPINTNSSPDDQIGYYR	
		LLPAADLDDFFK	
		MAGDAALALL	
		NPANNAAIVIQLPQGTTLPK	

		NPANNAAIVLQIPQGTTLPK	
		NSIPGSSMGTSPARMA	
		NSTPGSSKPTSPARMAGNGGDAA	
		SDNGPQNLPNAPR	
		SLVPGFNEK	
		SLNVAKSEFDRDAAMQR	
		IGAGICASYQTQTNSPR	
		NLNESLIDLQVL	
		NSTPGSSMGTSPAR	
		AYETQALPQR	
		KAYETQALPQR	
		LNQLGSKMSG	
		NGIIWVATEGALNTPK	
		LNQLESKMAGK	
		ITFGGPSDSTGSNQNGGAR	
		RATRRIPGGDGK	
		VTSPARMAGN	
		ITFGGPSDSTGSNQNGQR	
		LNKVEAEVQIDR	
