# Supplementary material for: Catching the Wave: Detecting Strain-Specific SARS-CoV-2 Peptides in Clinical Samples Collected during Infection Waves from Diverse Geographical Locations
Source: Viruses. 2022 Oct 7;14(10):2205. doi: 10.3390/v14102205 (PMC9609567; doi:10.3390/v14102205)
Supplement: Supplementary file 1 [file viruses-14-02205-s001.zip › Supplementary Data S5.pdf]

PEPTIDE	PROTEIN	PANGO LINEAGE	FIRST OBSERVED	BLASTP IDENTITY (WT)
**GQGVPINTNSSR**	N	P.1.1	2021-02-22	88
**ITFGGPSDSTGSNQNGGAR**	N	BA.1	2021-11-11	86.36
**SMGTSPTRMAGNGGDAALALLLLDR**	N	B.1.617.2	2021-04-17	86.67
**KAYETQALPQR**	N	B.1.36	2020-08	91
**AYETQALPQR**	N	B.1.617.2	2020-08	80
**GEGVPINTNSSPDDQIGYYR**	N	B.1.1.7	2021-02-16	95
**ITFGGPSDSTGSNQDGER**	N	B.1.1.7	2021-02-27	94.44
**MAGDGGDAALALLLLDR**	N	B.1.1.7	2021-02-27	85.714
**ADETQALPQR**	N	B.1.1.7	2019-12-30	100
**RGPEQTQGNFGDQELTR**	N	B.1.1.33	2020-05	94.118
**GPEQTQGNFGDQELTR**	N	B.1.1.33	2020-05	93.75
**DGIIWVATEGALNTPKG**	N	B.1	2021-03-01	100
**QYNVTQAFGR**	N	B.1	2019-10-22	90
**EITVATSR**	M	B	2019-12-30	100
**FDNPVLPFNDGVYFASTEK**	Spike	B	2019-12-30	100
**IAGHHLGR**	M	B	2019-12-30	100
**SNLKPFER**	Spike	B	2019-12-30	100
**VYSTGSNVFQTR**	Spike	B	2019-12-30	100
**DDKDPNFKDQVILLNK**	N	B	2019-12-30	100
**DDQIGYYR**	N	B	2019-12-30	100
**DGIIWVATEGALNTPK**	N	B	2019-12-30	100
**DRLNQLESK**	N	B	2019-12-30	100
**FNGIGVTQNVLYENQK**	Spike	B	2019-12-30	100
**GFQPTNGVGYQPYR**	Spike	B	2019-12-30	100
**GFYAEGSR**	N	B	2019-12-30	100
**GGDAALALLLLDR**	N	B	2019-12-30	100
**GIIWVATEGALNTPK**	N	B	2019-12-30	100
**GPEQTQGNFGDQELIR**	N	B	2019-12-30	100
**GQGVPINTNSSPDDQIGY**	N	B	2019-12-30	100
**GQGVPINTNSSPDDQIGYYR**	N	B	2019-12-30	100
**HWPQIAQFAPSASAFF**	N	B	2019-12-30	100

**HWPQIAQFAPSASAFFGMSR**	N	B	2019-12-30	100
**IGMEVTPSGTWLTYTGAIK**	N	B	2019-12-30	100
**IIWVATEGALNTPK**	N	B	2019-12-30	100
**ITFGGPSDSTGSNQNGER**	N	B	2019-12-30	100
**KQQTVTLLPAADLDDFSK**	N	B	2019-12-30	100
**LDDKDPNFK**	N	B	2019-12-30	100
**LDDKDPNFKDQVILLNK**	N	B	2019-12-30	100
**LGSPLSLNMAR**	NS9b	B	2019-12-30	100
**LGTGPEAGLPYGANK**	N	B	2019-12-30	100
**LIANQFNSAIGK**	Spike	B	2019-12-30	100
**LQDVVNQNAQALNTLVK**	Spike	B	2019-12-30	100
**LQSLQTYVTQQLIR**	Spike	B	2019-12-30	100
**MAGNGGDAALALLLLDR**	N	B	2019-12-30	100
**MAGNGGDAALALLLLDRLNQLESK**	N	B	2019-12-30	100
**NPANNAAIVLQLPQGT**	N	B	2019-12-30	100
**NSSPDDQIGYYR**	N	B	2019-12-30	100
**NTNSSPDDQIGYYR**	N	B	2019-12-30	100
**QGTDYKHWPQIAQFAPSASAFFGMSR**	N	B	2019-12-30	100
**QKRTATKAYNVTQAFGR**	N	B	2019-12-30	100
**QLQQSMSSADSTQA**	N	B	2019-12-30	100
**QQTVTLLPAADLDDFSK**	N	B	2019-12-30	100
**RPQGLPNNTASW**	N	B	2019-12-30	100
**RPQGLPNNTASWF**	N	B	2019-12-30	100
**RPQGLPNNTASWFTALTQHGK**	N	B	2019-12-30	100
**RPQGLPNNTASWFTALTQHGKEDLK**	N	B	2019-12-30	100
**TALTQHGKEDLK**	N	B	2019-12-30	100
**TALTQHGKEDLKFPR**	N	B	2019-12-30	100
**TATKAYNVTQAFGR**	N	B	2019-12-30	100
**TQHGKEDLKFPR**	N	B	2019-12-30	100
**VAGDSGFAAYSR**	M	B	2019-12-30	100
**WVATEGALNTPK**	N	B	2019-12-30	100

**WYFYYLGTGPEAGLPYGANK**	N	B	2019-12-30	100
**YLGTGPEAGLPYGANK**	N	B	2019-12-30	100
**YYLGTGPEAGLPYGANK**	N	B	2019-12-30	100
**NPANNAAIVIQLPQGTTLPK**	N	B	2019-12-30	95
**NSTPGSSMGTSPAR**	N	B	2019-12-30	91.667
**ASANLAATK**	Spike	B	2019-12-30	100
**AYNVTQAFGR**	N	B	2019-12-30	100
**CDIKDLPK**	M	B	2019-12-30	100
**KADETQALPQR**	N	B	2019-12-30	100
**KKADETQALPQR**	N	B	2019-12-30	100
**KSNLKPFER**	Spike	B	2019-12-30	100
**LTYTGAIK**	N	B	2019-12-30	100
**LVDPQIQLAVTR**	NS9b	B	2019-12-30	50
**MSECVLGQSK**	Spike	B	2019-12-30	100
**PAADLDDFSK**	N	B	2019-12-30	100
**SFIEDLLFNK**	Spike	B	2019-12-30	100
**VATEGALNTPK**	N	B	2019-12-30	100
**VGGNYNYLYR**	Spike	B	2019-12-30	100
**VTLADAGFIK**	Spike	B	2019-12-30	100
**PGNGCDAALALLLLDR**	N	AY.117	2021-06-21	93.33
